# A high-quality genome assembly for the endangered golden snub-nosed monkey (*Rhinopithecus roxellana*)

**DOI:** 10.1093/gigascience/giz098

**Published:** 2019-08-22

**Authors:** Lu Wang, Jinwei Wu, Xiaomei Liu, Dandan Di, Yuhong Liang, Yifei Feng, Suyun Zhang, Baoguo Li, Xiao-Guang Qi

**Affiliations:** 1 Shaanxi Key Laboratory for Animal Conservation, College of Life Sciences, Northwest University, Xi'an, 710069, China; 2 Center for Excellence in Animal Evolution and Genetics, Chinese Academy of Sciences, Kunming, 650223, China

**Keywords:** high-quality, *Rhinopithecus roxellana*, genome assembly, annotation, BioNano optical maps

## Abstract

**Background:**

The golden snub-nosed monkey (*Rhinopithecus roxellana*) is an endangered colobine species endemic to China, which has several distinct traits including a unique social structure. Although a genome assembly for *R. roxellana* is available, it is incomplete and fragmented because it was constructed using short-read sequencing technology. Thus, important information such as genome structural variation and repeat sequences may be absent.

**Findings:**

To obtain a high-quality chromosomal assembly for *R. roxellana qinlingensis*, we used 5 methods: Pacific Bioscience single-molecule real-time sequencing, Illumina paired-end sequencing, BioNano optical maps, 10X Genomics link-reads, and high-throughput chromosome conformation capture. The assembled genome was ∼3.04 Gb, with a contig N50 of 5.72 Mb and a scaffold N50 of 144.56 Mb. This represented a 100-fold improvement over the previously published genome. In the new genome, 22,497 protein-coding genes were predicted, of which 22,053 were functionally annotated. Gene family analysis showed that 993 and 2,745 gene families were expanded and contracted, respectively. The reconstructed phylogeny recovered a close relationship between *R. rollexana* and *Macaca mulatta*, and these 2 species diverged ∼13.4 million years ago.

**Conclusion:**

We constructed a high-quality genome assembly of the Qinling golden snub-nosed monkey; it had superior continuity and accuracy, which might be useful for future genetic studies in this species and as a new standard reference genome for colobine primates. In addition, the updated genome assembly might improve our understanding of this species and could assist conservation efforts.

## Background

**Figure 1:**
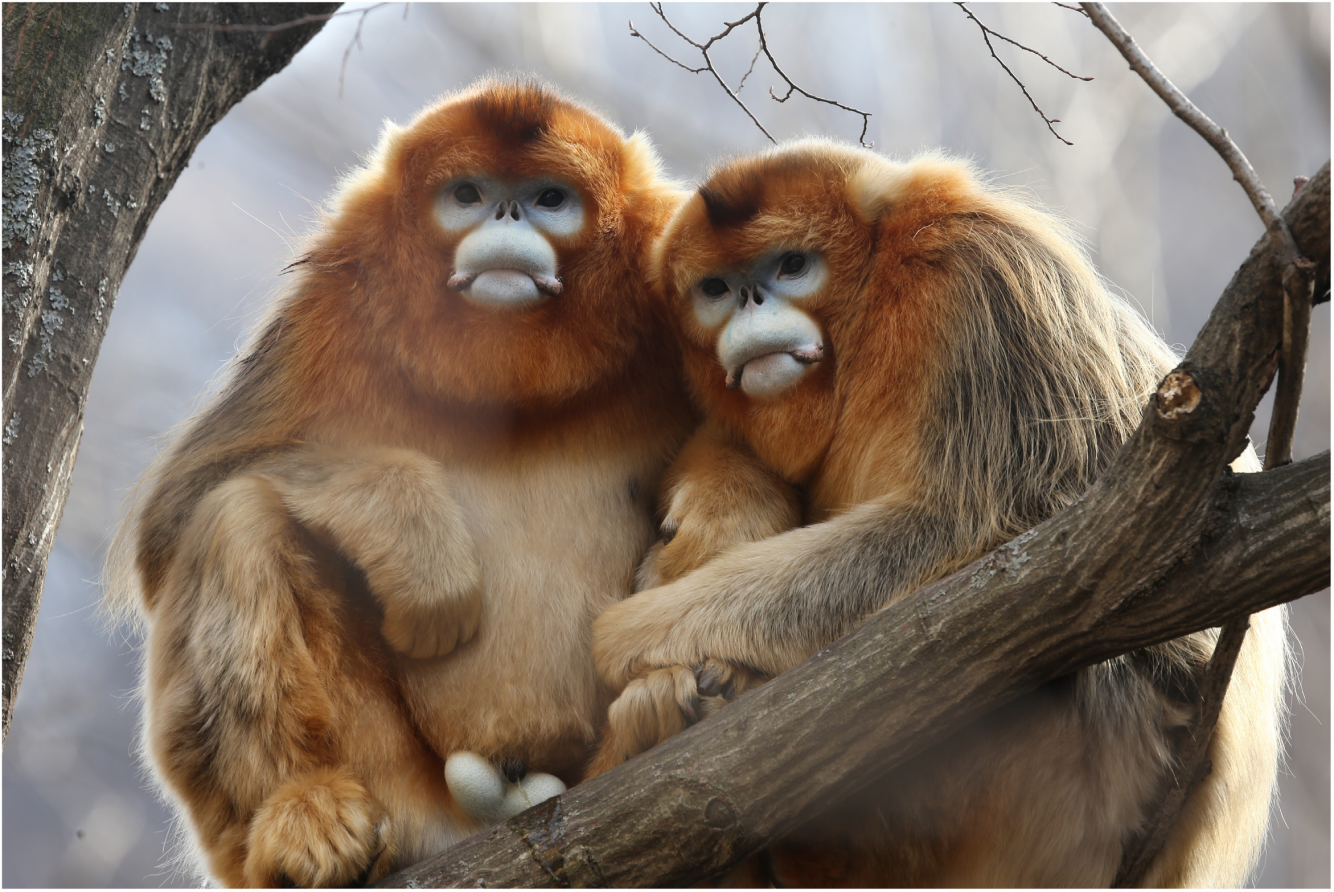
Image of *R. roxellana*, taken on Qinling Mountain, China.

The snub-nosed monkeys (genus *Rhinopithecus*) consist of 5 endangered species narrowly restricted to China and Vietnam [1]. Of those, the golden snub-nosed monkey (*Rhinopithecus roxellana*, NCBI:txid61622), also known as the Sichuan snub-nosed monkey, has the northernmost distribution of all Asian colobine species; this monkey is found only in 3 isolated regions in central and northwest China (the Sichuan, Gansu, Shaanxi, and Hubei Provinces) [2, 3]. The golden snub-nosed monkey is characterized by several distinctive traits, including golden fur, a blue facial color, an odd-shaped nose, and folivory (Fig. 1). In addition, the species has a unique multilevel social system; such complex systems are found only in a few mammals, including humans [4]. Therefore, the Qinling golden snub-nosed monkey is an ideal model for the analysis of social structure evolution in primates and may also provide opportunities to investigate evolutionary and socio-anthropological patterns of human society.

Based on morphological variations and discontinuous distributions, *R. roxellana* is distinguished into 3 subspecies: *R. roxellana roxellana* from the Minshan Mountain in the Sichuan and Gansu Provinces, *R. roxellana qinlingensis* from the Qinling Mountain in Shaanxi Province, and *R. roxellana hubeiensis* from Shennongjia Mountain in Hubei Province [3]. Recent studies of *R. roxellana* have focused on behavioral dynamics, population history, and social systems [5–7]. To date, only a single genome assembly is available for the golden snub-nosed monkey. This assembly, published in 2014, was derived from short sequencing reads generated by the Illumina HiSeq 2000 platform [8]. Several studies have been published based on these data, including analyses of the folivorous dietary adaptations of *R. roxellana* and its evolutionary history [8–10]. Despite the utility of these previously published data, much relevant information, including structural variations and repeat sequences, is largely absent or unreliable owing to the incomplete and fragmented genome assembly [11, 12].

Owing to advances in sequencing technology, it is now possible to obtain high-quality genome assemblies that can provide new insights in organismal research. Indeed, many previously unreported transposable elements and specific genes in maize were identified using an improved reference genome [13]. By combining new sequencing approaches, Seo et al. [11] discovered clinically relevant structural variants and previously unreported genes in the updated human genome. New sequencing technologies, including Pacific Bioscience's (PacBio's) single-molecule real-time (SMRT) sequencing, BioNano optical mapping, and high-throughput chromosome conformation capture (Hi-C)–based chromatin interaction maps, have been used in several species closely related to humans, including gorillas (*Gorilla gorilla gorilla*) [14], chimpanzees (*Pan troglodytes*) [15], and Sumatran orangutans (*Pongo abelii*) [15], as well as in other species, including the domestic goat (*Capra hircus*) [16]. Importantly, it was estimated that 87% of the missing reference exons and incomplete gene models were recovered using the new gorilla assembly [14]. In addition, several novel genes expressed in the brain were identified using the new orangutan assembly, and complete immune genes with longer repetitive structures were identified in the updated goat genome [16]. However, the *R. roxellana* genome has not yet been updated using new sequencing approaches, slowing progress towards a better understanding of this endangered species.

Here, we report a greatly improved assembly of the reference genome for *R. roxellana* generated by a combination of 5 technologies: SMRT sequencing from PacBio, HiSeq paired-end sequencing from Illumina (HiSeq), BioNano optical maps (BioNano), 10X Genomics link-reads (10X Genomics), and Hi-C. Our results represent the first colobine genome sequenced and assembled with both long reads and short reads. This updated genome assembly may allow us to further investigate *R. roxellana*, providing new opportunities to analyze evolutionary history and to identify genetic changes associated with the development of specific traits in this species. Such analyses may provide insights helpful for the conservation of this endangered primate. In addition, this genome, which has superior continuity and accuracy, will act as a new reference genome for colobine primates.

## Data Description

### Sample collection and sequencing

The animal used for sequencing was an adult male *R. roxellana qinlingensis* from Qinling Mountain, who died of natural causes and then was stored shortly after death in an ultra-cold storage freezer at Louguantai Breeding Centre, Xi'an, Shaanxi Province, China. Total genomic DNA was extracted from the heart tissue. To acquire a high-quality genome assembly, we combined 5 sequencing methods. Initially, PacBio SMRT sequencing was performed on the SEQUEL platform following the manufacturer's instructions. After quality control, during which subreads shorter than 500 bp were removed, 304.84 Gb clean long reads (95.86× coverage) remained. The N50 length of the PacBio reads was 16.69 kb. Simultaneously, paired-end sequencing was performed using an Illumina NovaSeq 6000 platform, with an insert size of 350 bp. Then those short reads were filtered using the SOAPdenovo2 software [17], removing reads with adapters, contaminations, >10% unknown bases (N), or low quality. After filtering, 422.00 Gb clean reads remained (133.12× coverage). A high-quality optical genome map was also constructed with the Irys platform (BioNano Genomics). The N50 length of the molecules used for optical mapping was 338 kb. The average BioNano optimal marker density examined was 11.66 per 100 kb, while the average marker density was 12.62 per 100 kb for the predicted map based on the assembled contigs. Thus, the observed BioNano map was consistent with the predicted map. The BioNano map generated 463.75 Gb of large DNA molecules. Next, 10X genomic linked-reads sequencing was performed on an Illumina Hiseq Xten platform, generating 340.90 Gb clean reads (109.56× coverage). Finally, a Hi-C library was prepared and sequenced with an Illumina NovaSeq 6000 platform to produce a chromosome-scale scaffolding of the genome assembly. Adapter sequences and low-quality reads were discarded using Cutadapt v1.0 [18] with the parameters “-e 0.1 -O 5 -m 100 –n 2 –pair-filter = both”, yielding 307.90 Gb clean data (97.77× coverage). Detailed sequencing statistics are given in Table   1.

**Table 1:** Reads generated by the 5 sequencing methods

Paired-end libraries	Insert size (bp)	Total clean data (Gb)	Read length (bp)	Sequence coverage (×)
Illumina	350	422.00	150	133.12
PacBio	20 k	304.84	n/a	95.86
10X Genomics	500−700	340.90	150	109.56
BioNano	n/a	463.75	n/a	n/a
Hi-C	350	307.90	n/a	97.77
Total	n/a	1,839.39	n/a	582.15

Note: The sequence coverage was calculated based on an estimated genome size of 3.18 Gb. n/a: not applicable.

### *De novo* assembly of the *R. roxellana* genome

An estimation of genome size would increase our understanding of *R. roxellana* and the challenges in sequencing it. Thus, we estimated the size of the *R. roxellana* genome as *G* = (*K*_total_ − *K*_error_)/*D*, where *G* represents genome size, *K*_total_ represents the total number of *k*-mers, *K*_error_ represents the number of *k*-mers with sequencing errors, and *D* indicates the *k*-mer depth. We generated 109,210,004,556 *k*-mers, 1,159,024,556 of which had sequencing errors. The peak *k*-mer depth was 34. Thus, the genome size of *R. roxellana* was estimated to be ∼3.18 Gb. The distribution of *k*-mer frequencies is given in Supplementary Fig. S1.

The *de novo* assembly of the newly sequenced *R. roxellana* genome was performed in 4 progressive stages. First, long reads obtained from the PacBio platform were assembled as follows: detection of overlap and read correction, detection of overlap between pairs of corrected reads, and string graph construction. Assembly of the PacBio long reads was performed using FALCON (version 0.4.0, Falcon, RRID:SCR_016089) [19] with the parameter set “length_cutoff = 5000, length_cutoff_pr = 5000, pa_HPCdaligner_option = -v -B128 -e.70 -k14 -h128 -l2000 -w8 -T8 -s700, ovlp_HPCdaligner_option = -v -B128 -e.96 -k16 -h480 -l1500 -w8 -T16 -s700”. Next, the assembled PacBio contigs were polished using Quiver (SMRTLink version 5.1.0) with PacBio long reads [20], and also the contig assembly was corrected by Pilon-1.18 (java -Xmx500G -jar pilon-1.18.jar –diploid –threads 30) with Illumina short reads [21]. The contig N50 of the initial assembly was 4.74 Mb (Supplementary Table S1). Using the initial genome assembly, SSPACE-LongRead v1–1 [22] was implemented for getting a longer scaffold by processing PacBio long reads and the initial genome assembly with the command “perl SSPACE-LongRead.pl -c <contig-sequences> -p <pacbio-reads>”. This procedure generated a genome assembly with scaffold N50 of 7.81 Mb (Supplementary Table S2). The remaining gaps in the assembly were closed using the PBjelly module in the PBSuite (version 15.8.24) [23] with default settings. Thus, at the end of the first stage, the genome assembly had a contig N50 of 5.72 Mb and a scaffold N50 of 8.20 Mb (Supplementary Table S3).

In the second stage, the BioNano molecules were filtered, requiring a minimum length of 150 kb and minimum of 9 labels per molecule. Then, a genome map was assembled *de novo* with IrysView (version 2.3; BioNano Genomics), based on the optically mapped molecules. The assembled PacBio scaffolds were input into hybridScaffold [24]. In brief, the hybrid scaffolding process included the alignment of the PacBio scaffolds against the BioNano genome maps, followed by the identification and resolution of conflicting alignments. At the end of stage 2, the hybrid genome assembly had a scaffold N50 of 9.22 Mb (Supplementary Table S4).

In the third stage, the 10X genomic linked reads were connected with the scaffolds generated in stage 2 to construct super-scaffolds. In brief, we used the Long Ranger basic pipeline [25] to handle the basic read-in and barcode processing of the 10X genomic linked reads. The processed 10X linked reads were then mapped to the hybrid genome assembly from stage 2 with bowtie2 [26], using the command “bowtie2 genome.fa -1 reads1.fq.gz -2 reads2.fq.gz -p 12 -D 1 -R 1 -N 0 -L 28 -i S,0,2.50 –n-ceil L,0,0.02 –rdg 5,10 –rfg 5,10”. We also used a self-against-self (genome.fa-against-genome.fa) BLASTN to generate 2 bed files and merged these files using fragScaff (version 140,324.1) [27], with the parameters “-fs1 '-m 3000 -q 20 -E 30 000 -o 60000', -fs2 '-C 2', -fs3 '-j 1.5 -u 2'” These procedures generated an updated genome assembly with a scaffold N50 of 24.09 Mb (Supplementary Table S5). Subsequently, we corrected errors in the assembly, based on the Illumina short reads, using the Burrows-Wheeler Aligner (BWA, RRID:SCR_010910) [28] and Pilon-1.18 (Pilon, RRID:SCR_014731) [21].

**Figure 2:**
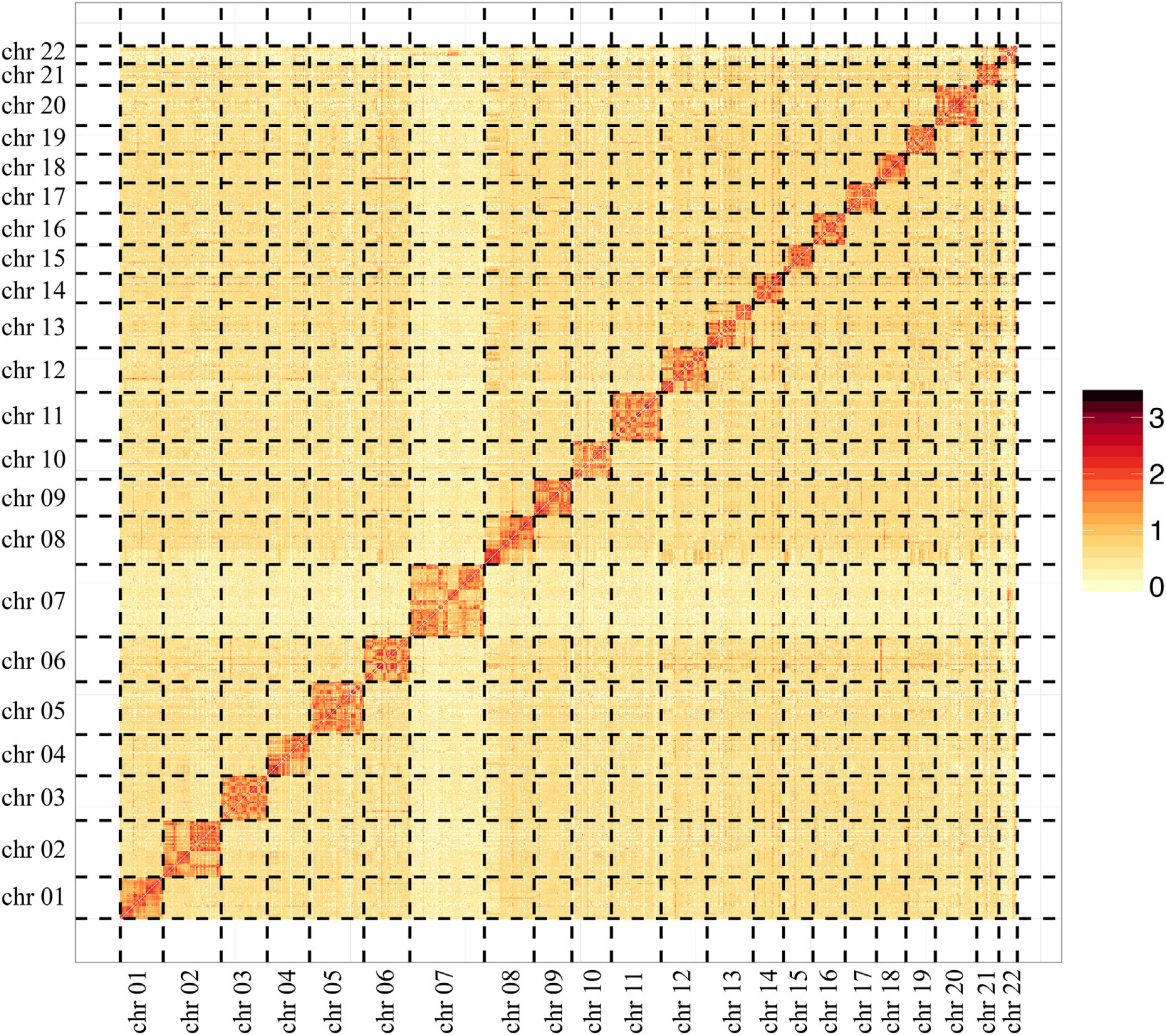
Hi-C heat map of interactions between pairs of chromosomal loci throughout the genome. Hi-C interactions within and among *R. roxellana* chromosomes (Chr 1–Chr 22); interactions were drawn based on the chromatin interaction frequencies between pairs of 100-kb genomic regions (as determined by Hi-C). In principle, darker red cells indicate stronger and more frequent interactions, which in turn imply that the 2 sequences are spatially close.

In the fourth stage, the Hi-C data were used to build chromosome­-level assembly scaffolds. In brief, Hi-C sequencing data were first aligned to the assembled genome using BWA [28]. Scaffolds were then clustered, ordered, and oriented using Lachesis [29], with the parameter set “CLUSTER_MIN_RE_SITES = 1800, CLUSTER_MAX_LINK_DENSITY = 4, and CLUSTER_NONINFORMATIVE_RATIO = 0” This procedure generated 22 accurately clustered and ordered pseudo-chromosomes, with a genome size of 3.04 Gb, a contig N50 of 5.72 Mb, and a scaffold N50 of 144.56 Mb (Table   2). The pseudo-chromosomes were divided into 100-kb bins and the interaction frequencies between pairs of 100-kb genomic regions were determined (Fig. 2).

**Table 2:** Summary of the final *R. roxellana* genome assembly

Category	Contig		Scaffold	
	Length (bp)	Number	Length (bp)	Number
Total	3,038,184,325	6,099	3,038,467,325	3,269
Maximum	30,757,641	n/a	206,558,726	n/a
≥2000 bp	n/a	5,708	n/a	2,879
N50	5,723,610	151	144,559,847	9
N60	4,241,389	211	141,075,955	11
N70	3,173,235	292	135,203,321	14
N80	2,063,823	408	118,350,466	16
N90	896,517	622	83,045,532	19

Note: The “Number” column represents the number of contigs/scaffolds longer than the value of the corresponding category. n/a: not applicable.

### Assessment of the genome newly assembled

We evaluated our newly assembled *R. roxellana* genome against the previously published assembly. The contiguity of our *R. roxellana* genome was 100­-fold greater (contig N50: 5.72 Mb; scaffold N50: 144.56) than the previous version (contig N50: 25.5 kb; scaffold N50: 1.55 Mb) [8]. We also aligned our genome against the previous version using MUMMER (v4.0.0beta2) [30] and identified 6,452 gaps in the previous version that were predicted to be filled by >29.7 Mb of sequence in our new assembly. These filled gaps were mainly located in the intergenic and repetitive regions, with a small fraction of the sequence data annotated as gene regions. Our new assembly also had a higher proportion of repeat sequences (50.82%) than the previous version (46.15%); in particular, the number of LINE (long interspersed elements) transposable elements and tandem repeats was greatly increased (further details are given below, in the “Identification of repeat elements” section). Thus, the newly assembled genome was substantially more complete and continuous. It was likely that the remarkable improvement in contiguity was due to the increased read length, deeper sequencing depth, improved gap assembly, and more sophisticated assembly algorithm.

To assess the accuracy of our genome assembly, we aligned the Illumina short reads to the assembly using BWA [28], with the parameters “-o 1 -i 15”. Approximately 99.17% of the short reads were mapped to the genome assembly. Further investigations indicated that these reads covered ∼99.27% of the total assembly (Supplementary Table S6). Genome assembly accuracy was also measured using the standard variant calling method in samtools [31], with the command “samtools mpileup -q 20 -Q 20 -C 50 -uDEf”. We found a total of 7,690 homozygous non-reference single-nucleotide polymorphisms, which reflected a low homozygous rate (0.0004%), suggesting that our genome assembly was highly accurate (Supplementary Table S7). In addition, we estimated assembly completeness using BUSCO (RRID:SCR_015008) v3.0.2 [32], with the parameters “-i -o -l -m genome -f -t” based on mammalia_odb9 (creation date: 13 February 2016; number of species: 50; number of BUSCOs: 4,104). BUSCO analysis identified 4,104 mammalian BUSCOs in the newly assembled *R. roxellana* genome: 94.0% complete BUSCOs, 2.9% fragmented BUSCOs, and 3.1% missing BUSCOs (Supplementary Table S8). Assembly completeness was also measured using the core eukaryotic gene (CEG)-mapping approach (CEGMA v2.5) [33]. Of the 248 CEGs known from 6 model species, 93.95% (233 of 248) were identified in our new genome assembly. Of these, 220 CEGs were complete and unfragmented, and the remaining 13 were complete but fragmented (Supplementary Table S9). Together, these analyses indicated that our new genome assembly was highly accurate and complete.

### Identification of repeat elements

Repeated sequences account for a large proportion of the total genome. It is thus important to identify repeat elements. Here, we predicted and classified repeat elements both based on homology and *de novo*. In the homology approach, we searched the genome for repetitive DNA elements (as listed in the Repbase database v16.02) using RepeatMasker v4.0.6 (RepeatMasker, RRID:SCR_012954 [34]) [35] with the parameters “-a -nolow -no_is -norna -parallel 1” and using RepeatProteinMask (implemented in RepeatMasker). To identify repetitive elements *de novo*, we used RepeatModeler v1.0.11 (RepeatModeler, RRID:SCR_015027) [36], with the parameters “-database genome -engine ncbi -pa 15". Tandem repeats in the genome were detected using Tandem Repeat Finder (TRF) v4.07b [37], with parameters “2 7 7 80 10 50 2000 -d -h”. We merged the results of the 2 methods. In total, the new genome assembly comprised 50.81% repetitive sequences (Supplementary Table S10). Closer investigation indicated that the largest categories of repeat elements in the *R. roxellana* genome were the short and long interspersed nuclear elements (SINEs and LINEs, respectively). In addition, several repeat elements absent from the Repbase database were detected in the *de novo* approach (Supplementary Table S10). The total length of these repeat elements was 186,195,432 bp, accounting for 6.13% of the genome, suggesting that these repeat elements may be specific for *R. roxellana*. Compared with the repeat sequences in the previous assembly, our genome included relatively more LINE transposable elements (28.23% vs 6.21%) and tandem repeats (6.20% vs 2.82%). The detailed categories of repeat elements are summarized in Supplementary Table S11.

### Duplicate sequence identification

We also performed duplicate sequence identification analysis, which was fulfilled based on the read depth of Illumina short reads. In brief, we first mapped the Illumina short reads to the assembled genome using BWA with default parameters. Then, the sorted mapping bam file was used as input for CNVnator v0.3.3 [38], a tool targeting alterations in the read depth, with the parameters of “-unique -his 100 -stat 100 -call 100”. The obtained duplicate sequences were filtered, retaining only those where q0 was <0.5 and e-val1 was <0.05. After filtering, 676 duplicate sequences remained, with a total length of 9,198,900 bp (Supplementary Table S12). Further analysis showed 101 duplications located at the end of scaffolds (5% of the total length in both ends). And there were 136 genes present in the duplicated regions—these genes mainly involved in basic biological processes such as ribonucleoside binding, phosphatase activity, and protein dephosphorylation.

### Non-coding RNA prediction

Non-coding RNAs included ribosomal RNAs (rRNAs), transfer RNAs (tRNAs), microRNAs (miRNAs), and small nuclear RNAs (snRNAs). Non-coding RNAs primarily regulate biological processes. Using BLASTN (BLASTN, RRID:SCR_001598) with an E-value of 1E−10, we identified 4 rRNAs in the *R. roxellana* genome homologous to human rRNAs: 28S, 18S, 5.8S, and 5S (GenBank accession numbers NR_0 03287.2, NR_0 03286.2, NR_0 03285.2, and NR_02 3363.1, respectively). We also searched for miRNAs and snRNAs in the new genome using INFERNAL v1.1rc4 (Infernal, RRID:SCR_011809) [39] against the Rfam database release 13.0 [40]. The tRNAs were predicted by tRNAscan-SE 1.3.1 (tRNAscan-SE, RRID:SCR_010835) [41]. We identified 608 rRNAs, 17,813 miRNAs, 3,656 snRNAs, and 460 tRNAs in the *R. roxellana* genome (Supplementary Table S13).

### Gene prediction and functional annotation

We predicted genes using a combination of approaches: *de novo*, homology prediction, and transcriptome. For *ab initio* predictions of protein-coding genes, we used Augustus v3.2.2 (Augustus, RRID:SCR_008417) [42], with parameters “–uniqueGeneId = true –noInFrameStop = true –gff3 = on –genemodel = complete –strand = both”; GlimmeHMM v3.0.1 [43], with parameters “-g -f”; GENSCAN (GENSCAN, RRID:SCR_012902) [44], GENEID [45], and SNAP v2013–11-29 [46].

Next, we predicted genes using a homology-based approach. Protein sequences from 5 homologous species (*Homo sapiens, Gorilla gorilla, Macaca mulatta, Rhinopithecus bieti*,and*Rhinopithecus roxellana hubeiensis*) were downloaded from Ensembl Release 75 [47]. We compared these sequences to the repeat-masked *R. roxellana* genome using TBLASTN (TBLASTN, RRID:SCR_011822, -p tblastn -e 1e-05 -F T -m 8 -d) against the repeat-masked genome sequences [48], with parameters “-p tblastn -e 1e-05 -F T -m 8 -d”. The identified homologous genome sequences were annotated using GeneWise (Version 2.4.1, GeneWise, RRID:SCR_015054) [49], with the parameters “-tfor -genesf -gff”.

Finally, we estimated genes based on transcriptome data. High-quality RNA samples from the heart and skin tissue of the *R. roxellana qinlingensis* specimen (the same individual used for DNA sequencing and reference assembly) were sequenced on an Illumina Novaseq 6000 platform. RNA-sequencing reads were assembled using trinityrnaseq-2.1.1 [50], with the parameters “–seqType fq –CPU 20 –max_memory 200G –normalize_reads –full_cleanup –min_glue 2 –min_kmer_cov 2 –KMER_SIZE 25”. To identify validate transcripts, the assembled transcript sequences were aligned to the *R. roxellana* genome using Assemble Spliced Alignment (PASA) [51], with default parameters. We estimated transcript expression levels using Tophat 2.0.13 (TopHat, RRID:SCR_013035) [52] (with the parameters “-p 6 –max-intron-length 500 000 -m 2 –library-type fr-unstranded”) and Cufflinks (Cufflinks, RRID:SCR_014597) [53].

**Figure 3:**
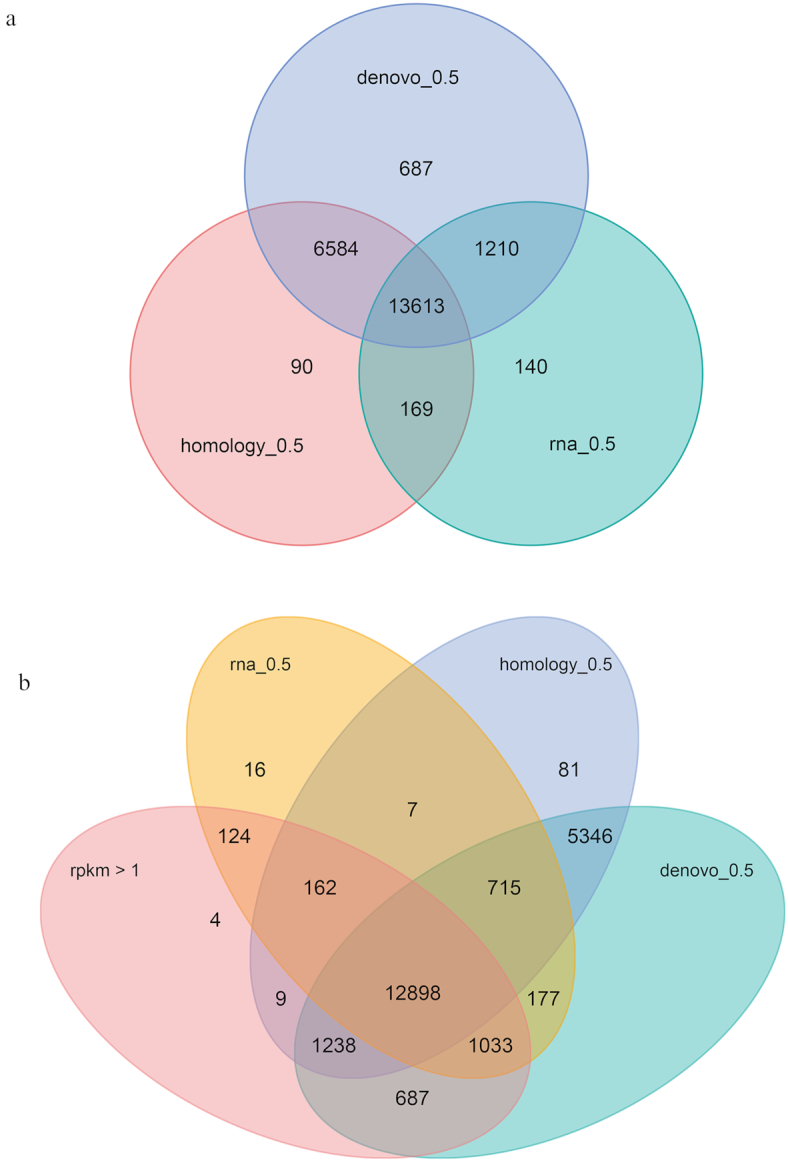
Gene predictions. (a) Number of genes estimated by various prediction approaches: *de novo* (blue), homology (pink), and RNA-sequencing data (green). The labels rna_0.5, denove_0.5, and homology_0.5 indicate the genes predicted by each method with an overlap >50%. (b) Number of genes predicted based on *de novo*, homology, and RNA-sequencing approaches, in addition to expression level (in reads per kilobase of transcript, per million mapped reads [rpkm]). The labels rna_0.5, denove_0.5, and homology_0.5 indicate the genes predicted by each method with an overlap >50%, while rpkm > 1 indicates those genes with a relative expression level >1.

The genes predicted by each of the 3 approaches were merged using EVidenceModeler (EVidenceModeler, RRID:SCR_014659) [54] with the parameters “–segmentSize 200 000 –overlapSize 20 000”. We weighted transcript predictions most highly, followed by homology-based predictions and *ab initio* predictions. Untranslated regions and alternative splicing of the predicted gene were explored using PASA, in conjunction with the transcriptome data [51]. In total, 22,497 genes were predicted in the *R. roxellana* genome (Table 3), each containing a mean of 7.71 exons. The detailed results of the gene prediction process are given in Table 3 and Fig. 3.

**Table 3:** Summary and characteristics of the predicted protein-coding genes

Gene set		Number	Mean transcript length (bp)	Mean coding sequence length (bp)	Mean exon length (bp)	Mean intron length (bp)	Mean exons per gene
*De novo*	Augustus	32,928	23,441	1,052	196	5,112	5.38
	GlimmerHMM	618,957	4,204	404	166	2,654	2.43
	SNAP	97,298	49,851	755	144	11,597	5.23
	Geneid	36,863	35,242	1,035	188	7,615	5.49
	Genscan	50,419	40,635	1,137	167	6,800	6.81
Homology	Ggo	25,281	19,893	1,055	184	3,971	5.74
	Hsa	38,444	14,763	826	182	3,942	4.54
	Mmu	21,959	29,709	1,470	187	4,123	7.85
	Rbi	25,320	25,685	1,387	196	3,991	7.09
	Rro	24,121	28,439	1,420	185	4,043	7.68
RNASeq	PASA	66,620	28,449	1,219	164	4,247	7.41
	Cufflinks	73,199	31,497	2,737	409	5,052	6.69
EVM		30,102	22,298	1,098	182	4,199	6.05
Pasa-update*		29,403	27,638	1,180	181	4,782	6.53
Final set*		22,497	34,153	1,369	178	4,885	7.71

Note: Pasa-update* includes only the untranslated regions; other regions were not included. Final set* represents the results after the Pasa filtering process, where the longest isoform was chosen in the case of multiple splicing isoforms; redundant single exons were also discarded. The “Number” column gives the number of protein-coding genes predicted by each method.

We also compared the gene structure, including mRNA length, exon length, intron length, and exon number, among *R. roxellana* and other representative primates (e.g., *H. sapiens, G. gorilla, M. mulatta, R. bieti*, and*R. roxellana hubeiensis*). We found that genome assembly patterns were similar among *R. roxellana* and the other primates (Supplementary Fig. S2).

To better understand the biological functions of the predicted genes, we used BLASTP (BLASTP, RRID:SCR_001010, with an E-value of 1E−5) to identify the best match for each predicted gene across several databases, including the NCBI nonredundant protein database (NR v20180129), SwissProt (v20150821) [55], KEGG (v20160503) [56], InterPro v29.0 (InterPro, RRID:SCR_006695) [57], Pfam v31.0 (Pfam, RRID:SCR_004726) [58], and GO (Gene Ontology) [59]. In this way, 22,053 predicted genes (98.42%) were functionally annotated (Supplementary Table S14). Nearly half (10,670 of 22,497) of these genes were annotated to the predicted proteins in the NR database derived from the previous genome annotation for *R. roxellana*.

In addition, we estimated the genome assembly completeness using transcriptome data. The transcripts were derived from the *de novo* assembly with trinityrnaseq-2.1.1 mentioned above. Those transcripts were clustered into unigenes with the help of using TGICL (TIGR gene indices clustering program, v2.1) [60] with 95% identity similarity cut-off. The generated unigenes were aligned to our assembly version and previous version using BLAT version 36 (BLAT, RRID:SCR_011919). Results showed that the completeness degree (percentage of unigenes aligned to a single scaffold in the genome) was higher in our assembly (95.35%) than in the previous assembly (89.28%) for unigenes >1,000 bp (Supplementary Table S15), demonstrating the contiguity of our new assembly.

### Phylogenetic analysis and gene family estimation

**Figure 4:**
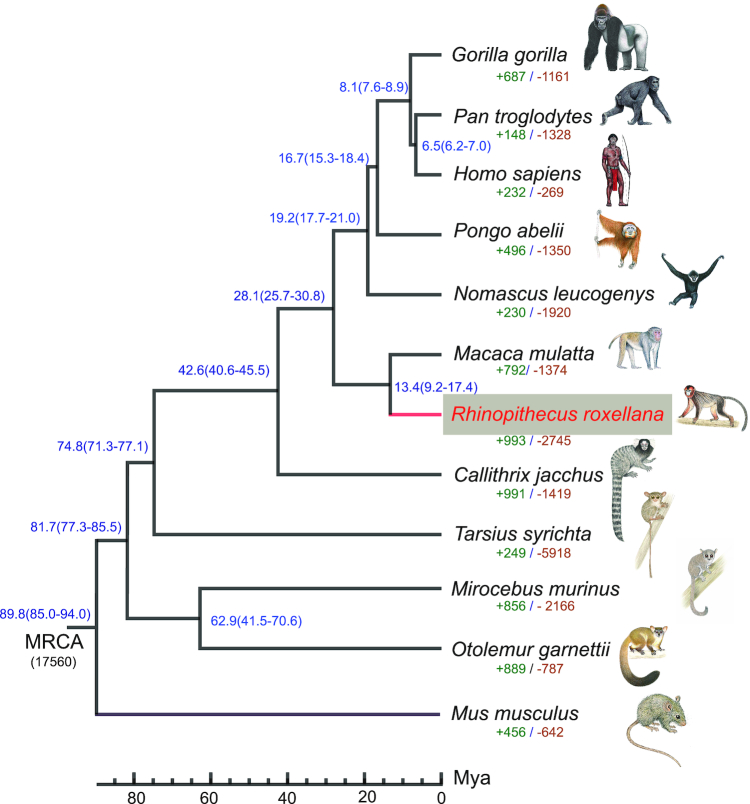
*R. roxellana* phylogenetic relationships and gene families. Phylogenetic relationships were inferred from 5,418 single-copy gene families in *R. roxellana* and other mammals. All nodes had support values of 100%. Estimated divergence times are given near each node. Numbers under each species indicate the number of gene families that have been expanded (green) and contracted (brown) since the split of species from the most recent common ancestor (MRCA). The numbers near each node (blue) correspond to the estimated divergence time of these species. Monkey images are copyright 2013 Stephen D. Nash of the International Union for Conservation of Nature Species Survival Commission Primate Specialist Group and are used with permission. MYA: million years ago.

The coding regions and protein sequences of 11 representative mammals were downloaded from Ensembl (Ensembl Release 75). For genes with multiple transcript isoforms, the longest was chosen. Treefam [61] was used to estimate gene families. Using an all-to-all blast, we identified 17,560 gene families. We reconstructed the phylogenetic relationships among *R. roxellana* and other mammals based on 4-fold degenerate sites extracted from the 5,418 single-copy gene families. Phyml v3.2 (PhyML, RRID:SCR_014629) [62] was used to construct a maximum-likelihood tree using the general time reversible + γ model, as inferred by JMODELTEST v2.1.10 (jModelTest, RRID:SCR_015244) [63]. We estimated divergence times with MCMCTREE in PAML v4.8 (PAML, RRID:SCR_014932) [64], using the Bayesian method and the fossil calibration times from TimeTree [65, 66]. The following fossil calibrations were used: *H. sapiens* vs *Callithrix jacchus* (40.6–45.7 MYA [million years ago]), *H. sapiens* vs *P. troglodytes* (∼6.2–7 MYA), *H. sapiens* vs *Mus musculus* (85–94 MYA), and *H. sapiens* vs *Tarsius syrichta* (∼71–77 MYA). The reconstructed phylogeny recovered a close relationship between *R. rollexana* and *M. mulatta*. We estimated that *R. rollexana* and *M. mulatta* diverged ∼13.4 MYA (Fig. 4).

To investigate the evolutionary history of *R. roxellana*, we estimated the expansion and contraction of gene families in this species with CAFE 3.0 (CAFÉ, RRID:SCR_005983) [67]. A random birth and death model was used to study gene family variations along each lineage in the phylogenetic tree. This analysis identified 993 expanded gene families and 2,745 contracted gene families in the *R. roxellana* genome (Fig. 4). To determine the significance of each gene family, *P*-values in each lineage were estimated by comparing conditional likelihoods derived from a probabilistic graphical model. All gene families with *P*-values < 0.05 were further analyzed. To explore the significantly expanded gene families, we performed a GO-term enrichment analysis with EnrichPipeline32 [68, 69], using the 1,370 genes belonging to the 314 significantly expanded gene families as input, and using all predicted genes as background. We considered a GO term significant if after adjustment the *P-*value was <0.05. We found that the significantly expanded gene families were mainly associated with the hemoglobin complex, energy metabolism, and oxygen transport (Supplementary Table S16).

## Conclusion

In this study, we generated a high-quality genome assembly for the golden snub-nosed monkey (*R. roxellana*) using a combination of 5 advanced genomics technologies. Our results will inform studies of the origin and evolutionary history of the snub-nosed monkey. In addition, this genome may provide a framework within which to survey the mechanisms underlying the formation of the distinct morphological and sociological characters of *R. roxellana*. This genome may also stimulate new insights into the improvement of strategies to conserve and manage this endangered species. Finally, this genome, which has superior continuity and accuracy, may serve as a new standard reference genome for colobine primates.

## Availability of supporting data and materials

The raw data discussed in this publication have been deposited in NCBI's SRA under accession number PRJNA524949. All supporting data and materials and a JBrowse genome browser are available in the *GigaScience* GigaDB database [70].

## Additional files

**Supplementary Figure S1**. Genome size estimation using the k-mer method.

**Supplementary Figure S2**. Comparisons of each element among genomes of homologous species.

**Supplementary Table S1**. The contig assembly based on PacBio subreads.

**Supplementary Table S2**. The scaffold assembly based on sspace-longreads results.

**Supplementary Table S3**. The assembly after gap-filling.

**Supplementary Table S4**. The assembly based on BioNano optical map data.

**Supplementary Table S5**. The assembly based on 10X Genomics linked reads.

**Supplementary Table S6**. The read mapping rate and the coverage of the assembled genome determined with BWA.

**Supplementary Table S7**. The single-nucleotide polymorphisms identified in the genome of *R. roxellana*.

**Supplementary Table S8**. Genome assessment based on BUSCO annotations.

**Supplementary Table S9**. Genome assessment based on CEGMA annotations.

**Supplementary Table S10**. Prediction of repeat elements prediction in the genome assembly.

**Supplementary Table S11**. Prediction of repetitive sequences in the genome assembly.

**Supplementary Table S12**. The duplicated sequences (DS) identified in the genome assembly.

**Supplementary Table S13**. Summary and characteristics of the predicted RNAs.

**Supplementary Table S14**. The functional annotations of the genes predicted in the *R. roxellana* genome.

**Supplementary Table S15**. Assessment of the new genome assembly using unigene

sequences.

**Supplementary Table S16**. The GO annotations of the expanded gene families in the *R. roxellana* genome (adjusted *P-*value < 0.05).

giz098_GIGA-D-19-00030_Original_Submission

giz098_GIGA-D-19-00030_Revision_1

giz098_GIGA-D-19-00030_Revision_2

giz098_Response_to_Reviewer_Comments_Original_Submission

giz098_Response_to_Reviewer_Comments_Revision_1

giz098_Reviewer_1_Report_Original_Submission Jeffrey Rogers -- 4/1/2019 Reviewed

giz098_Reviewer_1_Report_Revision_1 Jeffrey Rogers -- 5/29/2019 Reviewed

giz098_Reviewer_2_Report_Original_Submission Jeff Kidd -- 4/2/2019 Reviewed

giz098_Reviewer_2_Report_Revision_1 Jeff Kidd -- 5/15/2019 Reviewed

giz098_Reviewer_3_Report_Original_Submission Tomas Marques-Bonet -- 4/5/2019 Reviewed

giz098_Reviewer_3_Report_Revision_1 Tomas Marques-Bonet -- 5/30/2019 Reviewed

giz098_Supplemental_File

## Abbreviations

BLAST: Basic Local Alignment Search Tool; BUSCO: Benchmarking Universal Single-Copy Orthologs; CEGMA: core eukaryotic gene-mapping approach; Gb: gigabase pairs; GO: gene ontology; Hi-C: high-throughput chromosome conformation capture; kb: kilobase pairs; KEGG: Kyoto Encyclopedia of Genes and Genomes; LINEs: long interspersed nuclear elements; miRNA: microRNA; Mya: million years ago; NCBI: National Center for Biotechnology Information; NR: NCBI nonredundant protein database; PacBio: Pacific Biosciences; PASA: genome by Assemble Spliced Alignment; rRNA: ribosomal RNA; SINEs: short interspersed nuclear elements; SMRT: single-molecule real-time sequencing; snRNA: small nuclear RNA; SRA: Sequence Read Archive; TFS: transposable element; TRF: Tandem Repeat Finder; tRNA: transfer RNA.

## Competing interests

The authors declare that they have no competing interests.

## Funding

This work was financially supported by Strategic Priority Research Program of the Chinese Academy of Sciences (XDB31020302), the National Natural Science Foundation of China (31 622 053), the Promotional project for Innovation team, the Department of Science and Technology of Shaanxi Prov. China (2018TD-017), and the National Key Programme of Research and Development, the Ministry of Science and Technology of China (2016YFC0503200).

## Authors' contributions

X.G.Q. conceived and designed the project. L.W. and J.W.W. contributed to the work on genomic sequencing and performing data analyses. J.W.W., L.W., and X.G.Q. wrote the manuscript. B.G.L. helped with sample collection. All authors provided input for the paper and approved the final version.

## References

[1] Li BG, Pan RL, Oxnard CE Extinction of snub-nosed monkeys in China during the past 400 years. Int J Primatol. 2002;23(6):1227–44.

[2] Luo MF, Liu ZJ, Pan HJ, et al. Historical geographic dispersal of the golden snub-nosed monkey (*Rhinopithecus roxellana*) and the influence of climatic oscillations. Am J Primatol. 2012;74(2):91–101. 22025257 10.1002/ajp.21006

[3] Fang G, Li M, Liu X-J, et al. Preliminary report on Sichuan golden snub-nosed monkeys (*Rhinopithecus roxellana roxellana*) at Laohegou Nature Reserve, Sichuan, China. Sci Rep. 2018;8(1):16183. 30385788 10.1038/s41598-018-34311-z PMC6212442

[4] Grueter CC, Qi X, Li B, et al. Multilevel societies. Curr Biol. 2017;27(18):R984–6. 28950088 10.1016/j.cub.2017.06.063

[5] Qi XG, Li BG, Garber PA, et al. Social dynamics of the golden snub-nosed monkey (*Rhinopithecus roxellana*): female transfer and one-male unit succession. Am J Primatol. 2009;71(8):670–9. 19434626 10.1002/ajp.20702

[6] Li H, Meng S-J, Men Z-M, et al. Genetic diversity and population history of golden monkeys (*Rhinopithecus roxellana*). Genetics. 2003;164(1):269–75. 12750338 10.1093/genetics/164.1.269 PMC1462553

[7] Qi X-G, Garber PA, Ji W, et al. Satellite telemetry and social modeling offer new insights into the origin of primate multilevel societies. Nat Commun. 2014;5:5296. 25335993 10.1038/ncomms6296 PMC4220467

[8] Zhou X, Wang B, Pan QG, et al. Whole-genome sequencing of the snub-nosed monkey provides insights into folivory and evolutionary history. Nat Genet. 2014;46:1303–10. 25362486 10.1038/ng.3137

[9] Kuang W-M, Ming C, Li H-P, et al. The origin and population history of the endangered golden snub-nosed monkey (*Rhinopithecus roxellana*). Mol Biol Evol. 2019, 36, 3:487–99. 30481341 10.1093/molbev/msy220

[10] Hong YY, Duo HR, Hong JY, et al. Resequencing and comparison of whole mitochondrial genome to gain insight into the evolutionary status of the Shennongjia golden snub-nosed monkey (SNJ *R-roxellana*). Ecol Evol. 2017;7(12):4456–64. 28649355 10.1002/ece3.3011 PMC5478077

[11] Seo JS, Rhie A, Kim J, et al. De novo assembly and phasing of a Korean human genome. Nature. 2016;538(7624):243–7. 27706134 10.1038/nature20098

[12] Chaisson MJ, Wilson RK, Eichler EE Genetic variation and the de novo assembly of human genomes. Nat Rev Genet. 2015;16(11):627–40. 26442640 10.1038/nrg3933 PMC4745987

[13] Jiao YP, Peluso P, Shi JH, et al. Improved maize reference genome with single-molecule technologies. Nature. 2017;546(7659):524–7. 28605751 10.1038/nature22971 PMC7052699

[14] Gordon D, Huddleston J, Chaisson MJP, et al. Long-read sequence assembly of the gorilla genome. Science. 2016;352(6281):aae0344. 27034376 10.1126/science.aae0344 PMC4920363

[15] Kronenberg ZN, Fiddes IT, Gordon D, et al. High-resolution comparative analysis of great ape genomes. Science. 2018;360(6393):eaar6343. 29880660 10.1126/science.aar6343 PMC6178954

[16] Bickhart DM, Rosen BD, Koren S, et al. Single-molecule sequencing and chromatin conformation capture enable de novo reference assembly of the domestic goat genome. Nat Genet. 2017;49:643–50. 28263316 10.1038/ng.3802 PMC5909822

[17] Luo R, Liu B, Xie Y, et al. SOAPdenovo2: an empirically improved memory-efficient short-read de novo assembler. Gigascience. 2012;1(1):18. 23587118 10.1186/2047-217X-1-18 PMC3626529

[18] Martin M Cutadapt removes adapter sequences from high-throughput sequencing reads. EMBnet J. 2011;17:10–12.

[19] Chin CS, Peluso P, Sedlazeck FJ Phased diploid genome assembly with single-molecule real-time sequencing. Nat Methods. 2016;13(12):1050–4. 27749838 10.1038/nmeth.4035 PMC5503144

[20] Chin CS, Alexander DH, Marks P, et al. Nonhybrid, finished microbial genome assemblies from long-read SMRT sequencing data. Nat Methods. 2013;10(6):563–9. 23644548 10.1038/nmeth.2474

[21] Walker BJ, Abeel T, Shea T, et al. Pilon: an integrated tool for comprehensive microbial variant detection and genome assembly improvement. PLoS One. 2014;9(11), doi:10.1371/journal.pone.0112963. PMC4237348 25409509

[22] Boetzer M, Pirovano W SSPACE-LongRead: scaffolding bacterial draft genomes using long read sequence information. BMC Bioinformatics. 2014;15:211. 24950923 10.1186/1471-2105-15-211 PMC4076250

[23] English AC, Richards S, Han Y, et al. Mind the gap: upgrading genomes with Pacific Biosciences RS long-read sequencing technology. PLoS One. 2012;7(11):e47768. 23185243 10.1371/journal.pone.0047768 PMC3504050

[24] Shelton JM, Coleman MC, Herndon N, et al. Tools and pipelines for BioNano data: molecule assembly pipeline and FASTA super scaffolding tool. BMC Genomics. 2015;16:734. 26416786 10.1186/s12864-015-1911-8 PMC4587741

[25] Long Ranger Support https://support.10xgenomics.com/genome-exome/software/pipelines/2.1/what-is-long-ranger. Accessed 1st July 2019

[26] Langmead B, Trapnell C, Pop M, et al. Ultrafast and memory-efficient alignment of short DNA sequences to the human genome. Genome Biol. 2009;10(3):R25. 19261174 10.1186/gb-2009-10-3-r25 PMC2690996

[27] Adey A, Kitzman JO, Burton JN, et al. In vitro, long-range sequence information for de novo genome assembly via transposase contiguity. Genome Res. 2014;24(12):2041–9. 25327137 10.1101/gr.178319.114 PMC4248320

[28] Li H, Durbin R Fast and accurate short read alignment with Burrows-Wheeler transform. Bioinformatics. 2009;25(14):1754–60. 19451168 10.1093/bioinformatics/btp324 PMC2705234

[29] Burton JN, Adey A, Patwardhan RP, et al. Chromosome-scale scaffolding of de novo genome assemblies based on chromatin interactions. Nat Biotechnol. 2013;31(12):1119–25. 24185095 10.1038/nbt.2727 PMC4117202

[30] Kurtz S, Phillippy A, Delcher AL, et al. Versatile and open software for comparing large genomes. Genome Biol. 2004;5(2):R12. 14759262 10.1186/gb-2004-5-2-r12 PMC395750

[31] SAMtools. http://samtools.sourceforge.net/. Accessed 1st July 2019

[32] Simao FA, Waterhouse RM, Ioannidis P, et al. BUSCO: assessing genome assembly and annotation completeness with single-copy orthologs. Bioinformatics. 2015;31(19):3210–2. 26059717 10.1093/bioinformatics/btv351

[33] Parra G, Bradnam K, Korf I CEGMA: a pipeline to accurately annotate core genes in eukaryotic genomes. Bioinformatics. 2007;23(9):1061–7. 17332020 10.1093/bioinformatics/btm071

[34] RepeatMasker. http://www.repeatmasker.org/. Accessed 1st July 2019

[35] Jurka J, Kapitonov VV, Pavlicek A, et al. Repbase Update, a database of eukaryotic repetitive elements. Cytogenet Genome Res. 2005;110(1-4):462–7. 16093699 10.1159/000084979

[36] Price AL, Jones NC, Pevzner PA De novo identification of repeat families in large genomes. Bioinformatics. 2005;21(Suppl 1):i351–8. 15961478 10.1093/bioinformatics/bti1018

[37] Benson G Tandem repeats finder: a program to analyze DNA sequences. Nucleic Acids Res. 1999;27(2):573–80. 9862982 10.1093/nar/27.2.573 PMC148217

[38] Abyzov A, Urban AE, Snyder M, et al. CNVnator: an approach to discover, genotype, and characterize typical and atypical CNVs from family and population genome sequencing. Genome Res. 2011;21(6):974–84. 21324876 10.1101/gr.114876.110 PMC3106330

[39] Nawrocki EP, Kolbe DL, Eddy SR Infernal 1.0: inference of RNA alignments. Bioinformatics. 2009;25(10):1335–7. 19307242 10.1093/bioinformatics/btp157 PMC2732312

[40] Griffiths-Jones S, Moxon S, Marshall M, et al. Rfam: annotating non-coding RNAs in complete genomes. Nucleic Acids Res. 2005;33:D121–4. 15608160 10.1093/nar/gki081 PMC540035

[41] Lowe TM, Eddy SR tRNAscan-SE: a program for improved detection of transfer RNA genes in genomic sequence. Nucleic Acids Res. 1997;25(5):955–64. 9023104 10.1093/nar/25.5.955 PMC146525

[42] Stanke M, Keller O, Gunduz I, et al. AUGUSTUS: ab initio prediction of alternative transcripts. Nucleic Acids Res. 2006;34:W435–9. 16845043 10.1093/nar/gkl200 PMC1538822

[43] Majoros WH, Pertea M, Salzberg SL TigrScan and GlimmerHMM: two open source ab initio eukaryotic gene-finders. Bioinformatics. 2004;20(16):2878–9. 15145805 10.1093/bioinformatics/bth315

[44] Burge C, Karlin S Prediction of complete gene structures in human genomic DNA. J Mol Biol. 1997;268(1):78–94. 9149143 10.1006/jmbi.1997.0951

[45] Guigo R Assembling genes from predicted exons in linear time with dynamic programming. J Comput Biol. 1998;5(4):681–702. 10072084 10.1089/cmb.1998.5.681

[46] Korf I Gene finding in novel genomes. BMC Bioinformatics. 2004;5:59. 15144565 10.1186/1471-2105-5-59 PMC421630

[47] Ensembl. http://www.ensembl.org/info/data/ftp/index.html. Accessed 1st July 2019

[48] Kent WJ BLAT - The BLAST-like alignment tool. Genome Res. 2002;12(4):656–64. 11932250 10.1101/gr.229202 PMC187518

[49] Birney E, Clamp M, Durbin R GeneWise and Genomewise. Genome Res. 2004;14(5):988–95. 15123596 10.1101/gr.1865504 PMC479130

[50] Grabherr MG, Haas BJ, Yassour M, et al. Full-length transcriptome assembly from RNA-Seq data without a reference genome. Nat Biotechnol. 2011;29(7):644–U130. 21572440 10.1038/nbt.1883 PMC3571712

[51] Haas BJ, Delcher AL, Mount SM, et al. Improving the *Arabidopsis*genome annotation using maximal transcript alignment assemblies. Nucleic Acids Res. 2003;31(19):5654–66. 14500829 10.1093/nar/gkg770 PMC206470

[52] Kim D, Pertea G, Trapnell C, et al. TopHat2: accurate alignment of transcriptomes in the presence of insertions, deletions and gene fusions. Genome Biol. 2013;14(4):R36. 23618408 10.1186/gb-2013-14-4-r36 PMC4053844

[53] Trapnell C, Roberts A, Goff L, et al. Differential gene and transcript expression analysis of RNA-seq experiments with TopHat and Cufflinks. Nat Protoc. 2012;7(3):562–78. 22383036 10.1038/nprot.2012.016 PMC3334321

[54] Haas BJ, Salzberg SL, Zhu W, et al. Automated eukaryotic gene structure annotation using EVidenceModeler and the program to assemble spliced alignments. Genome Biol. 2008;9(1):R7. 18190707 10.1186/gb-2008-9-1-r7 PMC2395244

[55] Bairoch A, Apweiler R, Wu CH, et al. The Universal Protein Resource (UniProt). Nucleic Acids Res. 2005;33(Database issue):D154–9. 15608167 10.1093/nar/gki070 PMC540024

[56] Kanehisa M, Goto S KEGG: Kyoto Encyclopedia of Genes and Genomes. Nucleic Acids Res. 2000;28(1):27–30. 10592173 10.1093/nar/28.1.27 PMC102409

[57] Mitchell AL, Attwood TK, Babbitt PC et al. InterPro in 2019: improving coverage, classification and access to protein sequence annotations. Nucleic Acids Res. 2019;47(D1):D351–60. 30398656 10.1093/nar/gky1100 PMC6323941

[58] Finn RD, Coggill P, Eberhardt RY, et al. The Pfam protein families database: towards a more sustainable future. Nucleic Acids Res. 2016;44(D1):D279–85. 26673716 10.1093/nar/gkv1344 PMC4702930

[59] Ashburner M, Ball CA, Blake JA, et al. Gene Ontology: tool for the unification of biology. Nat Genet. 2000;25(1):25–29. 10802651 10.1038/75556 PMC3037419

[60] Pertea G, Huang X, Liang F, et al. TIGR Gene Indices clustering tools (TGICL): a software system for fast clustering of large EST datasets. Bioinformatics. 2003;19(5):651–2. 12651724 10.1093/bioinformatics/btg034

[61] Li H, Coghlan A, Ruan J, et al. TreeFam: a curated database of phylogenetic trees of animal gene families. Nucleic Acids Res. 2006;34:D572–80. 16381935 10.1093/nar/gkj118 PMC1347480

[62] Guindon S, Delsuc F, Dufayard J-F, et al. Estimating maximum likelihood phylogenies with PhyML. In: Bioinformatics for DNA Sequence Analysis. Springer; 2009:113–37. 10.1007/978-1-59745-251-9_6 19378142

[63] Posada D jModelTest: phylogenetic model averaging. Mol Biol Evol. 2008;25(7):1253–6. 18397919 10.1093/molbev/msn083

[64] Yang Z PAML 4: Phylogenetic Analysis by Maximum Likelihood. Mol Biol Evol. 2007;24(8):1586–91. 17483113 10.1093/molbev/msm088

[65] TimeTree. http://www.timetree.org/. Accessed 1st July 2019

[66] Kumar S, Stecher G, Suleski M, et al. TimeTree: a resource for timelines, timetrees, and divergence times. Mol Biol Evol. 2017;34(7):1812–9. 28387841 10.1093/molbev/msx116

[67] De Bie T, Cristianini N, Demuth JP, et al. CAFE: a computational tool for the study of gene family evolution. Bioinformatics. 2006;22(10):1269–71. 16543274 10.1093/bioinformatics/btl097

[68] Huang da W, Sherman BT, Lempicki RA Bioinformatics enrichment tools: paths toward the comprehensive functional analysis of large gene lists. Nucleic Acids Res. 2009;37(1):1–13. 19033363 10.1093/nar/gkn923 PMC2615629

[69] Beissbarth T, Speed TP GOstat: find statistically overrepresented Gene Ontologies within a group of genes. Bioinformatics. 2004;20(9):1464–5. 14962934 10.1093/bioinformatics/bth088

[70] Wang L, Wu J, Liu X, et al. Supporting data for “A high-quality genome assembly of the endangered golden snub-nosed monkey *Rhinopithecus roxellana*.”. GigaScience Database. 2019 10.5524/100619. PMC6705546 31437279

