# Supplementary material for: A high-quality genome assembly for the endangered golden snub-nosed monkey (Rhinopithecus roxellana)
Source: Gigascience. 2019 Aug 22;8(8):giz098. doi: 10.1093/gigascience/giz098 (PMC6705546; doi:10.1093/gigascience/giz098)
Supplement: giz098_GIGA-D-19-00030_Original_Submission [file giz098_giga-d-19-00030_original_submission.pdf]

GigaScience

A high-quality genome assembly of the endangered golden snub-nosed monkey (Rhinopithecus roxellana)

--Manuscript Draft--

Manuscript Number:	GIGA-D-19-00030
Full Title:	A high-quality genome assembly of the endangered golden snub-nosed monkey (Rhinopithecus roxellana)
Article Type:	Data Note
Funding Information:	National Natural Science Foundation of China (31622053) — Dr. Xiao-Guang Qi
Abstract:	Background: The golden snub-nosed monkey (Rhinopithecus roxellana), is an endangered colobine monkey species endemic to China. This species has several distinctive traits, and it is an ideal model for analysing evolutionary development of the social structure due to its unique social organization. Although there has been reported a genome assembly of the subspecies R. roxellana hubeiensis, the assembly is incomplete and fragmented due to employing short reads sequencing technology. This drawback may lose information, such as structural variation and repeat sequences which are important for understanding this endangered species. Therefore, to have a better understanding of evolutionary history and genetic-specific signatures, a high-quality reference genome of the taxon is need.<br>Findings: To obtain a high-quality chromosome assembly of R. roxellana qinlingensis, we combined a total of five techniques including Pacific Bioscience's single-molecule real-time sequencing, Illumina's paired-end sequencing, BioNano optical maps, 10X Genomics link-reads and high-throughput chromosome conformation capture. The results indicate the assembled genome is about 3.04 Gb with a contig N50 of 5.72 Mbp and a scaffold N50 of 144.56 Mbp, which have made a 10-fold improvement compared to past published. It is shown that a total of 22497 protein coding genes were predicted, of which 22053 were functionally annotated. Moreover, gene family analysis shows that 993 and 2745 gene families are expanded and contracted in the R. roxellana genome, respectively.<br>Conclusion: We present the updated high-quality genome assembly of R. roxellana with superior continuity and accuracy. The assembled genome can be used as reference for future genetic studies of the species. Also, the updated genome assembly may contribute to our comprehensive understanding of the species, which is particularly helpful in the conservation of this endangered species. Furthermore, such genome with superior continuity and accuracy can provide a new standard reference for Colobine primates.
Corresponding Author:	Xiao-Guang Qi<br><br>CHINA
Corresponding Author Secondary Information:	
Corresponding Author's Institution:	
Corresponding Author's Secondary Institution:	
First Author:	Xiao-Guang Qi
First Author Secondary Information:	
Order of Authors:	Xiao-Guang Qi
	Lu Wang
	Jinwei Wu
	Xiaomei Liu

Powered by Editorial Manager® and ProduXion Manager® from Aries Systems Corporation

	Dandan Di
	Yuhong Liang
	Yifei Feng
	Baoguo Li
**Order of Authors Secondary Information:**	
**Additional Information:**	
**Question**	**Response**
Are you submitting this manuscript to a special series or article collection?	No
**Experimental design and statistics**<br><br>Full details of the experimental design and statistical methods used should be given in the Methods section, as detailed in our Minimum Standards Reporting Checklist. Information essential to interpreting the data presented should be made available in the figure legends.<br><br>Have you included all the information requested in your manuscript?	Yes
**Resources**<br><br>A description of all resources used, including antibodies, cell lines, animals and software tools, with enough information to allow them to be uniquely identified, should be included in the Methods section. Authors are strongly encouraged to cite Research Resource Identifiers (RRIDs) for antibodies, model organisms and tools, where possible.<br><br>Have you included the information requested as detailed in our Minimum Standards Reporting Checklist?	Yes
**Availability of data and materials**<br><br>All datasets and code on which the conclusions of the paper rely must be either included in your submission or	Yes

Powered by Editorial Manager® and ProduXion Manager® from Aries Systems Corporation

deposited in publicly available repositories (where available and ethically appropriate), referencing such data using a unique identifier in the references and in the “Availability of Data and Materials” section of your manuscript.<br><br>Have you have met the above requirement as detailed in our Minimum Standards Reporting Checklist?	

Manuscript
Click here to view linked References
Click here to access/download;Manuscript;renamed_dade0.docx

A high-quality genome assembly of the endangered golden snub-nosed monkey (*Rhinopithecus roxellana*)

Lu Wang[1,†], Jinwei Wu[1,†], Xiaomei Liu[1], Dandan Di[1], Yuhong Liang[1], Yifei Feng[1], Baoguo Li[1,2], Xiao-Guang Qi[1,*]

[1] Shaanxi Key Laboratory for Animal Conservation, College of Life Sciences, Northwest University, Xi'an, 710069, China.

[2] Center for Excellence in Animal Evolution and Genetics, Chinese Academy of Sciences, Kunming, 650223, China.

[*]**Correspondence address.** Xiao-Guang Qi, E-mail: qixg@nwu.edu.cn

[†]These authors contributed equally to this work.

ABSTRACT

Background: The golden snub-nosed monkey (*Rhinopithecus roxellana*), is an endangered colobine monkey species endemic to China. This species has several distinctive traits, and it is an ideal model for analysing evolutionary development of the social structure due to its unique social organization. Although there has been reported a genome assembly of the subspecies *R. roxellana hubeiensis*, the assembly is incomplete and fragmented due to employing short reads sequencing technology. This drawback may lose information, such as structural variation and repeat sequences which are important for understanding this endangered species. Therefore, to have a better understanding of evolutionary history and genetic-specific signatures, a high-quality reference genome of the taxon is need.

Findings: To obtain a high-quality chromosome assembly of *R. roxellana qinlingensis*, we combined a total of five techniques including Pacific Bioscience's single-molecule real-time sequencing, Illumina's paired-end sequencing, BioNano optical maps, 10X Genomics link-reads and high-throughput chromosome conformation capture. The results indicate the assembled genome is about 3.04 Gb with a contig N50 of 5.72 Mbp and a scaffold N50 of 144.56 Mbp, which have made a 10-fold improvement compared to past published. It is shown that a total of 22497 protein coding genes were predicted, of which 22053 were functionally

annotated. Moreover, gene family analysis shows that 993 and 2745 gene families are expanded and contracted in the *R. roxellana* genome, respectively.

Conclusion: We present the updated high-quality genome assembly of *R. roxellana* with superior continuity and accuracy. The assembled genome can be used as reference for future genetic studies of the species. Also, the updated genome assembly may contribute to our comprehensive understanding of the species, which is particularly helpful in the conservation of this endangered species. Furthermore, such genome with superior continuity and accuracy can provide a new standard reference for Colobine primates.

Keywords: high-quality; *Rhinopithecus roxellana*; genome assembly; annotation; BioNano optical maps

Data Description

Background information

Snub-nosed monkeys (*Rhinopithecus*) consist of five endangered species narrowly restricted to China and Vietnam [1]. Among those, the golden snub-nosed monkey (*Rhinopithecus roxellana*) is also referred to as the Sichuan snub-nosed monkey, with the northernmost distribution of all Asian colobine species, found only in three isolated regions (Sichuan and Gansu, Shaanxi and Hubei provinces) in central and northwest China [2, 3]. This species is characterized by several distinctive traits, such as golden fur, blue facial colour, odd-shaped nose, more folivorous, most striking unique social system with multilevel societies, a rare and complex system that is found only in a few mammal species, including human beings [4]. Therefore, *R. roxellana* is an ideal model for the studies analysing evolutionary development of the social structure in primates and understanding the behaviour patterns of human society in social-anthropology.

As a research hotspot, studies on *R. roxellana* have investigated various aspects [4-6]. Recently, genomic analysis offered a powerful tool and has successfully been employed to underlie the molecular evolution of several groups [7-9]. According to the morphological variation and distribution difference, *R. roxellana* can differentiate into three subspecies: *Rhinopithecus roxellana roxellana* from Minshan mountains of Sichuan and Gansu province,

R. r. Qinlingensis from Qinling mountains of Shaanxi province, *R. r. hubeiensis* from shennongjia mountains [3]. Up to now, the best genome assembly of *R. roxellana* was published in 2014 [10], which was derived from short reads sequencing on Illumina HiSeq 2000 platform. Based on this achievement, studies on its folivorous dietary adaptations and the evolutionary history of *R. roxellana* have been conducted [10-12]. Despite such progress, the information including structural variation and repeat sequences was largely absent or unreliable due to the incomplete and fragmented genome assembly [13, 14].

Owing to the advances in sequencing technology, it is possible to obtain high-quality genome assembly that can provide new insights into the understanding of the organisms. Indeed, transposable elements and lineage-specific genes have never been reported to be identified by using the new improved maize reference genome [15]. By combining new sequencing approaches, Seo et al. [13] discovered clinically relevant structural variants and genes never reported before in updated human genome. New sequencing technologies have also been widely used in Gorillas [16] and Sumatran orangutan [17] that have the closet genetic relationship with humans, and domestic goat [18]. More importantly, a lot of new findings have been reported based on these updated genome assembly. However, a high-quality genome assembly of *R. roxellana* has not been reported yet, lagging the progress of understanding this endangered species.

Here, we report a greatly improved assembly and annotation of the reference genome for *R. roxellana* from through combined five technologies: Pacific Bioscience's single-molecule real-time sequencing (SMRT), Illumina's HiSeq paired-end sequencing (HiSeq), BioNano optical maps (BioNano), 10X Genomics link-reads (10X Genomics) and high-throughput chromosome conformation capture (Hi-C). Also, this is the first Colobine genome sequenced and assembled with both long reads and short reads. The updated genome assembly may allow us to comprehensively understand *R. roxellana*, offering new opportunities in analysing evolutionary history and genetic-specific signatures for this species, which may provide new insights in the conservation of this endangered primate. In addition, this genome with superior continuity and accuracy will provide a new standard reference for Colobine primates.

Sample collection and sequencing

An adult dead male *R. roxellana qinlingensis* used for sequencing was from Louguantai Breeding Centre, Xi'an, Shaanxi province, China, originally from Qinling Mountains. Total genomic DNA was extracted from heart tissue. To acquire a high-quality genome assembly, we applied a combined five sequencing methods. Initially, PacBio's SMRT sequencing was conducted on the SEQUEL platform according to manufactures, after removing adaptors in polymerase reads, resulting a total of 304.84 Gb clean long reads (95.86X coverage). Different

from PacBio sequencing, paired-end sequencing was performed using an Illumina NovaSeq 6000 platform with an insert size of 350 bp. Short reads derived from this step were filtered by SOAPfilter v. 2.2 [19] (a package from SOAPdenovo2) with the following criteria: filtering those reads with adapters, contaminations, N bases more than 10% and low quality, which generated 423.32 Gb sequencing clean reads (133.12X coverage). In addition, a high-quality optical genome map was constructed with Irys platform (BioNano Genomics), from which we acquired 463.75 Gb clean reads (145.83X coverage). Besides, 10X genomic link-reads sequencing was carried out on Illumina Hiseq Xten platform, and 348.41 Gb clean reads (109.56X coverage) were generated in total. Finally, a Hi-C library was prepared and sequenced with an Illumina NovaSeq 6000 platform for chromosome-scale scaffolding of genome assembly. Adapter sequences and low quality reads were discarded by using Cutadapter v1.0 [20], yielding a total of 310.92 Gb clean data (97.77X coverage). Statistics of the sequencing data was detailed in **Table 1**.

De novo **assembly of the** ***R. roxellana*** **genome**

Estimation of genome size is helpful to our understanding of *R. roxellana*. Generally, we estimated the genome size of *R. roxellana* with the formula of $G = (K_{total} – K_{error})/D$, in which G represents genome size, while K_{total}, K_{error} and D indicates the total number of k-mers, number of k-mers which caused by sequencing errors and k-mer depth respectively. Finally, 109,210,004,556 k-mers were generated, and the peak k-mer depth was 34. Thus, the genome

size of *R. roxellana* was estimated to be about 3.18 Gb. The distribution of k-mer frequency was shown in **Supplementary Fig. S1**.

The *de novo* assembly of newly sequenced *R. roxellana* genome was performed in four progressive steps. Firstly, the assembly was conducted with the FALCON assembler (default parameters) [21] with the long reads obtained from the PacBio platform, which mainly includes three steps: 1) detection of overlap and reads correction; 2) detection of overlap between corrected reads; and 3) construction of string graph. Following FALCON step, the string graph assembly was further polished by Quier with long reads [22] and then corrected by Pilon with Illumina short reads [23]. Based on this initial genome assembly, sspace-longreads [19] with default settings was implemented for getting a longer scaffold genome by using PacBio long reads. Despite attempts have been made, scaffolding gaps were still found, those gaps were further closed with the help of PBjelly software under default settings, which generated a phased genome assembly with scaffold N50 of 8.20 Mbp (**Supplementary Table S1**).

Secondly, a hybrid assembly with scaffold N50 of 9.22 Mbp was constructed on the basis of Bionano optical map data using Bionano Solve3.1 (www.bionanogenomics.com) with default parameters (**Supplementary Table S2**). Thirdly, 10X genomic linked reads were employed to connect scaffolds from the second step by fragScaff software [24], which has updated the scaffold N50 of genome assembly to 24.09 Mbp (**Supplementary Table S3**).

Subsequently , those short-reads derived from Illumina were applied to correcting errors due to Burrows-Wheeler Aligner (BWA) [25] and pilon-1.18 [23].

Finally, to build chromosome-level assembly scaffolds, we mapped the Hi-C reads to the assembled scaffolds with BWA [25]. Then Hi-C data was subsequently applied to cluster, order, and orient scaffolds by Lachesis software [26]. The chromosome-level scaffolds for *R. roxellana* allowed us to estimate the interaction frequency between chromosome loci, the interaction heatmap shown in **Fig. 2**.

These processes together yielded a updated genome assembly of *R. roxellana* with its genome size of 3.04 Gb, contig N50 of 5.72 Mbp and scaffold N50 of 144.56 Mbp (**Table 2**). In comparison, the newly acquired *R. roxellana* reference genome has 100-fold higher contiguity than its previous (contig N50: 5.72 Mb versus 25.5 kb and scaffold N50: 144.56 Mb versus 1.55 Mb) [10]. We suppose that the remarkable improvement in contiguity can be attributed to the longer read length, deeper sequencing depth, properly assembled gaps, and increased sophisticated assembly algorithm.

To assess the genome assembly accuracy, we aligned the Illumina short reads to the assembly by BWA program [25]. With a ratio number of 99.17%, mapped read covered approximately 99.27% of the assembly (**Supplementary Table S4**). In addition, we estimated the assembly completeness by conducting Benchmarking Universal Single-copy Orthologs (BUSCO) analysis with BUSCO V3.0 [27]. Among the 4,104 mammalian BUSCOs, 94% was

detected in the genome assembly (**Supplementary Table S5**). The assembly completeness was also checked by core eukaryotic gene-mapping approach (CEGMA) [28]. The results showed that 93.95% (233 of 248) conserved genes were found in our genome assembly (**Supplementary Table S6**). Together, these analyses indicated a high accuracy and completeness of our genome assembly.

Identification of repeat elements

Repeat sequences occupy a large proportion of the genome sequences. Thus, it is necessary for us to identified those repeat elements. In our study, we combined homolog based and *de novo* based approach to predict and classify repeat elements. As for the homolog approach, we searched transposable elements from the RepBase database [29] with RepeatMasker v4.0.6 (http://www.repeatmasker.org/) and RepeatProteinMask (implemented in RepeatMasker). The *de novo* method was employed with RepeatModeler V1.0.11 [30], RepeatMasker v4.0.6 and Tandem Repeat Finder (TRF) (Version 4.07b) [31]. We merged the findings from both methods. Results showed that 45.43% of the genome was predicted as repeat elements (**Supplementary Table S7)**. A closer investigation indicated that the largest category of repeat elements in the species is the short (SINEs) and long (LINEs) interspersed nuclear elements. The detailed categories of repeat elements are summarized in **Supplementary Table S8**.

Non-coding RNA prediction

Non-coding RNA consists of several RNAs, as such ribosomal RNA (rRNA), transfer RNA (tRNA), microRNAs (miRNA) and small nuclear RNA (snRNA). This RNA group mainly plays a regulation role in biological processes. In our study, we detected rRNA from a Human rRNA database with BLASTN command, and the E-value was set as 1E-10. Similarly, miRNAs and snRNAs were searched against the Rfam database [32] with INFERNAL 1.1rc4 [33]. The tRNAs were predicted by tRNAscan-SE 1.3.1 software [34]. The numbers of rRNA, miRNA, snRNA and tRNA were 608, 17,813, 3,656 and 460, respectively in the genome of the species (**Supplementary Table S9**).

Gene prediction and functional annotation

We combined prediction methods based on *de novo*, homolog prediction and transcriptome data to estimate genes. As for *ab initio* based prediction, a total of five programs, namely Augustus v. 3.2.2 [35], GlimmeHMM v. 3.0.1 [36], GENSCAN [37], GENEID [38] and SNAP V2013-11-29 [39] were employed to predict protein-coding genes. Subsequently, we used the homolog-based prediction approach. Protein sequences from five homolog species (*Homo sapiens, Gorilla gorilla*, *Macaca mulatta*, *Rhinopithecus bieti*, *Rhinopithecus roxellana hubeiensis*) were downloaded from Ensemble Release 75

(http://www.ensembl.org/info/data/ftp/index.html), and used to perform TBLASTN blast against the repeat-masked genome sequences [40]. The related homologous genome sequences were then annotated to the matching proteins by GeneWise 2.4.1 [41]. Finally, we estimated genes based on transcriptome data. During this process, high-quality RNAs from heart and skin tissue were sequenced by an Illumina Novaseq 6000 platform. RNA-seq reads were assembled with trinityrnaseq-2.1.1 [42]. The assembled transcript sequences were aligned to the *R. roxellana* genome by Assemble Spliced Alignment (PASA) [43] with default parameters. In addition, we estimated the expression levels of transcripts by Tophat 2.0.13 [44] and Cufflinks [45].

The genes predicted from those three approaches were merged with EVidenceModeler [46]. Furthermore, untranslated regions and alternative splicing of those predicted gene sets were further checked by PASA with the help of transcriptome data [43]. Finally, a total of 22497 genes were predicted for the assembly genome of *R. roxellana* (**Table 3**), and each of them consisted of 7.71 exons on average. The detailed results generated during the gene prediction process were shown in **Table 3.** And, the gene prediction evidence based on different methods were shown in **Fig. 3**. In addition, we made a comparison between the *R. roxellana qinlingensis* and other mammals, suggesting a comparable pattern of the genome assembly for *R. roxellana qinlingensis* **(Supplementary Fig. S2)**.

To have a better understanding the biological functions of those predicted genes, they were annotated with several databases including NCBI nonredundant protein database (NR), SwissProt [47], Kyoto Encyclopedia of Genes and Genomes (KEGG) [48], InterPro [49], Pfam [50] and GO database [51]. In total, 22053 genes (98.42%) were functionally annotated (**Supplementary Table S10**).

Phylogenetic relationship analysis and gene family estimation

Coding regions and protein sequences of 11 representative mammals were downloaded from Ensemble (Ensemble Release 75). The longest transcript was chosen if genes possess many transcript isoforms. Treefam [52] approach was adopted to estimate gene families. Following all-to-all blast, a total of 17,560 gene families were identified. We reconstructed the phylogenetic relationship between *R. roxellana* and other mammals based on four-fold degenerate sites extracted from the 5,418 single-copy gene families. Phyml (version 3.2) [53] was employed to construct a maximum-likelihood tree under the GTR + gamma model that was inferred from JMODELTEST (version 2.1.10) [54]. Furthermore, we estimated the divergence time with MCMCTREE in PAML [55]. MCMCTREE was performed on the basis of bayesian method and the fossil calibration times from timetree were used as input. The reconstructed phylogeny confirmed the close relationship between *R. rollexana* and *M. mulatta*.

Moreover, we estimated that *R. rollexana* and *M. mulatta* diverged approximately 13.4 million years ago (Mya) (**Fig. 4**).

To have a better understanding the evolutionary history of *R. roxellana*, we estimated the expansion and contraction of gene family in *R. roxellana* by using CAFE 3.0 [56]. A gene family with *p*-value less than 0.05 was considered for further analysis. As a result, 993 and 2,745 gene families were expanded and contracted in *R. roxellana* genome, respectively (**Fig. 4**). Its genome showed substantial expansion of gene families which are mainly related to hemoglobin complex, energy metabolisms and oxygen transport (**Supplementary Table S11**).

Conclusion

In this study, we generated a high-quality genome assembly of the golden snub-nosed monkey (*R. roxellana*) by using five advanced technologies. This will be helpful to investigate the origin and evolutionary history of snub-nosed monkey. In addition, the genome may lay a foundation to survey the mechanisms about the formation of distinct characters and understand the unique multilevel societies in *R. roxellana*. Also, such genome may provide new insights for amending the conservation strategies and management of this endangered species. Furthermore, this genome with superior continuity and accuracy can provide a new standard reference for Colobine primates.

Declarations

Availability of supporting data

The raw data discussed in this publication have been deposited in NCBI's short read archive under the accession number PRJNA524949. Supporting data are available in the GigaDB database.

Competing interests

The authors declare that they have no competing interests.

Funding

This work was financially supported by Strategic Priority Research Program of the Chinese Academy of Sciences (XDB31020302), the National Natural Science Foundation of China (31622053, 31730104), the Promotional project for Innovation team, the Department of Science and Technology of Shaanxi Prov. China (2018TD-017), and the National Key Programme of Research and Development, the Ministry of Science and Technology of China (2016YFC0503200).

Abbreviations

Gb: gigabase; kb: kilobase; Mb: megabase; PE: paired-end; PacBio: Pacfic Biosciences; SMRT: single molecule real-time sequencing; Hi-C: high-throughput chromosome conformation capture; BUSCO: Benchmarking Universal Single-copy Orthologs; GEGMA: core eukaryotic gene-mapping approach; GO: gene ontology; TFS: transposable element; TRF: Tandem Repeat Finder; SINEs: Short interspersed nuclear elements; LINEs: long

interspersed nuclear elements; PASA: genome by Assemble Spliced Alignment; NR: NCBI nonredundant protein database; KEGG: Kyoto Encyclopedia of Genes and Genomes. Mya: million years ago.

Author contributions

X.G.Q. conceived and designed the project, L.W., J.W. contributed to the work on genomic sequencing and performing data analyses. B.G.L. helped with sample collection. L.W., J.W. and X.G.Q. wrote the manuscript. All authors provided input for the paper and approved the final version.

Acknowledgements

We thank Mr. Yiliang Xu, Mr. Qiqi Liang, Mrs. Yue Xie from Novogene for their technical support. Mr. Xuanmin Guang and Mr. Chi Zhang from BGI for their assistance in data analysis. We thank to Mr. Ruliang Pan for his gracious help polishing the language. We are also grateful to Mr. Yinghu Lei from Louguantai Breeding Center, Dr. Zhipang Huang, and Dr. Pei Zhang from Northwest University for their helping with the sampling collection. We specially appreciate Prof. Zhengbing Wang, and Prof. Jiang Chang from Discipline Development Department of Northwest University for their support. This study was fundamentally supported by Discipline Construction Project of Northwest University.

Figures and tables

Figure legends:

Fig. 1. The photo of *R. roxellana* taken in the Qinling mountains.

Fig. 2. Hi-C heatmap between chromosome loci throughout the genome. Hi- C interactome within and among chromosomes of *R. roxellana* (Chr1–Chr22).

Fig. 3. The gene prediction evidence based on different methods. (a). Number of the genes estimated by the prediction approaches based on de novo (blue color), homolog prediction (pink color) and RNA_seq data (green color). The rna_0.5, denove_0.5 and homolog_0.5 indicates those genes predicted with an overlap are larger than 50% in each method; **(**b) Number of the genes shown in combination with the prediction approaches detailed in fig 2a and the expression level standard (rpkm). The rna_0.5, denovo_0.5, homolog_0.5 indicates that those genes predicted with an overlap are larger than 50% in each method, while rpkm>1 indicates those genes with an expression level larger than 1.

Fig. 4. The phylogenetic relationships of *R. roxellana* and other mammals and Gene family analysis in *R. roxellana* genome. Phylogenetic relationship was inferred from 5418 single-copy gene families. All nodes received 100% support values. The estimated divergence times are indicated near the nodes. The images in the figure are credited as “Illustrations

copyright 2013 Stephen D. Nash / IUCN SSC Primate Specialist Group. Used with permission". MYA: million years ago. The numbers on each branch correspond to the numbers of gene families that have expanded (red) and contracted (green) in mammalian genome. MRCA: most recent common ancestor.

Table 1. Reads generated from five different sequencing methods

Pair-end libraries	Insert size (bp)	Total clean data (Gb)	Read length (bp)	Sequence coverage (X)
Illumina reads	350	423.32	150	133.12
Pacbio reads	20 k	304.84	\	95.86
10X Genomics	500 -700	348.41	150	109.56
Bionano	\	463.75	\	145.83
Hi-C	350	310.92	\	97.77
Total	\	1,851.24	\	582.15

Note: The sequence coverage was calculated with an estimated genome size of 3.18 Gb. The sign of backslash indicates that the insert size was absent.

Table 2. The final genome assembly statistics of *R. roxellana*

Sample ID	length		number	
	Contig[a] (bp)	Scaffold (bp)	Contig[b]	Scaffold
Total	3,038,184,325	3,038,467,325	6,099	3,269
Max	30,757,641	206,558,726	\	\
Number >= 2000	\	\	5,708	2,879
N50	5,723,610	144,559,847	151	9
N60	4,241,389	141,075,955	211	11
N70	3,173,235	135,203,321	292	14
N80	2,063,823	118,350,466	408	16
N90	896,517	83,045,532	622	19

Note: [a] Contig after scaffolding. The sign of backslash indicates that the length/number was absent.

Table 3. Summary of predicted protein-coding genes and their characteristics

Gene set		Number	Average transcript length (bp)	Average CDS length (bp)	Average intron length (bp)	Average exon length (bp)	Average exons per gene
De novo	Augustus	32,928	23,441	1,052	196	5,112	5.38
	GlimmerHMM	618,957	4,204	404	166	2,654	2.43
	SNAP	97,298	49,851	755	144	1,1597	5.23
	Geneid	36,863	35,242	1,035	188	7,615	5.49
	Genscan	50,419	40,635	1,137	167	6,800	6.81
Homolog	Ggo	25,281	19,893	1,055	184	3,971	5.74
	Hsa	38,444	14,763	826	182	3,942	4.54
	Mmu	21,959	29,709	1,470	187	4,123	7.85
	Rbi	25,320	25,685	1,387	196	3,991	7.09
	Rro	24,121	28,439	1,420	185	4,043	7.68
RNASeq	PASA	66,620	28,449	1,219	164	4,247	7.41
	Cufflinks	73,199	31,497	2,737	409	5,052	6.69
EVM		30,102	22,298	1,098	182	4,199	6.05
Pasa-update*		29,403	27,638	1,180	181	4,782	6.53
Final set*		22,497	34,153	1,369	178	4885	7.71

Note: Pasa-update* indicates only the UTRs（Untranslated regions）were considered during the filter process, and other regions were not included. Final set* indicates the results were acquired following the Pasa-update process, with the criteria of the longest isoform was chosen if there were multiple splicing isoforms, and the redundant single exons were also discarded.

Supplementary files:

Supplementary Fig. S1. Genome size estimation using k-mer method

Supplementary Fig. S2. The comparison of each element in the genome of homologous species

Supplementary Table S1. The results of assembly with PacBio long reads and gap filling

Supplementary Table S2. The results of assembly with Bionano optical map data

Supplementary Table S3. The results of assembly with 10X Genomics link reads

Supplementary Table S4. The mapping rate of reads and coverage of assembled genome with BWA

Supplementary Table S5. Assessment results by using BUSCO annotation

Supplementary Table S6. The completeness test results of assembled genome with CEGMA software

Supplementary Table S7. Results of repeats elements predictions from the genome assembly

Supplementary Table S8. The results of TEs elements predicted from the genome assembly

Supplementary Table S9. Summary of predicted RNAs and their characteristics

Supplementary Table S10. The functional annotation of the genes predicted from *R. roxellana* genome

Supplementary Table S11. The GO annotation results of expansion gene families in *R. roxellana* genome

References

1. Li BG, Pan RL, Oxnard CE. Extinction of snub-nosed monkeys in China during the past 400 years. Int J Primatol 2002; **23:**1227-1244.
2. Luo MF, Liu ZJ, Pan HJ, et al. Historical geographic dispersal of the golden snub-nosed monkey (*Rhinopithecus roxellana*) and the influence of climatic oscillations. Am J Primatol 2012; **74:**91-101.
3. Fang G, Li M, Liu X-J, et al. Preliminary report on Sichuan golden snub-nosed monkeys (*Rhinopithecus roxellana roxellana*) at Laohegou Nature Reserve, Sichuan, China. Sci Rep 2018; **8:**16183.
4. Qi XG, Li BG, Garber PA, et al. Social dynamics of the golden snub-nosed monkey (*Rhinopithecus roxellana*): female transfer and one-male unit succession. Am J Primatol 2009; **71:**670-679.
5. Li H, Meng S-J, Men Z-M, et al. Genetic diversity and population history of golden monkeys (*Rhinopithecus roxellana*). Genetics 2003; **164:**269-275.
6. Qi X-G, Garber PA, Ji W, et al. Satellite telemetry and social modeling offer new insights into the origin of primate multilevel societies. Nat Commun 2014; **5:**5296.
7. Schnable PS, Ware D, Fulton RS, et al. The B73 Maize Genome: Complexity, Diversity, and Dynamics. Science 2009; **326:**1112-1115.
8. Seim I, Fang X, Xiong Z, et al. Genome analysis reveals insights into physiology and longevity of the Brandt's bat *Myotis brandtii*. Nature Communications 2013; **4:**2212.
9. Valenzano DR, Benayoun BA, Singh PP, et al. The African turquoise killifish genome provides insights into evolution and genetic architecture of lifespan. Cell 2015; **163:**1539-1554.
10. Zhou X, Wang B, Pan Q, et al. Whole-genome sequencing of the snub-nosed monkey provides insights into folivory and evolutionary history. Nat Genet 2014; **46:**1303-1310.
11. Kuang W-M, Ming C, Li H-P, et al. The origin and population history of the endangered golden snub-nosed monkey (*Rhinopithecus roxellana*). Mol Biol Evol 2018**:**msy220-msy220.
12. Hong YY, Duo HR, Hong JY, et al. Resequencing and comparison of whole mitochondrial genome to gain insight into the evolutionary status of the Shennongjia golden snub-nosed monkey (SNJ *R-roxellana*). Ecol Evol 2017; **7:**4456-4464.
13. Seo JS, Rhie A, Kim J, et al. De novo assembly and phasing of a Korean human genome. Nature 2016; **538:**243-247.
14. Chaisson MJ, Wilson RK, Eichler EE. Genetic variation and the de novo assembly of human genomes. Nat Rev Genet 2015; **16:**627-640.
15. Jiao YP, Peluso P, Shi JH, et al. Improved maize reference genome with single-molecule technologies. Nature 2017; **546:**524-527.

16. Gordon D, Huddleston J, Chaisson MJP, et al. Long-read sequence assembly of the gorilla genome. Science 2016; **352:**aae0344.

17. Kronenberg ZN, Fiddes IT, Gordon D, et al. High-resolution comparative analysis of great ape genomes. Science 2018; **360:**eaar6343.

18. Bickhart DM, Rosen BD, Koren S, et al. Single-molecule sequencing and chromatin conformation capture enable de novo reference assembly of the domestic goat genome. Nat Genet 2017; **49:**643-650.

19. Luo R, Liu B, Xie Y, et al. SOAPdenovo2: an empirically improved memory-efficient short-read de novo assembler. Gigascience 2012; **1:**18.

20. Martin M. Cutadapt removes adapter sequences from high-throughput sequencing reads. EMBnetjournal 2011; **17:**10 -12.

21. Pendleton M, Sebra R, Pang A, et al. Assembly and diploid architecture of an individual human genome via singlemolecule technologies. Nat Methods 2015; **12:**780-786.

22. Chin CS, Alexander DH, Marks P, et al. Nonhybrid, finished microbial genome assemblies from long-read SMRT sequencing data. Nat Methods 2013; **10:**563-+.

23. Walker BJ, Abeel T, Shea T, et al. Pilon: An Integrated Tool for Comprehensive Microbial Variant Detection and Genome Assembly Improvement. PLoS One 2014; **9**.

24. Adey A, Kitzman JO, Burton JN, et al. In vitro, long-range sequence information for de novo genome assembly via transposase contiguity. Genome Res 2014; **24:**2041-2049.

25. Li H, Durbin R. Fast and accurate short read alignment with Burrows-Wheeler transform. Bioinformatics 2009; **25:**1754-1760.

26. Burton JN, Adey A, Patwardhan RP, et al. Chromosome-scale scaffolding of de novo genome assemblies based on chromatin interactions. Nat Biotechnol 2013; **31:**1119-1125.

27. Simao FA, Waterhouse RM, Ioannidis P, et al. BUSCO: assessing genome assembly and annotation completeness with single-copy orthologs. Bioinformatics 2015; **31:**3210-3212.

28. Parra G, Bradnam K, Korf I. CEGMA: a pipeline to accurately annotate core genes in eukaryotic genomes. Bioinformatics 2007; **23:**1061-1067.

29. Jurka J, Kapitonov VV, Pavlicek A, et al. Repbase Update, a database of eukaryotic repetitive elements. Cytogenet Genome Res 2005; **110:**462-467.

30. Price AL, Jones NC, Pevzner PA. De novo identification of repeat families in large genomes. Bioinformatics 2005; **21 Suppl 1:**i351-358.

31. Benson G. Tandem repeats finder: a program to analyze DNA sequences. Nucleic Acids Res 1999; **27:**573-580.

32. Griffiths-Jones S, Moxon S, Marshall M, et al. Rfam: annotating non-coding RNAs in complete genomes. Nucleic Acids Res 2005; **33:**D121-D124.
33. Nawrocki EP, Kolbe DL, Eddy SR. Infernal 1.0: inference of RNA alignments (vol 25, pg 1335, 2009). Bioinformatics 2009; **25:**1713-1713.
34. Lowe TM, Eddy SR. tRNAscan-SE: a program for improved detection of transfer RNA genes in genomic sequence. Nucleic Acids Res 1997; **25:**955-964.
35. Stanke M, Keller O, Gunduz I, et al. AUGUSTUS: ab initio prediction of alternative transcripts. Nucleic Acids Res 2006; **34:**W435-W439.
36. Majoros WH, Pertea M, Salzberg SL. TigrScan and GlimmerHMM: two open source ab initio eukaryotic gene-finders. Bioinformatics 2004; **20:**2878-2879.
37. Burge C, Karlin S. Prediction of complete gene structures in human genomic DNA. J Mol Biol 1997; **268:**78-94.
38. Guigo R. Assembling genes from predicted exons in linear time with dynamic programming. J Comput Biol 1998; **5:**681-702.
39. Korf I. Gene finding in novel genomes. BMC Bioinformatics 2004; **5:**59.
40. Kent WJ. BLAT - The BLAST-like alignment tool. Genome Res 2002; **12:**656-664.
41. Birney E, Clamp M, Durbin R. GeneWise and Genomewise. Genome Res 2004; **14:**988-995.
42. Grabherr MG, Haas BJ, Yassour M, et al. Full-length transcriptome assembly from RNA-Seq data without a reference genome. Nat Biotechnol 2011; **29:**644-U130.
43. Haas BJ, Delcher AL, Mount SM, et al. Improving the Arabidopsis genome annotation using maximal transcript alignment assemblies. Nucleic Acids Res 2003; **31:**5654-5666.
44. Kim D, Pertea G, Trapnell C, et al. TopHat2: accurate alignment of transcriptomes in the presence of insertions, deletions and gene fusions. Genome Biol 2013; **14:**R36.
45. Trapnell C, Roberts A, Goff L, et al. Differential gene and transcript expression analysis of RNA-seq experiments with TopHat and Cufflinks. Nat Protoc 2012; **7:**562-578.
46. Haas BJ, Salzberg SL, Zhu W, et al. Automated eukaryotic gene structure annotation using EVidenceModeler and the program to assemble spliced alignments. Genome Biol 2008; **9:**R7.
47. Bairoch A, Apweiler R, Wu CH, et al. The Universal Protein Resource (UniProt). Nucleic Acids Res 2005; **33:**D154-D159.
48. Kanehisa M, Goto S. KEGG: kyoto encyclopedia of genes and genomes. Nucleic Acids Res 2000; **28:**27-30.
49. Mitchell AL, Attwood TK, Babbitt PC, et al. InterPro in 2019: improving coverage, classification and access to protein sequence annotations. Nucleic Acids Res 2019; **47:**D351-D360.

50. Finn RD, Coggill P, Eberhardt RY, et al. The Pfam protein families database: towards a more sustainable future. Nucleic Acids Res 2016; **44:**D279-D285.
51. Ashburner M, Ball CA, Blake JA, et al. Gene Ontology: tool for the unification of biology. Nat Genet 2000; **25:**25-29.
52. Li H, Coghlan A, Ruan J, et al. TreeFam: a curated database of phylogenetic trees of animal gene families. Nucleic Acids Res 2006; **34:**D572-D580.
53. Guindon S, Delsuc F, Dufayard J-F, et al: Estimating maximum likelihood phylogenies with PhyML. In *Bioinformatics for DNA sequence analysis.* Springer; 2009: 113-137
54. Posada D. jModelTest: phylogenetic model averaging. Mol Biol Evol 2008; **25:**1253-1256.
55. Yang Z. PAML 4: Phylogenetic Analysis by Maximum Likelihood. Mol Biol Evol 2007; **24:**1586-1591.
56. De Bie T, Cristianini N, Demuth JP, et al. CAFE: a computational tool for the study of gene family evolution. Bioinformatics 2006; **22:**1269-1271.

Fig 1

Click here to access/download;Figure;fig 1.JPG

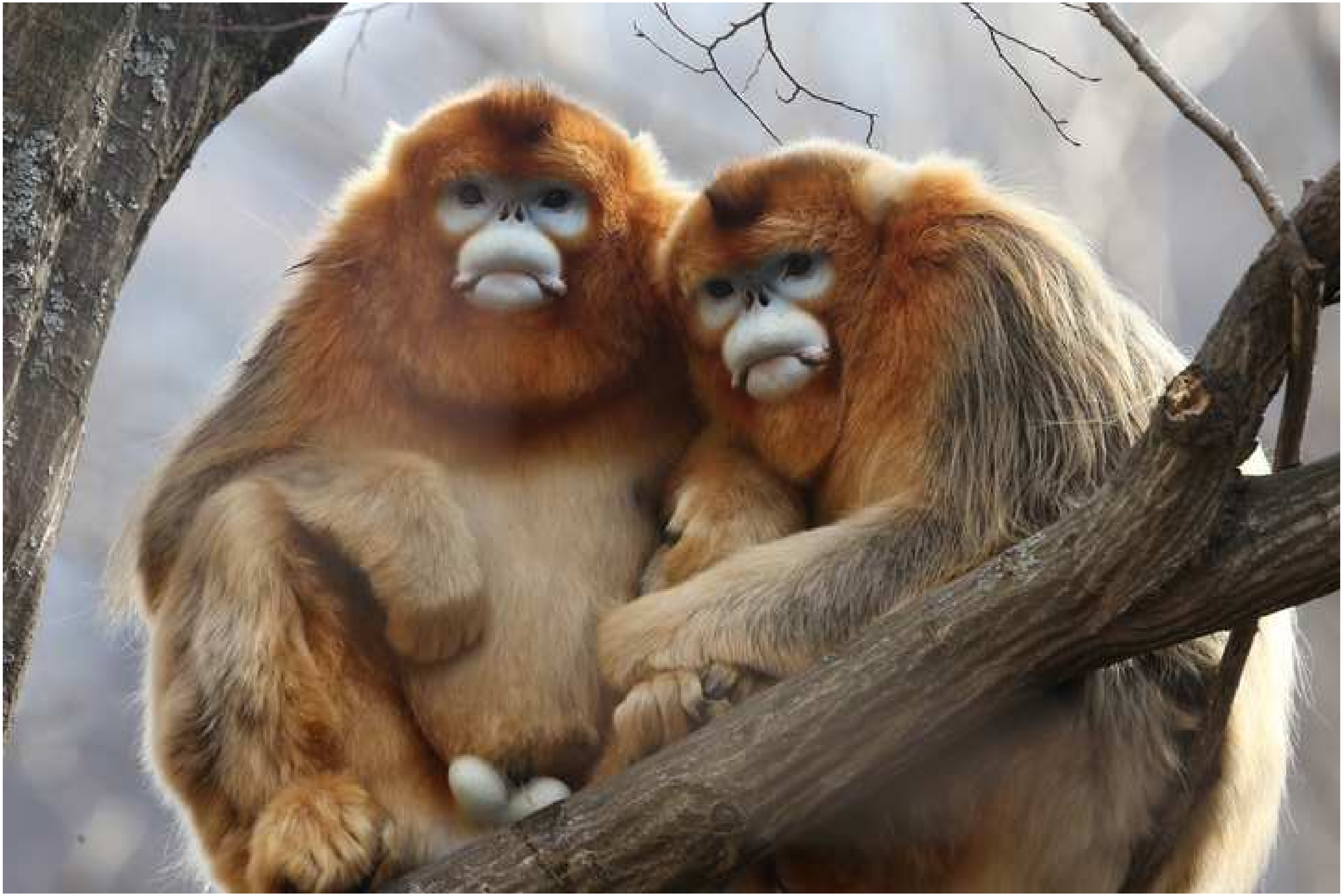

Fig 2

Click here to access/download;Figure;fig 2_Hic.pdf

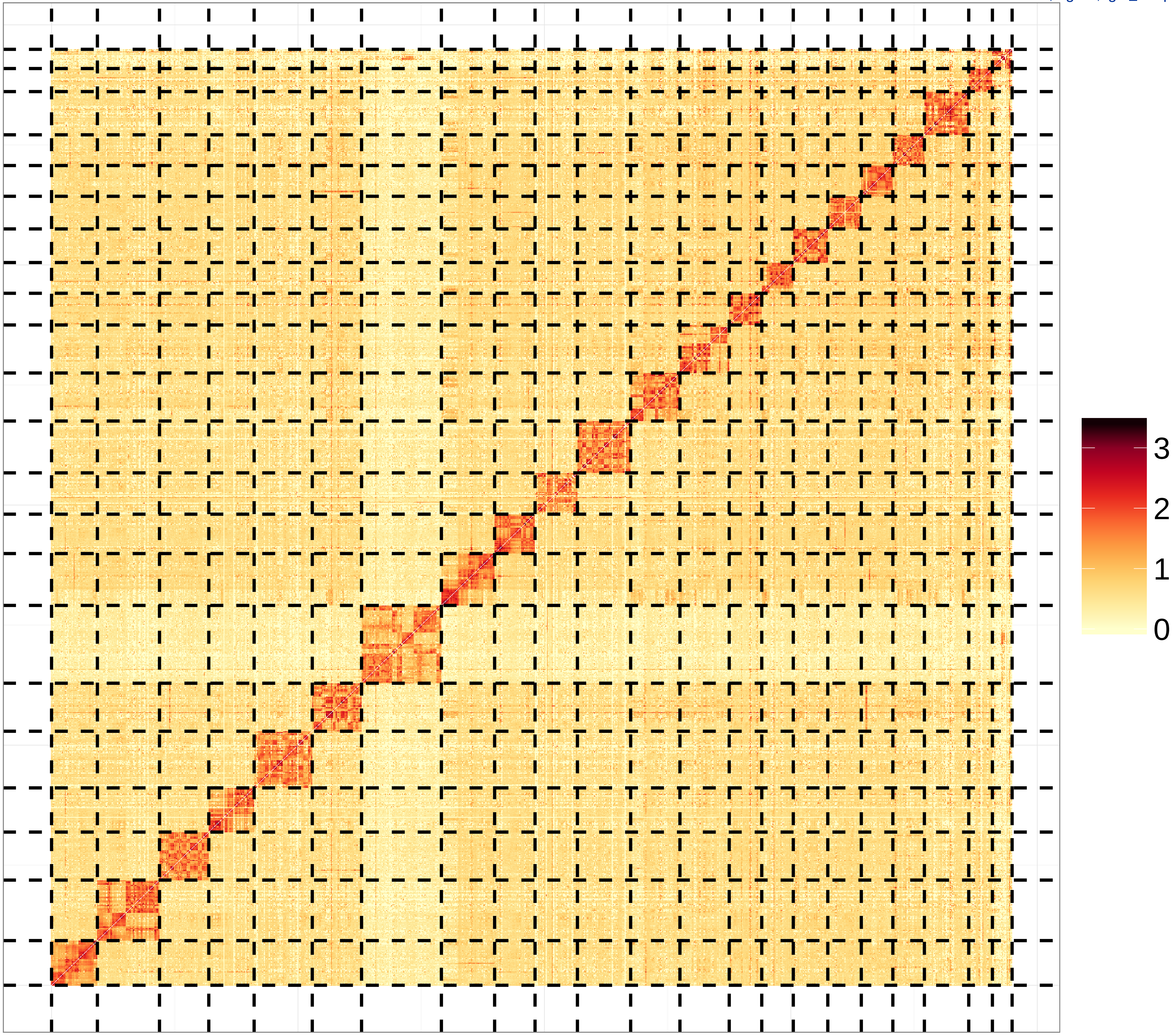

 Click here to access/download;Figure;fig 3.pdf

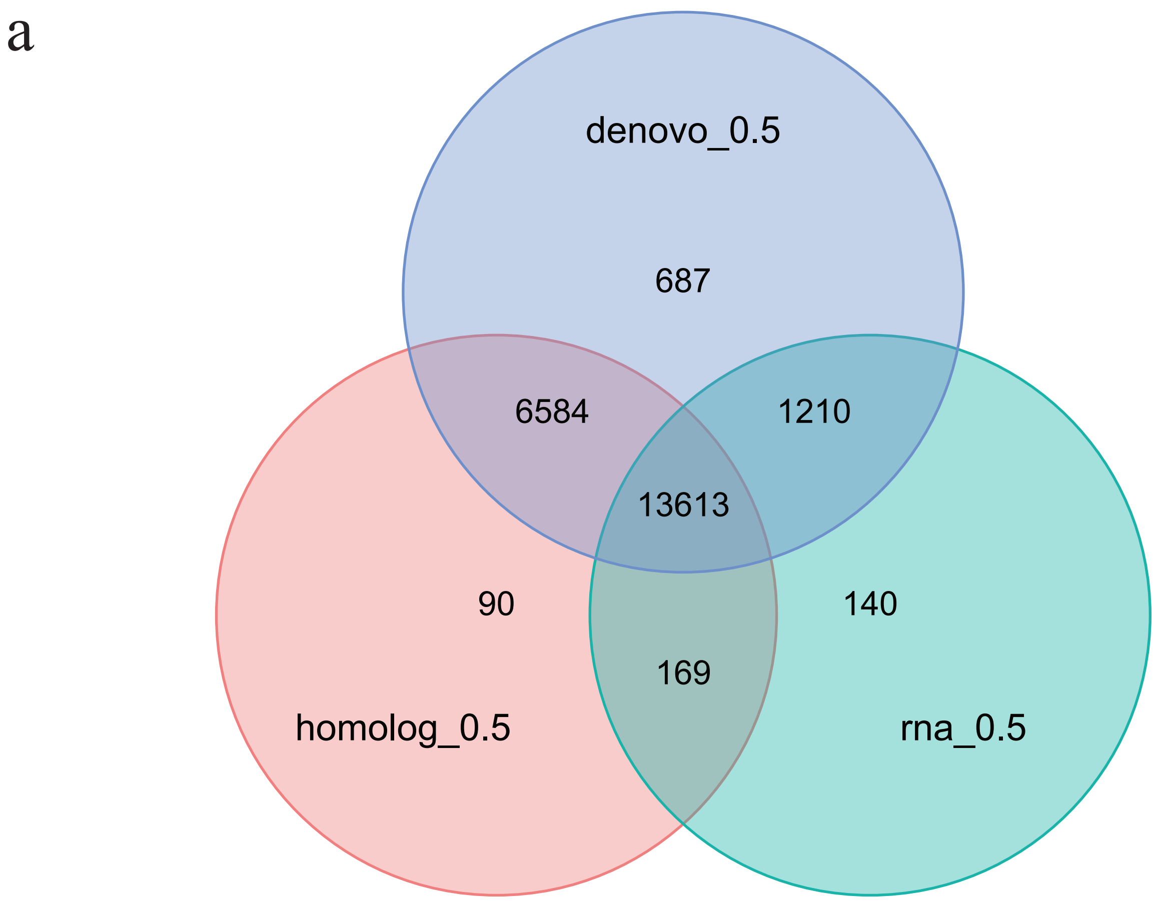

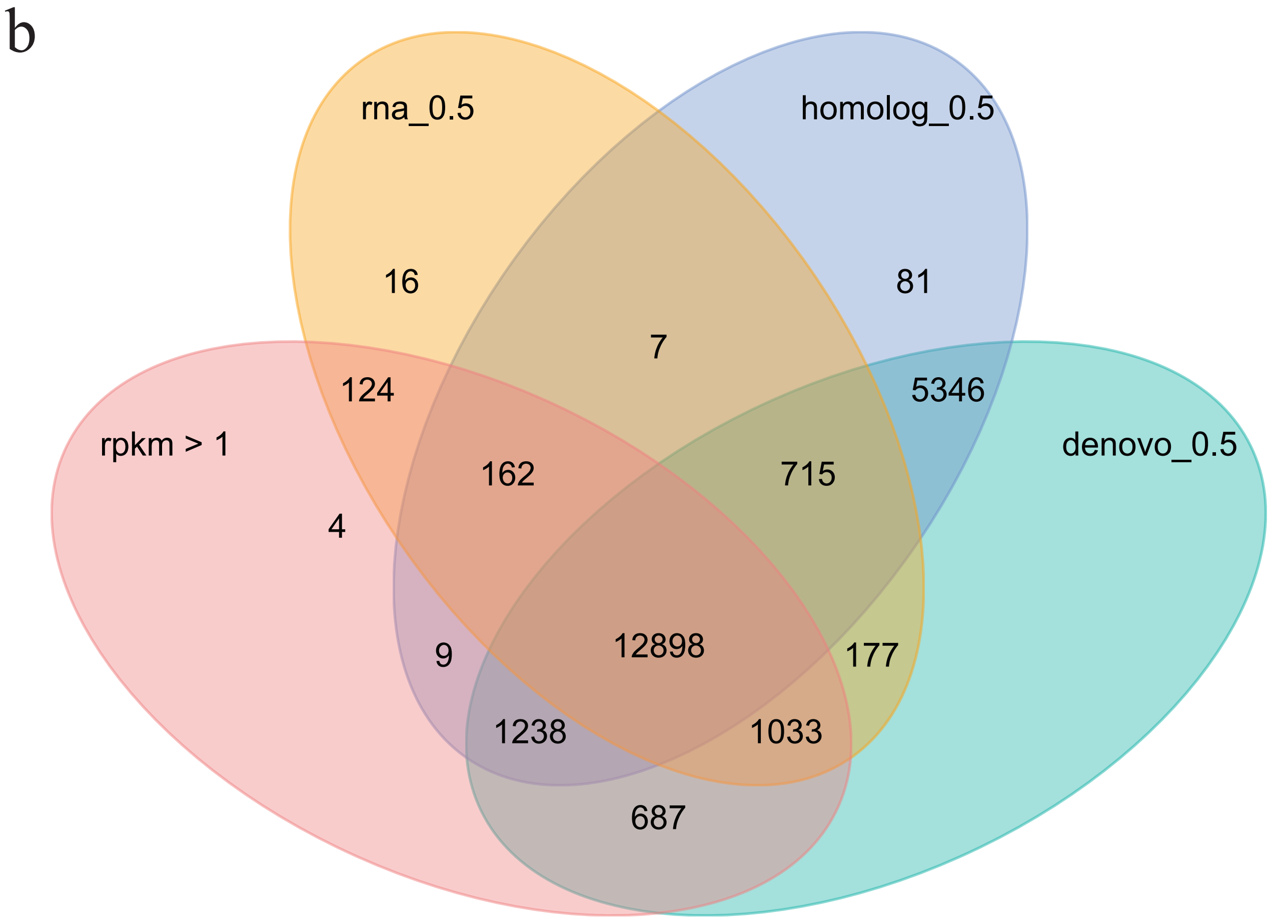

Fig 4

Click here to access/download;Figure;fig 4.pdf

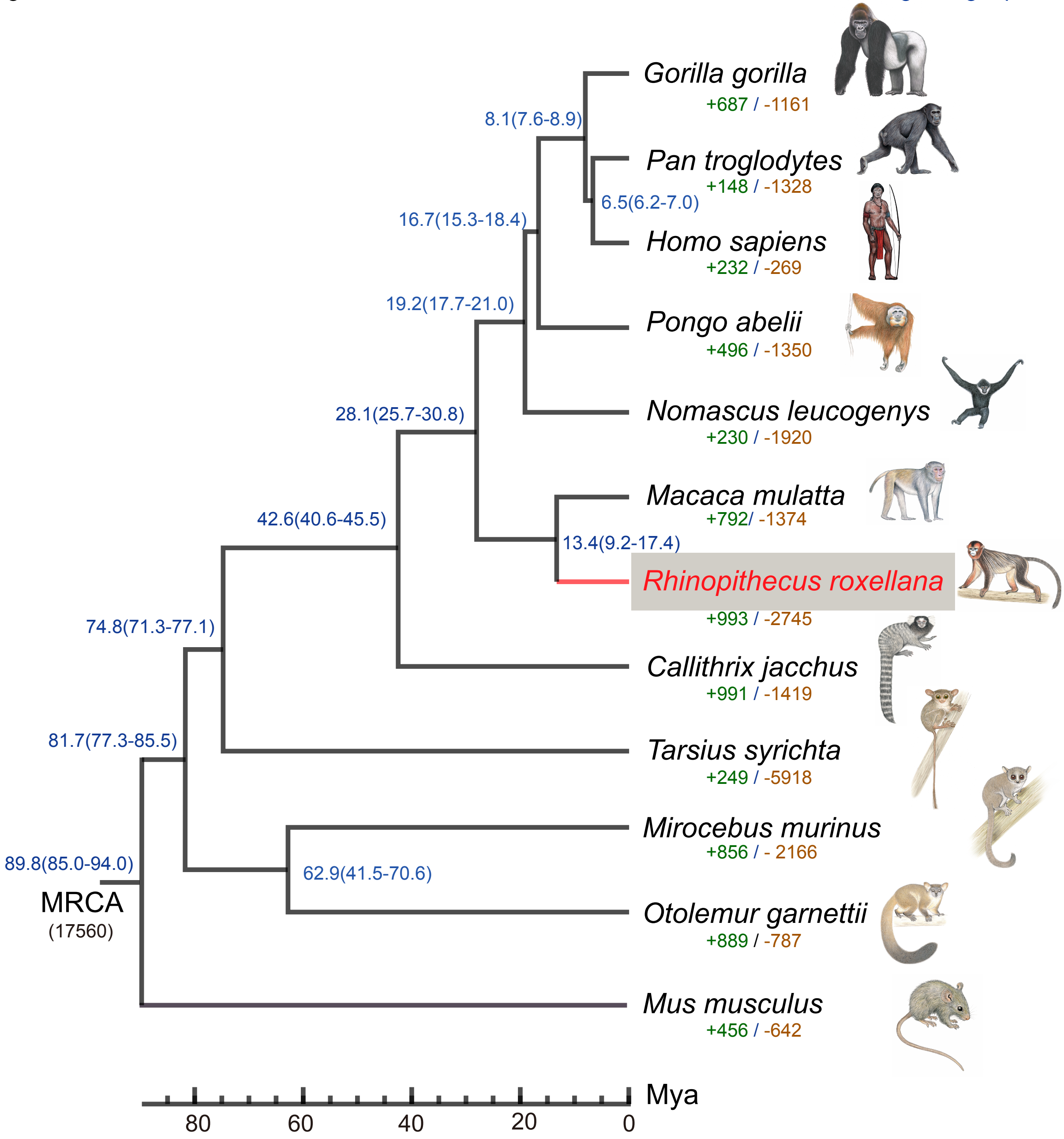

Click here to access/download
Supplementary Material
SI_V1.docx

Cover letter

Click here to access/download;Personal Cover;Cover letter_giga.docx

March 12, 2019

Dear *GigaScience* editor,

Thanks a lot for having reviewed our manuscript titled **"A high-quality genome assembly of the endangered golden snub-nosed monkey *Rhinopithecus roxellana*"** for consideration of publication in *GigaScience*. Now we have revised the manuscript according to the reviewer's comments. Most explanations regarding the revisions of our manuscript are as follows.

1 The raw data discussed in this publication have been deposited in NCBI's short read archive under the accession number PRJNA524949. Supporting data are available in the GigaDB database.

2 We have attached a photo of *Rhinopithecus roxellana* taken in the Qinling mountains in the attached file "Figures" (fig 1).

3 We have improved the language by a native speaker.

4 We have updated the abstract, introduction and conclusion.

4 We have improved the introduction to highlight the reason why we sequence the species (On Page 6, Line 84-89).

5 We demonstrated our genome improvement by comparing with previously reported (On Page 9, Line 143-149).

As a result, we believe that our work will be of general interest to a broad scientific audience including evolutionary biologists, phylogeneticists, geneticists, zoologists, ecologists, and popular media. Thank you for your time and consideration.

Yours sincerely,

Xiao-Guang Qi

Shaanxi Key Laboratory for Animal Conservation

College of Life Sciences

Northwest University

Email: qixg@nwu.edu.cn
