# Supplementary material for: A high-quality genome assembly for the endangered golden snub-nosed monkey (Rhinopithecus roxellana)
Source: Gigascience. 2019 Aug 22;8(8):giz098. doi: 10.1093/gigascience/giz098 (PMC6705546; doi:10.1093/gigascience/giz098)
Supplement: giz098_GIGA-D-19-00030_Revision_1 [file giz098_giga-d-19-00030_revision_1.pdf]

GigaScience

A high-quality genome assembly for the endangered golden snub-nosed monkey (Rhinopithecus roxellana)

--Manuscript Draft--

Manuscript Number:	GIGA-D-19-00030R1
Full Title:	A high-quality genome assembly for the endangered golden snub-nosed monkey (Rhinopithecus roxellana)
Article Type:	Data Note
Funding Information:	National Natural Science Foundation of China (31622053) — Dr. Xiao-Guang Qi
Abstract:	Background: The golden snub-nosed monkey (Rhinopithecus roxellana) is an endangered colobine species endemic to China. This species has several distinctive traits and is an ideal model for analyses of the evolutionary development of social structures due to its unique social organization. Although a genome assembly for the subspecies R. roxellana hubeiensis is available, this assembly is incomplete and fragmented because it was constructed using short read sequencing technology. Thus, information important for the understanding of R. roxellana, such as genome structural variation and repeat sequences, may be absent from the available assembly. Therefore, a high-quality reference genome is needed.<br>Findings: To obtain a high-quality chromosomal assembly for R. roxellana qinlingensis, we used five different methods: Pacific Bioscience single-molecule real-time sequencing, Illumina paired-end sequencing, BioNano optical maps, 10X Genomics link-reads, and high-throughput chromosome conformation capture. The assembled genome was ~3.04 Gb, with a contig N50 of 5.72 Mb and a scaffold N50 of 144.56 Mb. This represented a 100-fold improvement over the previously published genome. In the new genome, 22,497 protein-coding genes were predicted, of which 22,053 were functionally annotated. Gene family analysis showed that 993 and 2,745 gene families were expanded and contracted, respectively, in the R. r. qinlingensis genome. The reconstructed phylogeny recovered a close relationship between Rhinopithecus rollexana and Macaca mulatta, and these two species diverged approximately 13.4 MYA.<br>Conclusion: We constructed a high-quality genome assembly of Qinling golden snub-nosed monkey; this genome had superior continuity and accuracy, which might be useful as reference for future genetic studies in this species. In addition, the updated genome assembly might improve our understanding of this species and might be particularly relevant to conservation efforts. Furthermore, this high-quality genome might serve as a new standard reference genome for colobine primates.
Corresponding Author:	Xiao-Guang Qi<br><br>CHINA
Corresponding Author Secondary Information:	
Corresponding Author's Institution:	
Corresponding Author's Secondary Institution:	
First Author:	Xiao-Guang Qi
First Author Secondary Information:	
Order of Authors:	Xiao-Guang Qi
	Lu Wang
	Jinwei Wu
	Xiaomei Liu

Powered by Editorial Manager® and ProduXion Manager® from Aries Systems Corporation

	Dandan Di
	Yuhong Liang
	Yifei Feng
	Suyun Zhang
	Baoguo Li
**Order of Authors Secondary Information:**	
**Response to Reviewers:**	Editor Comments to Author:<br><br>In particular major improvements are required in the writing and we would strongly recommend you use a native English speaker or professional company to improve the writing.<br>Response to comment 1<br>Thanks for this comment, the manuscript has been revised and polished by an English language editing service of LetPub.<br><br>We have strong policies regarding reproducibility and agree with the referees that significant additional methodological detail is required.<br>Response to comment 2<br>Thanks for your comment, we added the methodological details substantially to be clear and straightforward. In addition, we added some key details about the generated data (sequencing, calibration times, N50 length et al.,) and performed several additional analyses including CNVs identification, synteny analysis and SNP calling et al. as you and reviewers suggested.<br><br>On top of including detail on the software versions and setting, we would strongly recommend you capture this detail using protocols.io. You can re-use and adapt the protocols we have stored in our group page or create your own (and if you provide these in stepwise manner we can even upload them for you):<br>Response to comment 3<br>Following this comment, we have captured those methodological details using protocols.io. with our own account. Please check out on the website of "https://www.protocols.io/private/EAFC44C786ABCE2257FD0D5B9E0D7EF3".<br><br><br><br>Reviewer Comments to Author:<br><br>Reviewer #1:<br>This manuscript reports a new whole genome assembly for an interesting nonhuman primate species, Rhinopithecus roxellana. This is a colobine species that has a number of unusual characteristics, including but not limited to unusual pelage, highly derived facial morphology, and social organization that is not entirely unique but is rare among Old World monkeys or other anthropoids. There are five species in the genus, and all are threatened or endangered, so there is a conservation benefit to this genome sequencing as well as basic comparative primate evolutionary genomics. There is a previously published whole genome assembly for this species, but this new assembly is a significant improvement (see below). Consequently, there are several elements of this work that make it noteworthy.<br><br>The new assembly is based on an effective and technically advanced combination of approaches. The authors began by sequencing this genome using PacBio Sequel long reads, and assembling them using FALCON and PBjelly. The authors generated Illumina short reads and polished the PacBio/FALCON assembly with those. The authors also make use of BioNano optical mapping and 10X linked-reads to increase completeness and contiguity. Finally, Hi-C mapping is used to produce near full chromosome length scaffolds. The result is a 3.04 gigabase assembly with contig N50 of 5.72 Mb and scaffold N50 of 144.56 Mb. These statistics make this one of the most complete and highly contiguous assemblies available for any nonhuman primate (confirmed using BUSCO and CEGMA analyses). The authors then annotated this genome using a series of annotation software tools, and identified 22,497 genes.

Powered by Editorial Manager® and ProduXion Manager® from Aries Systems Corporation

This new genome assembly is a valuable resource for any investigator working on the genetics or genomics of Rhinopithecus. In addition, this is a high quality - high contiguity assembly, so it will be useful for laboratories working on other closely related colobines. Lastly, the authors report some initial analyses of repetitive sequences and gene family expansions and contractions using this new Rhinopithecus assembly.

While this genome sequence seems to be a valuable resource for the primate genomics community, this manuscript has a significant number of serious flaws and problems. One issue is that the quality of the grammar and text is not adequate. I realize that the authors may not be native speakers of English, and that this can be a challenge. But this manuscript needs major assistance in terms of editing before it is ready for serious consideration.
Response to this comment
Thanks for this comment, the manuscript has been revised and polished by an English language editing service of LetPub.

I have other specific concerns as well.
1) This is minor but having two different line numbering systems printed on the same pages causes confusion. I will use the numbers that are actually tied to specific lines in the text, rather than the more densely packed numbers that seem to just run down each page. The authors should delete the dense numbers.
Response to comment 1
Thanks for your kindly review. We deleted the dense numbers.

2) Page 4, lines 54-56. While the social organization of Rhinopithecus roxellana is interesting and deserves more study, it seems overly optimistic for the authors to argue that production of this genome assembly will ultimately support genetic studies that make contributions to our "…understanding the behavior patterns of human society in social-anthropology." Studies of comparative social relationships and social organization are important and primates can provide information about human evolution. But this statement seems to me to be overly ambitious in terms of research outcomes.
Response to comment 2
Thanks for this comment. We changed the statement as follows:
"Therefore, R. roxellana is an ideal model for the analysis of social structure evolution in primates and may also provide opportunities to investigate evolutionary and socio-anthropological patterns of human society.".

3) There are mistakes in capitalization and spelling of words. For example, the Shennongjia Mountains are not capitalized in line 63, but "Gorillas" is incorrectly capitalized in line 75 and "Colobine" is regularly capitalized when it need not be. "Quiver" is misspelled in line 125.
Response to comment 3
Corrected. We also checked other mistakes in capitalization and spelling of words throughout the manuscript.

4) Line 75 states the gorillas and orangutans "…have the closest genetic relationship with humans" but of course that is chimpanzees and bonobos, not gorillas and/or orangutans.
Response to comment 4
Thanks for this comment. We are sorry that we made a mistake here and we changed the statement as follows:
"New sequencing technologies, including Pacific Bioscience's single-molecule real-time (SMRT) sequencing, BioNano optical mapping, and Hi-C-based chromatin interaction maps, have been used in several species closely related to humans, including gorillas (Gorilla gorilla gorilla) [17], chimpanzees (Pan troglodytes) [18], and Sumatran orangutans (Pongo abelii) [18], as well as in other species, including the domestic goat (Capra hircus) [19].".

5) I think the language in line 86 is a bit too optimistic and ambitious. The authors state that this assembly "…may allow us to comprehensively understand R. roxellana…". I do not know what it would mean to "comprehensively understand" a primate species, but I do not think we are yet close to that point.
Response to comment 5

Thanks for this comment. We changed the statement as follows:
“This updated genome assembly may allow us to further investigate R. roxellana, providing new opportunities to analyze evolutionary history and to identify genetic changes associated with the development of specific traits in this species”.

6) Page 6, line 87: It is not clear to me what the authors mean by "genetic-specific signatures of this species"?
Response to comment 6
Thanks for this valuable comment. In fact, we were intended to term those genetic changes associated with the development of species-specific traits as “genetic-specific signatures of this species”. We realized that this sentence was confusing and not clear enough. We changed the statement as follows:
“genetic changes associated with the development of specific traits in this species”.

7) Page 6, line 93-94. Was the animal used to produce the DNA for the sequencing wild-caught or captive bred at Louguantai? If captive bred, were the parents wild-caught?
Response to comment 7
Thanks for this comment. The animal used for the sequencing was an adult male R. roxellana qinlingensis in Qinling Mountain. The animal that died naturally in Qinling Mountain was immediately stored in ultra-cold storage freezer at Louguantai Breeding Centre. We reworded the statement as follows:
“The animal used for sequencing was an adult male R. r.. qinlingensis from Qinling Mountain, who died naturally and the dead body was stored in ultra-cold storage freezer at Louguantai Breeding Centre, Xi’an, Shaanxi Province, China.”

8) Page 7, lines 103-105. BioNano optical mapping is a technique for using restriction enzymes to nick and label DNA at short known target sequences. The map of nicked sites is used to scaffold a genome or confirm the organization of contigs. It is not clear what the authors mean when they state that they "…acquired 463.75 Gb clean reads" from the BioNano Genomics Irys platform. There are no sequence reads generated by the Irys platform. This section does not make sense to me. Instead, the authors should present the actual results of the optical mapping in terms of the number of sites examined and the concordance between the observed BioNano map and the predicted map based on the assembled contigs and scaffolds.
Response to comment 8
Thanks for your valuable comments. We are sorry that we used the wrong term here. Of course, there are no sequence reads generated by the Irys platform, the generation by which should be large DNA molecules. As for the number of sites examined in this study, the average label density for the BioNano map is 11.66 per 100 kb, while the average label density is 12.62 per 100 kb for the predicted map based on those assembled contigs and scaffolds. Thus, the observed BioNano map is consistent with the predicted map. We added several sentences to clarify this point.
“The average label density examined for the BioNano map is 11.66 per 100 kb, while the average label density is 12.62 per 100 kb for the predicted map based on the assembled contigs. Thus, the observed BioNano map is consistent with the predicted map. The BioNano map generated 463.75 Gb of large DNA molecules.”

9) I do not think that Figure 2 adds much to this paper. The authors used Hi-C for scaffolding, and that does provide useful data. But simply inserting a figure showing Hi-C interaction frequencies without doing any further analysis of the details of DNA-DNA interaction or characterizing the topologically associating domains provides no significant new information or insight.
Response to comment 9
Thanks for your valuable comment. The fig. 2 was based on the interaction frequencies between pairs of 100-kb genomic regions. In principle, higher counts indicate increased frequency of chromatin interaction and closer spatial distance between the two sequences, darker red means stronger interaction strength. This strategy has significantly advanced the assembly quality with chromosome-length scaffolds. The fig. 2 presented here was used to indicate the reliability of our assembly.
As for the further analysis of the details of DNA-DNA interaction or characterizing the topologically associating domains, we agree that these analysis were useful. However, they may be beyond the scope of this report, which aims to report a high-quality genome for further studies. We also added several sentences to make the figure

Powered by Editorial Manager® and ProduXion Manager® from Aries Systems Corporation

legend of fig. 2 more clear.
“Hi-C interactions within and among chromosomes of R. roxellana chromosomes (Chr1–Chr22); interactions were drawn based on the chromatin interaction frequencies between pairs of 100-kb genomic regions (as determined by Hi-C). In principle, darker red cells indicate stronger and more frequent interactions, which in turn imply that the two sequences are spatially close.”

10) Page 9, lines 151-152. I do not understand the sentence that begins "With a ratio number…"
Response to comment 10
Thanks for this comment. We reworded this sentence to clarify this point.
“Approximately 99.17% of the short reads were mapped to the genome assembly. Further investigations indicated that these reads covered approximately 99.27% of the total assembly (Supplementary Table S6).”.

11) Page 9-10, lines 152-159. Using BUSCO and CEGMA to assess the completeness of the genome assembly is a very good idea. But the authors should report not just how many BUSCO or CEGMA genes were identified, but how many were complete and unfragmented and how many were complete and fragmented.
Response to comment 11
Thanks for this comment. During the BUSCO analysis, the annotation results were classified as complete BUSCOs, fragmented BUSCOs and missing BUSCOs. We did not report those results in the manuscript, however, these detailes were shown in Supplementary Table S8. Simply, the complete BUSCOs occupied a proportion of 94.0%, while the fragmented BUSCOs occupied only 2.9%. In addition, we added the CEGMA results in Supplementary Table S9, which showed that the 220 genes were complete and unfragmented , while 13 was complete and fragmented. We also added these results in our manuscript.
“In addition, we estimated assembly completeness using Benchmarking Universal Single-copy Orthologs (BUSCO) v3.0.2 [27], with the parameters “-i -o -l -m genome -f -t.” based on mammalia_odb9 (creation date: 2016-02-13; number of species: 50; number of BUSCOs: 4,104). BUSCO analysis identified 4,104 mammalian BUSCOs in the newly assembled R. roxellana genome: 94.0% complete BUSCOs, 2.9% fragmented BUSCOs, and 3.1% missing BUSCOs (Supplementary Table S8). Assembly completeness was measured using the core eukaryotic gene (CEG)-mapping approach (CEGMA v2.5) [28]. Of the 248 CEGs known from six model species, 93.95% (233 of 248) were identified in our new genome assembly. Of these, 220 CEGs were complete and unfragmented, and the remaining 13 were complete but fragmented (Supplementary Table S9). Together, these analyses indicated that our new genome assembly was highly accurate and complete.”

12) Page 12, lines 226-227. What fossil calibration times were used?
Response to comment 12
Thanks for this comment. The fossil calibration times were derived from Timetree (http://www.timetree.org/). The following calibration times were used: Homo sapiens VS Callithrix jacchus (40.6-45.7 MYA); Homo sapiens VS Pan troglodytes (6.2~7 MYA); Homo sapiens VS Mus musculus (85-94 MYA) and Homo sapiens VS Tarsius syrichta (71~77 MYA). We also added these fossil calibration times in our manuscript.
“The following fossil calibrations were used: Homo sapiens vs. Callithrix jacchus (40.6–45.7 MYA, million years ago); Homo sapiens vs. Pan troglodytes (~6.2–7 MYA); Homo sapiens vs. Mus musculus (85–94 MYA); and Homo sapiens vs. Tarsius syrichta (~71–77 MYA). ”

13) Page 14, lines 232-235. Rhinopithecus gene families were expanded or contracted compared to what taxa? Compared to human? Compared to the ancestral primate genome? Compared to an Old World monkey outgroup?
Response to comment 13
Thanks for this comment. The expansion and contraction of gene families of Rhinopithecus roxellana were estimated by comparing those of the most recent common ancestor between Rhinopithecus roxellana and Macaca mulatta. We added one sentence in the figure legend of fig. 4 to clarify this point.
“Numbers under each species indicate the number of gene families that have been expanded (green) and contracted (light yellow) since the split of species from the most recent common ancestor (MRCA).”.

14) I think two column headings in Table 3 are switched. I doubt that the average intron length for the Rhinopithecus Augustus gene models is 196bp, while the average exon length for the same gene models is 5,112bp. Seems to me those two labels are probably switched.
Response to comment 14
Thanks for your valuable comment. We are sorry that we made a mistake here, we put them in right order now. Please see Table 3 for details.

Reviewer #2:
The authors present an assembly of golden snub-nosed monkey using a range of sequencing technologies, including long read sequencing. Overall the manuscript is mostly clear to follow and the assembly approaches are standard and appear to be well performed. A very large amount of data was generated, although the methods are very short and some details are lacking, it appears that standard and appropriate assembly approaches were used. Some key details about the generated data are missing, and there are some additional analyses that, if completed, would greatly improve the manuscript.
Response to comment 1
Thanks for your valuable comment. we added the methodological details substantially to be clear and straightforward. Please see the "De novo assembly" section for details. In addition, some key details about the generated data (N50 length, software parameters et al.) were present this time and several additional analyses including CNVs identification, synteny analysis and SNP calling et al. were also performed as suggested.

I could not find descriptions of the characteristics of the generated data, particularly average/n50 length of Pacbio reads, molecule size of the optical mapping and of 10X data. These are key parameters that should be reported.
Response to comment 2
Thanks for this comment. The average/N50 length of Pacbio reads and molecule size of the optical mapping was 16.69 kb and 338 kb, respectively. As for the 10X data, since paired-end of 350 bp sequencing was performed, N50 length was not applicable for this case. It was estimated that a total of 423.32 Gb clean reads were generated for 10X data. We added one sentence to descript the characteristics of the generated data.
"…., the average/N50 length of Pacbio reads was 16.69 kb."
"The average/N50 length of the molecules used for optical mapping was 338 kb.".

Line 104 The description of the Bionano data should be clarified. I am not sure that "reads" is the right term for data from this optical mapping platform. Same for term 'sequence coverage' for optical mapping data in Table 1.
Response to comment 3
Thanks for this comment. We added several sentences to detail the BioNano data. We agree that no reads generated from optical mapping platform and we changed the term of "reads" as molecules. Also, the term "sequence coverage" was not an proper term for optical mapping data, we removed the sequence coverage value of optical mapping data in Table 1.
"The average/N50 length of the molecules used for optical mapping was 338 kb. The average BioNano optimal marker density was 11.66 per 100 kb, while the average marker density was 12.62 per 100 kb for the predicted map based on the assembled scaffolds. Thus, the observed BioNano map was consistent with the predicted map. The BioNano map generated 463.75 Gb of large DNA molecules."

The manuscript would benefit from some comparison of how much better the gene annotation is relative to previous assembly, but this and other biological/comparative analyses may be beyond the scope of this report.
Response to comment 4
Thanks for this comment. As for the gene annotation, our new assembly was better than previous assembly from at least two aspects. Firstly, we assessed genome assembly completeness by mapping transcriptome unigenes to the two assembly versions using BLAT v.36. Results showed that the completeness degree (percentage

Powered by Editorial Manager® and ProduXion Manager® from Aries Systems Corporation

of unigenes aligned to a single scaffold in genome) was higher in our assembly (95.35%) compared with that in previous assembly (89.28%) for unigenes larger than 1000 bp (Supplementary Table S15), demonstrating the contiguity of our new assembly. Secondly, the number of genes annotated to the public database to the total number of predicted genes was higher in our new assembly (98.03%) than that in previous version (94.52%).

From Supplementary Tables S2-3, it seems that the largest increase in n50 scaffold length came from 10X linked read data, not from the bionano optical map. I do not think this is expected, given that optical map data should provide very long range information. The manuscript would be clearer for the reader if some description for why such a gain was found from 10X data was described, and if such results are typical.
Response to comment 5
Thanks for this valuable comment. We checked our assembly description carefully and found some details were not shown. Actually, the first stage of assembly was conducted mainly from three procedures: (a). PacBio long reads assembly using the falcon pipeline, assembly was further polished by Quiver and Pilon-1.18 (contig N50: 4.7 Mb); (b). SSPACE-LongRead (version 1-1) was implemented for getting a longer scaffold (contig N50, 4.7 Mb; scaffold N50, 7.8 Mb); (c). PBjelly was used to close gaps (contig N50, 5.7 Mb; scaffold N50, 8.2 Mb). As you see, the increase of scaffold N50 in this stage mainly came from SSPACE-LongRead procedure (7.8 Mb VS 4.7 Mb). Then the assembled PacBio scaffolds were used as input for scaffolding by hybridScaffold software at the BioNano stage, which generated a hybrid assembly with scaffold N50 of 9.22 Mb. It seems that BioNano optical map did not increase N50 too much (9.22 Mb VS 8.2 Mb), we predicted that the main reason was the employment of SSPACE-LongRead procedure during the first stage assembly. This program dealt with the scaffold construction effectively and the efficiency may be overlap with the performance at the BioNano stage in our study. Therefore, it was reasonable the increase in scaffold N50 was not largely from the BioNano optical map stage. Following this, the 10X genomic linked reads were employed to construct larger scaffolds, fragScaff software was employed to finish the super-scaffold construction. This procedure has increased the genome assembly with a scaffold N50 of 24.09 Mb, suggesting the efficiency of 10X genomic linked reads in our work (24 Mb VS 9.2 Mb). The efficiency of 10X genomic linked reads was also seen in other publication (Mostovoy et al., 2016, Nature Methods), which shows that 10X linked read data contributes more to the increase in N50 length than the BioNano optical map. Despite this, we still did not know whether the largely increase from 10X data was typical or not, as only few publications were available using the combination of 10X reads and BioNano map. We added several sentences to expand our method section, particularly in the “De novo assembly of the R. roxellana genome” section.

Standard repeat masker, gene prediction, and other analysis is performed. The manuscript would be strengthened by also a consideration of duplicated sequences, which could be identified based on Illumina sequence data read depth. This may be beyond scope of this report, but could be considered.
Response to comment 6
Thanks for this comment. We added duplicated sequences/copynumbervariant (CNVs) analysis based on read depth estimated from illumine short reads to the assembled genome using BWA. Results showed that a total of 676 duplicated blocks were identified, whose total length was 9,198,900 bp. We added one paragraph to clarify this point.
“We also performed a CNV analysis. In brief, we first mapped the Illumina short reads to the assembled genome using BWA with default parameters. Then, the sorted mapping bam file was used as input for CNVnator v0.3.3 [38], with the parameters “-unique -his 100 -stat 100 -call 100.”. The obtained CNVs were filtered, retaining only those where q0 was <0.5 and e-val1 was <0.05. After filtering, 676 CNVs remained, with a total length of 9,198,900 bp (Supplementary Table S12). ”.

Has the assembly itself been submitted to proper databases and repositories (such as Genbank)? I could not find this listed, only the raw data.
Response to comment 7
Thanks for this comment. The genome assembly and other supporting data have been submitted to GigaDB database and NCBI successfully. However, we did not release them now as interest competition exist and several research groups are also working

on this species. We appreciate the editor and reviewers understand the challenges in this case, and we will make related data available once this article is published.

In table 2 and others, what does the 'number' column mean? For example, are there 151 contigs >= to the N50 length of 5.7mb? The meaning of the columns in the tables should be clearly explained.
Response to comment 8
Thanks for this comment. Yes, this example explains the exact mean of 'number' column. Following your comment, we revised Table 2 to be more clear. We added one sentence to explain the meaning of the 'number' column. In addition, we checked and revised other tables if not clearly explained (for example, Table 3 and Supplementary Table S1).
"The "Number" column represents the number of contigs/scaffolds longer than the value of the corresponding category.".

The legend for figure 2 is not adequate. What does the color scale signify? What is the reader supposed to conclude from the figure?
Response to comment 9
Thanks for this comment. This plot shows the interactions between two 100-kb genomic regions (as determined by Hi-C), darker red means stronger interaction strength. We added two sentences in the figure legend to address this comment.
"Hi-C interactions within and among chromosomes of R. roxellana chromosomes (Chr1–Chr22); interactions were drawn based on the chromatin interaction frequencies between pairs of 100-kb genomic regions (as determined by Hi-C). In principle, darker red cells indicate stronger and more frequent interactions, which in turn imply that the two sequences are spatially close."
This figure tries to express the information of Hi–C interactions among 22 chromosomes with a 100 kb resolution. Stronger interactions are indicated in darker red and weak interactions are indicated in light yellow. The fig. 2 presented here was used to indicate the reliability of our assembly during the Hi-C stage.

Reviewer #3:
Wang, Wu et al. have produced a high-quality reference genome assembly for the emblematic golden snub-nosed monkey. The authors used a combination of long PacBio reads, 10-X linked reads, Hi-C contact maps, BioNano Optical maps, and Illumina paired end sequences, all of which were sequenced to a very high coverage. The resulting assembly has very high continuity and given the combination of different sequencing strategies essentially gives as good of an assembly as current methods can produce. The authors have used a state-of-the-art approach to produce their assembly, and the applied methodology is appropriate. The authors have also produced a gene annotation based on homology to other species, as well as expression data. The assembly provides a valuable genomic resource to study snub-nosed monkeys specifically, and Asian colobines in general.
General comments:

R. roxellana already has a genome assembly available, as the authors note in the manuscript. However, there is no comparison at all beyond a contig and scaffold N50. It would strengthen the manuscript if the authors could provide some comparisons to the previous assembly, e.g: A comparative, or what specific regions of the assembly were absent in the previous version, what do they contain, how many gaps were filled, how many of the gene family expansions/contractions are only detectable with the high quality assembly etc.
Response to comment 1
Thanks for this comment. We followed this comment and made some comparisons with previous assembly, including repeat analysis and synteny analysis. In comparison, our new assembly had a higher proportion of repeat sequences (50.82%) as compared to the previous version (46.15%); in particular, the number of LINE (long interspersed elements) transposable elements and tandem repeats was greatly increased (further details are given in the "Identification of repeat elements" section). Thus, the newly assembled genome was substantially more complete and continuous. Also, we aligned

our genome against the previous version using MUMMER (v4.0.0beta2) and identified a total of 2,217 insertions in our new assembly. These insertion regions were mainly located in the intergenic and repetitive regions. Further analysis showed that 6,452 gaps in the previous version that were predicted to be filled by >29.7 Mb of sequence in our new assembly. These filled gaps were mainly located in the intergenic and repetitive regions, with a small fraction of the sequence data annotated as gene regions.
We added several sentences to clarify this point.
“We evaluated our newly assembled R. roxellana genome against the previously published assembly. The contiguity of our R. roxellana genome was 100fold greater (contig N50: 5.72 Mb; scaffold N50: 144.56) than the previous version (contig N50: 25.5 kb; scaffold N50: 1.55 Mb) [11]. We also aligned our genome against the previous version using MUMMER (v4.0.0beta2) [37] and identified 6,452 gaps in the previous version that were predicted to be filled by >29.7 Mb of sequence in our new assembly. These filled gaps were mainly located in the intergenic and repetitive regions, with a small fraction of the sequence data annotated as gene regions. Our new assembly also had a higher proportion of repeat sequences (50.82%) as compared to the previous version (46.15%); in particular, the number of LINE (long interspersed elements) transposable elements and tandem repeats was greatly increased (further details are given below, in the “Identification of repeat elements” section). Thus, the newly assembled genome was substantially more complete and continuous. It was likely that the remarkable improvement in contiguity was due to the increased read length, deeper sequencing depth, improved gap assembly, and more sophisticated assembly algorithm.”

The authors use several different software packages for their analysis. The inclusions of version numbers for the software packages they used seems somewhat arbitrary. Furthermore, no parameter sets apart from "default parameters" are ever presented. Both package versions and parameter settings should absolutely be included, otherwise the methods of the study are not properly understandable. In its current state, I feel the methodological aspects of the manuscript need to be expanded.
Response to comment 2
Following this comment, we added the methodological details substantially to address this comment. Both package versions and parameter settings were included in this version. please see “De novo assembly of the R. roxellana genome” section and other sentences containing software names in our manuscript for details.

The manuscript will benefit from language editing, as at several points the phrasing is somewhat confusing.
Response to comment 3
Thanks for this comment, the manuscript has been revised and polished by an English-language editing service of LetPub.

Specific comments:
L19, L68, L80: The claim of "incompleteness" or "greatly improved" is not backed by a proper comparison to the previous assembly.
Response to comment 4
We followed this comment and made comparisons with previous assembly, including repeat analysis and synteny analysis. In comparison, our new assembly had a higher proportion of repeat sequences (50.82%) as compared to the previous version (46.15%); in particular, the number of LINE (long interspersed elements) transposable elements and tandem repeats was greatly increased. Also, We aligned our genome against the previous version using MUMMER (v4.0.0beta2) [37] and identified 6,452 gaps in the previous version that were predicted to be filled by >29.7 Mb of sequence in our new assembly. These filled gaps were mainly located in the intergenic and repetitive regions, with a small fraction of the sequence data annotated as gene regions. Most importantly, the newly assembled R. roxellana reference genome has 100fold higher contiguity than previous assembly (contig N50: 5.72 Mb versus 25.5 kb and scaffold N50: 144.56 Mb versus 1.55 Mb).
We added several sentences to address this comment in the “Assessment of the genome newly assembled” section. See also the response to your valuable comment 1.

L22: Genetic-specific signatures is awkwardly phrased.

Powered by Editorial Manager® and ProduXion Manager® from Aries Systems Corporation

Response to comment 5
Thanks for this valuable comment. In fact, we were intended to term those genetic changes associated with the development of species-specific traits as “genetic-specific signatures of this species”. We realized that this sentence was confusing and not straightforward. We changed the statement as follows:
“genetic changes associated with the development of specific traits in this species”.

L25: Technology, not technique
Response to comment 6
Thank you for your kindly review. We did it.

L57: This sentence is vague, please be specific about what these studies have looked at. The term research-hotspot for this species might be a stretch.
Response to comment 7
Thanks for this comment. Specifically, Recent studies of R. roxellana have focused on behavioral dynamics, population history, and social systems. We removed the term research-hotspot in this sentence.
“Recent studies of R. roxellana have focused on behavioral dynamics, population history, and social systems [5-7],”

L58f: This sentence needs rephrasing. What are the groups?
Response to comment 8
Following this comment, we reworded this sentence and also specify species the groups included.
“Genomic analyses have helped to untangle the molecular evolution of several groups, including maize (Zea mays), bats (Myotis brandtii), and killifish (Nothobranchius furzeri) [8-10]”.

L60: differentiate -> be distinguished
Response to comment 9
Thank you for your kindly review. We did it.

L63: Was there more than one assembly before this study?
Response to comment 10
Thanks for this comment. Actually, there is only one assembly published in 2014 before our study. We reworded this sentence as follows to avoid confusing.
“To date, only a single genome assembly is available for R. roxellana. This assembly, published in 2014, was derived from short sequencing reads generated by the Illumina HiSeq 2000 platform.”

L71f: This sentence needs rephrasing; it is not clear to me what the authors want to say.
Response to comment 11
We followed this comment and reworded this sentence to make it clear enough.
“Indeed, many previously unreported transposable elements and specific genes in maize were identified using an improved reference genome [16].”.

L74,L78: Please be specific with respect to the sequencing technology. "High quality" is subjective and changes with sequencing technologies, so arguing that no "high quality assembly of R. roxellana has been reported" is debatable.
Response to comment 12
Thanks for this comment. These new sequencing technologies used here referred to PacBio SMRT sequencing, BioNano optical mapping, and Hi-C based chromatin interaction maps. Additionally, we agree that "High-quality" is subjective and changes with sequencing technologies. We reworded this sentence to clarify this point.
“However, the R. roxellana genome has not yet been updated using new sequencing approaches, slowing progress towards a better understanding of this endangered species.”.

L75: Ref 15. Also includes an assembly for the Chimpanzee, which is closer to Human than either Gorilla or the Orangutan. 'Widely' should be omitted in this sentence.
Response to comment 13
Following this comment, we added the assembly of the chimpanzee in our manuscript. In addition, we removed 'Widely' in this sentence.

"New sequencing technologies including Pacific Bioscience's single-molecule real-time (SMRT) sequencing, BioNano optical mapping, and Hi-C-based chromatin interaction maps, have been used in several species closely related to humans, including gorillas (Gorilla gorilla gorilla) [17], chimpanzees (Pan troglodytes) [18], and Sumatran orangutans (Pongo abelii) [18], as well as in other species, including the domestic goat (Capra hircus) [19].".

L76: "A lot of new findings" is vague, please specify the specific advantages of the new assemblies.
Response to comment 14
Following this comment, we added several sentences to clarify the specific advantages of the new assemblies.
"Importantly, it was estimated that 87% of the missing reference exons and incomplete gene models were recovered using the new gorilla assembly [17]. In addition, several novel genes expressed in the brain were identified using the new orangutan assembly, and complete immune genes with longer repetitive structures were identified in the updated goat genome [19].".

L81: Through combined -> by combining
Response to comment 15
Thank you for your kindly review. We did it.

L110: Cutadapter -> Cutadapt
Response to comment 16
Thank you for your kindly review. We did it.

L115ff: The value for Kerror was omitted.
Response to comment 17
Thanks for this comment, we added the value for Kerror.
"Finally, a total number of 109,210,004,556 k-mers, 1,159,024,556 k-mers with sequencing errors were generated and the peak k-mer depth was 34.".

L125: Quier -> Quiver
Response to comment 18
Thank you for your kindly review. We did it.

L130: To the best of my knowledge, PBJelly doesn't know how to deal with phased assemblies. All previous assembly steps (Falcon, Quiver, Pilon, sspace) also do not talk about phasing information. Please clarify how phasing was dealt with or maintained at this point.
Response to comment 19
Thanks for your valuable comment. We agree that PBJelly and previous assembly steps (Falcon, Quiver, Pilon) could not deal with phased assemblies. The term "phased genome assembly" here was used to indicate the genome assembly finished at this period, but not the "phased haplotype-resolved genome assembly". This sentence was confusing here, we now say: "Thus, at the end of the first stage, the genome assembly had a contig N50 of 5.72 Mb and a scaffold N50 of 8.20 Mb (Supplementary Table S3).".

L130: The authors only mention the scaffold N50 after gap-filling. I see the contig N50 is mentioned in the supplementary, but I cannot find the contig N50 of the base assembly before gap-filling anywhere. It would be worth to mention it to understand the relative contributions of additional steps.
Response to comment 20
Thanks for your comment. Following gap-filling with PBjelly software, contig N50 increased to 8.2 Mb from N50 of 7.8 Mb at previous step. We added details to clarify this point.
"Using the initial genome assembly, SSPACE-LongRead v1-1 [33] was implemented for getting a longer scaffold by processing PacBio long reads and the initial genome assembly with the command "perl SSPACE-LongRead.pl -c <contig-sequences> -p <pacbio-reads>." This procedure generated a genome assembly with scaffold N50 of 7.81 Mb (Supplementary Table S2). The remaining gaps in the assembly were closed using the PBjelly module in the PBSuite (version 15.8.24) [34] with default settings. Thus, at the end of the first stage, the genome assembly had a contig N50 of 5.72 Mb

and a scaffold N50 of 8.20 Mb (Supplementary Table S3)."

L136: due -> using
Response to comment 21
Thank you for your kindly review. We did it.

L144: Can the authors comment on the difference between the genome size based on k-mer estimates and the actual assembly size?
Response to comment 22
Thanks for your comment. This difference may be due to the large number of repeat sequences in the genome, which occupied more than 50% of the genome region. Despite the Pacbio reads were used, a lot of repeat sequences were still could not be assembled, for example in the centromeres regions. In addition, we checked the duplicated genes and found only 1.6% duplicated genes compared to 92.4% of complete BUSCO matches. This suggests major duplication did not account for this assembly.

L145: acquired -> assembled
Response to comment 23
Thank you for your kindly review. We did it.

L147ff: It would be great to actually show this, e.g. by checking what the filled gaps contain. What added value does the new assembly have.
Response to comment 24
Thanks for this comment. We made some comparisons between our new assembly and the previous assembly. we aligned our genome against the previous version using MUMMER (v4.0.0beta2) and identified a total of 2,217 insertions in our new assembly. These insertion regions were mainly located in the intergenic and repetitive regions. Further analysis showed that 6,452 gaps in the previous version that were predicted to be filled by >29.7 Mb of sequence in our new assembly. These filled gaps were mainly located in the intergenic and repetitive regions, with a small fraction of the sequence data annotated as gene regions. Also, our new assembly had a higher proportion of repeat sequences (50.82%) as compared to the previous version (46.15%); in particular, the number of LINE (long interspersed elements) transposable elements and tandem repeats was greatly increased (further details are given in the "Identification of repeat elements" section). Thus, the newly assembled genome was substantially more complete and continuous.
We added several sentences to address this comment. See also the response to your valuable comments 1 and 4.

L150: I feel that mapping ratios of Illumina data are not an adequate measure for assembly accuracy, especially given that BWA mem maps all reads very liberaly. I understand the desire to include such a number, a better (albeit not perfect) solution might be to map the Illumina data, perform a standard variant calling and quantify the number of high confidence homozygous alternative variants as a proxy to the assemblies' error rate.
Response to comment 25
Thanks for this comment. We performed a standard variant calling by Samtools, results showed that the number of homozygous SNP was 7690, occupying a proportion of 0.0004% in all SNPs, suggesting a high assembly accuracy rate. We added two sentences and one table (supplementary table S7) to address this comment.
"Genome assembly accuracy was also measured using the standard variant calling method in samtools (http://samtools.sourceforge.net/), with the command "samtools mpileup -q 20 -Q 20 -C 50 -uDEf." We found that the homozygous SNP (single nucleotide polymorphism) s comprised 0.0004% of all SNPs (7,690 of 559,048), suggesting that our genome assembly was highly accurate (Supplementary Table S7).
"

L163: identified -> identify
Response to comment 26
Thank you for your kindly review. We did it.

L163: homolog -> homology
Response to comment 27

	Thank you for your kindly review. We did it.<br><br>L165: I suppose the authors used all of RepBase, not only the TEs within it?<br>Response to comment 28<br>Thanks for this comment. Yes, we used all elements in the RepBase database, but not only the TEs within it. We corrected this sentence as follows.<br>“In the homology approach, we searched the genome for repetitive DNA elements (as listed in the Repbase database v16.02) using RepeatMasker v4.0.6 (http://www.repeatmasker.org/) [29] with the parameters “-a -nolow -no_is -norna -parallel 1” and using RepeatProteinMask (implemented in RepeatMasker).”.<br><br>L168: The authors ran RepeatModeler in addition to RepeatMasker. It would be interesting to know if they detected repeat elements that are absent from RepBase and might be unknown/lineage specific.<br>Response to comment 29<br>We followed this comment and examine the repeat elements detected from RepeatModeler and RepeatMasker respectively. Results showed that several repeat elements including LINE and SINE absent from Repbase database were detected in the de novo approach (Supplementary Table S10). The total length of these repeat elements was 186,195,432bp, accounting for 6.13% of the genome, suggesting that these repeat elements may be specific for R. roxellana.<br><br>L178: Specify what database was used.<br>Response to comment 30<br>We followed this comment and added two sentences to clarify this point.<br>“Using BLASTN with an E-value of 1E-10, we identified four rRNAs in the R. roxellana genome homologous to human rRNAs: 28S, 18S, 5.8S, and 5S (GenBank accession numbers NR_003287.2, NR_003286.2, NR_003285.2, and NR_023363.1, respectively).”<br><br>L208ff: This sentence is very vague. Please be specific about what this comparison is about, and what "other mammals" were included and why.<br>Response to comment 31<br>Thanks for your valuable comment. Here, we want to compare the gene structure information including mRNA length, exon length, intron length and exon number between R. roxellana qinlingensis and other representative mammals. In this sentence, "other mammals" including Homo sapiens, Gorilla gorilla, Macaca mulatta, Rhinopithecus bieti, Rhinopithecus roxellana hubeiensis. We chose these mammals as human and gorilla are the most representative primates with high-quality genome, while Macaca mulatta could represent Cercopithecinae, the sister group of Colobinae consisting the sequencing species Rhinopithecus roxellana qinlingensis. As for R. bieti and R. r. hubeiensis, they were the congeneric species of R. r. qinlingensis, more importantly, the R. r. hubeiensis and R. r. qinlingensis are both the subspecies of Rhinopithecus roxellana.<br>We added several sentences to clarify this point.<br>“We also compared the gene structure, including mRNA length, exon length, intron length, and exon number, among R. roxellana qinlingensis and other representative primates (e.g., H. sapiens, G. gorilla, M. mulatta, R. bieti, and R. r, hubeiensis). We found that genome assembly patterns were similar among R. roxellana qinlingensis and the other primates (Supplementary Fig. S2).”.<br><br>L211: The authors need to specify what they mean by functional annotation, and how this annotation was performed. Assigning a biological function to 22053 seems a bit high.<br>Response to comment 32<br>Thanks for this comment. Functional annotation indicated those predicted genes were annotated with the known protein databases to better understand their biological function. We performed the annotation analysis by annotating the predicted genes to the known protein database (NR, SwissProt and KEGG et al.) with the blastp command, and the best match for each gene was identified with the blast E value of 1E-5. Nearly half (10,670 of 22,497) of these genes were annotated to the predicted proteins in NR database derived from the previous genome annotation for the Rhinopithecus roxellana. And it therefore was reasonable for the assignment of 22,053 genes with biological function. We added several sentences to clarify this point.

Powered by Editorial Manager® and ProduXion Manager® from Aries Systems Corporation

	“To better understand the biological functions of the predicted genes, we used BLASTP (with an E-value of 1E-5) to identify the best match for each predicted gene across several databases, including the NCBI nonredundant protein database (NR v20180129), SwissProt (v20150821) [54], Kyoto Encyclopedia of Genes and Genomes (KEGG v20160503) [55], InterPro v29.0 [56], Pfam v31.0 [57], and GO (Gene Ontology)[58]. In this way, 22,053 predicted genes (98.42%) were functionally annotated (Supplementary Table S14). Nearly half (10,670 of 22,497) of these genes were annotated to the predicted proteins in NR database derived from the previous genome annotation for Rhinopithecus roxellana.”<br><br>L235f: The authors present what looks like a GO-term enrichment analysis, but I can't find any mention as to how this analysis was performed.<br>Response to comment 33<br>Thanks for this comment. It is true that we performed a GO-term enrichment analysis. This analysis was performed towards the significantly expanded gene families in Rhinopithecus roxellana. We added several sentences to address this comment.<br>“ To explore the significantly expanded gene families, we performed a GO-term enrichment analysis with EnrichPipeline32 [66, 67], using the 1,370 genes belonging to the 314 significantly expanded gene families as input, and using all predicted genes as background. We considered GO term significant if adjusted the P-value was <0.05. We found that the significantly expanded gene families were mainly associated with the hemoglobin complex, energy metabolism, and oxygen transport (Supplementary Table S16).”<br><br>L250: I can't find this repository on SRA.<br>Response to comment 34<br>Thanks for this comment. The genome assembly and other supporting data have been submitted to GigaDB database and NCBI successfully. However, we did not release them now as interest competition exist and several research groups are also working on this species. We appreciate the editor and reviewers understand the challenges in this situation and we will make related data available soon once this article is published.
**Additional Information:**	
**Question**	**Response**
Are you submitting this manuscript to a special series or article collection?	No
**Experimental design and statistics**<br><br>Full details of the experimental design and statistical methods used should be given in the Methods section, as detailed in our Minimum Standards Reporting Checklist. Information essential to interpreting the data presented should be made available in the figure legends.<br><br>Have you included all the information requested in your manuscript?	Yes
**Resources**<br><br>A description of all resources used, including antibodies, cell lines, animals	Yes

and software tools, with enough information to allow them to be uniquely identified, should be included in the Methods section. Authors are strongly encouraged to cite Research Resource Identifiers (RRIDs) for antibodies, model organisms and tools, where possible.<br><br>Have you included the information requested as detailed in our Minimum Standards Reporting Checklist?	
**Availability of data and materials**<br><br>All datasets and code on which the conclusions of the paper rely must be either included in your submission or deposited in publicly available repositories (where available and ethically appropriate), referencing such data using a unique identifier in the references and in the "Availability of Data and Materials" section of your manuscript.<br><br>Have you have met the above requirement as detailed in our Minimum Standards Reporting Checklist?	Yes

Powered by Editorial Manager® and ProduXion Manager® from Aries Systems Corporation

Manuscript

Click here to view linked References

Click here to access/download;Manuscript;Manuscript_giga_0505.docx

A high-quality genome assembly offor the endangered golden snub-nosed monkey (*Rhinopithecus roxellana*)

Lu Wang[1,†], Jinwei Wu[1,†], Xiaomei Liu[1], Dandan Di[1], Yuhong Liang[1], Yifei Feng[1], Suyun Zhang[1], Baoguo Li[1,2], Xiao-Guang Qi[1,*]

[1] Shaanxi Key Laboratory for Animal Conservation, College of Life Sciences, Northwest University, Xi'an, 710069, China.

[2] Center for Excellence in Animal Evolution and Genetics, Chinese Academy of Sciences, Kunming, 650223, China.

[*]**Correspondence address.** Xiao-Guang Qi, E-mail: qixg@nwu.edu.cn

[†]These authors contributed equally to this work.

ABSTRACT

Background: -The golden snub-nosed monkey (*Rhinopithecus roxellana*),) is an endangered colobine monkey species endemic to China. This species has several distinctive traits, and it is an ideal model for analysing analyses of the evolutionary development of the social structurestructures due to its unique social organization. Although there has been reported a genome assembly offor the subspecies *R. roxellana hubeiensis*, the is available, this assembly is incomplete and fragmented due to employingbecause it was constructed using short readsread sequencing technology. This drawback may loseThus, information important for the understanding of *R. roxellana*, such as genome structural variation and repeat sequences which are important for understanding this endangered species. , may be absent from the available assembly. Therefore, to have a better understanding of evolutionary history and genetic specific signatures, a high-quality reference genome of the taxon is need. needed.

Findings: To obtain a high-quality chromosomechromosomal assembly offor *R. roxellana qinlingensis*, we combined a total of used five techniques includingdifferent methods: Pacific Bioscience's single-molecule real-time sequencing, Illumina's paired-end sequencing, BioNano optical maps, 10X Genomics link-reads, and high-throughput chromosome conformation capture. The results indicate the assembled genome is about was ~3.04 Gb, with a contig N50 of 5.72 MbpMb and a scaffold N50 of 144.56 Mbp, which have madeMb. This

represented a ~~10~~100-fold improvement ~~compared to past~~over the previously published~~. It is shown that a total of 22497~~ genome. In the new genome, 22,497 protein~~-~~-coding genes were predicted, of which ~~22053~~22,053 were functionally annotated. ~~Moreover, gene~~Gene family analysis ~~shows~~showed that 993 and ~~2745~~2,745 gene families ~~are~~were expanded and contracted ~~in the *R. roxellana* genome~~, respectively~~.~~, in the *R. r. qinlingensis* genome. The reconstructed phylogeny recovered a close relationship between *Rhinopithecus rollexana* and *Macaca mulatta*, and these two species diverged approximately 13.4 MYA.

Conclusion: We ~~present the updated~~ constructed a high-quality genome assembly of ~~*R. roxellana* with~~Qinling golden snub-nosed monkey; this genome had superior continuity and accuracy~~. The assembled genome can~~, which might be ~~used~~useful as reference for future genetic studies ~~of the~~in this species. In addition, the updated genome assembly might improve our understanding of this species~~. Also, the updated genome assembly may contribute to our comprehensive understanding of the species, which is~~ and might be particularly ~~helpful in the~~ relevant to conservation ~~of this endangered species.~~efforts. Furthermore, ~~such genome with superior continuity and accuracy can provide~~this high-quality genome might serve as a new standard reference genome for ~~Colobine~~colobine primates.

Keywords: high-quality; *Rhinopithecus roxellana*; genome assembly; annotation; BioNano optical maps

~~Data Description~~

Background information

~~Snub-nosed monkeys (*Rhinopithecus*) consist of five endangered species narrowly restricted to China and Vietnam [1]. Among those, the golden snub-nosed monkey (*Rhinopithecus roxellana*) is also referred to as the Sichuan snub-nosed monkey, with the northernmost distribution of all Asian colobinae species, found only in three isolated regions (Sichuan and Gansu, Shaanxi and Hubei provinces) in central and northwest China~~ The snub-nosed monkeys (genus *Rhinopithecus*) consist of five endangered species narrowly restricted to China and Vietnam [1]. Of those, the golden snub-nosed monkey (*Rhinopithecus roxellana*), also known as the Sichuan snub-nosed monkey, has the northernmost distribution of all Asian colobine species; this monkey is found only in three isolated regions in central and northwest China (the Sichuan, Gansu, Shaanxi, and Hubei Provinces) [2, 3]. ~~This species~~ The golden snub-nosed monkey is characterized by several distinctive traits, ~~such as~~including golden fur, a blue facial ~~colour,~~color, an odd-shaped nose, ~~more folivorous, most striking~~ and folivory. In addition, the species has a unique multilevel social system ~~with multilevel societies, a rare and~~ ; such complex ~~system that is~~systems are found only in a few ~~mammal species, including human beings [4].~~mammals, including humans [4]. Therefore, ~~*R. roxellana*~~ Qinling golden snub-nosed monkey is an ideal model for ~~analyzing~~ the analysis of social structure evolution in primates

and may also provide ~~chances~~opportunities to investigate evolutionary and socio-anthropological patterns of human society~~ in social anthropology~~.

~~As a research hotspot, studies on *R. roxellana* have investigated various aspects~~ [5-7]~~. Recently, genomic analysis offered a powerful tool and has successfully been employed to underlie the molecular evolution of several groups~~ [8-10]~~. According to the morphological variation and distribution difference, *R. roxellana* can differentiate into three subspecies: *Rhinopithecus roxellana roxellana* from Minshan mountains of Sichuan and Gansu province, *R. r. Qinlingensis* from Qinling mountains of Shaanxi province, *R. r. hubeiensis* from Shennongjia Mountains [3]. Up to now, the best genome assembly of *R. roxellana* was published in 2014~~ Based on morphological variations and discontinuous distributions, *R. roxellana* is distinguished into three subspecies: *R. r. roxellana* from the Minshan Mountain in the Sichuan and Gansu Provinces, *R. r. qinlingensis* from the Qinling Mountain in Shaanxi Province, and *R. r. hubeiensis* from Shennongjia Mountain in Hubei Province [3]. Recent studies of *R. r.* have focused on behavioral dynamics, population history, and social systems [5-7]~~, which was derived from short reads sequencing on Illumina HiSeq 2000 platform. Based on this achievement, studies on its folivorous dietary adaptations and the evolutionary history of *R. roxellana* have been conducted~~. Genomic analyses have helped to untangle the molecular evolution of several groups, including maize (*Zea mays*), bats (*Myotis brandtii*), and killifish (*Nothobranchius furzeri*) [8-10]~~. Despite such progress, the information including structural~~

~~variation and repeat sequences was largely absent or unreliable due to the incomplete and fragmented genome assembly~~ . To date, only a single genome assembly is available for golden snub-nosed monkey. This assembly, published in 2014, was derived from short sequencing reads generated by the Illumina HiSeq 2000 platform [11]~~.~~

~~Owing to the advances in sequencing technology, it is possible to obtain high-quality genome assembly that can provide new insights into the understanding of the organisms. Indeed, many unreported transposable elements and specific genes were identified by using the improved maize reference genome~~ . Several studies have been published based on these data, including analyses of the folivorous dietary adaptations of *R. r.* and its evolutionary history [11-13]. Despite the utility of this previously published data, much relevant information, including structural variations and repeat sequences, is largely absent or unreliable due to the incomplete and fragmented genome assembly ~~. By combining new sequencing approaches, Seo et al.~~ [14, 15].

Owing to advances in sequencing technology, it is now possible to obtain high-quality genome assemblies that can provide new insights in organismal research. Indeed, many previously unreported transposable elements and specific genes in maize were identified using an improved reference genome [16]. By combining new sequencing approaches, Seo et al. [14] discovered clinically relevant structural variants and previously unreported genes in the updated human genome. New sequencing technologies, including Pacific Bioscience’s single-

molecule real-time (SMRT) sequencing, BioNano optical mapping, and Hi-C-based chromatin interaction maps, have been used in several species closely related to humans, including gorillas (*Gorilla gorilla gorilla*) [17], chimpanzees (*Pan troglodytes*) [18], and Sumatran orangutans (*Pongo abelii*) [18], as well as in other species, including the domestic goat (*Capra hircus*) [19]. Importantly, it was estimated that 87% of the missing reference exons and incomplete gene models were recovered using the new gorilla assembly [17]. In addition, several novel genes expressed in the brain were identified using the new orangutan assembly, and complete immune genes with longer repetitive structures were identified in the updated goat genome [19]. However, the *R. r.* genome has not yet been updated using new sequencing approaches, slowing progress towards a better understanding of this endangered species.

Here, we report a greatly improved assembly~~ and annotation~~ of the reference genome for *R.* ~~*roxellana* ~~*r.* ~~from through combined~~generated by a combination of five technologies: ~~Pacific Bioscience's single molecule real-time~~SMRT sequencing ~~(SMRT), Illumina's~~from Pacific Biosciences (PacBio), HiSeq paired-end sequencing from Illumina (HiSeq), BioNano optical maps (BioNano), 10X Genomics link-reads (10X Genomics~~)~~), and high-throughput chromosome conformation capture (Hi-C). ~~Also, this is~~Our results represent the first colobine genome sequenced and assembled with both long reads and short reads. ~~The~~This updated genome assembly may allow us to further investigate *R.* ~~*roxellana*~~*r.*, ~~offering~~providing new opportunities ~~in analyzing~~to analyze evolutionary history and ~~searching those~~to identify

genetic changes associated with the development of specific traits in this species~~, which~~. Such analyses may provide ~~new~~ insights ~~in~~helpful for the conservation of this endangered primate. In addition, this genome~~ with~~, which has superior continuity and accuracy, will ~~provide~~act as a new ~~standard~~ reference genome for colobine primates.

Data Description

Sample collection and sequencing

The animal used for ~~the~~ sequencing was an adult ~~dead~~ male *R.* ~~roxellana~~*r. qinlingensis* ~~in Qinlin Mountains,~~ from Qinling Mountain, who died naturally and the dead body was stored in ultra -cold storage freezer at Louguantai Breeding Centre, Xi'an, Shaanxi ~~province~~Province, China. Total genomic DNA was extracted from the heart tissue. To acquire a high-quality genome assembly, we ~~applied a~~ combined five sequencing methods. Initially, PacBio~~'s~~ SMRT sequencing was ~~conducted~~performed on the SEQUEL platform ~~according to manufactures, after removing adaptors in polymerase reads, resulting a total of~~following the manufacturer's instructions. After quality control, during which subreads shorter than 500 bp were removed, 304.84 Gb clean long reads (95.86X coverage~~). Different from~~) remained. The average/N50 length of the PacBio ~~sequencing~~reads was 16.69 kb. Simultaneously, paired-end sequencing was performed using an Illumina NovaSeq 6000 platform, with an insert size of 350 bp. Then those ~~S~~short reads ~~derived from this step~~ were filtered ~~by SOAPfilter v. 2.2~~ [20] ~~(a~~using the

SOAPdenovo2 software from SOAPdenovo2) with the following criteria: filtering those [20], removing reads with adapters, contaminations, N>10% unknown bases more than 10% and (N), or low quality, which generated . After filtering, 423.32 Gb sequencing clean reads remained (133.12X coverage). In addition, aA high--quality optical genome map was also constructed with the Irys platform (BioNano Genomics), from which we acquired). The average/N50 length of the molecules used for optical mapping was 338 kb. The average BioNano optimal marker density examined was 11.66 per 100 kb, while the average marker density was 12.62 per 100 kb for the predicted map based on the assembled contigs. Thus, the observed BioNano map was consistent with the predicted map. The BioNano map generated 463.75 Gb of large DNA molecules. BesidesNext, 10X genomic linklinked-reads sequencing was carried outperformed on an Illumina Hiseq Xten platform, andgenerating 348.41 Gb clean reads (109.56X coverage) were generated in total.). Finally, aan Hi-C library was prepared and sequenced with an Illumina NovaSeq 6000 platform forto produce a chromosome-scale scaffolding of the genome assembly. Adapter sequences and low--quality reads were discarded by using CutadapterCutadapt v1.0 [21],) [21] with the parameters “-e 0.1 -O 5 -m 100 –n 2 --pair-filter=both,” yielding a total of 310.92 Gb clean data (97.77X coverage). Statistics of the Detailed sequencing data was detailedstatistics are given in **Table 1**.

De novo assembly of the _R. roxellana_ genome

EstimationAn estimation of genome size is helpful towould increase our understanding of *R. roxellana*. GenerallyThus, we estimated the size of the *R. roxellana* genome size of *R. roxellana* with the formula ofas $G = (K_{total} - K_{error})/D$, in whichwhere G representsrepresented genome size, while K_{total}, K_{error} and D indicates represented the total number of k-mers, K_{error} represented the number of k-mers which caused bywith sequencing errors, and D indicated the k-mer depth respectively. Finally, . We generated 109,210,004,556 k-mers were generated, and the, 1,159,024,556 of which had sequencing errors. The peak k-mer depth was 34. Thus, the genome size of *R. roxellana* was estimated to be about 3.18 Gb. The distribution of k-mer frequency was shownfrequencies is given in **Supplementary Fig. S1**.

The *de novo* assembly of newly sequenced *R. roxellana* genome was performed in four progressive steps. Firstly, the assembly was conducted with the FALCON assembler (default parameters) [11] with the long reads obtained from the PacBio platform, which mainly includes three steps: 1) detection of overlap and reads correction; 2) detection of overlap between corrected reads; and 3) construction of string graph. Following FALCON step, the string graph assembly was further polished by Quiver with long reads [22] and then corrected by Pilon with Illumina short reads [23]. Based on this initial genome assembly, sspace-longreads [20] with default settings was implemented for getting a longer scaffold genome by using PacBio long reads. Despite attempts have been made, scaffolding gaps were still found, those gaps were

further closed with the help of PBjelly software under default settings, which generated a Time-phased genome assembly with scaffold N50 of 8.20 Mbp (**Supplementary Table S1**).

Secondly, a hybrid assembly with scaffold N50 of 9.22 Mbp was constructed on the basis of Bionano optical map data using Bionano Solve3.1 (www.bionanogenomics.com) with default parameters (**Supplementary Table S2**). Thirdly, 10X genomic linked reads were employed to connect scaffolds from the second step by fragScaff software [24], which has updated the scaffold N50 of genome assembly to 24.09 Mbp (**Supplementary Table S3**). Subsequently , those short-reads derived from Illumina were applied to correcting errors due to Burrows-Wheeler Aligner (BWA) [25] and pilon-1.18 [23].

Finally, to build chromosome-level assembly scaffolds, we mapped the Hi-C reads to the assembled scaffolds with BWA [25]. Then Hi-C data was subsequently applied to cluster, order, and orient scaffolds by Lachesis software [26]. The chromosome-level scaffolds for *R. roxellana* allowed us to estimate the interaction frequency between chromosome loci, the interaction heatmap shown in **Fig. 2**.

These processes together yielded a updated genome assembly of *R. roxellana* with its genome size of 3.04 Gb, contig N50 of 5.72 Mbp and scaffold N50 of 144.56 Mbp (**Table 2**). In comparison, the newly acquired *R. roxellana* reference genome has 100-fold higher contiguity than its previous (contig N50: 5.72 Mb versus 25.5 kb and scaffold N50: 144.56 Mb versus 1.55 Mb) [11]. We suppose that the remarkable improvement in contiguity can be

attributed to the longer read length, deeper sequencing depth, properly assembled gaps, and increased sophisticated assembly algorithm.

To assess the genome assembly accuracy, we aligned the Illumina short reads to the assembly by BWA program [25]. The mapping rate for the reads was about 99.17%, further investigations showed that those mapped reads covered approximately 99.27% of the assembly (**Supplementary Table S4**). In addition, we estimated the assembly completeness by conducting Benchmarking Universal Single-copy Orthologs (BUSCO) analysis with BUSCO V3.0 [27]. As for the BUSCO analysis, the annotation results were classified as complete BUSCOs, fragmented BUSCOs and missing BUSCOs. The results showed that among the 4,104 mammalian BUSCOs, the complete BUSCOs, the fragmented BUSCOs and the missing BUSCOs occupied a proportions of 94.0%, 2.9% and 3.1% in the genome assembly of *R. roxellana qinlingensis*, respectively (**Supplementary Table S5**). The assembly completeness was also checked by core eukaryotic gene mapping approach (CEGMA) [28]. The results showed that 93.95% (233 of 248) conserved genes were found in our genome assembly (**Supplementary Table S6**). Together, these analyses indicated a high accuracy and completeness of our genome assembly.

Identification of repeat elements

Repeat sequences occupy a large proportion of the genome sequences. Thus, it is necessary for us to identified those repeat elements. In our study, we combined homolog based and *de novo* based approach to predict and classify repeat elements. As for the homolog approach, we searched transposable elements from the RepBase database [29] with RepeatMasker v4.0.6 (http://www.repeatmasker.org/) and RepeatProteinMask (implemented in RepeatMasker). The *de novo* method was employed with RepeatModeler V1.0.11 [30], RepeatMasker v4.0.6 and Tandem Repeat Finder (TRF) (Version 4.07b) [31]. We merged the findings from both methods. Results showed that 45.43% of the genome was predicted as repeat elements (**Supplementary Table S7**). A closer investigation indicated that the largest category of repeat elements in the species is the short (SINEs) and long (LINEs) interspersed nuclear elements. The detailed categories of repeat elements are summarized in **Supplementary Table S8**.

The *de novo* assembly of the newly sequenced *R. roxellana* genome was performed in four progressive stages. First, long reads obtained from the PacBio platform were assembled as follows: detection of overlap and read correction, detection of overlap between pairs of corrected reads, and string graph construction. Assembly of the PacBio long reads was performed using FALCON (version 0.4.0) [32] with the parameter set “length_cutoff = 5000, length_cutoff_pr = 5000, pa_HPCdaligner_option = -v -B128 -e.70 -k14 -h128 -l2000 -w8 -T8 -s700, ovlp_HPCdaligner_option = -v -B128 -e.96 -k16 -h480 -l1500 -w8 -T16 -s700” . Next,

the assembled PacBio contigs was polished using Quiver (SMRTLink version 5.1.0) with PacBio long reads [22], and also the contig assembly was corrected by Pilon-1.18 (java -Xmx500G -jar pilon-1.18.jar --diploid --threads 30) with Illumina short reads [23]. The contig N50 of the initial assembly was 4.74 Mb (**Supplementary Table S1**). Using the initial genome assembly, SSPACE-LongRead v1-1 [33] was implemented for getting a longer scaffold by processing PacBio long reads and the initial genome assembly with the command “perl SSPACE-LongRead.pl -c <contig-sequences> -p <pacbio-reads>.” This procedure generated a genome assembly with scaffold N50 of 7.81 Mb (**Supplementary Table S2**). The remaining gaps in the assembly were closed using the PBjelly module in the PBSuite (version 15.8.24) [34] with default settings. Thus, at the end of the first stage, the genome assembly had a contig N50 of 5.72 Mb and a scaffold N50 of 8.20 Mb (**Supplementary Table S3**).

In the second stage, the BioNano molecules were filtered, requiring a minimum length of 150 kb and minimum of nine labels per molecule. Then, a genome map was assembled *de novo* with IrysView (version 2.3; BioNano Genomics), based on the optically mapped molecules. The assembled PacBio scaffolds were input into hybridScaffold [35]. In brief, the hybrid scaffolding process included the alignment of the PacBio scaffolds against the BioNano genome maps, followed by the identification and resolution of conflicting alignments. At the end of stage two, the hybrid genome assembly had a scaffold N50 of 9.22 Mb (**Supplementary Table S4**).

In the third stage, the 10X genomic linked reads were connected with the scaffolds generated in stage two to construct super-scaffolds. In brief, we used the long ranger basic pipeline (https://support.10xgenomics.com/genome-exome/software/downloads/) to handle the basic read in and barcode processing of the 10X genomic linked reads. The processed 10X linked reads were then mapped to the hybrid genome assembly from stage two with bowtie2 [36], using the command "bowtie2 genome.fa -1 reads1.fq.gz -2 reads2.fq.gz -p 12 -D 1 -R 1 -N 0 -L 28 -i S,0,2.50 --n-ceil L,0,0.02 --rdg 5,10 --rfg 5,10).". We also used a self-against-self (genome.fa-against-genome.fa) blastn to generate two bed files, and merged these files using fragScaff (version 140324.1) [24], with the parameters "-fs1 '-m 3000 -q 20 -E 30000 -o 60000', -fs2 '-C 2', -fs3 '-j 1.5 -u 2'.". These procedures generated an updated genome assembly with a scaffold N50 of 24.09 Mb (**Supplementary Table S5**). Subsequently, we corrected errors in the assembly, based on the Illumina short reads, using the Burrows-Wheeler Aligner (BWA) [25] and Pilon-1.18 [23].

In the fourth stage, the Hi-C data were used to build chromosome-level assembly scaffolds. In brief, Hi-C sequencing data were first aligned to the assembled genome using BWA [25]. Scaffolds were then clustered, ordered, and oriented using Lachesis [26], with the parameter set "CLUSTER_MIN_RE_SITES = 1800, CLUSTER_MAX_LINK_DENSITY = 4, and CLUSTER_NONINFORMATIVE_RATIO = 0." This procedure generated 22 accurately clustered and ordered pseudo-chromosomes, with a genome size of 3.04 Gb, a contig N50 of

5.72 Mb, and a scaffold N50 of 144.56 Mb (**Table 2**). The pseudo-chromosomes were divided into 100-kb bins and the interaction frequencies between pairs of 100-kb genomic regions were determined (**Fig. 2**).

Assessment of the genome newly assembled

We evaluated our newly assembled *R. roxellana* genome against the previously published assembly. The contiguity of our *R. roxellana* genome was 100-fold greater (contig N50: 5.72 Mb; scaffold N50: 144.56) than the previous version (contig N50: 25.5 kb; scaffold N50: 1.55 Mb) [11]. We also aligned our genome against the previous version using MUMMER (v4.0.0beta2) [37] and identified 6,452 gaps in the previous version that were predicted to be filled by >29.7 Mb of sequence in our new assembly. These filled gaps were mainly located in the intergenic and repetitive regions, with a small fraction of the sequence data annotated as gene regions. Our new assembly also had a higher proportion of repeat sequences (50.82%) as compared to the previous version (46.15%); in particular, the number of LINE (long interspersed elements) transposable elements and tandem repeats was greatly increased (further details are given below, in the "Identification of repeat elements" section). Thus, the newly assembled genome was substantially more complete and continuous. It was likely that the remarkable improvement in contiguity was due to the increased read length, deeper sequencing depth, improved gap assembly, and more sophisticated assembly algorithm.

To assess the accuracy of our genome assembly, we aligned the Illumina short reads to the assembly using BWA [25], with the parameters "-o 1 -i 15". Approximately 99.17% of the short reads were mapped to the genome assembly. Further investigations indicated that these reads covered approximately 99.27% of the total assembly (**Supplementary Table S6**). Genome assembly accuracy was also measured using the standard variant calling method in samtools (http://samtools.sourceforge.net/), with the command "samtools mpileup -q 20 -Q 20 -C 50 -uDEf." We found that the homozygous SNP (single nucleotide polymorphism) s comprised 0.0004% of all SNPs (7,690 of 559,048), suggesting that our genome assembly was highly accurate (**Supplementary Table S7**). In addition, we estimated assembly completeness using Benchmarking Universal Single-copy Orthologs (BUSCO) v3.0.2 [27], with the parameters "-i -o -l -m genome -f -t." based on mammalia_odb9 (creation date: 2016-02-13; number of species: 50; number of BUSCOs: 4,104). BUSCO analysis identified 4,104 mammalian BUSCOs in the newly assembled *R. roxellana* genome: 94.0% complete BUSCOs, 2.9% fragmented BUSCOs, and 3.1% missing BUSCOs (**Supplementary Table S8**). Assembly completeness was measured using the core eukaryotic gene (CEG)-mapping approach (CEGMA v2.5) [28]. Of the 248 CEGs known from six model species, 93.95% (233 of 248) were identified in our new genome assembly. Of these, 220 CEGs were complete and unfragmented, and the remaining 13 were complete but fragmented (**Supplementary Table**

S9). Together, these analyses indicated that our new genome assembly was highly accurate and complete.

Identification of repeat elements

Repeat sequences account for a large proportion of the total genome is thus important to identify repeat elements. Here, we predicted and classified repeat elements both based on homology and *de novo*. In the homology approach, we searched the genome for repetitive DNA elements (as listed in the Repbase database v16.02) using RepeatMasker v4.0.6 (http://www.repeatmasker.org/) [29] with the parameters "-a -nolow -no_is -norna -parallel 1" and using RepeatProteinMask (implemented in RepeatMasker). To identify repetitive elements *de novo*, we used RepeatModeler v1.0.11 [30], with the parameters "-database genome -engine ncbi -pa 15)." Tandem repeats in the genome were detected using Tandem Repeat Finder (TRF) v4.07b [31], with parameters "2 7 7 80 10 50 2000 -d -h"). We merged the results of the two methods. In total, the new genome assembly comprised 50.81% repetitive sequences (**Supplementary Table S10)**. Closer investigation indicated that the largest categories of repeat elements in the *R. roxellana* genome were the short and long interspersed nuclear elements (SINEs and LINEs, respectively). In addition, several repeat elements absent from Repbase database were detected in the *de novo* approach (**Supplementary Table S10**). The total length of these repeat elements was 186,195,432bp, accounting for 6.13% of the genome,

suggesting that these repeat elements may be specific for *R. roxellana*. Compared with the repeat sequences in the previous assembly, our genome included relatively more LINE transposable elements (28.23% vs. 6.21%) and tandem repeats (6.20% vs. 2.82%). The detailed categories of repeat elements are summarized in **Supplementary Table S11**.

Copy number variation (CNV)

We also performed a CNV analysis. In brief, we first mapped the Illumina short reads to the assembled genome using BWA with default parameters. Then, the sorted mapping bam file was used as input for CNVnator v0.3.3 [38], with the parameters "-unique -his 100 -stat 100 -call 100.". The obtained CNVs were filtered, retaining only those where q0 was <0.5 and e-val1 was <0.05. After filtering, 676 CNVs remained, with a total length of 9,198,900 bp **(Supplementary Table S12)**.

Non-coding RNA prediction

Non-coding RNA consists of several RNAs, as such ribosomal RNA (rRNA), transfer RNA (tRNA), microRNAs (miRNA) and small nuclear RNA (snRNA). This RNA group mainly plays a regulation role in biological processes. In our study, we detected rRNA from a Human rRNA database with BLASTN command, and the E-value was set as 1E-10. Similarly, miRNAs and snRNAs were searched against the Rfam database [39] with INFERNAL 1.1rc4 [40]. The tRNAs were predicted by tRNAscan-SE 1.3.1 software [41]. The numbers of rRNA,

miRNA, snRNA and tRNA were 608, 17,813, 3,656 and 460, respectively in the genome of the species (**Supplementary Table S9**).

Non-coding RNAs included ribosomal RNAs (rRNAs), transfer RNAs (tRNAs), microRNAs (miRNAs), and small nuclear RNAs (snRNAs). Non-coding RNAs primarily regulate biological processes. Using BLASTN with an E-value of 1E-10, we identified four rRNAs in the *R. roxellana* genome homologous to human rRNAs: 28S, 18S, 5.8S, and 5S (GenBank accession numbers NR_003287.2, NR_003286.2, NR_003285.2, and NR_023363.1, respectively). We also searched for miRNAs and snRNAs in the new genome using INFERNAL v1.1rc4 [40] against the Rfam database release 13.0 [39]. The tRNAs were predicted by tRNAscan-SE 1.3.1 [41]. We identified 608 rRNAs, 17,813 miRNAs, 3,656 snRNAs, and 460 tRNAs in the *R. roxellana* genome (**Supplementary Table S13**).

Gene prediction and functional annotation

We combined prediction methods based on *de novo*, homolog prediction and transcriptome data to estimate genes. As for *ab initio* based prediction, a total of five programs, namely Augustus v. 3.2.2 [42], GlimmeHMM v. 3.0.1 [43], GENSCAN [44], GENEID [45] and SNAP V2013-11-29 [46] were employed to predict protein-coding genes. Subsequently, we used the homolog-based prediction approach. Protein sequences from five homolog species (*Homo sapiens*, *Gorilla gorilla*, *Macaca mulatta*, *Rhinopithecus bieti*, *Rhinopithecus roxellana*

~~*hubeiensis*) were downloaded from Ensemble Release 75 (http://www.ensembl.org/info/data/ftp/index.html), and used to perform TBLASTN blast against the repeat-masked genome sequences~~ [47]~~. The related homologous genome sequences were then annotated to the matching proteins by GeneWise 2.4.1~~ [48]~~. Finally, we estimated genes based on transcriptome data. During this process, high-quality RNAs from heart and skin tissue were sequenced by an Illumina Novaseq 6000 platform. RNA-seq reads were assembled with trinityrnaseq-2.1.1~~ We predicted genes using a combination of approaches: *de novo*, homology prediction, and transcriptome. For *ab initio* predictions of protein-coding genes, we used Augustus v3.2.2 [42], with parameters "--uniqueGeneId = true –noInFrameStop = true --gff3 = on –genemodel = complete –strand = both"; GlimmeHMM v3.0.1 [43], with parameters "-g -f"; GENSCAN [44], GENEID [45], and SNAP v2013-11-29 [46].

Next, we predicted genes using homology-based approach. Protein sequences from five homologous species (*Homo sapiens*, *Gorilla gorilla*, *Macaca mulatta*, *Rhinopithecus bieti*, and *Rhinopithecus roxellana hubeiensis*) were downloaded from Ensemble Release 75 (http://www.ensembl.org/info/data/ftp/index.html). We compared these sequences to the repeat-masked *R. roxellana* genome using TBLASTN (-p tblastn -e 1e-05 -F T -m 8 -d) against the repeat-masked genome sequences [47], with parameters "-p tblastn -e 1e-05 -F T -m 8 -d." The identified homologous genome sequences were annotated using GeneWise (Version 2.4.1) [48], with the parameters "-tfor -genesf -gff."

Finally, we estimated genes based on transcriptome data. High-quality RNAs from the heart and skin tissue of the *R. roxellana qinlingensis* specimen were sequenced on an Illumina Novaseq 6000 platform. RNA-seq reads were assembled using trinityrnaseq-2.1.1 [49]~~. The~~, with the parameters "--seqType fq --CPU 20 --max_memory 200G --normalize_reads --full_cleanup --min_glue 2 --min_kmer_cov 2 --KMER_SIZE 25." To identify validate transcripts, the assembled transcript sequences were aligned to the *R. roxellana* genome ~~by~~using Assemble Spliced Alignment (PASA) [50]~~ with default parameters. In addition, we estimated the expression levels of transcripts by Tophat 2.0.13~~ [51]~~ and Cufflinks~~ [52]~~.~~

~~The genes predicted from those three approaches were merged with EVidenceModeler~~ [53]~~. Furthermore, untranslated regions and alternative splicing of those predicted gene sets were further checked by PASA with the help of transcriptome data~~ , with default parameters. We estimated transcript expression levels using Tophat 2.0.13 [51] (with the parameters "-p 6 --max-intron-length 500000 -m 2 --library-type fr-unstranded") and Cufflinks [52].

The genes predicted by each of the three approaches were merged using EVidenceModeler [53] with the parameters "--segmentSize 200000 --overlapSize 20000." We weighted transcript predictions most highly, followed by homology-based predictions and *ab initio* predictions. Untranslated regions and alternative splicing of the predicted gene were explored using PASA, in conjunction with the transcriptome data [50]. ~~Finally, a~~In total~~ of~~ ~~22497~~, 22,497 genes were predicted ~~for~~in the ~~assembly genome of~~ *R. roxellana* genome (**Table**

3), ~~and~~ each ~~of them consisted~~containing an average of 7.71 exons~~ on average~~. The detailed results ~~generated during~~ of the gene prediction process ~~were shown~~are given in **Table 3**~~. And, and~~ **~~Fig. 3~~**~~.~~

We also compared the gene ~~prediction evidence based on different methods were shown in~~ **~~Fig. 3~~**~~. In addition, we made a comparison between the~~ structure, including mRNA length, exon length, intron length, and exon number, among *R. roxellana* ~~*qinlingensis*~~ and other ~~mammals, suggesting a comparable pattern of the~~representative primates (e.g., *Homo sapiens, Gorilla gorilla, Macaca mulatta, Rhinopithecus bieti*, and *Rhinopithecus roxellana hubeiensis*). We found that genome assembly ~~for~~patterns were similar among *R. roxellana* ~~*qinlingensis*~~and the other primates **(Supplementary Fig. S2)**.

~~To have a better understanding the biological functions of those predicted genes, they were annotated with several databases including NCBI nonredundant protein database (NR), SwissProt~~ [54]~~, Kyoto Encyclopedia of Genes and Genomes (KEGG)~~ [55]~~, InterPro~~ To better understand the biological functions of the predicted genes, we used BLASTP (with an E-value of 1E-5) to identify the best match for each predicted gene across several databases, including the NCBI nonredundant protein database (NR v20180129), SwissProt (v20150821) [54], Kyoto Encyclopedia of Genes and Genomes (KEGG v20160503) [55], InterPro v29.0 [56]~~, Pfam~~ [57] ~~and GO database~~ [58]~~. In total, 22053 genes (98.42%) were functionally annotated (~~**~~Supplementary Table S10~~**~~).~~ , Pfam v31.0 [57], and GO (Gene Ontology)[58]. In this way,

22,053 predicted genes (98.42%) were functionally annotated (**Supplementary Table S14**). Nearly half (10,670 of 22,497) of these genes were annotated to the predicted proteins in NR database derived from the previous genome annotation for *Rhinopithecus roxellana*.

In addition, we estimated the genome assembly completeness using transcriptome data. The transcripts were derived from the *de novo* assembly with trinityrnaseq-2.1.1 mentioned above. Those transcripts were clustered into unigenes with the help of using TGICL (TIGR gene indices clustering program, v2.1) [59] with 95% identity similarity cut-off. The generated unigenes were aligned to our assembly version and previous version using BLAT v. 36. Results showed that the completeness degree (percentage of unigenes aligned to a single scaffold in genome) was higher in our assembly (95.35%) compared with that in previous assembly (89.28%) for unigenes larger than 1000 bp (**Supplementary Table S15**), demonstrating the contiguity of our new assembly.

Phylogenetic ~~relationship~~ analysis and gene family estimation

~~Coding~~The coding regions and protein sequences of 11 representative mammals were downloaded from ~~Ensemble (Ensemble~~Ensembl (Ensembl Release 75). ~~The longest transcript was chosen if~~For genes ~~possess many~~with multiple transcript isoforms~~.~~, the longest was chosen. Treefam [60] ~~approach was adopted to estimate gene families. Following all-to-all blast, a total of 17,560 gene families were identified. We reconstructed the phylogenetic relationship between *R. roxellana* and other mammals based on four-fold degenerate sites extracted from~~

the 5,418 single-copy gene families. Phyml (version 3.2) [61] was employed to construct a maximum-likelihood tree under the GTR + gamma model that was inferred from JMODELTEST (version 2.1.10) [62]. Furthermore, we estimated the divergence time with MCMCTREE in PAML [63]. MCMCTREE was performed on the basis of bayesian method and the fossil calibration times from timetree were used as input. Generally, the following calibration times were used: *Homo sapiens* VS *Callithrix jacchus* (40.6-45.7MYA); *Homo sapiens* VS *Pan troglodytes* (6.2~7MYA); *Homo sapiens* VS *Mus musculus* (85-94MYA) and *Homo sapiens* VS *Tarsius syrichta* (71~77MYA). The reconstructed phylogeny confirmed the close relationship between *R. rollexana* and *M. mulatta*. Moreover, we estimated that *R. rollexana* and *M. mulatta* diverged approximately 13.4 million years ago (Mya) (**Fig. 4**).

To have a better understanding the evolutionary history of *R. roxellana*, we estimated the expansion and contraction of gene family in *R. roxellana* by using CAFE 3.0 [64]. A gene family with *p*-value less than 0.05 was considered for further analysis. As a result, 993 and 2,745 gene families were expanded and contracted in *R. roxellana* genome, respectively (**Fig. 4**). Its genome showed substantial expansion of gene families which are mainly related to hemoglobin complex, energy metabolisms and oxygen transport (**Supplementary Table S11**).

- was used to estimate gene families. Using an all-to-all blast, we identified 17,560 gene families. We reconstructed the phylogenetic relationships among *R. roxellana* and other mammals based on four-fold degenerate sites extracted from the 5,418 single-copy gene

families. Phyml v3.2 [61] was used to construct a maximum-likelihood tree using the GTR + gamma model, as inferred by JMODELTEST v2.1.10 [62]. We estimated divergence times with MCMCTREE in PAML v4.8 [63], using the Bayesian method and the fossil calibration times from timetree (http://www.timetree.org/) [65]. The following fossil calibrations were used: *H. sapiens* vs. *Callithrix jacchus* (40.6–45.7 MYA, million years ago); *Homo sapiens* vs. *Pan troglodytes* (~6.2–7 MYA); *Homo sapiens* vs. *Mus musculus* (85–94 MYA); and *Homo sapiens* vs. *Tarsius syrichta* (~71–77 MYA). The reconstructed phylogeny recovered a close relationship between *R. rollexana* and *M. mulatta*. We estimated that *R. rollexana* and *M. mulatta* diverged approximately 13.4 MYA (**Fig. 4**).

To investigate the evolutionary history of *R. r.*, we estimated the expansion and contraction of gene family in this species with CAFE 3.0 [64]. A random birth and death model was used to study gene family variations along each lineage in the phylogenetic tree. This analysis identified 993 expanded gene families and 2,745 contracted gene families in the *R. roxellana* genome (**Fig. 4**). To determine the significance of each gene family, *P*-values in each lineage were estimated by comparing conditional likelihoods derived from a probabilistic graphical model (PGM). All gene family with *P*-values < 0.05 were further analyzed. To explore the significantly expanded gene families, we performed a GO-term enrichment analysis with EnrichPipeline32 [66, 67], using the 1,370 genes belonging to the 314 significantly expanded gene families as input, and using all predicted genes as background. We considered

GO term significant if adjusted the *P*-value was <0.05. We found that the significantly expanded gene families were mainly associated with the hemoglobin complex, energy metabolism, and oxygen transport (**Supplementary Table S16**).

Conclusion

In this study, we generated a high-quality genome assembly ~~of~~for the golden snub-nosed monkey (*R. roxellana*) ~~by~~ using a combination of five advanced technologies. ~~This~~Our results will ~~be helpful to investigate~~inform studies of the origin and evolutionary history of the snub-nosed monkey. In addition, ~~the~~this genome may ~~lay~~provide a ~~foundation~~framework within which to survey the mechanisms ~~about~~underlying the formation of the distinct morphological and sociological characters ~~and understand the unique multilevel societies in *R.*~~of *R. roxellana*. ~~Also, such~~This genome may ~~provide~~ also stimulate new insights ~~for amending~~into the ~~conservation~~improvement of strategies to conserve and ~~management of~~manage this endangered species. ~~Furthermore~~Finally, this genome ~~with~~, which has superior continuity and accuracy ~~can provide~~, may serve as a new standard reference genome for colobine primates.

Declarations

Availability of supporting data

The raw data discussed in this publication have been deposited in NCBI's short read archive under the accession number PRJNA524949. Supporting data are available in the GigaDB database.

Competing interests

The authors declare that they have no competing interests.

Funding

This work was financially supported by Strategic Priority Research Program of the Chinese Academy of Sciences (XDB31020302), the National Natural Science Foundation of China (31622053), the Promotional project for Innovation team, the Department of Science and Technology of Shaanxi Prov. China (2018TD-017), and the National Key Programme of Research and Development, the Ministry of Science and Technology of China (2016YFC0503200).

Abbreviations

Gb: gigabase; kb: kilobase; Mb: megabase; PE: paired-end; PacBio: Pacfic Biosciences; SMRT: single molecule real-time sequencing; Hi-C: high-throughput chromosome conformation capture; BUSCO: Benchmarking Universal Single-copy Orthologs; GEGMA: core eukaryotic gene-mapping approach; GO: gene ontology; TFS: transposable element;

TRF: Tandem Repeat Finder; SINEs: Short interspersed nuclear elements; LINEs: long interspersed nuclear elements; PASA: genome by Assemble Spliced Alignment; NR: NCBI nonredundant protein database; KEGG: Kyoto Encyclopedia of Genes and Genomes. Mya: million years ago.

Author contributions

X.G.Q. conceived and designed the project, L.W., J.W.W. contributed to the work on genomic sequencing and performing data analyses. B.G.L. helped with sample collection. L.W., J.W. W. and X.G.Q. wrote the manuscript. All authors provided input for the paper and approved the final version.

Acknowledgements

We thank Mr. Yiliang Xu, Mr. Qiqi Liang, Mrs. Yue Xie from Novogene for their technical support. Mr. Xuanmin Guang and Mr. Chi Zhang from BGI for their assistance in data analysis. We thank to Mr. Ruliang Pan for his gracious help polishing the language. We are also grateful to Mr. Yinghu Lei from Louguantai Breeding Center, Dr. Zhipang Huang, and Dr. Pei Zhang from Northwest University for their helping with the sampling collection. We specially appreciate Prof. Zhengbing Wang, and Prof. Jiang Chang from Discipline Development Department of Northwest University for their support. This study was fundamentally supported by Discipline Construction Project of Northwest University.

Figures and tables

Figure legends:

Fig. 1. ~~**The photo**~~**Image of *R. roxellana*, taken in the Qinling** ~~**mountains.**~~ **Mountain, China.**

Fig. 2. Hi-C heatmap of interactions between ~~**chromosome**~~**pairs of chromosomal loci throughout the genome.** Hi~~-~~-C ~~interactome~~interactions within and among ~~chromosomes of~~ *R. roxellana* chromosomes (Chr1–Chr22~~).~~); interactions were drawn based on the chromatin interaction frequencies between pairs of 100-kb genomic regions (as determined by Hi-C). In principle, darker red cells indicate stronger and more frequent interactions, which in turn imply that the two sequences are spatially close.

~~**Fig. 3. The gene prediction evidence** based on **different methods. (**a). Number of the~~

Fig. 3. Gene predictions. (a) Number of genes estimated by ~~the~~various prediction approaches ~~based on~~: *de novo* (blue~~ color), homolog prediction~~), homologys (pink~~ color~~)), and RNA~~-~~-seq data (green~~ color~~). The labels rna_0.5, denove_0.5, and homology_0.5 ~~indicates those~~indicate the genes predicted by each method with an overlap ~~are larger than~~ >50~~% in each method;~~%. (b) Number of ~~the genes shown in combination with the prediction~~genes predicted based on *de novo*, homology, and RNA-seq approaches~~ detailed~~, in ~~fig 2a and the~~addition to expression level ~~standard (~~(in rpkm). The labels rna_0.5, ~~denovo~~denove_0.5, and homology_0.5 ~~indicates~~

that those indicate the genes predicted by each method with an overlap are larger than >50% in each method,%, while rpkm>1 indicates those genes with ana relative expression level larger than >1.

Fig. 4. The*R. roxellana* phylogenetic relationships of *R. roxellana* and other mammals and Gene family analysis in *R. roxellana* genome. and gene families. Phylogenetic relationship wasrelationships were inferred from 54185,418 single-copy gene families. in *R. roxellana* and other mammals. All nodes received 100%had support values. The estimated of 100%. Estimated divergence times are indicatedgiven near the nodes. The images in the figure are credited as “Illustrations copyright 2013 Stephen D. Nash / IUCN SSC Primate Specialist Group. Used with permission”. MYA: million years ago.each node. Numbers under each species indicate the number of gene families that have been expanded (green) and contracted (light yellow) since the split of species from the most recent common ancestor (MRCA). The numbers on each branch correspond to the numbers of gene families that have been expanded (red) and contracted (green) in the mammalian genome. MRCA: most recent common ancestor. Those monkey images are copyright 2013 Stephen D. Nash of the IUCN SSC Primate Specialist Group and are used with permission. MYA: million years ago.

Table 1. Reads generated fromby the five different sequencing methods.

PairPaired-end libraries	Insert size (bp)	Total clean data (Gb)	Read length (bp)	Sequence coverage (X)
Illumina reads	350	423.32	150	133.12
Pacbio reads	20 k	304.84	\n/a	95.86
10X Genomics	500-–700	348.41	150	109.56
BionanoBioNano	\n/a	463.75	\n/a	\n/a
Hi-C	350	310.92	\n/a	97.77
Total	\n/a	1,851.24	\n/a	582.15

Note: The sequence coverage was calculated withbased on an estimated genome size of 3.18 Gb. The sign of backslash indicates that the insert size was absent. n/a: not applicable.

Table 2. ~~**The**~~ **Summary of the final *R. roxellana* genome assembly**~~***statistics of R. roxellana***~~**.**

**Category**	**Contig**		**Scaffold**	
	~~length~~Length (bp)	~~number~~Number	Length (bp)	~~number~~Number
Total	3,038,184,325	6,099	3,038,467,325	3,269
Max	30,757,641	~~\~~n/a	206,558,726	~~\~~n/a
~~>=~~≥2000 bp	~~\~~n/a	5,708	~~\~~n/a	2,879
N50	5,723,610	151	144,559,847	9
N60	4,241,389	211	141,075,955	11
N70	3,173,235	292	135,203,321	14
N80	2,063,823	408	118,350,466	16
N90	896,517	622	83,045,532	19

Note: The "Number" column represents the number ~~indicated those~~ of contigs/scaffolds ~~larger~~longer than the ~~length~~value of ~~its~~the corresponding category. ~~The sign of backslash indicates that the length/number was absent.~~ n/a: not applicable.

Table 3. Summary ~~of~~and characteristics of the predicted protein-coding genes~~ and their characteristics~~.

Gene set		Number	Average transcript length (bp)	Average CDS length (bp)	Average ~~intron~~exon length (bp)	Average intron ~~exon~~ length (bp)	Average exons per gene
De novo	Augustus	32,928	23,441	1,052	196	5,112	5.38
	GlimmerHMM	618,957	4,204	404	166	2,654	2.43
	SNAP	97,298	49,851	755	144	1,1597	5.23
	Geneid	36,863	35,242	1,035	188	7,615	5.49
	Genscan	50,419	40,635	1,137	167	6,800	6.81
Homology	Ggo	25,281	19,893	1,055	184	3,971	5.74
	Hsa	38,444	14,763	826	182	3,942	4.54
	Mmu	21,959	29,709	1,470	187	4,123	7.85
	Rbi	25,320	25,685	1,387	196	3,991	7.09
	Rro	24,121	28,439	1,420	185	4,043	7.68
RNASeq	PASA	66,620	28,449	1,219	164	4,247	7.41
	Cufflinks	73,199	31,497	2,737	409	5,052	6.69
EVM		30,102	22,298	1,098	182	4,199	6.05
Pasa-update*		29,403	27,638	1,180	181	4,782	6.53
Final set*		22,497	34,153	1,369	178	~~4885~~4,885	7.71

Note: Pasa-update* ~~indicates~~includes only the ~~UTRs（Untranslated~~untranslated regions~~） were considered during the filter process, and~~; other regions were not included. Final set* ~~indicates~~represents the results ~~were acquired following~~after the Pasa~~-update~~ filtering process, ~~with the criteria of~~where the longest isoform was chosen if ~~there were~~the case of multiple splicing isoforms~~, and the~~; redundant single exons were also discarded. ~~Number indicates~~The "Number" column gives the ~~specific values~~number of protein-coding genes predicted by each method.

Formatted: None, Line spacing: Double

Supplementary files:

Supplementary Fig. S1. Genome size estimation using the k-mer method.

Supplementary Fig. S2. ~~The comparison~~Comparisons of each element ~~in the genome~~among genomes of homologous species.

Supplementary Table S1. The ~~results of~~contig assembly ~~with~~based on PacBio ~~long reads and gap filling~~ subreads.

Supplementary Table S2. The ~~results of~~scaffold assembly ~~with Bionano optical map data~~based on sspace-longreads results.

Supplementary Table S3. The ~~results of~~ assembly ~~with 10X Genomics link reads~~ after gap-filling.

Supplementary Table S4. The ~~mapping rate of reads and coverage of assembled genome with BWA~~assembly based on BioNano optical map data.

Supplementary Table S5. ~~Assessment results by using BUSCO annotation~~The assembly based on 10X Genomics linked reads.

Supplementary Table S6. The ~~completeness test results~~read mapping rate and the coverage of the assembled genome determined with ~~CEGMA software~~BWA.

Supplementary Table S7. ~~Results of repeats elements predictions from~~The SNPs identified in the genome ~~assembly~~of *R. roxellana***.**

Supplementary Table S8. ~~The results of TEs elements predicted from the genome assembly~~Genome assessment based on BUSCO annotations.

Supplementary Table S9. ~~Summary of predicted RNAs and their characteristics~~Genome assessment based on CEGMA annotations.

Supplementary Table S10. ~~The functional annotation~~Prediction of repeat elements prediction in the ~~genes predicted from *R. roxellana*~~ genome assembly.

Supplementary Table S11. ~~The GO annotation results~~Prediction of ~~expansion gene families~~repetitive sequences in the genome assembly.

Supplementary Table S12. The CNVs identified in the genome assembly.

Supplementary Table S13. Summary and characteristics of the predicted RNAs.

Supplementary Table S14. The functional annotations of the genes predicted in the *R. roxellana* genome.

Supplementary Table S15. Assessment of the new genome assembly using unigenes.

sequences

Supplementary Table S16. The GO annotations of the expanded gene families in the *R. roxellana* genome (adjusted *P*-value < 0.05)

References

1. Li BG, Pan RL, Oxnard CE. Extinction of snub-nosed monkeys in China during the past 400 years. Int J Primatol, 2002; **23**(6):1227-1244.
2. Luo MF, Liu ZJ, Pan HJ, Zhao L, Li M. Historical geographic dispersal of the golden snub-nosed monkey (*Rhinopithecus roxellana*) and the influence of climatic oscillations. Am J Primatol, 2012; **74**(2):91-101.
3. Fang G, Li M, Liu X-J, Guo W-J, Jiang Y-T, Huang Z-P, Tang S-Y, Li D-Y, Yu J, Jin T *et al*. Preliminary report on Sichuan golden snub-nosed monkeys (*Rhinopithecus roxellana roxellana*) at Laohegou Nature Reserve, Sichuan, China. Sci Rep, 2018; **8**(1):16183.
4. Grueter CC, Qi X-G, Li B, Li M. Multilevel societies. Curr Biol, 2017; **27**(18):R984-R986.
5. Qi X-G, Li BG, Garber PA, Ji WH, Watanabe K. Social dynamics of the golden snub-nosed monkey (*Rhinopithecus roxellana*): female transfer and one-male unit succession. Am J Primatol, 2009; **71**(8):670-679.
6. Li H, Meng S-J, Men Z-M, Fu Y-X, Zhang Y-P. Genetic diversity and population history of golden monkeys (*Rhinopithecus roxellana*). Genetics, 2003; **164**(1):269-275.
7. Qi X-G, Garber PA, Ji W, Huang Z-P, Huang K, Zhang P, Guo S-T, Wang X-W, He G, Zhang P *et al*. Satellite telemetry and social modeling offer new insights into the origin of primate multilevel societies. Nat Commun, 2014; **5**:5296.
8. Schnable PS, Ware D, Fulton RS, Stein JC, Wei FS, Pasternak S, Liang CZ, Zhang JW, Fulton L, Graves TA *et al*. The B73 Maize Genome: Complexity, Diversity, and Dynamics. Science, 2009; **326**(5956):1112-1115.
9. Seim I, Fang X, Xiong Z, Lobanov AV, Huang Z, Ma S, Feng Y, Turanov AA, Zhu Y, Lenz TL *et al*. Genome analysis reveals insights into physiology and longevity of the Brandt's bat *Myotis brandtii*. Nat Commun, 2013; **4**:2212.
10. Valenzano DR, Benayoun BA, Singh PP, Zhang E, Etter PD, Hu C-K, Clément-Ziza M, Willemsen D, Cui R, Harel I *et al*. The African turquoise killifish genome provides insights into evolution and genetic architecture of lifespan. Cell, 2015; **163**(6):1539-1554.
11. Zhou X, Wang B, Pan Q, Zhang J, Kumar S, Sun X, Liu Z, Pan H, Lin Y, Liu G *et al*. Whole-genome sequencing of the snub-nosed monkey provides insights into folivory and evolutionary history. Nat Genet, 2014; **46**:1303-1310.
12. Kuang W-M, Ming C, Li H-P, Wu H, Frantz L, Roos C, Zhang Y-P, Zhang C-L, Jia T, Yang J-Y *et al*. The origin and population history of the endangered golden snub-nosed monkey (*Rhinopithecus roxellana*). Mol Biol Evol, 2018:msy220-msy220.

13. Hong YY, Duo HR, Hong JY, Yang JY, Liu SM, Yu LH, Yi TY. Resequencing and comparison of whole mitochondrial genome to gain insight into the evolutionary status of the Shennongjia golden snub-nosed monkey (SNJ *R-roxellana*). Ecol Evol, 2017; **7**(12):4456-4464.
14. Seo JS, Rhie A, Kim J, Lee S, Sohn MH, Kim CU, Hastie A, Cao H, Yun JY, Kim J *et al*. De novo assembly and phasing of a Korean human genome. Nature, 2016; **538**(7624):243-247.
15. Chaisson MJ, Wilson RK, Eichler EE. Genetic variation and the de novo assembly of human genomes. Nat Rev Genet, 2015; **16**(11):627-640.
16. Jiao YP, Peluso P, Shi JH, Liang T, Stitzer MC, Wang B, Campbell MS, Stein JC, Wei XH, Chin CS *et al*. Improved maize reference genome with single-molecule technologies. Nature, 2017; **546**(7659):524-527.
17. Gordon D, Huddleston J, Chaisson MJP, Hill CM, Kronenberg ZN, Munson KM, Malig M, Raja A, Fiddes I, Hillier LW *et al*. Long-read sequence assembly of the gorilla genome. Science, 2016; **352**(6281):aae0344.
18. Kronenberg ZN, Fiddes IT, Gordon D, Murali S, Cantsilieris S, Meyerson OS, Underwood JG, Nelson BJ, Chaisson MJP, Dougherty ML *et al*. High-resolution comparative analysis of great ape genomes. Science, 2018; **360**(6393):eaar6343.
19. Bickhart DM, Rosen BD, Koren S, Sayre BL, Hastie AR, Chan S, Lee J, Lam ET, Liachko I, Sullivan ST *et al*. Single-molecule sequencing and chromatin conformation capture enable de novo reference assembly of the domestic goat genome. Nat Genet, 2017; **49**:643-650.
20. Luo R, Liu B, Xie Y, Li Z, Huang W, Yuan J, He G, Chen Y, Pan Q, Liu Y *et al*. SOAPdenovo2: an empirically improved memory-efficient short-read de novo assembler. Gigascience, 2012; **1**(1):18.
21. Martin M. Cutadapt removes adapter sequences from high-throughput sequencing reads. EMBnetjournal, 2011; **17**:10 -12.
22. Chin CS, Alexander DH, Marks P, Klammer AA, Drake J, Heiner C, Clum A, Copeland A, Huddleston J, Eichler EE *et al*. Nonhybrid, finished microbial genome assemblies from long-read SMRT sequencing data. Nat Methods, 2013; **10**(6):563-+.
23. Walker BJ, Abeel T, Shea T, Priest M, Abouelliel A, Sakthikumar S, Cuomo CA, Zeng QD, Wortman J, Young SK *et al*. Pilon: An Integrated Tool for Comprehensive Microbial Variant Detection and Genome Assembly Improvement. Plos One, 2014; **9**(11).
24. Adey A, Kitzman JO, Burton JN, Daza R, Kumar A, Christiansen L, Ronaghi M, Amini S, Gunderson KL, Steemers FJ *et al*. In vitro, long-range sequence information for de novo genome assembly via transposase contiguity. Genome Res, 2014; **24**(12):2041-2049.

25. Li H, Durbin R. Fast and accurate short read alignment with Burrows-Wheeler transform. Bioinformatics, 2009; **25**(14):1754-1760.
26. Burton JN, Adey A, Patwardhan RP, Qiu R, Kitzman JO, Shendure J. Chromosome-scale scaffolding of de novo genome assemblies based on chromatin interactions. Nat Biotechnol, 2013; **31**(12):1119-1125.
27. Simao FA, Waterhouse RM, Ioannidis P, Kriventseva EV, Zdobnov EM. BUSCO: assessing genome assembly and annotation completeness with single-copy orthologs. Bioinformatics, 2015; **31**(19):3210-3212.
28. Parra G, Bradnam K, Korf I. CEGMA: a pipeline to accurately annotate core genes in eukaryotic genomes. Bioinformatics, 2007; **23**(9):1061-1067.
29. Jurka J, Kapitonov VV, Pavlicek A, Klonowski P, Kohany O, Walichiewicz J. Repbase Update, a database of eukaryotic repetitive elements. Cytogenet Genome Res, 2005; **110**(1-4):462-467.
30. Price AL, Jones NC, Pevzner PA. De novo identification of repeat families in large genomes. Bioinformatics, 2005; **21 Suppl 1**:i351-358.
31. Benson G. Tandem repeats finder: a program to analyze DNA sequences. Nucleic Acids Res, 1999; **27**(2):573-580.
32. Chin CS, Peluso P, Sedlazeck FJ. Phased diploid genome assembly with single-molecule real-time sequencing. Nat Methods, 2016; **13**(12):1050-1054.
33. Boetzer M, Pirovano W. SSPACE-LongRead: scaffolding bacterial draft genomes using long read sequence information. BMC Bioinformatics, 2014; **15**:211.
34. English AC, Richards S, Han Y, Wang M, Vee V, Qu J, Qin X, Muzny DM, Reid JG, Worley KC *et al*. Mind the gap: upgrading genomes with Pacific Biosciences RS long-read sequencing technology. Plos One, 2012; **7**(11):e47768.
35. Shelton JM, Coleman MC, Herndon N, Lu N, Lam ET, Anantharaman T, Sheth P, Brown SJ. Tools and pipelines for BioNano data: molecule assembly pipeline and FASTA super scaffolding tool. BMC Genomics, 2015; **16**:734-734.
36. Langmead B, Trapnell C, Pop M, Salzberg SL. Ultrafast and memory-efficient alignment of short DNA sequences to the human genome. Genome Biol, 2009; **10**(3):R25.
37. Kurtz S, Phillippy A, Delcher AL, Smoot M, Shumway M, Antonescu C, Salzberg SL. Versatile and open software for comparing large genomes. Genome Biol, 2004; **5**(2):R12.
38. Abyzov A, Urban AE, Snyder M, Gerstein M. CNVnator: an approach to discover, genotype, and characterize typical and atypical CNVs from family and population genome sequencing. Genome Res, 2011; **21**(6):974-984.

39. Griffiths-Jones S, Moxon S, Marshall M, Khanna A, Eddy SR, Bateman A. Rfam: annotating non-coding RNAs in complete genomes. Nucleic Acids Res, 2005; **33**:D121-D124.
40. Nawrocki EP, Kolbe DL, Eddy SR. Infernal 1.0: inference of RNA alignments (vol 25, pg 1335, 2009). Bioinformatics, 2009; **25**(13):1713-1713.
41. Lowe TM, Eddy SR. tRNAscan-SE: a program for improved detection of transfer RNA genes in genomic sequence. Nucleic Acids Res, 1997; **25**(5):955-964.
42. Stanke M, Keller O, Gunduz I, Hayes A, Waack S, Morgenstern B. AUGUSTUS: ab initio prediction of alternative transcripts. Nucleic Acids Res, 2006; **34**:W435-W439.
43. Majoros WH, Pertea M, Salzberg SL. TigrScan and GlimmerHMM: two open source ab initio eukaryotic gene-finders. Bioinformatics, 2004; **20**(16):2878-2879.
44. Burge C, Karlin S. Prediction of complete gene structures in human genomic DNA. J Mol Biol, 1997; **268**(1):78-94.
45. Guigo R. Assembling genes from predicted exons in linear time with dynamic programming. J Comput Biol, 1998; **5**(4):681-702.
46. Korf I. Gene finding in novel genomes. BMC Bioinformatics, 2004; **5**:59.
47. Kent WJ. BLAT - The BLAST-like alignment tool. Genome Res, 2002; **12**(4):656-664.
48. Birney E, Clamp M, Durbin R. GeneWise and Genomewise. Genome Res, 2004; **14**(5):988-995.
49. Grabherr MG, Haas BJ, Yassour M, Levin JZ, Thompson DA, Amit I, Adiconis X, Fan L, Raychowdhury R, Zeng QD *et al*. Full-length transcriptome assembly from RNA-Seq data without a reference genome. Nat Biotechnol, 2011; **29**(7):644-U130.
50. Haas BJ, Delcher AL, Mount SM, Wortman JR, Smith RK, Hannick LI, Maiti R, Ronning CM, Rusch DB, Town CD *et al*. Improving the Arabidopsis genome annotation using maximal transcript alignment assemblies. Nucleic Acids Res, 2003; **31**(19):5654-5666.
51. Kim D, Pertea G, Trapnell C, Pimentel H, Kelley R, Salzberg SL. TopHat2: accurate alignment of transcriptomes in the presence of insertions, deletions and gene fusions. Genome Biol, 2013; **14**(4):R36.
52. Trapnell C, Roberts A, Goff L, Pertea G, Kim D, Kelley DR, Pimentel H, Salzberg SL, Rinn JL, Pachter L. Differential gene and transcript expression analysis of RNA-seq experiments with TopHat and Cufflinks. Nat Protoc, 2012; **7**(3):562-578.
53. Haas BJ, Salzberg SL, Zhu W, Pertea M, Allen JE, Orvis J, White O, Buell CR, Wortman JR. Automated eukaryotic gene structure annotation using EVidenceModeler and the program to assemble spliced alignments. Genome Biol, 2008; **9**(1):R7.

54. Bairoch A, Apweiler R, Wu CH, Barker WC, Boeckmann B, Ferro S, Gasteiger E, Huang H, Lopez R, Magrane M *et al*. The Universal Protein Resource (UniProt). Nucleic Acids Res, 2005; **33**(Database issue):D154-D159.
55. Kanehisa M, Goto S. KEGG: kyoto encyclopedia of genes and genomes. Nucleic Acids Res, 2000; **28**(1):27-30.
56. Mitchell AL, Attwood TK, Babbitt PC, Blum M, Bork P, Bridge A, Brown SD, Chang HY, El-Gebali S, Fraser MI *et al*. InterPro in 2019: improving coverage, classification and access to protein sequence annotations. Nucleic Acids Res, 2019; **47**(D1):D351-D360.
57. Finn RD, Coggill P, Eberhardt RY, Eddy SR, Mistry J, Mitchell AL, Potter SC, Punta M, Qureshi M, Sangrador-Vegas A *et al*. The Pfam protein families database: towards a more sustainable future. Nucleic Acids Res, 2016; **44**(D1):D279-D285.
58. Ashburner M, Ball CA, Blake JA, Botstein D, Butler H, Cherry JM, Davis AP, Dolinski K, Dwight SS, Eppig JT *et al*. Gene Ontology: tool for the unification of biology. Nat Genet, 2000; **25**(1):25-29.
59. Pertea G, Huang X, Liang F, Antonescu V, Sultana R, Karamycheva S, Lee Y, White J, Cheung F, Parvizi B *et al*. TIGR Gene Indices clustering tools (TGICL): a software system for fast clustering of large EST datasets. Bioinformatics, 2003; **19**(5):651-652.
60. Li H, Coghlan A, Ruan J, Coin LJ, Heriche JK, Osmotherly L, Li RQ, Liu T, Zhang Z, Bolund L *et al*. TreeFam: a curated database of phylogenetic trees of animal gene families. Nucleic Acids Res, 2006; **34**:D572-D580.
61. Guindon S, Delsuc F, Dufayard J-F, Gascuel O: **Estimating maximum likelihood phylogenies with PhyML**. In: *Bioinformatics for DNA sequence analysis.* Springer; 2009: 113-137.
62. Posada D. jModelTest: phylogenetic model averaging. Mol Biol Evol, 2008; **25**(7):1253-1256.
63. Yang Z. PAML 4: Phylogenetic Analysis by Maximum Likelihood. Mol Biol Evol, 2007; **24**(8):1586-1591.
64. De Bie T, Cristianini N, Demuth JP, Hahn MW. CAFE: a computational tool for the study of gene family evolution. Bioinformatics, 2006; **22**(10):1269-1271.
65. Kumar S, Stecher G, Suleski M, Hedges SB. TimeTree: A resource for timelines, timetrees, and divergence times. Mol Biol Evol, 2017; **34**(7):1812-1819.
66. Huang DW, Sherman BT, Lempicki RA. Bioinformatics enrichment tools: paths toward the comprehensive functional analysis of large gene lists. Nucleic Acids Res, 2009; **37**(1):1-13.
67. Beissbarth T, Speed TP. GOstat: find statistically overrepresented Gene Ontologies within a group of genes. Bioinformatics, 2004; **20**(9):1464-1465.

Figure 1

Click here to access/download;Figure;fig 1_monkey.JPG

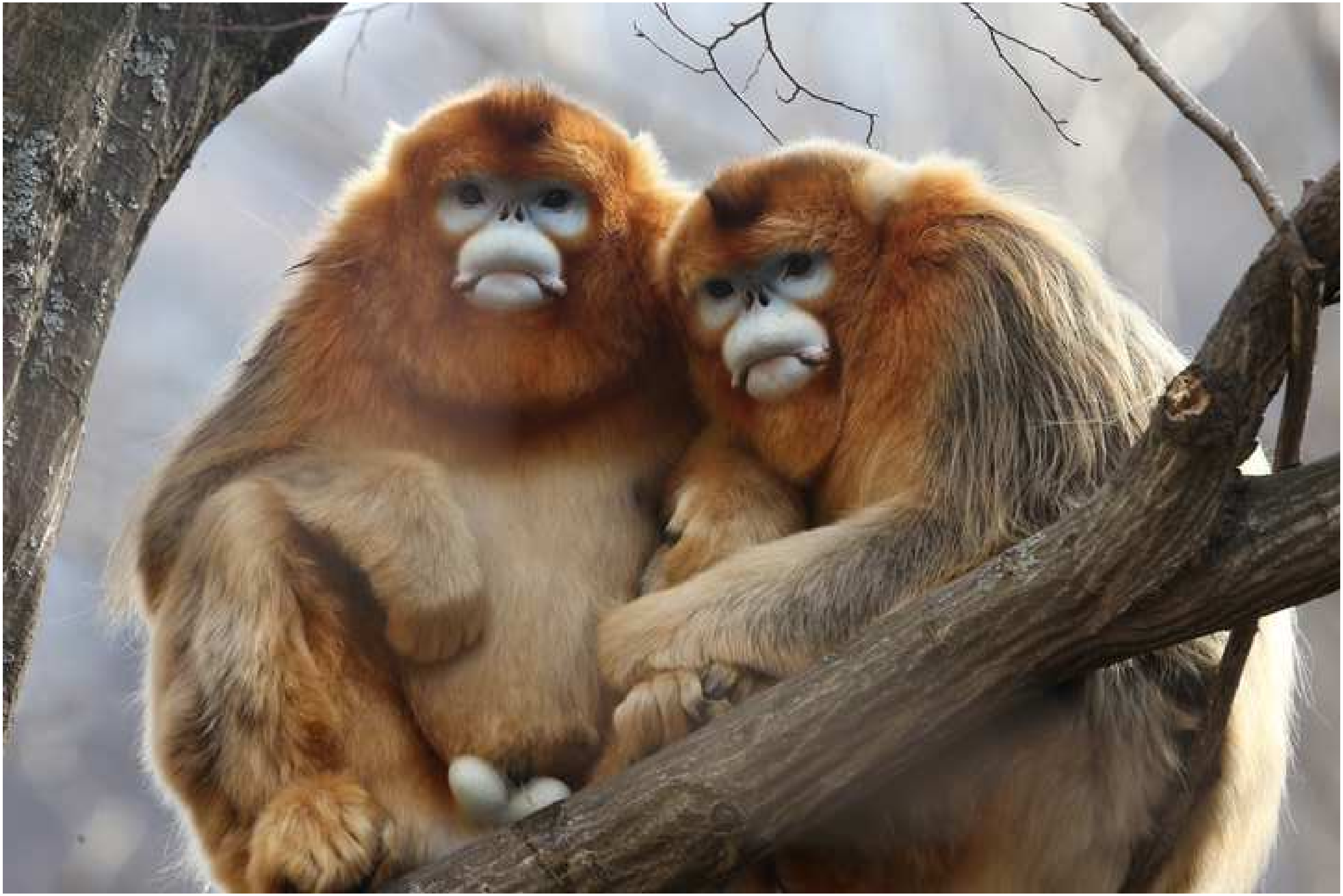

Figure 2

Click here to access/download;Figure;fig. 2_HIC.png

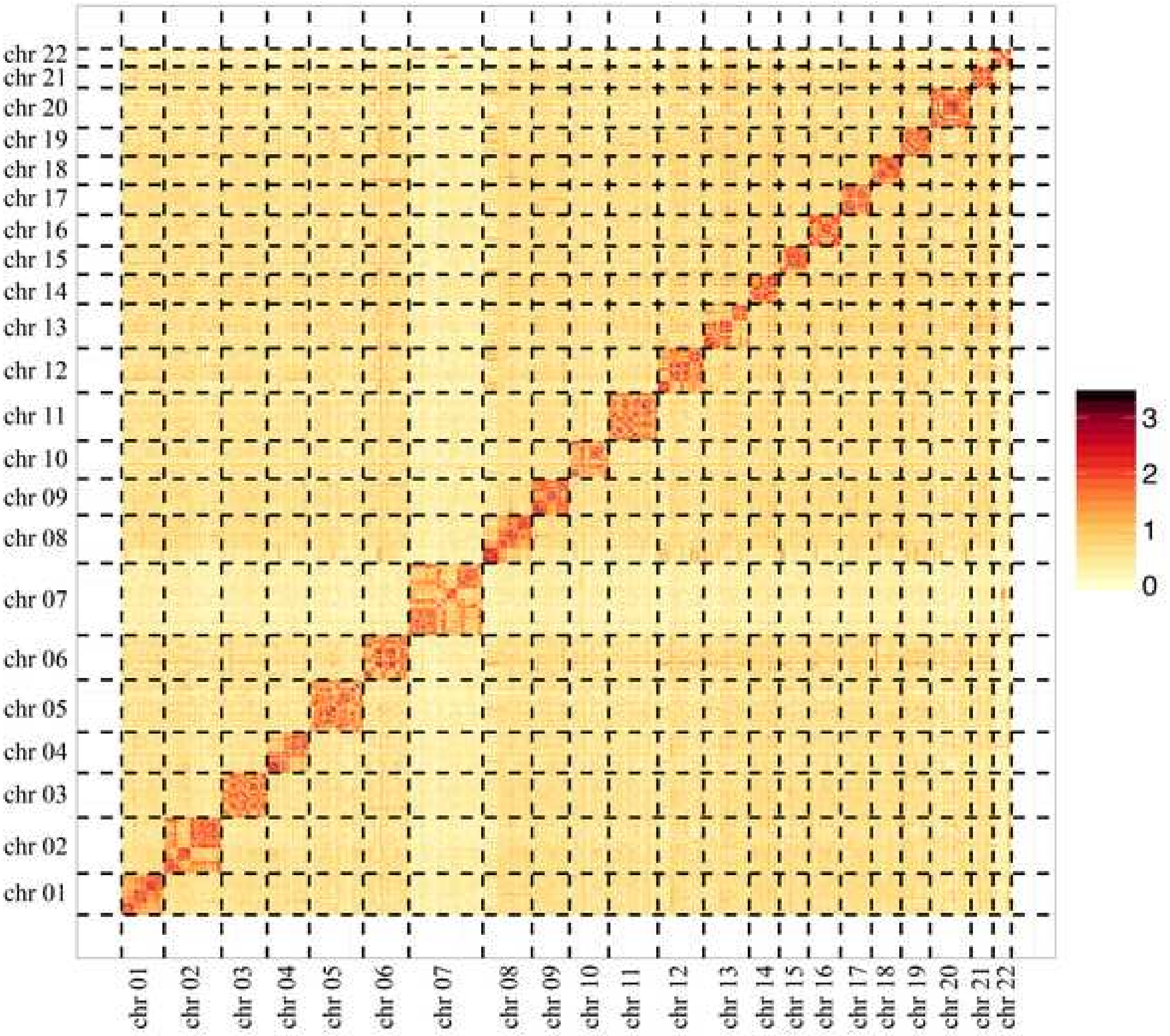

Figure 3

Click here to access/download;Figure;fig. 3.pdf

a

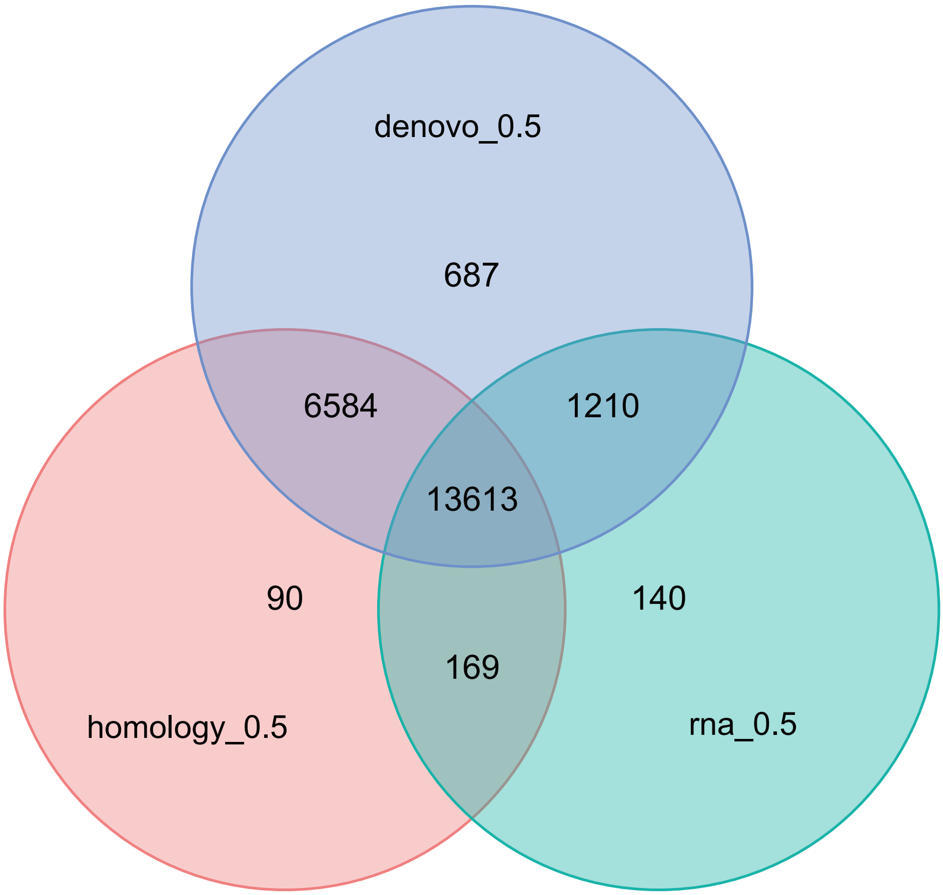

b

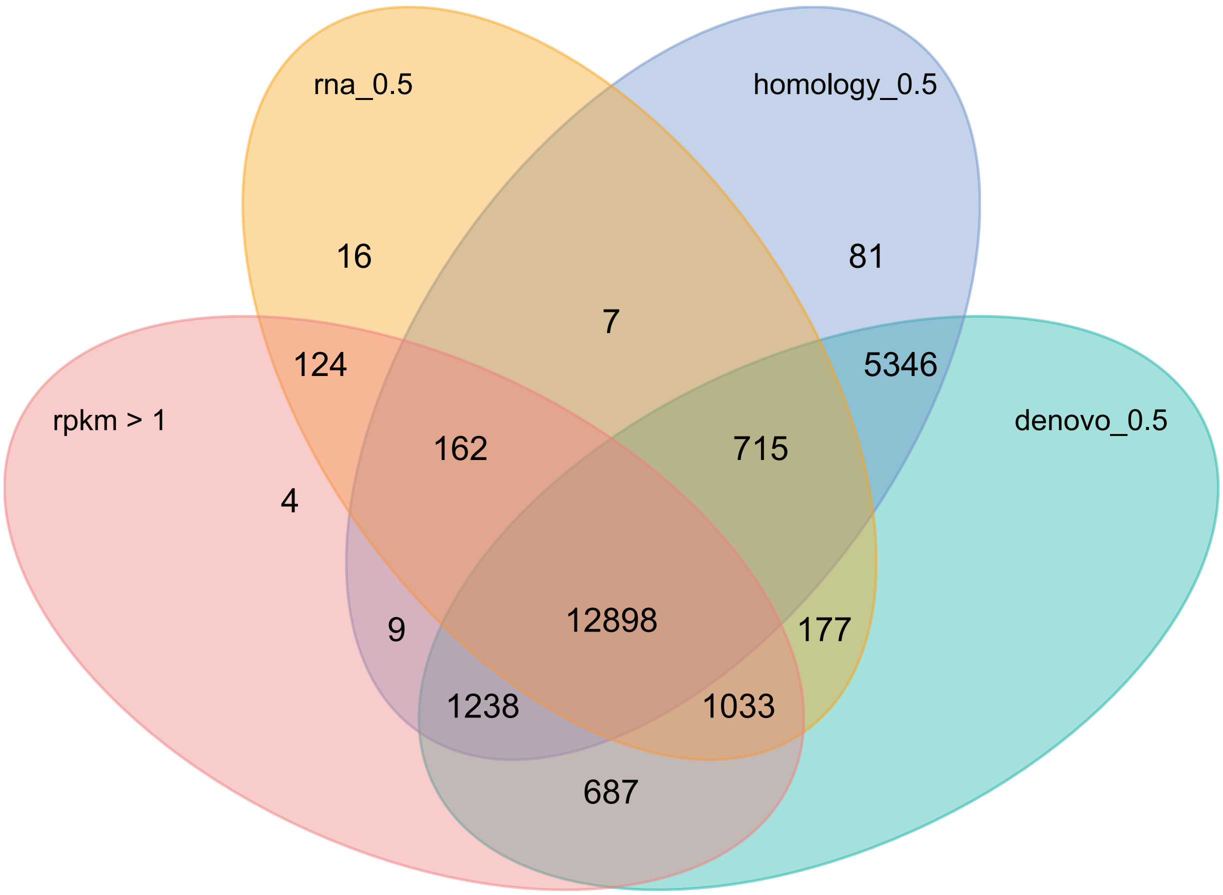

Figure 4

Click here to access/download;Figure;fig. 4_phylogeny.png

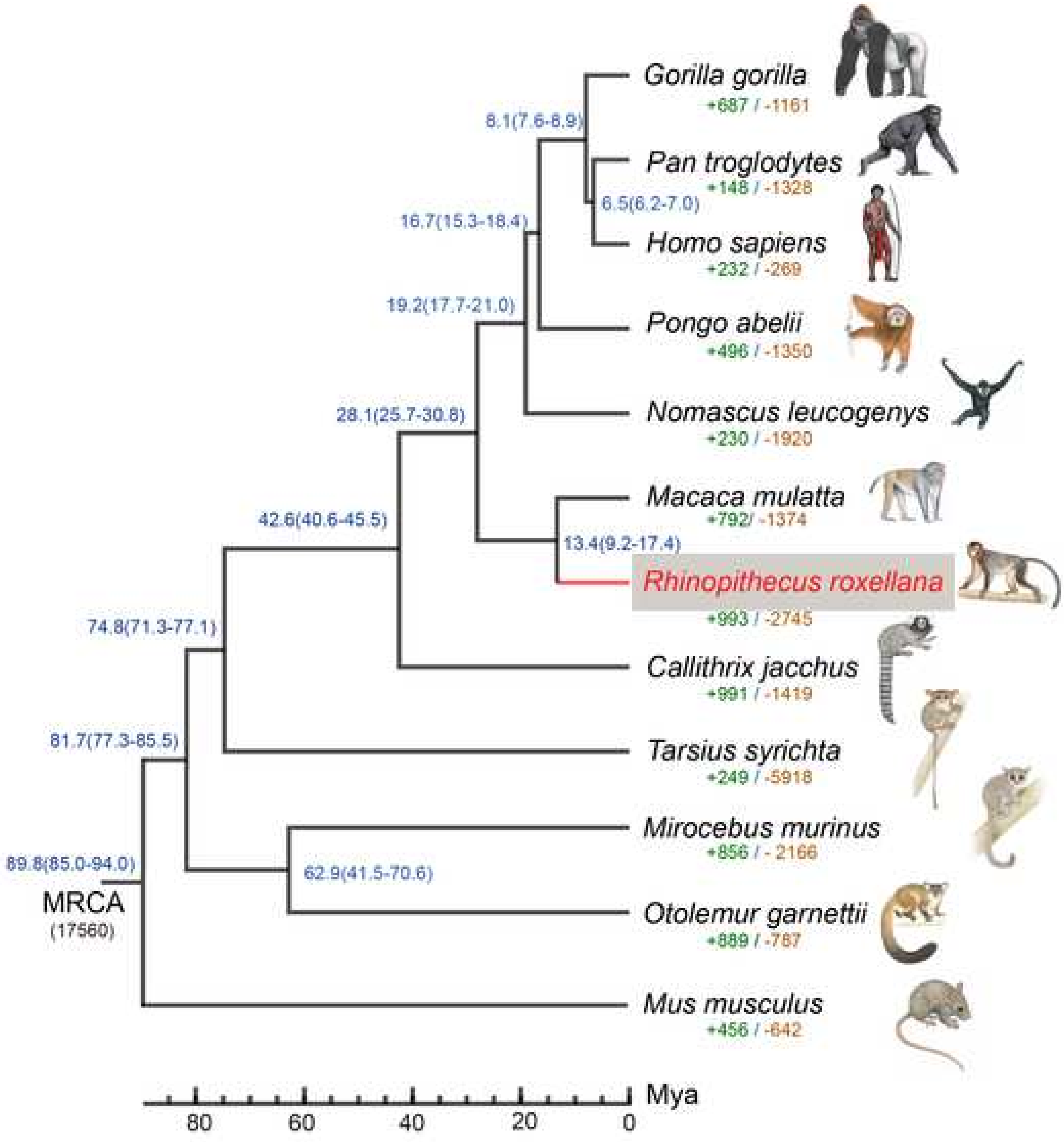

Click here to access/download
Supplementary Material
SI_giga_0505.docx

Personal Cover Click here to access/download;Personal Cover;Coverletter-0505_giga.docx

Dear Scott and Handling Editor,

Thanks for handling our manuscript, and we appreciate the valuable comments from you and three referees.

After digesting these comments, we have substantially revised our manuscript. Firstly, we employed an English-language editing service, LetPub, to polish our wording. Secondly, we expanded the methodological details substantially including analysis procedures, software versions and settings, we also capture these details using protocols.io.. as recommended. Thirdly, we added some key details about the generated data, including sequencing data, calibration times and N50 length to be more clear. Forth, we performed several additional analysis including CNVs identification, synteny analysis and SNP calling et al. as reviewers suggested. In addition, other comments were also addressed following the instructions from you and three referees.

In this revised version, corrections were made in a document with "Track Changes" mode. Point-by-point responses to the reviewers are also submitted. After addressing the issues raised, we feel the quality of the paper is much improved and hope that our revised manuscript is acceptable for publication in *GigaScience*.

Thanks for your consideration, we look forward to your advice.

Yours sincerely,

Xiao-Guang Qi
Shaanxi Key Laboratory for Animal Conservation
College of Life Sciences
Northwest University
Email: qixg@nwu.edu.cn
