# Supplementary material for: A high-quality genome assembly for the endangered golden snub-nosed monkey (Rhinopithecus roxellana)
Source: Gigascience. 2019 Aug 22;8(8):giz098. doi: 10.1093/gigascience/giz098 (PMC6705546; doi:10.1093/gigascience/giz098)
Supplement: giz098_GIGA-D-19-00030_Revision_2 [file giz098_giga-d-19-00030_revision_2.pdf]

GigaScience

A high-quality genome assembly for the endangered golden snub-nosed monkey (Rhinopithecus roxellana)

--Manuscript Draft--

**Manuscript Number:**	GIGA-D-19-00030R2
**Full Title:**	A high-quality genome assembly for the endangered golden snub-nosed monkey (Rhinopithecus roxellana)
**Article Type:**	Data Note
**Funding Information:**	National Natural Science Foundation of China (31622053) — Dr. Xiao-Guang Qi
**Abstract:**	Background: The golden snub-nosed monkey (Rhinopithecus roxellana) is an endangered colobine species endemic to China. This species has several distinctive traits and is an ideal model for analyses of the evolutionary development of social structures due to its unique social organization. Although a genome assembly for the subspecies R. roxellana hubeiensis is available, this assembly is incomplete and fragmented because it was constructed using short read sequencing technology. Thus, information important for the understanding of R. roxellana, such as genome structural variation and repeat sequences, may be absent from the available assembly. Therefore, a high-quality reference genome is needed.<br>Findings: To obtain a high-quality chromosomal assembly for R. roxellana qinlingensis, we used five different methods: Pacific Bioscience single-molecule real-time sequencing, Illumina paired-end sequencing, BioNano optical maps, 10X Genomics link-reads, and high-throughput chromosome conformation capture. The assembled genome was ~3.04 Gb, with a contig N50 of 5.72 Mb and a scaffold N50 of 144.56 Mb. This represented a 100-fold improvement over the previously published genome. In the new genome, 22,497 protein-coding genes were predicted, of which 22,053 were functionally annotated. Gene family analysis showed that 993 and 2,745 gene families were expanded and contracted, respectively, in the R. r. qinlingensis genome. The reconstructed phylogeny recovered a close relationship between Rhinopithecus rollexana and Macaca mulatta, and these two species diverged approximately 13.4 MYA.<br>Conclusion: We constructed a high-quality genome assembly of Qinling golden snub-nosed monkey; this genome had superior continuity and accuracy, which might be useful as reference for future genetic studies in this species. In addition, the updated genome assembly might improve our understanding of this species and might be particularly relevant to conservation efforts. Furthermore, this high-quality genome might serve as a new standard reference genome for colobine primates.
**Corresponding Author:**	Xiao-Guang Qi<br><br>CHINA
**Corresponding Author Secondary Information:**	
**Corresponding Author's Institution:**	
**Corresponding Author's Secondary Institution:**	
**First Author:**	Xiao-Guang Qi
**First Author Secondary Information:**	
**Order of Authors:**	Xiao-Guang Qi
	Lu Wang
	Jinwei Wu
	Xiaomei Liu

Powered by Editorial Manager® and ProduXion Manager® from Aries Systems Corporation

	Dandan Di
	Yuhong Liang
	Yifei Feng
	Suyun Zhang
	Baoguo Li
**Order of Authors Secondary Information:**	
**Response to Reviewers:**	Editor Comments to Author:<br><br>Based on these reports, and my own assessment as Editor, I am pleased to inform you that it is potentially acceptable for publication in GigaScience, once you have carried out some final essential revisions suggested by our reviewers.<br><br>Response to this comment<br>We are delighted to see these positive comments. Following the instruction from the editor and the two reviewers, we have fully addressed all comments. See our responses below.<br><br><br>Reviewer Comments to Author:<br><br>Reviewer #1:<br>This manuscript reports a new whole genome assembly for an interesting nonhuman primate species, Rhinopithecus roxellana. This is a colobine species that has a number of unusual characteristics, including but not limited to unusual pelage, highly derived facial morphology, and social organization that is not entirely unique but is rare among Old World monkeys or other anthropoids. There are five species in the genus, and all are threatened or endangered, so there is a conservation benefit to this genome sequencing as well as basic comparative primate evolutionary genomics. There is a previously published whole genome assembly for this species, but this new assembly is a significant improvement (see below). Consequently, there are several elements of this work that make it noteworthy.<br>This is a revised manuscript. This version of the paper is significantly improved from the first version. Most of my comments and concerns have been satisfactorily addressed. But I do have some minor issues with this revision. I believe the authors can easily correct these remaining problems.<br><br>1) The sentence in lines 85-87 ("Genomic analyses have helped…") seems unnecessary and out of place. I suggest deleting this sentence.<br>Response to comment 1<br>Following this comment, we deleted this sentence. Please see lines 58 - 60 on page 4 for details.<br><br>2) Line 141. An N50 value is not the same as an average. The authors should indicate whether this value of 16.69 kb is an average or an N50. The latter is the preferred way to report this statistic.<br>Response to comment 2<br>Thanks for this comment. We agree that an N50 value is not the same as an average. we checked this value carefully and found that it indicated an N50 value. We changed the statement as follows (Lines 105, bottom of page 6).<br><br>“TheN50 length of the PacBio reads was 16.69 kb.”.<br><br>3) Line 149: Same comment as #2<br>Response to comment 3<br>Thanks for this comment. We changed the statement as follows (Line 110 on top of page 7):<br>“The N50 length of the molecules used for optical mapping was 338 kb.”.<br><br>4) Line 301: I would suggest adding the word "non-reference" so that it reads "We found that the homozygous non-reference SNPs comprise 0.0004%...."<br>Response to comment 4

Following this comment, we added “non-reference” in this sentence. (Line 201 on top of page 12)

“We found that the homozygous non-reference SNPs (single nucleotide polymorphism) comprised 0.0004% of all SNPs (7,690 of 559,048).”

5) Line 309: I think you need "also" inserted - "completeness was also measured…"
Response to comment 5
Thanks for this comment. We followed this comment and inserted “also” in this sentence. (Line 209 on page 12)

“Assembly completeness was also measured using the core eukaryotic gene (CEG)-mapping approach (CEGMA v2.5) [31]”.

6) Line 317: There is at least one word missing or out of place here. Please edit.

Response to comment 6
Thanks for your valuable comment. We are sorry we made a mistake here. We revised this sentence (lines 217, bottom of page 12).

“Repeat sequences account for a large proportion of the total genome. It is thus important to identify repeat elements.”.

7) Lines 336 - 342. I do not understand how the authors identify copy number variation when they did not study and do not report DNA sequences from multiple individuals. There is only one reference sequence reported in this paper. Did the authors look at copy number differences between haplotypes of that one diploid monkey? This section is very confusing to me. Either the source of the samples used for CNV analysis must be presented, or this could be deleted. Some editing is required.

Response to comment 7
Thanks for your valuable comment. It is true that analysis of one sample does not show copy number variations. We are sorry that this section was not clear enough. The term CNVs analysis, should be better termed duplications in our study. And those duplicate sequences were identified based on read depth. We added several sentences to address this comment (Lines 237-247, top of page 14):

“We also performed duplicate sequences identification analysis, which was fulfilled based on the read depth of Illumina short reads. In brief, we first mapped the Illumina short reads to the assembled genome using BWA with default parameters. Then, the sorted mapping bam file was used as input for CNVnator v0.3.3 [35], a tool targeting alterations in the read depth, with the parameters of “-unique -his 100 -stat 100 -call 100.”. The obtained duplicate sequences were filtered, retaining only those where q0 was <0.5 and e-val1 was <0.05. After filtering, 676 duplicate sequences remained, with a total length of 9,198,900 bp (Supplementary Table S12). Further analysis showed that 101 duplications located at the end of scaffolds (5% of the total length in both ends). And there were 136 gene present in the duplicated regions, these genes were mainly involved in basic biological processes such as ribonucleoside binding, phosphatase activity, and protein dephosphorylation et al.”.

8) Lines 389 - 391. What animal was used to obtain the heart and skin tissue for RNA sequencing? Were these tissues obtained from the same animal used for DNA sequencing and reference assembly? Please state source of tissue for RNA sequencing.

Thanks for this comment. The animal used for RNA sequencing was the same individual with DNA sequencing and reference assembly. We stated source of tissue for RNA sequencing in Lines 275 -276 (top of page 16):
“High-quality RNAs from the heart and skin tissue of the R. roxellana qinlingensis specimen (the same individual used for DNA sequencing and reference assembly) were sequenced on an Illumina Novaseq 6000 platform.”.

9) I think Figure 2 would be better in the Supplement than main text. If the authors think this is important, presenting it in the supplement is fine. But I do not see that this

	contributes significantly to the major findings of the paper. If the authors feel strongly that it must remain in the main text, that is acceptable and I would not make an issue out of that. But I do not see the major significance beyond providing validation. No biological insight is provided by this figure.<br><br>Response to comment 9<br>Thanks for this valuable comment. The fig. 2 was based on the interaction frequencies between pairs of 100-kb genomic regions, which could be used to indicate the reliability of our assembly. Despite that no great biological insights provided by this figure, this figure was generated with great effort and could convert some information about our data quality, an important topic in this high quality genome work. Thus, the Figure 2 would be better in the main text.<br><br><br><br>Reviewer #2: The revision is substantially improved and most of the concerns of the reviewers have been resolved.<br><br>CNV analysis: (line 337). I think these are better termed duplications, not CNVs, as analysis of one sample does not show whether they are copy number variable in the population. Are the duplications found at the end of contigs? Were there any gene annotations present in the duplicated sequences.<br>Response to this comment<br>Thanks for your valuable comment. It is true that analysis of one sample does not show copy number variations. We are sorry that this section was not clear enough. The term CNVs analysis, should be better termed duplications in our study. And those duplicate sequences were identified based on read depth. Further analysis of the positions of these duplicate sequences showed that 101 duplications located at the end of scaffolds. In addition, there were 136 genes present in the duplicated regions, they were mainly involved in basic biological processes. We added several sentences to address this comment (Lines 237-247, top of page 14):<br><br>“We also performed duplicate sequences identification analysis, which was fulfilled based on the read depth of Illumina short reads. In brief, we first mapped the Illumina short reads to the assembled genome using BWA with default parameters. Then, the sorted mapping bam file was used as input for CNVnator v0.3.3 [35], a tool targeting alterations in the read depth, with the parameters of “-unique -his 100 -stat 100 -call 100.”. The obtained duplicate sequences were filtered, retaining only those where q0 was <0.5 and e-val1 was <0.05. After filtering, 676 duplicate sequences remained, with a total length of 9,198,900 bp (Supplementary Table S12). Further analysis showed that 101 duplications located at the end of scaffolds (5% of the total length in both ends). And there were 136 gene present in the duplicated regions, these genes were mainly involved in basic biological processes such as ribonucleoside binding, phosphatase activity, and protein dephosphorylation et al.”.
**Additional Information:**	
**Question**	**Response**
Are you submitting this manuscript to a special series or article collection?	No
**Experimental design and statistics**<br><br><br>Full details of the experimental design and statistical methods used should be given in the Methods section, as detailed in our Minimum Standards Reporting Checklist. Information essential to interpreting the data presented should be made available in the figure legends.	Yes

Powered by Editorial Manager® and ProduXion Manager® from Aries Systems Corporation

Have you included all the information requested in your manuscript?	
**Resources**<br><br>A description of all resources used, including antibodies, cell lines, animals and software tools, with enough information to allow them to be uniquely identified, should be included in the Methods section. Authors are strongly encouraged to cite Research Resource Identifiers (RRIDs) for antibodies, model organisms and tools, where possible.<br><br>Have you included the information requested as detailed in our Minimum Standards Reporting Checklist?	Yes
**Availability of data and materials**<br><br>All datasets and code on which the conclusions of the paper rely must be either included in your submission or deposited in publicly available repositories (where available and ethically appropriate), referencing such data using a unique identifier in the references and in the "Availability of Data and Materials" section of your manuscript.<br><br>Have you have met the above requirement as detailed in our Minimum Standards Reporting Checklist?	Yes

Powered by Editorial Manager® and ProduXion Manager® from Aries Systems Corporation

Manuscript
Click here to access/download;Manuscript;Manuscript_giga_0609_version_
Click here to view linked References

A high-quality genome assembly for the endangered golden snub-nosed monkey (*Rhinopithecus roxellana)*

Lu Wang[1,†], Jinwei Wu[1,†], Xiaomei Liu[1], Dandan Di[1], Yuhong Liang[1], Yifei Feng[1], Suyun Zhang[1], Baoguo Li[1,2], Xiao-Guang Qi[1,*]

[1] Shaanxi Key Laboratory for Animal Conservation, College of Life Sciences, Northwest University, Xi'an, 710069, China.

[2] Center for Excellence in Animal Evolution and Genetics, Chinese Academy of Sciences, Kunming, 650223, China.

[*]**Correspondence address.** Xiao-Guang Qi, ORCID: 0000-0003-2602-4441, E-mail: qixg@nwu.edu.cn

[†]These authors contributed equally to this work.

ABSTRACT

Background: The golden snub-nosed monkey (*Rhinopithecus roxellana*) is an endangered colobine species endemic to China. This species has several distinctive traits and is an ideal model for analyses of the evolutionary development of social structures due to its unique social organization. Although a genome assembly for the subspecies *R. roxellana hubeiensis* is available, this assembly is incomplete and fragmented because it was constructed using short read sequencing technology. Thus, information important for the understanding of *R. roxellana*, such as genome structural variation and repeat sequences, may be absent from the available assembly. Therefore, a high-quality reference genome is needed.

Findings: To obtain a high-quality chromosomal assembly for *R. roxellana qinlingensis,* we used five different methods: Pacific Bioscience single-molecule real-time sequencing, Illumina paired-end sequencing, BioNano optical maps, 10X Genomics link-reads, and high-throughput chromosome conformation capture. The assembled genome was ~3.04 Gb, with a contig N50 of 5.72 Mb and a scaffold N50 of 144.56 Mb. This represented a 100-fold improvement over the previously published genome. In the new genome, 22,497 protein-coding genes were predicted, of which 22,053 were functionally annotated. Gene family analysis showed that 993 and 2,745 gene families were expanded and contracted, respectively, in the *R. r. qinlingensis*

genome. The reconstructed phylogeny recovered a close relationship between *Rhinopithecus rollexana* and *Macaca mulatta*, and these two species diverged approximately 13.4 MYA.

Conclusion: We constructed a high-quality genome assembly of Qinling golden snub-nosed monkey; this genome had superior continuity and accuracy, which might be useful as reference for future genetic studies in this species. In addition, the updated genome assembly might improve our understanding of this species and might be particularly relevant to conservation efforts. Furthermore, this high-quality genome might serve as a new standard reference genome for colobine primates.

Keywords: high-quality; *Rhinopithecus roxellana*; genome assembly; annotation; BioNano optical maps

Background information

The snub-nosed monkeys (genus *Rhinopithecus*) consist of five endangered species narrowly restricted to China and Vietnam [1]. Of those, the golden snub-nosed monkey (*Rhinopithecus roxellana*, NCBI:txid61622), also known as the Sichuan snub-nosed monkey, has the northernmost distribution of all Asian colobine species; this monkey is found only in three isolated regions in central and northwest China (the Sichuan, Gansu, Shaanxi, and Hubei Provinces) [2, 3]. The golden snub-nosed monkey is characterized by several distinctive traits,

including golden fur, a blue facial color, an odd-shaped nose, and folivory. In addition, the species has a unique multilevel social system; such complex systems are found only in a few mammals, including humans [4]. Therefore, the Qinling golden snub-nosed monkey is an ideal model for the analysis of social structure evolution in primates and may also provide opportunities to investigate evolutionary and socio-anthropological patterns of human society.

Based on morphological variations and discontinuous distributions, *R. roxellana* is distinguished into three subspecies: *R. r. roxellana* from the Minshan Mountain in the Sichuan and Gansu Provinces, *R. r. qinlingensis* from the Qinling Mountain in Shaanxi Province, and *R. r. hubeiensis* from Shennongjia Mountain in Hubei Province [3]. Recent studies of *R. r.* have focused on behavioral dynamics, population history, and social systems [5-7]. To date, only a single genome assembly is available for the golden snub-nosed monkey. This assembly, published in 2014, was derived from short sequencing reads generated by the Illumina HiSeq 2000 platform [8]. Several studies have been published based on these data, including analyses of the folivorous dietary adaptations of *R. r.* and its evolutionary history [8-10]. Despite the utility of this previously published data, much relevant information, including structural variations and repeat sequences, is largely absent or unreliable due to the incomplete and fragmented genome assembly [11, 12].

Owing to advances in sequencing technology, it is now possible to obtain high-quality genome assemblies that can provide new insights in organismal research. Indeed, many

previously unreported transposable elements and specific genes in maize were identified using an improved reference genome [13]. By combining new sequencing approaches, Seo et al. [11] discovered clinically relevant structural variants and previously unreported genes in the updated human genome. New sequencing technologies, including Pacific Bioscience's single-molecule real-time (SMRT) sequencing, BioNano optical mapping, and Hi-C-based chromatin interaction maps, have been used in several species closely related to humans, including gorillas (*Gorilla gorilla gorilla*) [14], chimpanzees (*Pan troglodytes*) [15], and Sumatran orangutans (*Pongo abelii*) [15], as well as in other species, including the domestic goat (*Capra hircus*) [16]. Importantly, it was estimated that 87% of the missing reference exons and incomplete gene models were recovered using the new gorilla assembly [14]. In addition, several novel genes expressed in the brain were identified using the new orangutan assembly, and complete immune genes with longer repetitive structures were identified in the updated goat genome [16]. However, the *R. r.* genome has not yet been updated using new sequencing approaches, slowing progress towards a better understanding of this endangered species.

Here, we report a greatly improved assembly of the reference genome for *R. r.* generated by a combination of five technologies: SMRT sequencing from Pacific Biosciences (PacBio), HiSeq paired-end sequencing from Illumina (HiSeq), BioNano optical maps (BioNano), 10X Genomics link-reads (10X Genomics), and high-throughput chromosome conformation capture (Hi-C). Our results represent the first colobine genome sequenced and assembled with

both long reads and short reads. This updated genome assembly may allow us to further investigate *R. r.*, providing new opportunities to analyze evolutionary history and to identify genetic changes associated with the development of specific traits in this species. Such analyses may provide insights helpful for the conservation of this endangered primate. In addition, this genome, which has superior continuity and accuracy, will act as a new reference genome for colobine primates.

Data Description

Sample collection and sequencing

The animal used for sequencing was an adult male *R. r. qinlingensis* from Qinling Mountain, who died of natural causes, and then stored shortly after death in an ultra-cold storage freezer at Louguantai Breeding Centre, Xi'an, Shaanxi Province, China. Total genomic DNA was extracted from the heart tissue. To acquire a high-quality genome assembly, we combined five sequencing methods. Initially, PacBio SMRT sequencing was performed on the SEQUEL platform following the manufacturer's instructions. After quality control, during which subreads shorter than 500 bp were removed, 304.84 Gb clean long reads (95.86X coverage) remained. The N50 length of the PacBio reads was 16.69 kb. Simultaneously, paired-end sequencing was performed using an Illumina NovaSeq 6000 platform, with an insert size of 350 bp. Then those short reads were filtered using the SOAPdenovo2 software [17], removing

reads with adapters, contaminations, >10% unknown bases (N), or low quality. After filtering, 423.32 Gb clean reads remained (133.12X coverage). A high-quality optical genome map was also constructed with the Irys platform (BioNano Genomics). The N50 length of the molecules used for optical mapping was 338 kb. The average BioNano optimal marker density examined was 11.66 per 100 kb, while the average marker density was 12.62 per 100 kb for the predicted map based on the assembled contigs. Thus, the observed BioNano map was consistent with the predicted map. The BioNano map generated 463.75 Gb of large DNA molecules. Next, 10X genomic linked-reads sequencing was performed on an Illumina Hiseq Xten platform, generating 348.41 Gb clean reads (109.56X coverage). Finally, a Hi-C library was prepared and sequenced with an Illumina NovaSeq 6000 platform to produce a chromosome-scale scaffolding of the genome assembly. Adapter sequences and low-quality reads were discarded using Cutadapt v1.0 [18] with the parameters "-e 0.1 -O 5 -m 100 –n 2 --pair-filter=both," yielding 310.92 Gb clean data (97.77X coverage). Detailed sequencing statistics are given in **Table 1**.

***De novo* assembly of the *R. roxellana* genome**

An estimation of genome size would increase our understanding of *R. roxellana* and the challenges in sequencing it. Thus, we estimated the size of the *R. roxellana* genome as $G = (K_{total} - K_{error})/D$, where G represented genome size, K_{total} represented the total number of k-

mers, K_{error} represented the number of k-mers with sequencing errors, and *D* indicated the k-mer depth. We generated 109,210,004,556 k-mers, 1,159,024,556 of which had sequencing errors. The peak k-mer depth was 34. Thus, the genome size of *R. roxellana* was estimated to be about 3.18 Gb. The distribution of k-mer frequencies is given in **Supplementary Fig. S1**.

The *de novo* assembly of the newly sequenced *R. roxellana* genome was performed in four progressive stages. First, long reads obtained from the PacBio platform were assembled as follows: detection of overlap and read correction, detection of overlap between pairs of corrected reads, and string graph construction. Assembly of the PacBio long reads was performed using FALCON (version 0.4.0, Falcon, RRID:SCR_016089) [19] with the parameter set "length_cutoff = 5000, length_cutoff_pr = 5000, pa_HPCdaligner_option = -v -B128 -e.70 -k14 -h128 -l2000 -w8 -T8 -s700, ovlp_HPCdaligner_option = -v -B128 -e.96 -k16 -h480 -l1500 -w8 -T16 -s700". Next, the assembled PacBio contigs was polished using Quiver (SMRTLink version 5.1.0) with PacBio long reads [20], and also the contig assembly was corrected by Pilon-1.18 (java -Xmx500G -jar pilon-1.18.jar --diploid --threads 30) with Illumina short reads [21]. The contig N50 of the initial assembly was 4.74 Mb (**Supplementary Table S1**). Using the initial genome assembly, SSPACE-LongRead v1-1 [22] was implemented for getting a longer scaffold by processing PacBio long reads and the initial genome assembly with the command "perl SSPACE-LongRead.pl -c <contig-sequences> -p <pacbio-reads>." This procedure generated a genome assembly with scaffold N50 of 7.81 Mb

(**Supplementary Table S2**). The remaining gaps in the assembly were closed using the PBjelly module in the PBSuite (version 15.8.24) [23] with default settings. Thus, at the end of the first stage, the genome assembly had a contig N50 of 5.72 Mb and a scaffold N50 of 8.20 Mb (**Supplementary Table S3**).

In the second stage, the BioNano molecules were filtered, requiring a minimum length of 150 kb and minimum of nine labels per molecule. Then, a genome map was assembled *de novo* with IrysView (version 2.3; BioNano Genomics), based on the optically mapped molecules. The assembled PacBio scaffolds were input into hybridScaffold [24]. In brief, the hybrid scaffolding process included the alignment of the PacBio scaffolds against the BioNano genome maps, followed by the identification and resolution of conflicting alignments. At the end of stage two, the hybrid genome assembly had a scaffold N50 of 9.22 Mb (**Supplementary Table S4**).

In the third stage, the 10X genomic linked reads were connected with the scaffolds generated in stage two to construct super-scaffolds. In brief, we used the long ranger basic pipeline (https://support.10xgenomics.com/genome-exome/software/downloads/) to handle the basic read in and barcode processing of the 10X genomic linked reads. The processed 10X linked reads were then mapped to the hybrid genome assembly from stage two with bowtie2 [25], using the command "bowtie2 genome.fa -1 reads1.fq.gz -2 reads2.fq.gz -p 12 -D 1 -R 1 -N 0 -L 28 -i S,0,2.50 --n-ceil L,0,0.02 --rdg 5,10 --rfg 5,10).". We also used a self-against-self

(genome.fa-against-genome.fa) blastn to generate two bed files, and merged these files using fragScaff (version 140324.1) [26], with the parameters "-fs1 '-m 3000 -q 20 -E 30000 -o 60000', -fs2 '-C 2', -fs3 '-j 1.5 -u 2'.". These procedures generated an updated genome assembly with a scaffold N50 of 24.09 Mb (**Supplementary Table S5**). Subsequently, we corrected errors in the assembly, based on the Illumina short reads, using the Burrows-Wheeler Aligner (BWA, RRID:SCR_010910) [27] and Pilon-1.18 (Pilon, RRID:SCR_014731) [21].

In the fourth stage, the Hi-C data were used to build chromosome-level assembly scaffolds. In brief, Hi-C sequencing data were first aligned to the assembled genome using BWA [27]. Scaffolds were then clustered, ordered, and oriented using Lachesis [28], with the parameter set "CLUSTER_MIN_RE_SITES = 1800, CLUSTER_MAX_LINK_DENSITY = 4, and CLUSTER_NONINFORMATIVE_RATIO = 0." This procedure generated 22 accurately clustered and ordered pseudo-chromosomes, with a genome size of 3.04 Gb, a contig N50 of 5.72 Mb, and a scaffold N50 of 144.56 Mb (**Table 2**). The pseudo-chromosomes were divided into 100-kb bins and the interaction frequencies between pairs of 100-kb genomic regions were determined (**Fig. 2**).

Assessment of the genome newly assembled

We evaluated our newly assembled *R. roxellana* genome against the previously published assembly. The contiguity of our *R. roxellana* genome was 100-fold greater (contig N50: 5.72

Mb; scaffold N50: 144.56) than the previous version (contig N50: 25.5 kb; scaffold N50: 1.55 Mb) [8]. We also aligned our genome against the previous version using MUMMER (v4.0.0beta2) [29] and identified 6,452 gaps in the previous version that were predicted to be filled by >29.7 Mb of sequence in our new assembly. These filled gaps were mainly located in the intergenic and repetitive regions, with a small fraction of the sequence data annotated as gene regions. Our new assembly also had a higher proportion of repeat sequences (50.82%) as compared to the previous version (46.15%); in particular, the number of LINE (long interspersed elements) transposable elements and tandem repeats was greatly increased (further details are given below, in the "Identification of repeat elements" section). Thus, the newly assembled genome was substantially more complete and continuous. It was likely that the remarkable improvement in contiguity was due to the increased read length, deeper sequencing depth, improved gap assembly, and more sophisticated assembly algorithm.

To assess the accuracy of our genome assembly, we aligned the Illumina short reads to the assembly using BWA [27], with the parameters "-o 1 -i 15". Approximately 99.17% of the short reads were mapped to the genome assembly. Further investigations indicated that these reads covered approximately 99.27% of the total assembly (**Supplementary Table S6**). Genome assembly accuracy was also measured using the standard variant calling method in samtools (http://samtools.sourceforge.net/), with the command "samtools mpileup -q 20 -Q 20 -C 50 -uDEf." We found that the homozygous non-reference SNPs (single nucleotide

polymorphism) comprised 0.0004% of all SNPs (7,690 of 559,048), suggesting that our genome assembly was highly accurate (**Supplementary Table S7**). In addition, we estimated assembly completeness using Benchmarking Universal Single-copy Orthologs (BUSCO, RRID:SCR_015008) v3.0.2 [30], with the parameters "-i -o -l -m genome -f -t." based on mammalia_odb9 (creation date: 2016-02-13; number of species: 50; number of BUSCOs: 4,104). BUSCO analysis identified 4,104 mammalian BUSCOs in the newly assembled *R. roxellana* genome: 94.0% complete BUSCOs, 2.9% fragmented BUSCOs, and 3.1% missing BUSCOs (**Supplementary Table S8**). Assembly completeness was also measured using the core eukaryotic gene (CEG)-mapping approach (CEGMA v2.5) [31]. Of the 248 CEGs known from six model species, 93.95% (233 of 248) were identified in our new genome assembly. Of these, 220 CEGs were complete and unfragmented, and the remaining 13 were complete but fragmented (**Supplementary Table S9**). Together, these analyses indicated that our new genome assembly was highly accurate and complete.

Identification of repeat elements

Repeat sequences account for a large proportion of the total genome. It is thus important to identify repeat elements. Here, we predicted and classified repeat elements both based on homology and *de novo*. In the homology approach, we searched the genome for repetitive DNA elements (as listed in the Repbase database v16.02) using RepeatMasker v4.0.6 (RepeatMasker,

RRID:SCR_012954, http://www.repeatmasker.org/) [32] with the parameters "-a -nolow -no_is -norna -parallel 1" and using RepeatProteinMask (implemented in RepeatMasker). To identify repetitive elements *de novo*, we used RepeatModeler v1.0.11 (RepeatModeler, RRID:SCR_015027) [33], with the parameters "-database genome -engine ncbi -pa 15)." Tandem repeats in the genome were detected using Tandem Repeat Finder (TRF) v4.07b [34], with parameters "2 7 7 80 10 50 2000 -d -h"). We merged the results of the two methods. In total, the new genome assembly comprised 50.81% repetitive sequences (**Supplementary Table S10)**. Closer investigation indicated that the largest categories of repeat elements in the *R. roxellana* genome were the short and long interspersed nuclear elements (SINEs and LINEs, respectively). In addition, several repeat elements absent from Repbase database were detected in the *de novo* approach (**Supplementary Table S10**). The total length of these repeat elements was 186,195,432bp, accounting for 6.13% of the genome, suggesting that these repeat elements may be specific for *R. roxellana*. Compared with the repeat sequences in the previous assembly, our genome included relatively more LINE transposable elements (28.23% vs. 6.21%) and tandem repeats (6.20% vs. 2.82%). The detailed categories of repeat elements are summarized in **Supplementary Table S11**.

Duplicate sequences identification

We also performed duplicate sequences identification analysis, which was fulfilled based on the read depth of Illumina short reads. In brief, we first mapped the Illumina short reads to

the assembled genome using BWA with default parameters. Then, the sorted mapping bam file was used as input for CNVnator v0.3.3 [35], a tool targeting alterations in the read depth, with the parameters of "-unique -his 100 -stat 100 -call 100.". The obtained duplicate sequences were filtered, retaining only those where q0 was <0.5 and e-val1 was <0.05. After filtering, 676 duplicate sequences remained, with a total length of 9,198,900 bp **(Supplementary Table S12)**. Further analysis showed that 101 duplications located at the end of scaffolds (5% of the total length in both ends). And there were 136 genes present in the duplicated regions, these genes mainly involved in basic biological processes such as ribonucleoside binding, phosphatase activity, and protein dephosphorylation.

Non-coding RNA prediction

Non-coding RNAs included ribosomal RNAs (rRNAs), transfer RNAs (tRNAs), microRNAs (miRNAs), and small nuclear RNAs (snRNAs). Non-coding RNAs primarily regulate biological processes. Using BLASTN (BLASTN, RRID:SCR_001598) with an E-value of 1E-10, we identified four rRNAs in the *R. roxellana* genome homologous to human rRNAs: 28S, 18S, 5.8S, and 5S (GenBank accession numbers NR_003287.2, NR_003286.2, NR_003285.2, and NR_023363.1, respectively). We also searched for miRNAs and snRNAs in the new genome using INFERNAL v1.1rc4 (Infernal, RRID:SCR_011809) [36] against the Rfam database release 13.0 [37]. The tRNAs were predicted by tRNAscan-SE 1.3.1

(tRNAscan-SE, RRID:SCR_010835) [38]. We identified 608 rRNAs, 17,813 miRNAs, 3,656 snRNAs, and 460 tRNAs in the *R. roxellana* genome (**Supplementary Table S13**).

Gene prediction and functional annotation

We predicted genes using a combination of approaches: *de novo*, homology prediction, and transcriptome. For *ab initio* predictions of protein-coding genes, we used Augustus v3.2.2 (Augustus, RRID:SCR_008417) [39], with parameters "--uniqueGeneId = true –noInFrameStop = true --gff3 = on –genemodel = complete –strand = both"; GlimmeHMM v3.0.1 [40], with parameters "-g -f"; GENSCAN (GENSCAN, RRID:SCR_012902) [41], GENEID [42], and SNAP v2013-11-29 [43].

Next, we predicted genes using homology-based approach. Protein sequences from five homologous species (*Homo sapiens, Gorilla gorilla, Macaca mulatta, Rhinopithecus bieti, and Rhinopithecus roxellana hubeiensis) were down*loaded from Ensemble Release 75 (http://www.ensembl.org/info/data/ftp/index.html). We compared these sequences to the repeat-masked *R. roxellana* genome using TBLASTN (TBLASTN, RRID:SCR_011822, -p tblastn -e 1e-05 -F T -m 8 -d) against the repeat-masked genome sequences [44], with parameters "-p tblastn -e 1e-05 -F T -m 8 -d." The identified homologous genome sequences were annotated using GeneWise (Version 2.4.1, GeneWise, RRID:SCR_015054) [45], with the parameters "-tfor -genesf -gff."

Finally, we estimated genes based on transcriptome data. High-quality RNAs from the heart and skin tissue of the *R. roxellana qinlingensis* specimen (the same individual used for DNA sequencing and reference assembly) were sequenced on an Illumina Novaseq 6000 platform. RNA-seq reads were assembled using trinityrnaseq-2.1.1 [46], with the parameters "--seqType fq --CPU 20 --max_memory 200G --normalize_reads --full_cleanup --min_glue 2 --min_kmer_cov 2 --KMER_SIZE 25." To identify validate transcripts, the assembled transcript sequences were aligned to the *R. roxellana* genome using Assemble Spliced Alignment (PASA) [47], with default parameters. We estimated transcript expression levels using Tophat 2.0.13 (TopHat, RRID:SCR_013035) [48] (with the parameters "-p 6 --max-intron-length 500000 -m 2 --library-type fr-unstranded") and Cufflinks (Cufflinks, RRID:SCR_014597) [49].

The genes predicted by each of the three approaches were merged using EVidenceModeler (EVidenceModeler, RRID:SCR_014659) [50] with the parameters "--segmentSize 200000 --overlapSize 20000." We weighted transcript predictions most highly, followed by homology-based predictions and *ab initio* predictions. Untranslated regions and alternative splicing of the predicted gene were explored using PASA, in conjunction with the transcriptome data [47]. In total, 22,497 genes were predicted in the *R. roxellana* genome (**Table 3**), each containing an average of 7.71 exons. The detailed results of the gene prediction process are given in **Table 3** and **Fig. 3**.

We also compared the gene structure, including mRNA length, exon length, intron length, and exon number, among *R. roxellana* and other representative primates (e.g., *Homo sapiens, Gorilla gorilla*, *Macaca mulatta*, *Rhinopithecus bieti*, *and Rhinopithecus roxellana hubeiensis*). We found that genome assembly patterns were similar among *R. roxellana* and the other primates **(Supplementary Fig. S2)**.

To better understand the biological functions of the predicted genes, we used BLASTP (BLASTP, RRID:SCR_001010, with an E-value of 1E-5) to identify the best match for each predicted gene across several databases, including the NCBI nonredundant protein database (NR v20180129), SwissProt (v20150821) [51], Kyoto Encyclopedia of Genes and Genomes (KEGG v20160503) [52], InterPro v29.0 (InterPro, RRID:SCR_006695) [53], Pfam v31.0 (Pfam, RRID:SCR_004726) [54], and GO (Gene Ontology)[55]. In this way, 22,053 predicted genes (98.42%) were functionally annotated (**Supplementary Table S14**). Nearly half (10,670 of 22,497) of these genes were annotated to the predicted proteins in NR database derived from the previous genome annotation for *Rhinopithecus roxellana*.

In addition, we estimated the genome assembly completeness using transcriptome data. The transcripts were derived from the *de novo* assembly with trinityrnaseq-2.1.1 mentioned above. Those transcripts were clustered into unigenes with the help of using TGICL (TIGR gene indices clustering program, v2.1) [56] with 95% identity similarity cut-off. The generated unigenes were aligned to our assembly version and previous version using BLAT v. 36 (BLAT,

RRID:SCR_011919). Results showed that the completeness degree (percentage of unigenes aligned to a single scaffold in genome) was higher in our assembly (95.35%) compared with that in previous assembly (89.28%) for unigenes larger than 1000 bp (**Supplementary Table S15**), demonstrating the contiguity of our new assembly.

Phylogenetic analysis and gene family estimation

The coding regions and protein sequences of 11 representative mammals were downloaded from Ensembl (Ensembl Release 75). For genes with multiple transcript isoforms, the longest was chosen. Treefam [57] was used to estimate gene families. Using an all-to-all blast, we identified 17,560 gene families. We reconstructed the phylogenetic relationships among *R. roxellana* and other mammals based on four-fold degenerate sites extracted from the 5,418 single-copy gene families. Phyml v3.2 (PhyML, RRID:SCR_014629) [58] was used to construct a maximum-likelihood tree using the GTR + gamma model, as inferred by JMODELTEST v2.1.10 (jModelTest, RRID:SCR_015244) [59]. We estimated divergence times with MCMCTREE in PAML v4.8 (PAML, RRID:SCR_014932) [60], using the Bayesian method and the fossil calibration times from timetree (http://www.timetree.org/) [61]. The following fossil calibrations were used: *H. sapiens* vs. *Callithrix jacchus* (40.6–45.7 MYA, million years ago); *Homo sapiens* vs. *Pan troglodytes* (~6.2–7 MYA); *Homo sapiens* vs. *Mus musculus* (85–94 MYA); and *Homo sapiens* vs. *Tarsius syrichta* (~71–77 MYA). The

reconstructed phylogeny recovered a close relationship between *R. rollexana* and *M. mulatta*. We estimated that *R. rollexana* and *M. mulatta* diverged approximately 13.4 MYA (**Fig. 4**).

To investigate the evolutionary history of *R. r.*, we estimated the expansion and contraction of gene family in this species with CAFE 3.0 (CAFÉ, RRID:SCR_005983) [62]. A random birth and death model was used to study gene family variations along each lineage in the phylogenetic tree. This analysis identified 993 expanded gene families and 2,745 contracted gene families in the *R. roxellana* genome (**Fig. 4**). To determine the significance of each gene family, *P*-values in each lineage were estimated by comparing conditional likelihoods derived from a probabilistic graphical model (PGM). All gene family with *P*-values < 0.05 were further analyzed. To explore the significantly expanded gene families, we performed a GO-term enrichment analysis with EnrichPipeline32 [63, 64], using the 1,370 genes belonging to the 314 significantly expanded gene families as input, and using all predicted genes as background. We considered GO term significant if adjusted the *P*-value was <0.05. We found that the significantly expanded gene families were mainly associated with the hemoglobin complex, energy metabolism, and oxygen transport (**Supplementary Table S16**).

Conclusion

In this study, we generated a high-quality genome assembly for the golden snub-nosed monkey (*R. roxellana*) using a combination of five advanced genomics technologies. Our

results will inform studies of the origin and evolutionary history of the snub-nosed monkey. In addition, this genome may provide a framework within which to survey the mechanisms underlying the formation of the distinct morphological and sociological characters of *R. roxellana*. This genome may also stimulate new insights into the improvement of strategies to conserve and manage this endangered species. Finally, this genome, which has superior continuity and accuracy, may serve as a new standard reference genome for colobine primates.

Declarations

Availability of supporting data

The raw data discussed in this publication have been deposited in NCBI's short read archive under the accession number PRJNA524949. All supporting data and materials and a JBrowse genome browser are available in the *GigaScience* GigaDB database [65].

Competing interests

The authors declare that they have no competing interests.

Funding

This work was financially supported by Strategic Priority Research Program of the Chinese Academy of Sciences (XDB31020302), the National Natural Science Foundation of China (31622053), the Promotional project for Innovation team, the Department of Science and Technology of Shaanxi Prov. China (2018TD-017), and the National Key Programme of

Research and Development, the Ministry of Science and Technology of China (2016YFC0503200).

Abbreviations

Gb: gigabase; kb: kilobase; Mb: megabase; PE: paired-end; PacBio: Pacfic Biosciences; SMRT: single molecule real-time sequencing; Hi-C: high-throughput chromosome conformation capture; BUSCO: Benchmarking Universal Single-copy Orthologs; GEGMA: core eukaryotic gene-mapping approach; GO: gene ontology; TFS: transposable element; TRF: Tandem Repeat Finder; SINEs: Short interspersed nuclear elements; LINEs: long interspersed nuclear elements; PASA: genome by Assemble Spliced Alignment; NR: NCBI nonredundant protein database; KEGG: Kyoto Encyclopedia of Genes and Genomes. Mya: million years ago.

Author contributions

X.G.Q. conceived and designed the project, L.W., J.W.W. contributed to the work on genomic sequencing and performing data analyses. J.W.W., L.W. and X.G.Q. wrote the manuscript. B.G.L. helped with sample collection. All authors provided input for the paper and approved the final version.

Acknowledgements

We thank Mr. Yiliang Xu, Mr. Qiqi Liang, Mrs. Yue Xie from Novogene for their technical support. Mr. Xuanmin Guang and Mr. Chi Zhang from BGI for their assistance in data analysis. We thank to Mr. Ruliang Pan for his gracious help polishing the language. We are also grateful to Mr. Yinghu Lei from Louguantai Breeding Center, Dr. Zhipang Huang, and Dr. Pei Zhang from Northwest University for their helping with the sampling collection. We specially appreciate Prof. Zhengbing Wang, and Prof. Jiang Chang from Discipline Development Department of Northwest University for their support. This study was supported by through the Discipline Construction Project of Northwest University.

Figures and tables

Figure legends:

Fig. 1. Image of *R. roxellana,* taken in the Qinling Mountain, China.

Fig. 2. Hi-C heatmap of interactions between pairs of chromosomal loci throughout the genome. Hi-C interactions within and among *R. roxellana* chromosomes (Chr 1– Chr 22); interactions were drawn based on the chromatin interaction frequencies between pairs of 100-kb genomic regions (as determined by Hi-C). In principle, darker red cells indicate stronger and more frequent interactions, which in turn imply that the two sequences are spatially close.

Fig. 3. Gene predictions. (a) Number of genes estimated by various prediction approaches: *de novo* (blue), homologys (pink), and RNA-seq data (green). The labels rna_0.5, denove_0.5,

and homology_0.5 indicate the genes predicted by each method with an overlap >50%. (b) Number of genes predicted based on *de novo*, homology, and RNA-seq approaches, in addition to expression level (in rpkm). The labels rna_0.5, denove_0.5, and homology_0.5 indicate the genes predicted by each method with an overlap >50%, while rpkm>1 indicates those genes with a relative expression level >1.

Fig. 4. *R. roxellana* phylogenetic relationships and gene families. Phylogenetic relationships were inferred from 5,418 single-copy gene families in *R. roxellana* and other mammals. All nodes had support values of 100%. Estimated divergence times are given near each node. Numbers under each species indicate the number of gene families that have been expanded (green) and contracted (light yellow) since the split of species from the most recent common ancestor (MRCA). The numbers on each branch correspond to the numbers of gene families that have been expanded (red) and contracted (green) in the mammalian genome. Those monkey images are copyright 2013 Stephen D. Nash of the IUCN SSC Primate Specialist Group and are used with permission. MYA: million years ago.

Table 1. Reads generated by the five sequencing methods.

Paired-end libraries	Insert size (bp)	Total clean data (Gb)	Read length (bp)	Sequence coverage (X)
Illumina	350	423.32	150	133.12
Pacbio	20 k	304.84	n/a	95.86
10X Genomics	500–700	348.41	150	109.56
BioNano	n/a	463.75	n/a	n/a
Hi-C	350	310.92	n/a	97.77
Total	n/a	1,851.24	n/a	582.15

Note: The sequence coverage was calculated based on an estimated genome size of 3.18 Gb.

n/a: not applicable.

Table 2. Summary of the final *R. roxellana* genome assembly.

Category	Contig		Scaffold	
	Length (bp)	Number	Length (bp)	Number
Total	3,038,184,325	6,099	3,038,467,325	3,269
Max	30,757,641	n/a	206,558,726	n/a
≥2000 bp	n/a	5,708	n/a	2,879
N50	5,723,610	151	144,559,847	9
N60	4,241,389	211	141,075,955	11
N70	3,173,235	292	135,203,321	14
N80	2,063,823	408	118,350,466	16
N90	896,517	622	83,045,532	19

Note: The "Number" column represents the number of contigs/scaffolds longer than the value of the corresponding category. n/a: not applicable.

Table 3. Summary and characteristics of the predicted protein-coding genes.

Gene set		Number	Average transcript length (bp)	Average CDS length (bp)	Average exon length (bp)	Average intron length (bp)	Average exons per gene
De novo	Augustus	32,928	23,441	1,052	196	5,112	5.38
	GlimmerHMM	618,957	4,204	404	166	2,654	2.43
	SNAP	97,298	49,851	755	144	1,1597	5.23
	Geneid	36,863	35,242	1,035	188	7,615	5.49
	Genscan	50,419	40,635	1,137	167	6,800	6.81
Homology	Ggo	25,281	19,893	1,055	184	3,971	5.74
	Hsa	38,444	14,763	826	182	3,942	4.54
	Mmu	21,959	29,709	1,470	187	4,123	7.85
	Rbi	25,320	25,685	1,387	196	3,991	7.09
	Rro	24,121	28,439	1,420	185	4,043	7.68
RNASeq	PASA	66,620	28,449	1,219	164	4,247	7.41
	Cufflinks	73,199	31,497	2,737	409	5,052	6.69
EVM		30,102	22,298	1,098	182	4,199	6.05
Pasa-update*		29,403	27,638	1,180	181	4,782	6.53
Final set*		22,497	34,153	1,369	178	4,885	7.71

Note: Pasa-update* includes only the untranslated regions; other regions were not included. Final set* represents the results after the Pasa filtering process, where the longest isoform was chosen if the case of multiple splicing isoforms; redundant single exons were also discarded. The "Number" column gives the number of protein-coding genes predicted by each method.

Supplementary files:

Supplementary Fig. S1. Genome size estimation using the k-mer method.

Supplementary Fig. S2. Comparisons of each element among genomes of homologous species.

Supplementary Table S1. The contig assembly based on PacBio subreads.

Supplementary Table S2. The scaffold assembly based on sspace-longreads results.

Supplementary Table S3. The assembly after gap-filling.

Supplementary Table S4. The assembly based on BioNano optical map data.

Supplementary Table S5. The assembly based on 10X Genomics linked reads.

Supplementary Table S6. The read mapping rate and the coverage of the assembled genome determined with BWA.

Supplementary Table S7. The SNPs identified in the genome of *R. roxellana*.

Supplementary Table S8. Genome assessment based on BUSCO annotations.

Supplementary Table S9. Genome assessment based on CEGMA annotations.

Supplementary Table S10. Prediction of repeat elements prediction in the genome assembly.

Supplementary Table S11. Prediction of repetitive sequences in the genome assembly.

Supplementary Table S12. The duplicated sequences (DS) identified in the genome assembly.

Supplementary Table S13. Summary and characteristics of the predicted RNAs.

Supplementary Table S14. The functional annotations of the genes predicted in the *R. roxellana* genome.

Supplementary Table S15. Assessment of the new genome assembly using unigene sequences

Supplementary Table S16. The GO annotations of the expanded gene families in the *R. roxellana* genome (adjusted *P*-value < 0.05)

References

1. Li BG, Pan RL, Oxnard CE. Extinction of snub-nosed monkeys in China during the past 400 years. Int J Primatol, 2002; **23**(6):1227-1244.
2. Luo MF, Liu ZJ, Pan HJ, Zhao L, Li M. Historical geographic dispersal of the golden snub-nosed monkey (*Rhinopithecus roxellana*) and the influence of climatic oscillations. Am J Primatol, 2012; **74**(2):91-101.
3. Fang G, Li M, Liu X-J, Guo W-J, Jiang Y-T, Huang Z-P, Tang S-Y, Li D-Y, Yu J, Jin T *et al*. Preliminary report on Sichuan golden snub-nosed monkeys (*Rhinopithecus roxellana roxellana*) at Laohegou Nature Reserve, Sichuan, China. Sci Rep, 2018; **8**(1):16183.
4. Grueter CC, Qi X, Li B, Li M. Multilevel societies. Curr Biol, 2017; **27**(18):R984-R986.
5. Qi XG, Li BG, Garber PA, Ji WH, Watanabe K. Social dynamics of the golden snub-nosed monkey (*Rhinopithecus roxellana*): female transfer and one-male unit succession. Am J Primatol, 2009; **71**(8):670-679.
6. Li H, Meng S-J, Men Z-M, Fu Y-X, Zhang Y-P. Genetic diversity and population history of golden monkeys (*Rhinopithecus roxellana*). Genetics, 2003; **164**(1):269-275.
7. Qi X-G, Garber PA, Ji W, Huang Z-P, Huang K, Zhang P, Guo S-T, Wang X-W, He G, Zhang P *et al*. Satellite telemetry and social modeling offer new insights into the origin of primate multilevel societies. Nat Commun, 2014; **5**:5296.

8. Zhou X, Wang B, Pan Q, Zhang J, Kumar S, Sun X, Liu Z, Pan H, Lin Y, Liu G *et al*. Whole-genome sequencing of the snub-nosed monkey provides insights into folivory and evolutionary history. Nat Genet, 2014; **46**:1303-1310.
9. Kuang W-M, Ming C, Li H-P, Wu H, Frantz L, Roos C, Zhang Y-P, Zhang C-L, Jia T, Yang J-Y *et al*. The origin and population history of the endangered golden snub-nosed monkey (*Rhinopithecus roxellana*). Mol Biol Evol, 2018:msy220-msy220.
10. Hong YY, Duo HR, Hong JY, Yang JY, Liu SM, Yu LH, Yi TY. Resequencing and comparison of whole mitochondrial genome to gain insight into the evolutionary status of the Shennongjia golden snub-nosed monkey (SNJ *R-roxellana*). Ecol Evol, 2017; **7**(12):4456-4464.
11. Seo JS, Rhie A, Kim J, Lee S, Sohn MH, Kim CU, Hastie A, Cao H, Yun JY, Kim J *et al*. De novo assembly and phasing of a Korean human genome. Nature, 2016; **538**(7624):243-247.
12. Chaisson MJ, Wilson RK, Eichler EE. Genetic variation and the de novo assembly of human genomes. Nat Rev Genet, 2015; **16**(11):627-640.
13. Jiao YP, Peluso P, Shi JH, Liang T, Stitzer MC, Wang B, Campbell MS, Stein JC, Wei XH, Chin CS *et al*. Improved maize reference genome with single-molecule technologies. Nature, 2017; **546**(7659):524-527.
14. Gordon D, Huddleston J, Chaisson MJP, Hill CM, Kronenberg ZN, Munson KM, Malig M, Raja A, Fiddes I, Hillier LW *et al*. Long-read sequence assembly of the gorilla genome. Science, 2016; **352**(6281):aae0344.
15. Kronenberg ZN, Fiddes IT, Gordon D, Murali S, Cantsilieris S, Meyerson OS, Underwood JG, Nelson BJ, Chaisson MJP, Dougherty ML *et al*. High-resolution comparative analysis of great ape genomes. Science, 2018; **360**(6393):eaar6343.
16. Bickhart DM, Rosen BD, Koren S, Sayre BL, Hastie AR, Chan S, Lee J, Lam ET, Liachko I, Sullivan ST *et al*. Single-molecule sequencing and chromatin conformation capture enable de novo reference assembly of the domestic goat genome. Nat Genet, 2017; **49**:643-650.
17. Luo R, Liu B, Xie Y, Li Z, Huang W, Yuan J, He G, Chen Y, Pan Q, Liu Y *et al*. SOAPdenovo2: an empirically improved memory-efficient short-read de novo assembler. Gigascience, 2012; **1**(1):18.
18. Martin M. Cutadapt removes adapter sequences from high-throughput sequencing reads. EMBnetjournal, 2011; **17**:10 -12.
19. Chin CS, Peluso P, Sedlazeck FJ. Phased diploid genome assembly with single-molecule real-time sequencing. Nat Methods, 2016; **13**(12):1050-1054.
20. Chin CS, Alexander DH, Marks P, Klammer AA, Drake J, Heiner C, Clum A, Copeland A, Huddleston J, Eichler EE *et al*. Nonhybrid, finished microbial genome assemblies from long-read SMRT sequencing data. Nat Methods, 2013; **10**(6):563-+.

21. Walker BJ, Abeel T, Shea T, Priest M, Abouelliel A, Sakthikumar S, Cuomo CA, Zeng QD, Wortman J, Young SK *et al*. Pilon: An Integrated Tool for Comprehensive Microbial Variant Detection and Genome Assembly Improvement. Plos One, 2014; **9**(11).

22. Boetzer M, Pirovano W. SSPACE-LongRead: scaffolding bacterial draft genomes using long read sequence information. BMC Bioinformatics, 2014; **15**:211.

23. English AC, Richards S, Han Y, Wang M, Vee V, Qu J, Qin X, Muzny DM, Reid JG, Worley KC *et al*. Mind the gap: upgrading genomes with Pacific Biosciences RS long-read sequencing technology. Plos One, 2012; **7**(11):e47768.

24. Shelton JM, Coleman MC, Herndon N, Lu N, Lam ET, Anantharaman T, Sheth P, Brown SJ. Tools and pipelines for BioNano data: molecule assembly pipeline and FASTA super scaffolding tool. BMC Genomics, 2015; **16**:734-734.

25. Langmead B, Trapnell C, Pop M, Salzberg SL. Ultrafast and memory-efficient alignment of short DNA sequences to the human genome. Genome Biol, 2009; **10**(3):R25.

26. Adey A, Kitzman JO, Burton JN, Daza R, Kumar A, Christiansen L, Ronaghi M, Amini S, Gunderson KL, Steemers FJ *et al*. In vitro, long-range sequence information for de novo genome assembly via transposase contiguity. Genome Res, 2014; **24**(12):2041-2049.

27. Li H, Durbin R. Fast and accurate short read alignment with Burrows-Wheeler transform. Bioinformatics, 2009; **25**(14):1754-1760.

28. Burton JN, Adey A, Patwardhan RP, Qiu R, Kitzman JO, Shendure J. Chromosome-scale scaffolding of de novo genome assemblies based on chromatin interactions. Nat Biotechnol, 2013; **31**(12):1119-1125.

29. Kurtz S, Phillippy A, Delcher AL, Smoot M, Shumway M, Antonescu C, Salzberg SL. Versatile and open software for comparing large genomes. Genome Biol, 2004; **5**(2):R12.

30. Simao FA, Waterhouse RM, Ioannidis P, Kriventseva EV, Zdobnov EM. BUSCO: assessing genome assembly and annotation completeness with single-copy orthologs. Bioinformatics, 2015; **31**(19):3210-3212.

31. Parra G, Bradnam K, Korf I. CEGMA: a pipeline to accurately annotate core genes in eukaryotic genomes. Bioinformatics, 2007; **23**(9):1061-1067.

32. Jurka J, Kapitonov VV, Pavlicek A, Klonowski P, Kohany O, Walichiewicz J. Repbase Update, a database of eukaryotic repetitive elements. Cytogenet Genome Res, 2005; **110**(1-4):462-467.

33. Price AL, Jones NC, Pevzner PA. De novo identification of repeat families in large genomes. Bioinformatics, 2005; **21 Suppl 1**:i351-358.

34. Benson G. Tandem repeats finder: a program to analyze DNA sequences. Nucleic Acids Res, 1999; **27**(2):573-580.
35. Abyzov A, Urban AE, Snyder M, Gerstein M. CNVnator: an approach to discover, genotype, and characterize typical and atypical CNVs from family and population genome sequencing. Genome Res, 2011; **21**(6):974-984.
36. Nawrocki EP, Kolbe DL, Eddy SR. Infernal 1.0: inference of RNA alignments (vol 25, pg 1335, 2009). Bioinformatics, 2009; **25**(13):1713-1713.
37. Griffiths-Jones S, Moxon S, Marshall M, Khanna A, Eddy SR, Bateman A. Rfam: annotating non-coding RNAs in complete genomes. Nucleic Acids Res, 2005; **33**:D121-D124.
38. Lowe TM, Eddy SR. tRNAscan-SE: a program for improved detection of transfer RNA genes in genomic sequence. Nucleic Acids Res, 1997; **25**(5):955-964.
39. Stanke M, Keller O, Gunduz I, Hayes A, Waack S, Morgenstern B. AUGUSTUS: ab initio prediction of alternative transcripts. Nucleic Acids Res, 2006; **34**:W435-W439.
40. Majoros WH, Pertea M, Salzberg SL. TigrScan and GlimmerHMM: two open source ab initio eukaryotic gene-finders. Bioinformatics, 2004; **20**(16):2878-2879.
41. Burge C, Karlin S. Prediction of complete gene structures in human genomic DNA. J Mol Biol, 1997; **268**(1):78-94.
42. Guigo R. Assembling genes from predicted exons in linear time with dynamic programming. J Comput Biol, 1998; **5**(4):681-702.
43. Korf I. Gene finding in novel genomes. BMC Bioinformatics, 2004; **5**:59.
44. Kent WJ. BLAT - The BLAST-like alignment tool. Genome Res, 2002; **12**(4):656-664.
45. Birney E, Clamp M, Durbin R. GeneWise and Genomewise. Genome Res, 2004; **14**(5):988-995.
46. Grabherr MG, Haas BJ, Yassour M, Levin JZ, Thompson DA, Amit I, Adiconis X, Fan L, Raychowdhury R, Zeng QD *et al*. Full-length transcriptome assembly from RNA-Seq data without a reference genome. Nat Biotechnol, 2011; **29**(7):644-U130.
47. Haas BJ, Delcher AL, Mount SM, Wortman JR, Smith RK, Hannick LI, Maiti R, Ronning CM, Rusch DB, Town CD *et al*. Improving the Arabidopsis genome annotation using maximal transcript alignment assemblies. Nucleic Acids Res, 2003; **31**(19):5654-5666.
48. Kim D, Pertea G, Trapnell C, Pimentel H, Kelley R, Salzberg SL. TopHat2: accurate alignment of transcriptomes in the presence of insertions, deletions and gene fusions. Genome Biol, 2013; **14**(4):R36.
49. Trapnell C, Roberts A, Goff L, Pertea G, Kim D, Kelley DR, Pimentel H, Salzberg SL, Rinn JL, Pachter L. Differential gene and transcript expression analysis of RNA-seq experiments with TopHat and Cufflinks. Nat Protoc, 2012; **7**(3):562-578.

50. Haas BJ, Salzberg SL, Zhu W, Pertea M, Allen JE, Orvis J, White O, Buell CR, Wortman JR. Automated eukaryotic gene structure annotation using EVidenceModeler and the program to assemble spliced alignments. Genome Biol, 2008; **9**(1):R7.
51. Bairoch A, Apweiler R, Wu CH, Barker WC, Boeckmann B, Ferro S, Gasteiger E, Huang H, Lopez R, Magrane M *et al*. The Universal Protein Resource (UniProt). Nucleic Acids Res, 2005; **33**(Database issue):D154-D159.
52. Kanehisa M, Goto S. KEGG: kyoto encyclopedia of genes and genomes. Nucleic Acids Res, 2000; **28**(1):27-30.
53. Mitchell AL, Attwood TK, Babbitt PC, Blum M, Bork P, Bridge A, Brown SD, Chang HY, El-Gebali S, Fraser MI *et al*. InterPro in 2019: improving coverage, classification and access to protein sequence annotations. Nucleic Acids Res, 2019; **47**(D1):D351-D360.
54. Finn RD, Coggill P, Eberhardt RY, Eddy SR, Mistry J, Mitchell AL, Potter SC, Punta M, Qureshi M, Sangrador-Vegas A *et al*. The Pfam protein families database: towards a more sustainable future. Nucleic Acids Res, 2016; **44**(D1):D279-D285.
55. Ashburner M, Ball CA, Blake JA, Botstein D, Butler H, Cherry JM, Davis AP, Dolinski K, Dwight SS, Eppig JT *et al*. Gene Ontology: tool for the unification of biology. Nat Genet, 2000; **25**(1):25-29.
56. Pertea G, Huang X, Liang F, Antonescu V, Sultana R, Karamycheva S, Lee Y, White J, Cheung F, Parvizi B *et al*. TIGR Gene Indices clustering tools (TGICL): a software system for fast clustering of large EST datasets. Bioinformatics, 2003; **19**(5):651-652.
57. Li H, Coghlan A, Ruan J, Coin LJ, Heriche JK, Osmotherly L, Li RQ, Liu T, Zhang Z, Bolund L *et al*. TreeFam: a curated database of phylogenetic trees of animal gene families. Nucleic Acids Res, 2006; **34**:D572-D580.
58. Guindon S, Delsuc F, Dufayard J-F, Gascuel O: **Estimating maximum likelihood phylogenies with PhyML**. In: *Bioinformatics for DNA sequence analysis.* Springer; 2009: 113-137.
59. Posada D. jModelTest: phylogenetic model averaging. Mol Biol Evol, 2008; **25**(7):1253-1256.
60. Yang Z. PAML 4: Phylogenetic Analysis by Maximum Likelihood. Mol Biol Evol, 2007; **24**(8):1586-1591.
61. Kumar S, Stecher G, Suleski M, Hedges SB. TimeTree: A resource for timelines, timetrees, and divergence times. Mol Biol Evol, 2017; **34**(7):1812-1819.
62. De Bie T, Cristianini N, Demuth JP, Hahn MW. CAFE: a computational tool for the study of gene family evolution. Bioinformatics, 2006; **22**(10):1269-1271.

63. Huang da W, Sherman BT, Lempicki RA. Bioinformatics enrichment tools: paths toward the comprehensive functional analysis of large gene lists. Nucleic Acids Res, 2009; **37**(1):1-13.

64. Beissbarth T, Speed TP. GOstat: find statistically overrepresented Gene Ontologies within a group of genes. Bioinformatics, 2004; **20**(9):1464-1465.

65. Wang L; Wu J; Liu X; Di D; Liang Y; Feng Y; Zhang S; Li B; Qi X (2019): Supporting data for "A high-quality genome assembly of the endangered golden snub-nosed monkey Rhinopithecus roxellana" GigaScience Database. http://dx.doi.org/10.5524/100619.

Figure 1

Click here to access/download;Figure;fig 1_monkey.JPG

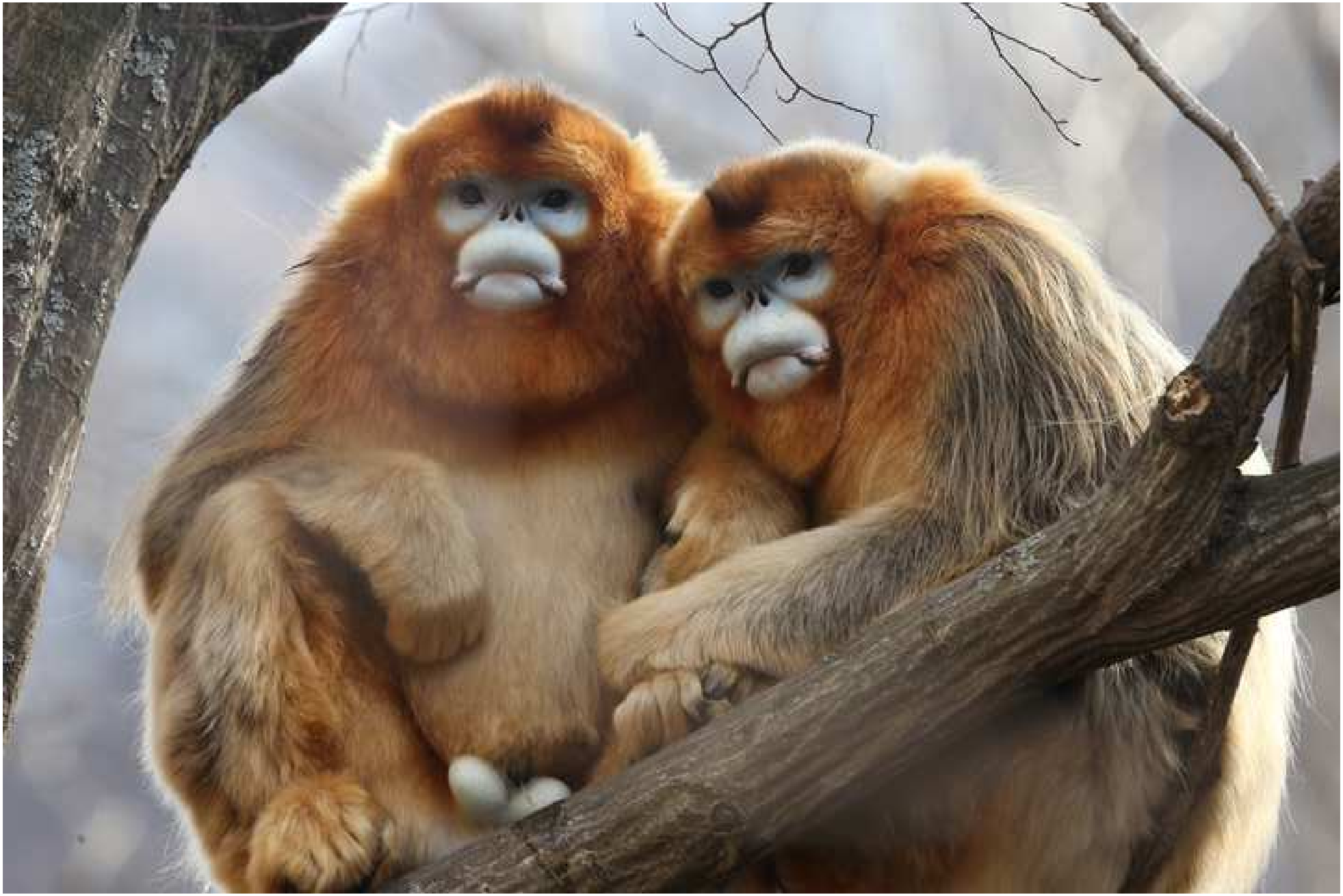

Figure 2

Click here to access/download;Figure;fig 2_Hic-compressed.pdf

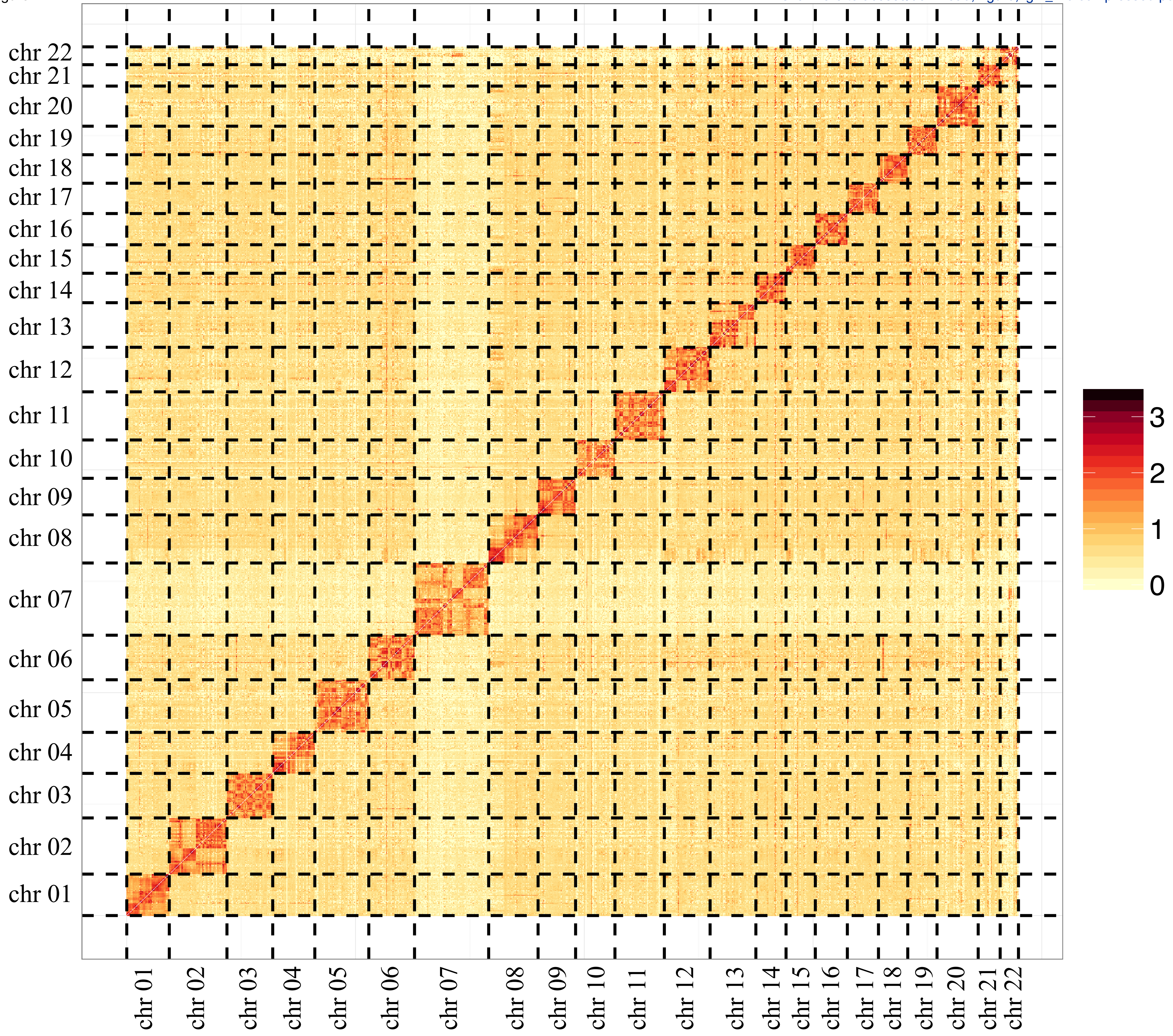

 Click here to access/download;Figure;fig 3.pdf

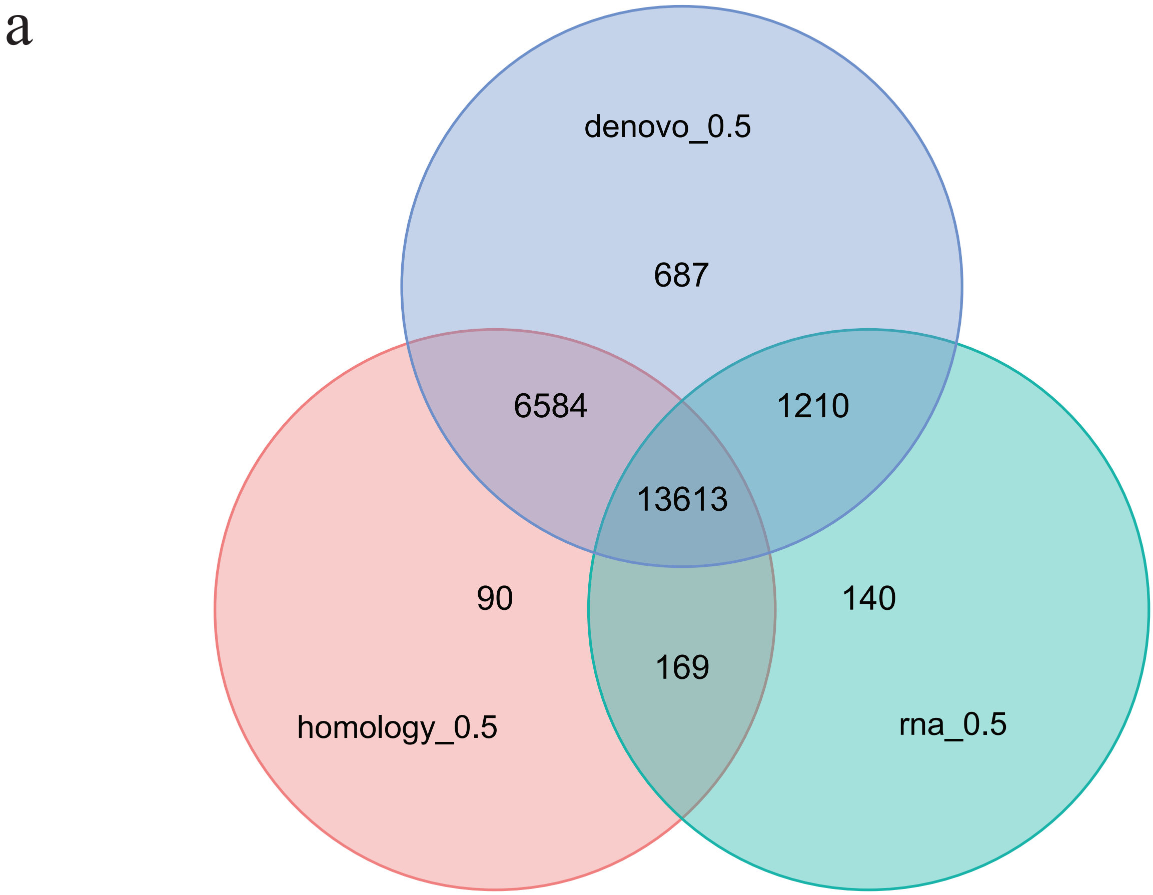

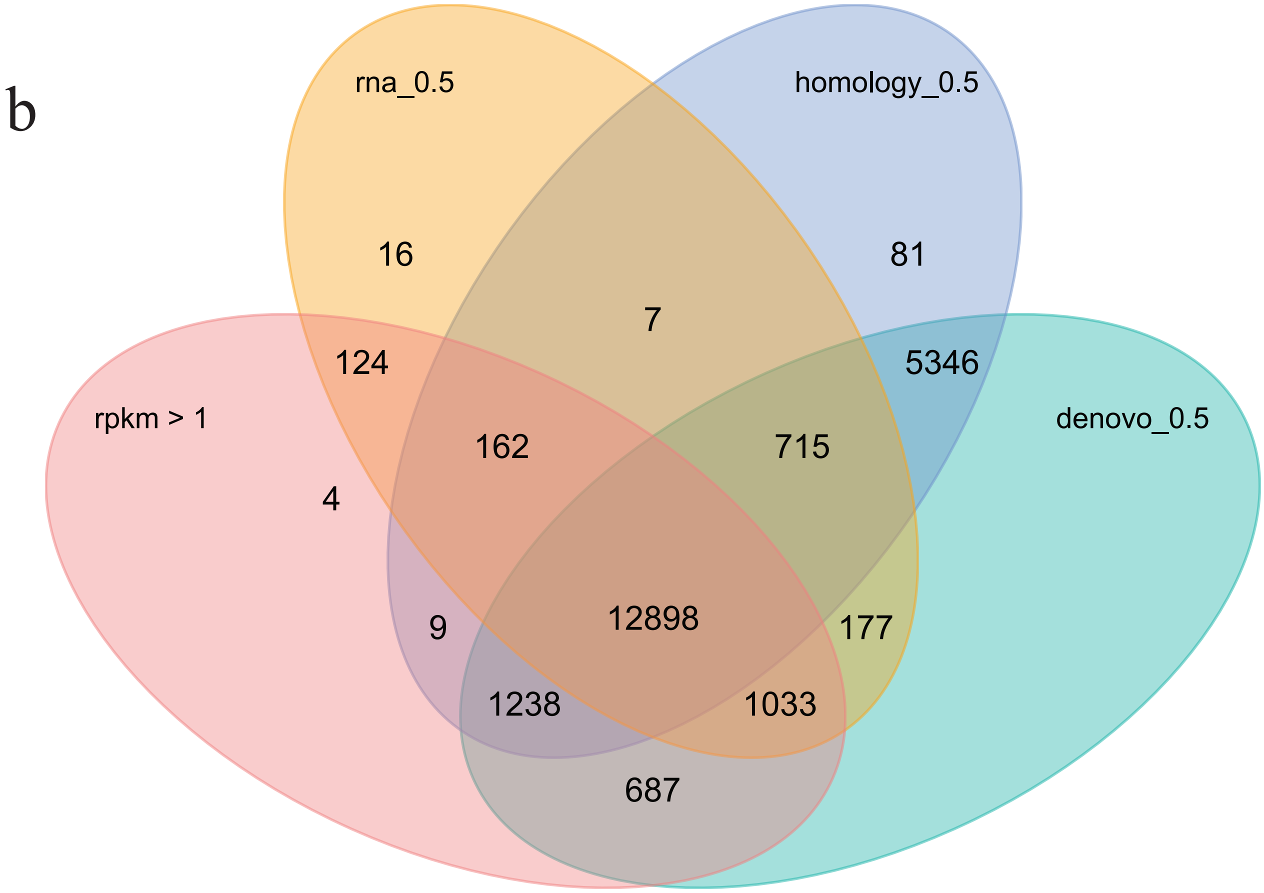

Figure 4

Click here to access/download;Figure;fig 4-compressed.pdf

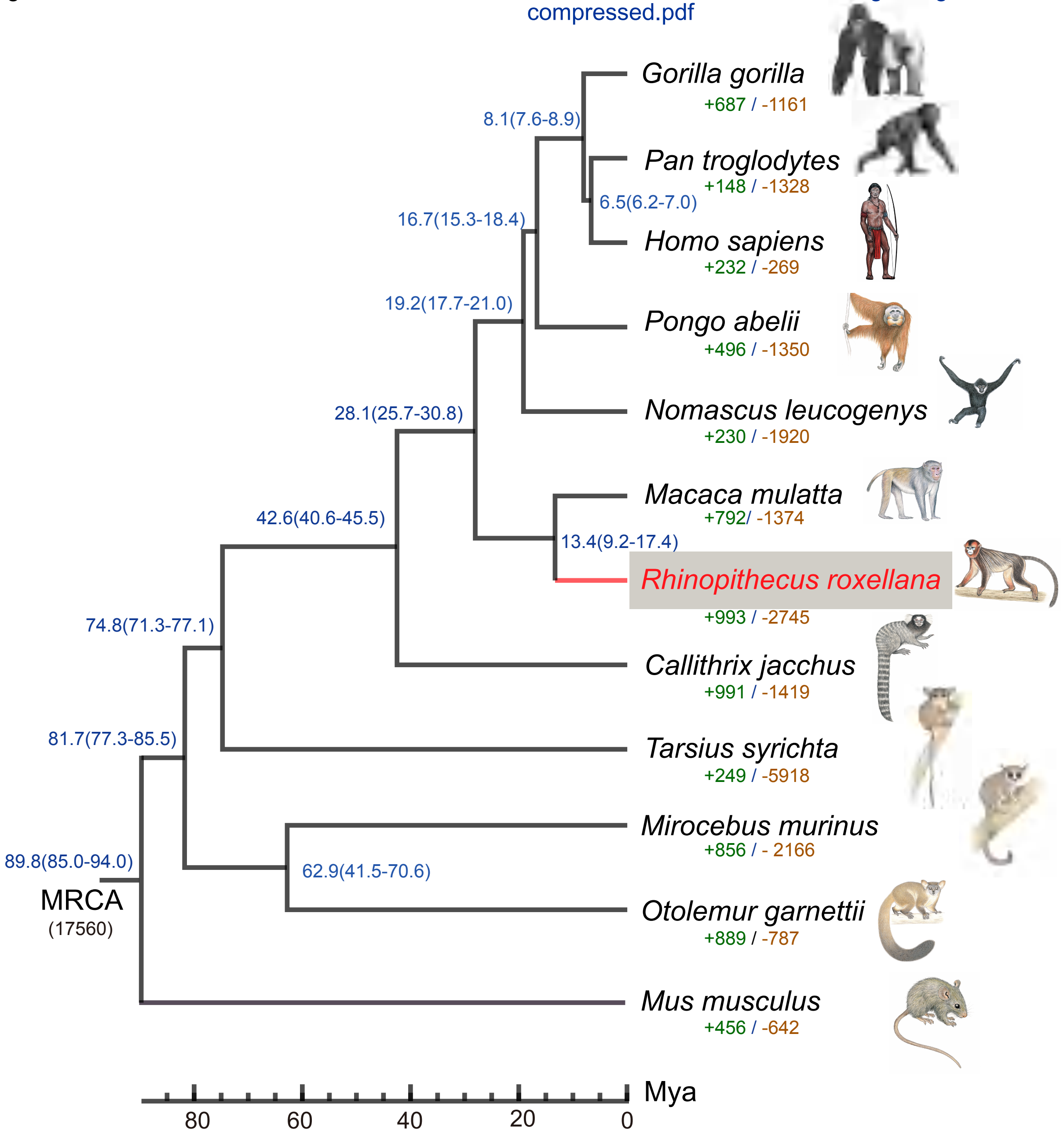

Click here to access/download
Supplementary Material
SI_giga_0609.docx

Personal Cover Click here to access/download;Personal Cover;Coverletter-0609_giga.docx

Dear Hongling,

Thanks for handling our manuscript, and we appreciate the valuable comments from you and two referees.

After digesting these comments, we have carefully revised our manuscript. Firstly, we corrected grammar mistakes and reworded several sentences as suggested by reviewer #1. Secondly, we termed CNVs as duplications and added details about how to identify these duplicate sequences. Thirdly, further analysis of duplicate sequences including location of duplications and examination of genes among these duplications was performed as suggested by reviewer #2.

In this revised version, corrections were made in a document with "Track Changes" mode. Point-by-point responses to the reviewers are also submitted. After addressing the issues raised, we feel the quality of the paper is much improved and hope that our revised manuscript is acceptable for publication in *GigaScience*.

Thanks for your consideration, we look forward to your advice.

Yours sincerely,

Xiao-Guang Qi
Shaanxi Key Laboratory for Animal Conservation
College of Life Sciences
Northwest University
Email: qixg@nwu.edu.cn
