# Supplementary material for: A high-quality genome assembly for the endangered golden snub-nosed monkey (Rhinopithecus roxellana)
Source: Gigascience. 2019 Aug 22;8(8):giz098. doi: 10.1093/gigascience/giz098 (PMC6705546; doi:10.1093/gigascience/giz098)
Supplement: giz098_Supplemental_File [file giz098_supplemental_file.docx]

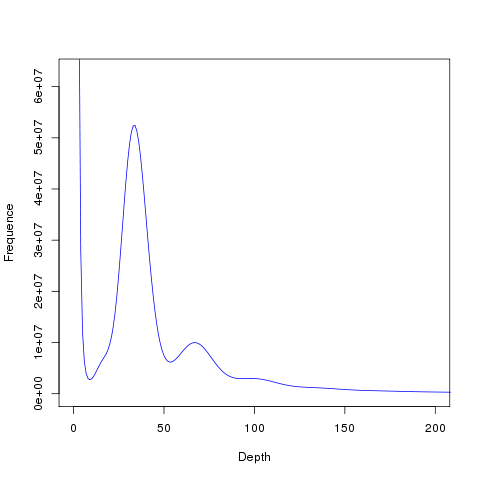


**Supplementary Fig. S1. Genome size estimation using the k-mer method.** The x-axis represented sequence depth (X), while the y-axis was the frequency at that depth.


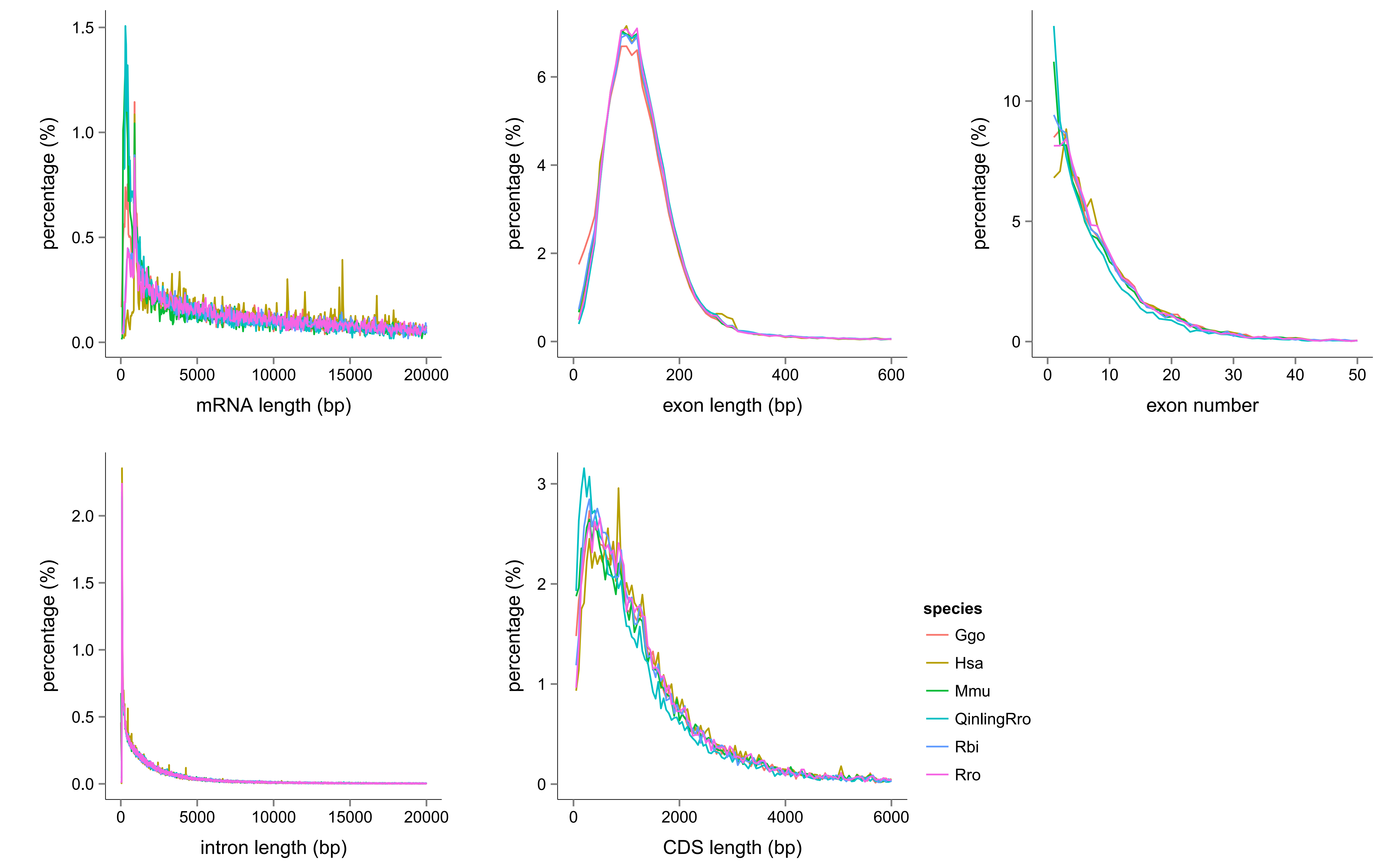


**Supplementary Fig. S2.** Comparisons of each element among genomes of homologous species. Has: *Homo sapiens*; Ggo: *Gorilla gorilla*; Mmu: *Macaca mulatta*; Rbi: *Rhinopithecus bieti*; Rro: *Rhinopithecus roxellana* *hubeiensis*; QinlingRro: *R. r. qinlingensis***.**

**Supplementary Table S1.** The contig assembly based on PacBio subreads.

| Sample ID | Contig(bp) | Number |
| --- | --- | --- |
|  | length | Contig** |
| Total | 3,022,758,273 | 6,730 |
| Max | 30,752,732 | n/a |
| Number >=2000 | n/a | 6,287 |
| N50 | 4,756,836 | 183 |
| N60 | 3,620,692 | 258 |
| N70 | 2,716,165 | 354 |
| N80 | 1,711,034 | 494 |
| N90 | 762,808 | 745 |

Note: The “Number” column represents the number of contigs/scaffolds longer than the value of the corresponding category. n/a:not applicable.

**Supplementary Table S2.** The scaffold assembly based on sspace-longreads results.

| Sample ID | Contig** | | | Scaffold | |
| --- | --- | --- | --- | --- | --- |
|  | Length(bp) | Number | Length(bp) | | Number |
| Total | 3,023,494,490 | 6,829 | 3,033,722,843 | | 4,033 |
| Max | 30,752,732 | n/a | 38,764,396 | | n/a |
| Number >=2000 | n/a | 6365 | n/a | | 3804 |
| N50 | 4,738,625 | 183 | 7,814,108 | | 107 |
| N60 | 3,620,692 | 257 | 6,369,612 | | 151 |
| N70 | 2,716,165 | 353 | 4,783,930 | | 205 |
| N80 | 1,701,671 | 494 | 2,932,713 | | 286 |
| N90 | 758,251 | 745 | 1,349,436 | | 436 |

Note: ** Contig indicates those contigs after scaffolding. The “Number” column represents the number of contigs/scaffolds longer than the value of the corresponding category. n/a:not applicable.

**Supplementary Table S3.** The assembly after gap-filling.

| Sample ID | Contig** | |  | Scaffold | |
| --- | --- | --- | --- | --- | --- |
|  | length (bp) | Number (bp) |  | length (bp) | Number (bp) |
| Total | 3,039,245,139 | 6,147 |  | 3,043,953,449 | 4,011 |
| Max | 30,758,336 | n/a |  | 38,772,111 | n/a |
| >=2000 | n/a | 5,758 |  | n/a | 3,785 |
| N50 | 5,722,222 | 152 |  | 8,197,657 | n/a |
| N60 | 4,239,967 | 213 |  | 6,391,956 | 149 |
| N70 | 3,146,463 | 294 |  | 4,866,996 | 203 |
| N80 | 2,034,229 | 412 |  | 2,934,118 | 284 |
| N90 | 874,222 | 630 |  | 1,329,489 | 437 |

Note: ** Contig indicates those contigs after scaffolding. The “Number” column represents the number of contigs/scaffolds longer than the value of the corresponding category. n/a:not applicable.

**Supplementary Table S4.** The assembly based on BioNano optical map data

| Sample ID | Contig** | |  | Scaffold | |
| --- | --- | --- | --- | --- | --- |
|  | length (bp) | Number (bp) |  | length (bp) | Number |
| Total | 3,039,245,139 | 6,147 |  | 3,192,792,782 | 3,930 |
| Max | 30,758,336 | n/a |  | 38,772,111 | n/a |
| >=2000 | n/a | 5,758 |  | n/a | 3,704 |
| N50 | 5,722,222 | 152 |  | 9,221,994 | 100 |
| N60 | 4,239,967 | 213 |  | 7,457,147 | 139 |
| N70 | 3,146,463 | 294 |  | 5,963,143 | 186 |
| N80 | 2,034,229 | 412 |  | 3,776,219 | 252 |
| N90 | 874,222 | 630 |  | 1,629,500 | 377 |

Note: ** Contig indicates those contigs after scaffolding. The “Number” column represents the number of contigs/scaffolds longer than the value of the corresponding category. n/a:not applicable.

**Supplementary Table S5.** The assembly based on 10X Genomics linked reads.

| Sample ID | Contig**(bp) | |  | Scaffold (bp) | |
| --- | --- | --- | --- | --- | --- |
|  | length | number |  | length | number |
| Total | 3,039,245,139 | 6,147 |  | 3,202,029,686 | 2,314 |
| Max | 30,758,336 | n/a |  | 308,177,001 | n/a |
| >=2000 bp | n/a | 5,758 |  | n/a | 2,088 |
| N50 | 5,722,222 | 152 |  | 24,091,772 | 37 |
| N60 | 4,239,967 | 213 |  | 17,145,987 | 52 |
| N70 | 3,146,463 | 294 |  | 12,467,634 | 74 |
| N80 | 2,034,229 | 412 |  | 8,583,066 | 106 |
| N90 | 874,222 | 630 |  | 4,723,675 | 154 |

Note: ** Contig indicates those contigs after scaffolding. The “Number” column represents the number of contigs/scaffolds longer than the value of the corresponding category. n/a: not applicable.

**Supplementary** **Table S6**. The read mapping rate and the coverage of the assembled genome determined with BWA.

|  |  | Percentage |
| --- | --- | --- |
| Reads | Mapping rate (%) | 99.17 |
| Genome | Average sequencing depth | 100.96 |
|  | Coverage (%) | 99.27 |
|  | Coverage at least 4X (%) | 99.08 |
|  | Coverage at least 10X (%) | 98.91 |
|  | Coverage at least 20X (%) | 98.63 |

**Supplementary** **Table S7**. The SNPS identified in the genome of *R. roxellana***.**

|  | Number | Percentage（%） |
| --- | --- | --- |
| All SNP | 559048 | 0.0320 |
| Heterozygosis SNP | 551358 | 0.0315 |
| Homology SNP | 7690 | 0.0004 |

All SNP：total SNPs found in the genome, including heterozygosis SNPs and homology SNPs.

**Supplementary Table S8.** Genome assessment based on BUSCO annotations.

| Species | BUSCO annotation assessment results |
| --- | --- |
| QinlingRro | C:94.0% [S:92.4%, D:1.6%], F:2.9%, M:3.1%, n:4104 |

Note: QinlingRro: *R. roxellana* of Qinling Mountain; C: Complete BUSCOs; S: Complete Single-Copy BUSCOs; D: Complete Duplicated BUSCOs; F: Fragmented BUSCOs; M: Missing BUSCOs; n: Total BUSCO groups searched.

**Supplementary Table S9.** Genome assessment based on CEGMA annotation

| Species | Complete unfragmented | Complete fragmented |
| --- | --- | --- |
| QinlingRro | 220 (88.71%) | 13 (5.24%) |

Note: QinlingRro: *R. roxellana* of Qinling Mountain.

**Supplementary** **Table S10.** Prediction of repeat elements prediction in the genome assembly.

| Type | Repeat Size(bp) | % of genome |
| --- | --- | --- |
| Trf | 188,281,472 | 6.20 |
| Repeatmasker | 1,343,796,331 | 32.46 |
| Proteinmask | 349,457,816 | 11.50 |
| *De novo* | 1,338,423,791 | 44.05 |
| Total | 1,380,391,966 | 50.81 |

**Supplementary** **Table S11.** Prediction of repetitive sequences in the genome assembly

|  | **Repbase TEs** | |  | ***De novo* TEs** | |  | **Only *De novo* TEs without Repbase TEs** | |  | **Combined TEs** | |
| --- | --- | --- | --- | --- | --- | --- | --- | --- | --- | --- | --- |
| **Type** | length(bp) | percentage in genome |  | length(bp) | percentage in genome |  | length(bp) | percentage in genome |  | length(bp) | percentage in genome |
| DNA | 41,779,013 | 1.375003 |  | 16,484,923 | 0.542541 |  | 970049 | 0.031926 |  | 24,595,646 | 0.81 |
| LINE | 499,491,586 | 16.438932 |  | 815,734,520 | 26.846908 |  | 133072579 | 4.379596 |  | 857,892,164 | 28.23 |
| SINE | 301,643,640 | 9.927493 |  | 342252176 | 11.263974 |  | 27074960 | 0.891073 |  | 332,475,942 | 10.94 |
| LTR | 145,115,023 | 4.775928 |  | 274,512,614 | 9.034575 |  | 37879360 | 1.24666 |  | 288,310,596 | 9.49 |
| Other | 247 | 0.000008 |  | 0 | 0 |  | 0 | 0 |  | 247 | 0.000008 |
| Unknown | 521,408 | 0.01716 |  | 6073246 | 0.199879 |  | 2276575 | 0.074925 |  | 5,665,024 | 0.19 |
| Total | 986,430,197 | 32.464729 |  | 1,338,423,791 | 44.049307 |  | 186195432 | 6.127939 |  | 1,355,311,649 | 44.605109 |

**Supplementary** **Table S12.** The duplicated sequences (DS) identified in the genome assembly

| DS length | Number | Total length(bp) |
| --- | --- | --- |
| <2k | 75 | 122000 |
| 2k-5k | 195 | 650100 |
| 5k-10k | 148 | 1094300 |
| 10k-15k | 97 | 1194600 |
| 15k-20k | 56 | 962400 |
| 20k-40k | 76 | 2018700 |
| 40k-60k | 15 | 724900 |
| 60k-80k | 4 | 305700 |
| 80k-100k | 3 | 259200 |
| >100k | 7 | 1867000 |
| Total | 676 | 9198900 |

**Supplementary** **Table S13.** Summary and characteristics of the predicted RNAs.

| Type | | Copy (w*) | Average length (bp) | Total length (bp) | percentage of genome |
| --- | --- | --- | --- | --- | --- |
| miRNA | | 17,813 | 97 | 1,731,711 | 0.056993 |
| tRNA | | 460 | 75 | 34,588 | 0.001138 |
| rRNA | rRNA | 608 | 120 | 72,705 | 0.002393 |
|  | 18S | 26 | 175 | 4,551 | 0.00015 |
|  | 28S | 167 | 201 | 33,602 | 0.001106 |
|  | 5.8S | 6 | 107 | 640 | 0.000021 |
|  | 5S | 409 | 83 | 33,912 | 0.001116 |
| snRNA | snRNA | 3,656 | 102 | 371,883 | 0.012239 |
|  | CD-box | 469 | 100 | 47,127 | 0.001551 |
|  | HACA-box | 430 | 145 | 62,535 | 0.002058 |
|  | splicing | 2,547 | 96 | 244,462 | 0.008046 |

**Supplementary Table S14**. The functional annotations of the genes predicted in the *R. roxellana* genome.

| Database | Number | Percentage (%) |
| --- | --- | --- |
| NR | 22019 | 97.88 |
| SwissProt | 20768 | 92.31 |
| KEGG | 18262 | 81.18 |
| InterPro | 20104 | 89.36 |
| Pfam | 17685 | 78.61 |
| GO | 14433 | 64.16 |
| Annotated | 22053 | 98.03 |
| Total | 22497 | \ |

**Supplementary Table S15**. Assessment of the new genome assembly using unigenes.
sequences

| Species | Dataset | Number | Total length  (bp) | With>90% sequence in one scaffold | | With>50% sequence in one scaffold | |
| --- | --- | --- | --- | --- | --- | --- | --- |
|  |  |  |  | Number | Percent (%) | Number | Percent  (%) |
| *R. r.* *hubeiensis* | >0bp | 237083 | 224698259 | 210919 | 88.96 | 224879 | 94.85 |
|  | >200bp | 237083 | 224698259 | 210919 | 88.96 | 224879 | 94.85 |
|  | >500bp | 92746 | 182536439 | 83177 | 89.68 | 91395 | 98.54 |
|  | >1000bp | 58140 | 158459148 | 51905 | 89.28 | 57588 | 99.05 |
| *R. r. qinlingensis* | >0bp | 237083 | 224698259 | 217508 | 91.74 | 225868 | 95.27 |
|  | >200bp | 237083 | 224698259 | 217508 | 91.74 | 225868 | 95.27 |
|  | >500bp | 92746 | 182536439 | 88124 | 95.02 | 91904 | 99.09 |
|  | >1000bp | 58140 | 158459148 | 55437 | 95.35 | 57880 | 99.55 |

**Supplementary Table S16.** The GO annotations of the expanded gene families in the R. roxellana genome (adjusted *P*-value < 0.05)

| GO_ID | GO_Term | GO_Class | Adjusted *P*value | x1 |
| --- | --- | --- | --- | --- |
| GO:0003735 | structural constituent of ribosome | MF | 0 | 295 |
| GO:0005840 | ribosome | CC | 0 | 295 |
| GO:0005198 | structural molecule activity | MF | 0 | 338 |
| GO:0030529 | ribonucleoprotein complex | CC | 0 | 297 |
| GO:0006412 | translation | BP | 2.98E-315 | 295 |
| GO:0043232 | intracellular non-membrane-bounded organelle | CC | 6.89E-221 | 362 |
| GO:0044444 | cytoplasmic part | CC | 2.46E-206 | 325 |
| GO:0005737 | cytoplasm | CC | 9.14E-170 | 350 |
| GO:0032991 | macromolecular complex | CC | 3.07E-152 | 392 |
| GO:0044267 | cellular protein metabolic process | BP | 2.47E-133 | 381 |
| GO:0019538 | protein metabolic process | BP | 2.17E-98 | 397 |
| GO:0043229 | intracellular organelle | CC | 2.05E-97 | 419 |
| GO:0010467 | gene expression | BP | 7.23E-96 | 348 |
| GO:0034645 | cellular macromolecule biosynthetic process | BP | 1.71E-95 | 342 |
| GO:0044424 | intracellular part | CC | 7.01E-95 | 469 |
| GO:0005622 | intracellular | CC | 6.97E-86 | 505 |
| GO:0044249 | cellular biosynthetic process | BP | 3.51E-76 | 353 |
| GO:0044464 | cell part | CC | 1.26E-74 | 512 |
| GO:0009058 | biosynthetic process | BP | 3.12E-71 | 354 |
| GO:0044260 | cellular macromolecule metabolic process | BP | 3.36E-62 | 451 |
| GO:0044237 | cellular metabolic process | BP | 8.83E-53 | 518 |
| GO:0043170 | macromolecule metabolic process | BP | 1.66E-47 | 467 |
| GO:0044238 | primary metabolic process | BP | 4.91E-37 | 519 |
| GO:0008152 | metabolic process | BP | 6.05E-37 | 589 |
| GO:0006457 | protein folding | BP | 3.76E-30 | 46 |
| GO:0003755 | peptidyl-prolyl cis-trans isomerase activity | MF | 6.96E-29 | 40 |
| GO:0044391 | ribosomal subunit | CC | 3.15E-23 | 35 |
| GO:0005882 | intermediate filament | CC | 8.64E-23 | 39 |
| GO:0009987 | cellular process | BP | 1.16E-20 | 658 |
| GO:0006091 | generation of precursor metabolites and energy | BP | 2.08E-20 | 30 |
| GO:0044282 | small molecule catabolic process | BP | 3.00E-16 | 30 |
| GO:0004984 | olfactory receptor activity | MF | 1.69E-15 | 49 |
| GO:0004459 | L-lactate dehydrogenase activity | MF | 1.56E-14 | 18 |
| GO:0006096 | glycolysis | BP | 2.29E-14 | 27 |
| GO:0015934 | large ribosomal subunit | CC | 9.20E-13 | 21 |
| GO:0003723 | RNA binding | MF | 1.10E-10 | 61 |
| GO:0016616 | oxidoreductase activity, acting on the CH-OH group of donors, NAD or NADP as acceptor | MF | 3.98E-09 | 23 |
| GO:0015935 | small ribosomal subunit | CC | 7.24E-09 | 14 |
| GO:0044446 | intracellular organelle part | CC | 4.37E-07 | 117 |
| GO:0005833 | hemoglobin complex | CC | 8.84E-06 | 9 |
| GO:0042254 | ribosome biogenesis | BP | 2.14E-05 | 18 |
| GO:0015671 | oxygen transport | BP | 7.02E-05 | 9 |
| GO:0019825 | oxygen binding | MF | 7.02E-05 | 9 |
| GO:0019843 | rRNA binding | MF | 8.68E-05 | 11 |
| GO:0045095 | keratin filament | CC | 9.08E-05 | 16 |
| GO:0020037 | heme binding | MF | 0.000128 | 24 |
| GO:0044445 | cytosolic part | CC | 0.000157 | 15 |
| GO:0044430 | cytoskeletal part | CC | 0.0004 | 50 |
| GO:0007156 | homophilic cell adhesion | BP | 0.001048 | 21 |
| GO:0004618 | phosphoglycerate kinase activity | MF | 0.001713 | 5 |
| GO:0016758 | transferase activity, transferring hexosyl groups | MF | 0.002593 | 24 |
| GO:0044262 | cellular carbohydrate metabolic process | BP | 0.003427 | 30 |
| GO:0044085 | cellular component biogenesis | BP | 0.010483 | 42 |
| GO:0016620 | oxidoreductase activity, acting on the aldehyde or  oxo group of donors, NAD or NADP as acceptor | MF | 0.015091 | 8 |
| GO:0004420 | hydroxymethylglutaryl-CoA reductase (NADPH) activity | MF | 0.023335 | 4 |
| GO:0006884 | cell volume homeostasis | BP | 0.023335 | 4 |
| GO:0004869 | cysteine-type endopeptidase inhibitor activity | MF | 0.034095 | 9 |

Note：MF indicates molecular function; BP indicates biological process; while CC indicates the cellular component.
