# Supplementary material for: A high-quality genome assembly for the endangered golden snub-nosed monkey (Rhinopithecus roxellana)
Source: Gigascience. 2019 Aug 22;8(8):giz098. doi: 10.1093/gigascience/giz098 (PMC6705546; doi:10.1093/gigascience/giz098)
Supplement: giz098_Response_to_Reviewer_Comments_Original_Submission [file giz098_response_to_reviewer_comments_original_submission.pdf]

Author's Response To Reviewer Comments

Close

Editor Comments to Author:

In particular major improvements are required in the writing and we would strongly recommend you use a native English speaker or professional company to improve the writing.
Response to comment 1
Thanks for this comment, the manuscript has been revised and polished by an English language editing service of LetPub.

We have strong policies regarding reproducibility and agree with the referees that significant additional methodological detail is required.
Response to comment 2
Thanks for your comment, we added the methodological details substantially to be clear and straightforward. In addition, we added some key details about the generated data (sequencing, calibration times, N50 length et al.,) and performed several additional analyses including CNVs identification, synteny analysis and SNP calling et al. as you and reviewers suggested.

On top of including detail on the software versions and setting, we would strongly recommend you capture this detail using protocols.io. You can re-use and adapt the protocols we have stored in our group page or create your own (and if you provide these in stepwise manner we can even upload them for you):
Response to comment 3
Following this comment, we have captured those methodological details using protocols.io. with our own account. Please check out on the website of "https://www.protocols.io/private/EAFC44C786ABCE2257FD0D5B9E0D7EF3".

Reviewer Comments to Author:

Reviewer #1:
This manuscript reports a new whole genome assembly for an interesting nonhuman primate species, Rhinopithecus roxellana. This is a colobine species that has a number of unusual characteristics, including but not limited to unusual pelage, highly derived facial morphology, and social organization that is not entirely unique but is rare among Old World monkeys or other anthropoids. There are five species in the genus, and all are threatened or endangered, so there is a conservation benefit to this genome sequencing as well as basic comparative primate evolutionary genomics. There is a previously published whole genome assembly for this species, but this new assembly is a significant improvement (see below). Consequently, there are several elements of this work that make it noteworthy.

The new assembly is based on an effective and technically advanced combination of approaches. The authors began by sequencing this genome using PacBio Sequel long reads, and assembling them using FALCON and PBjelly. The authors generated Illumina short reads and polished the PacBio/FALCON assembly with those. The authors also make use of BioNano optical mapping and 10X linked-reads to increase completeness and contiguity. Finally, Hi-C mapping is used to produce near full chromosome length scaffolds. The result is a 3.04 gigabase assembly with contig N50 of 5.72 Mb and scaffold N50 of 144.56 Mb. These statistics make this one of the most complete and highly contiguous assemblies available for any nonhuman primate (confirmed using BUSCO and CEGMA analyses). The authors then annotated this genome using a series of annotation software tools, and identified 22,497 genes.

This new genome assembly is a valuable resource for any investigator working on the genetics or genomics of Rhinopithecus. In addition, this is a high quality - high contiguity assembly, so it will be useful for laboratories working on other closely related colobines. Lastly, the authors report some initial analyses of repetitive sequences and gene family expansions and contractions using this new

Rhinopithecus assembly.

While this genome sequence seems to be a valuable resource for the primate genomics community, this manuscript has a significant number of serious flaws and problems. One issue is that the quality of the grammar and text is not adequate. I realize that the authors may not be native speakers of English, and that this can be a challenge. But this manuscript needs major assistance in terms of editing before it is ready for serious consideration.
Response to this comment
Thanks for this comment, the manuscript has been revised and polished by an English language editing service of LetPub.

I have other specific concerns as well.
1) This is minor but having two different line numbering systems printed on the same pages causes confusion. I will use the numbers that are actually tied to specific lines in the text, rather than the more densely packed numbers that seem to just run down each page. The authors should delete the dense numbers.
Response to comment 1
Thanks for your kindly review. We deleted the dense numbers.

2) Page 4, lines 54-56. While the social organization of Rhinopithecus roxellana is interesting and deserves more study, it seems overly optimistic for the authors to argue that production of this genome assembly will ultimately support genetic studies that make contributions to our "…understanding the behavior patterns of human society in social-anthropology." Studies of comparative social relationships and social organization are important and primates can provide information about human evolution. But this statement seems to me to be overly ambitious in terms of research outcomes.
Response to comment 2
Thanks for this comment. We changed the statement as follows:
"Therefore, R. roxellana is an ideal model for the analysis of social structure evolution in primates and may also provide opportunities to investigate evolutionary and socio-anthropological patterns of human society.".

3) There are mistakes in capitalization and spelling of words. For example, the Shennongjia Mountains are not capitalized in line 63, but "Gorillas" is incorrectly capitalized in line 75 and "Colobine" is regularly capitalized when it need not be. "Quiver" is misspelled in line 125.
Response to comment 3
Corrected. We also checked other mistakes in capitalization and spelling of words throughout the manuscript.

4) Line 75 states the gorillas and orangutans "…have the closest genetic relationship with humans" but of course that is chimpanzees and bonobos, not gorillas and/or orangutans.
Response to comment 4
Thanks for this comment. We are sorry that we made a mistake here and we changed the statement as follows:
"New sequencing technologies, including Pacific Bioscience's single-molecule real-time (SMRT) sequencing, BioNano optical mapping, and Hi-C-based chromatin interaction maps, have been used in several species closely related to humans, including gorillas (Gorilla gorilla gorilla) [17], chimpanzees (Pan troglodytes) [18], and Sumatran orangutans (Pongo abelii) [18], as well as in other species, including the domestic goat (Capra hircus) [19].".

5) I think the language in line 86 is a bit too optimistic and ambitious. The authors state that this assembly "…may allow us to comprehensively understand R. roxellana…". I do not know what it would mean to "comprehensively understand" a primate species, but I do not think we are yet close to that point.
Response to comment 5
Thanks for this comment. We changed the statement as follows:
"This updated genome assembly may allow us to further investigate R. roxellana, providing new opportunities to analyze evolutionary history and to identify genetic changes associated with the development of specific traits in this species".

6) Page 6, line 87: It is not clear to me what the authors mean by "genetic-specific signatures of this species"?
Response to comment 6

Thanks for this valuable comment. In fact, we were intended to term those genetic changes associated with the development of species-specific traits as “genetic-specific signatures of this species”. We realized that this sentence was confusing and not clear enough. We changed the statement as follows: “genetic changes associated with the development of specific traits in this species”.

7) Page 6, line 93-94. Was the animal used to produce the DNA for the sequencing wild-caught or captive bred at Louguantai? If captive bred, were the parents wild-caught?
Response to comment 7
Thanks for this comment. The animal used for the sequencing was an adult male R. roxellana qinlingensis in Qinling Mountain. The animal that died naturally in Qinling Mountain was immediately stored in ultra-cold storage freezer at Louguantai Breeding Centre. We reworded the statement as follows:
“The animal used for sequencing was an adult male R. r.. qinlingensis from Qinling Mountain, who died naturally and the dead body was stored in ultra-cold storage freezer at Louguantai Breeding Centre, Xi’an, Shaanxi Province, China.”

8) Page 7, lines 103-105. BioNano optical mapping is a technique for using restriction enzymes to nick and label DNA at short known target sequences. The map of nicked sites is used to scaffold a genome or confirm the organization of contigs. It is not clear what the authors mean when they state that they "…acquired 463.75 Gb clean reads" from the BioNano Genomics Irys platform. There are no sequence reads generated by the Irys platform. This section does not make sense to me. Instead, the authors should present the actual results of the optical mapping in terms of the number of sites examined and the concordance between the observed BioNano map and the predicted map based on the assembled contigs and scaffolds.
Response to comment 8
Thanks for your valuable comments. We are sorry that we used the wrong term here. Of course, there are no sequence reads generated by the Irys platform, the generation by which should be large DNA molecules. As for the number of sites examined in this study, the average label density for the BioNano map is 11.66 per 100 kb, while the average label density is 12.62 per 100 kb for the predicted map based on those assembled contigs and scaffolds. Thus, the observed BioNano map is consistent with the predicted map. We added several sentences to clarify this point.
“The average label density examined for the BioNano map is 11.66 per 100 kb, while the average label density is 12.62 per 100 kb for the predicted map based on the assembled contigs. Thus, the observed BioNano map is consistent with the predicted map. The BioNano map generated 463.75 Gb of large DNA molecules.”

9) I do not think that Figure 2 adds much to this paper. The authors used Hi-C for scaffolding, and that does provide useful data. But simply inserting a figure showing Hi-C interaction frequencies without doing any further analysis of the details of DNA-DNA interaction or characterizing the topologically associating domains provides no significant new information or insight.
Response to comment 9
Thanks for your valuable comment. The fig. 2 was based on the interaction frequencies between pairs of 100-kb genomic regions. In principle, higher counts indicate increased frequency of chromatin interaction and closer spatial distance between the two sequences, darker red means stronger interaction strength. This strategy has significantly advanced the assembly quality with chromosome-length scaffolds. The fig. 2 presented here was used to indicate the reliability of our assembly.
As for the further analysis of the details of DNA-DNA interaction or characterizing the topologically associating domains, we agree that these analysis were useful. However, they may be beyond the scope of this report, which aims to report a high-quality genome for further studies. We also added several sentences to make the figure legend of fig. 2 more clear.
“Hi-C interactions within and among chromosomes of R. roxellana chromosomes (Chr1–Chr22); interactions were drawn based on the chromatin interaction frequencies between pairs of 100-kb genomic regions (as determined by Hi-C). In principle, darker red cells indicate stronger and more frequent interactions, which in turn imply that the two sequences are spatially close.”

10) Page 9, lines 151-152. I do not understand the sentence that begins "With a ratio number…"
Response to comment 10
Thanks for this comment. We reworded this sentence to clarify this point.
“Approximately 99.17% of the short reads were mapped to the genome assembly. Further investigations indicated that these reads covered approximately 99.27% of the total assembly (Supplementary Table S6).”.

11) Page 9-10, lines 152-159. Using BUSCO and CEGMA to assess the completeness of the genome assembly is a very good idea. But the authors should report not just how many BUSCO or CEGMA genes were identified, but how many were complete and unfragmented and how many were complete and fragmented.
Response to comment 11
Thanks for this comment. During the BUSCO analysis, the annotation results were classified as complete BUSCOs, fragmented BUSCOs and missing BUSCOs. We did not report those results in the manuscript, however, these detailes were shown in Supplementary Table S8. Simply, the complete BUSCOs occupied a proportion of 94.0%, while the fragmented BUSCOs occupied only 2.9%. In addition, we added the CEGMA results in Supplementary Table S9, which showed that the 220 genes were complete and unfragmented , while 13 was complete and fragmented. We also added these results in our manuscript.
"In addition, we estimated assembly completeness using Benchmarking Universal Single-copy Orthologs (BUSCO) v3.0.2 [27], with the parameters "-i -o -l -m genome -f -t." based on mammalia_odb9 (creation date: 2016-02-13; number of species: 50; number of BUSCOs: 4,104). BUSCO analysis identified 4,104 mammalian BUSCOs in the newly assembled R. roxellana genome: 94.0% complete BUSCOs, 2.9% fragmented BUSCOs, and 3.1% missing BUSCOs (Supplementary Table S8). Assembly completeness was measured using the core eukaryotic gene (CEG)-mapping approach (CEGMA v2.5) [28]. Of the 248 CEGs known from six model species, 93.95% (233 of 248) were identified in our new genome assembly. Of these, 220 CEGs were complete and unfragmented, and the remaining 13 were complete but fragmented (Supplementary Table S9). Together, these analyses indicated that our new genome assembly was highly accurate and complete."

12) Page 12, lines 226-227. What fossil calibration times were used?
Response to comment 12
Thanks for this comment. The fossil calibration times were derived from Timetree (http://www.timetree.org/). The following calibration times were used: Homo sapiens VS Callithrix jacchus (40.6-45.7 MYA); Homo sapiens VS Pan troglodytes (6.2~7 MYA); Homo sapiens VS Mus musculus (85-94 MYA) and Homo sapiens VS Tarsius syrichta (71~77 MYA). We also added these fossil calibration times in our manuscript.
"The following fossil calibrations were used: Homo sapiens vs. Callithrix jacchus (40.6–45.7 MYA, million years ago); Homo sapiens vs. Pan troglodytes (~6.2–7 MYA); Homo sapiens vs. Mus musculus (85–94 MYA); and Homo sapiens vs. Tarsius syrichta (~71–77 MYA). "

13) Page 14, lines 232-235. Rhinopithecus gene families were expanded or contracted compared to what taxa? Compared to human? Compared to the ancestral primate genome? Compared to an Old World monkey outgroup?
Response to comment 13
Thanks for this comment. The expansion and contraction of gene families of Rhinopithecus roxellana were estimated by comparing those of the most recent common ancestor between Rhinopithecus roxellana and Macaca mulatta. We added one sentence in the figure legend of fig. 4 to clarify this point.
"Numbers under each species indicate the number of gene families that have been expanded (green) and contracted (light yellow) since the split of species from the most recent common ancestor (MRCA).".

14) I think two column headings in Table 3 are switched. I doubt that the average intron length for the Rhinopithecus Augustus gene models is 196bp, while the average exon length for the same gene models is 5,112bp. Seems to me those two labels are probably switched.
Response to comment 14
Thanks for your valuable comment. We are sorry that we made a mistake here, we put them in right order now. Please see Table 3 for details.

Reviewer #2:
The authors present an assembly of golden snub-nosed monkey using a range of sequencing technologies, including long read sequencing. Overall the manuscript is mostly clear to follow and the assembly approaches are standard and appear to be well performed. A very large amount of data was generated, although the methods are very short and some details are lacking, it appears that standard and appropriate assembly approaches were used. Some key details about the generated data are missing, and there are some additional analyses that, if completed, would greatly improve the manuscript.
Response to comment 1
Thanks for your valuable comment. we added the methodological details substantially to be clear and

straightforward. Please see the “De novo assembly” section for details. In addition, some key details about the generated data (N50 length, software parameters et al.) were present this time and several additional analyses including CNVs identification, synteny analysis and SNP calling et al. were also performed as suggested.

I could not find descriptions of the characteristics of the generated data, particularly average/n50 length of Pacbio reads, molecule size of the optical mapping and of 10X data. These are key parameters that should be reported.
Response to comment 2
Thanks for this comment. The average/N50 length of Pacbio reads and molecule size of the optical mapping was 16.69 kb and 338 kb, respectively. As for the 10X data, since paired-end of 350 bp sequencing was performed, N50 length was not applicable for this case. It was estimated that a total of 423.32 Gb clean reads were generated for 10X data. We added one sentence to descript the characteristics of the generated data.
“…., the average/N50 length of Pacbio reads was 16.69 kb.”
“The average/N50 length of the molecules used for optical mapping was 338 kb.”.

Line 104 The description of the Bionano data should be clarified. I am not sure that "reads" is the right term for data from this optical mapping platform. Same for term 'sequence coverage' for optical mapping data in Table 1.
Response to comment 3
Thanks for this comment. We added several sentences to detail the BioNano data. We agree that no reads generated from optical mapping platform and we changed the term of "reads" as molecules. Also, the term "sequence coverage" was not an proper term for optical mapping data, we removed the sequence coverage value of optical mapping data in Table 1.
“The average/N50 length of the molecules used for optical mapping was 338 kb. The average BioNano optimal marker density was 11.66 per 100 kb, while the average marker density was 12.62 per 100 kb for the predicted map based on the assembled scaffolds. Thus, the observed BioNano map was consistent with the predicted map. The BioNano map generated 463.75 Gb of large DNA molecules.”

The manuscript would benefit from some comparison of how much better the gene annotation is relative to previous assembly, but this and other biological/comparative analyses may be beyond the scope of this report.
Response to comment 4
Thanks for this comment. As for the gene annotation, our new assembly was better than previous assembly from at least two aspects. Firstly, we assessed genome assembly completeness by mapping transcriptome unigenes to the two assembly versions using BLAT v.36. Results showed that the completeness degree (percentage of unigenes aligned to a single scaffold in genome) was higher in our assembly (95.35%) compared with that in previous assembly (89.28%) for unigenes larger than 1000 bp (Supplementary Table S15), demonstrating the contiguity of our new assembly. Secondly, the number of genes annotated to the public database to the total number of predicted genes was higher in our new assembly (98.03%) than that in previous version (94.52%).

From Supplementary Tables S2-3, it seems that the largest increase in n50 scaffold length came from 10X linked read data, not from the bionano optical map. I do not think this is expected, given that optical map data should provide very long range information. The manuscript would be clearer for the reader if some description for why such a gain was found from 10X data was described, and if such results are typical.
Response to comment 5
Thanks for this valuable comment. We checked our assembly description carefully and found some details were not shown. Actually, the first stage of assembly was conducted mainly from three procedures: (a). PacBio long reads assembly using the falcon pipeline, assembly was further polished by Quiver and Pilon-1.18 (contig N50: 4.7 Mb); (b). SSPACE-LongRead (version 1-1) was implemented for getting a longer scaffold (contig N50, 4.7 Mb; scaffold N50, 7.8 Mb); (c). PBjelly was used to close gaps (contig N50, 5.7 Mb; scaffold N50, 8.2 Mb). As you see, the increase of scaffold N50 in this stage mainly came from SSPACE-LongRead procedure (7.8 Mb VS 4.7 Mb). Then the assembled PacBio scaffolds were used as input for scaffolding by hybridScaffold software at the BioNano stage, which generated a hybrid assembly with scaffold N50 of 9.22 Mb. It seems that BioNano optical map did not increase N50 too much (9.22 Mb VS 8.2 Mb), we predicted that the main reason was the employment of SSPACE-LongRead procedure during the first stage assembly. This program dealt with the scaffold construction effectively and the efficiency may be overlap with the performance at the BioNano stage in our study. Therefore, it was reasonable the increase in scaffold N50 was not largely from the BioNano optical map

stage. Following this, the 10X genomic linked reads were employed to construct larger scaffolds, fragScaff software was employed to finish the super-scaffold construction. This procedure has increased the genome assembly with a scaffold N50 of 24.09 Mb, suggesting the efficiency of 10X genomic linked reads in our work (24 Mb VS 9.2 Mb).
The efficiency of 10X genomic linked reads was also seen in other publication (Mostovoy et al., 2016, Nature Methods), which shows that 10X linked read data contributes more to the increase in N50 length than the BioNano optical map. Despite this, we still did not know whether the largely increase from 10X data was typical or not, as only few publications were available using the combination of 10X reads and BioNano map. We added several sentences to expand our method section, particularly in the "De novo assembly of the R. roxellana genome" section.

Standard repeat masker, gene prediction, and other analysis is performed. The manuscript would be strengthened by also a consideration of duplicated sequences, which could be identified based on Illumina sequence data read depth. This may be beyond scope of this report, but could be considered.
Response to comment 6
Thanks for this comment. We added duplicated sequences/copy number variant (CNVs) analysis based on read depth estimated from illumine short reads to the assembled genome using BWA. Results showed that a total of 676 duplicated blocks were identified, whose total length was 9,198,900 bp. We added one paragraph to clarify this point.
"We also performed a CNV analysis. In brief, we first mapped the Illumina short reads to the assembled genome using BWA with default parameters. Then, the sorted mapping bam file was used as input for CNVnator v0.3.3 [38], with the parameters "-unique -his 100 -stat 100 -call 100.". The obtained CNVs were filtered, retaining only those where q0 was <0.5 and e-val1 was <0.05. After filtering, 676 CNVs remained, with a total length of 9,198,900 bp (Supplementary Table S12). ".

Has the assembly itself been submitted to proper databases and repositories (such as Genbank)? I could not find this listed, only the raw data.
Response to comment 7
Thanks for this comment. The genome assembly and other supporting data have been submitted to GigaDB database and NCBI successfully. However, we did not release them now as interest competition exist and several research groups are also working on this species. We appreciate the editor and reviewers understand the challenges in this case, and we will make related data available once this article is published.

In table 2 and others, what does the 'number' column mean? For example, are there 151 contigs >= to the N50 length of 5.7mb? The meaning of the columns in the tables should be clearly explained.
Response to comment 8
Thanks for this comment. Yes, this example explains the exact mean of 'number' column. Following your comment, we revised Table 2 to be more clear. We added one sentence to explain the meaning of the 'number' column. In addition, we checked and revised other tables if not clearly explained (for example, Table 3 and Supplementary Table S1).
"The "Number" column represents the number of contigs/scaffolds longer than the value of the corresponding category.".

The legend for figure 2 is not adequate. What does the color scale signify? What is the reader supposed to conclude from the figure?
Response to comment 9
Thanks for this comment. This plot shows the interactions between two 100-kb genomic regions (as determined by Hi-C), darker red means stronger interaction strength. We added two sentences in the figure legend to address this comment.
"Hi-C interactions within and among chromosomes of R. roxellana chromosomes (Chr1–Chr22); interactions were drawn based on the chromatin interaction frequencies between pairs of 100-kb genomic regions (as determined by Hi-C). In principle, darker red cells indicate stronger and more frequent interactions, which in turn imply that the two sequences are spatially close."
This figure tries to express the information of Hi–C interactions among 22 chromosomes with a 100 kb resolution. Stronger interactions are indicated in darker red and weak interactions are indicated in light yellow. The fig. 2 presented here was used to indicate the reliability of our assembly during the Hi-C stage.

Reviewer #3:
Wang, Wu et al. have produced a high-quality reference genome assembly for the emblematic golden snub-nosed monkey. The authors used a combination of long PacBio reads, 10-X linked reads, Hi-C contact maps, BioNano Optical maps, and Illumina paired end sequences, all of which were sequenced to a very high coverage. The resulting assembly has very high continuity and given the combination of different sequencing strategies essentially gives as good of an assembly as current methods can produce. The authors have used a state-of-the-art approach to produce their assembly, and the applied methodology is appropriate. The authors have also produced a gene annotation based on homology to other species, as well as expression data. The assembly provides a valuable genomic resource to study snub-nosed monkeys specifically, and Asian colobines in general.
General comments:

R. roxellana already has a genome assembly available, as the authors note in the manuscript. However, there is no comparison at all beyond a contig and scaffold N50. It would strengthen the manuscript if the authors could provide some comparisons to the previous assembly, e.g: A comparative, or what specific regions of the assembly were absent in the previous version, what do they contain, how many gaps were filled, how many of the gene family expansions/contractions are only detectable with the high quality assembly etc.
Response to comment 1
Thanks for this comment. We followed this comment and made some comparisons with previous assembly, including repeat analysis and synteny analysis. In comparison, our new assembly had a higher proportion of repeat sequences (50.82%) as compared to the previous version (46.15%); in particular, the number of LINE (long interspersed elements) transposable elements and tandem repeats was greatly increased (further details are given in the "Identification of repeat elements" section). Thus, the newly assembled genome was substantially more complete and continuous. Also, we aligned our genome against the previous version using MUMMER (v4.0.0beta2) and identified a total of 2,217 insertions in our new assembly. These insertion regions were mainly located in the intergenic and repetitive regions. Further analysis showed that 6,452 gaps in the previous version that were predicted to be filled by >29.7 Mb of sequence in our new assembly. These filled gaps were mainly located in the intergenic and repetitive regions, with a small fraction of the sequence data annotated as gene regions. We added several sentences to clarify this point.
"We evaluated our newly assembled R. roxellana genome against the previously published assembly. The contiguity of our R. roxellana genome was 100fold greater (contig N50: 5.72 Mb; scaffold N50: 144.56) than the previous version (contig N50: 25.5 kb; scaffold N50: 1.55 Mb) [11]. We also aligned our genome against the previous version using MUMMER (v4.0.0beta2) [37] and identified 6,452 gaps in the previous version that were predicted to be filled by >29.7 Mb of sequence in our new assembly. These filled gaps were mainly located in the intergenic and repetitive regions, with a small fraction of the sequence data annotated as gene regions. Our new assembly also had a higher proportion of repeat sequences (50.82%) as compared to the previous version (46.15%); in particular, the number of LINE (long interspersed elements) transposable elements and tandem repeats was greatly increased (further details are given below, in the "Identification of repeat elements" section). Thus, the newly assembled genome was substantially more complete and continuous. It was likely that the remarkable improvement in contiguity was due to the increased read length, deeper sequencing depth, improved gap assembly, and more sophisticated assembly algorithm."

The authors use several different software packages for their analysis. The inclusions of version numbers for the software packages they used seems somewhat arbitrary. Furthermore, no parameter sets apart from "default parameters" are ever presented. Both package versions and parameter settings should absolutely be included, otherwise the methods of the study are not properly understandable. In its current state, I feel the methodological aspects of the manuscript need to be expanded.
Response to comment 2
Following this comment, we added the methodological details substantially to address this comment. Both package versions and parameter settings were included in this version. please see "De novo assembly of the R. roxellana genome" section and other sentences containing software names in our manuscript for details.

The manuscript will benefit from language editing, as at several points the phrasing is somewhat confusing.
Response to comment 3
Thanks for this comment, the manuscript has been revised and polished by an English-language editing service of LetPub.

Specific comments:
L19, L68, L80: The claim of "incompleteness" or "greatly improved" is not backed by a proper comparison to the previous assembly.
Response to comment 4
We followed this comment and made comparisons with previous assembly, including repeat analysis and synteny analysis. In comparison, our new assembly had a higher proportion of repeat sequences (50.82%) as compared to the previous version (46.15%); in particular, the number of LINE (long interspersed elements) transposable elements and tandem repeats was greatly increased. Also, We aligned our genome against the previous version using MUMMER (v4.0.0beta2) [37] and identified 6,452 gaps in the previous version that were predicted to be filled by >29.7 Mb of sequence in our new assembly. These filled gaps were mainly located in the intergenic and repetitive regions, with a small fraction of the sequence data annotated as gene regions. Most importantly, the newly assembled R. roxellana reference genome has 100fold higher contiguity than previous assembly (contig N50: 5.72 Mb versus 25.5 kb and scaffold N50: 144.56 Mb versus 1.55 Mb).
We added several sentences to address this comment in the “Assessment of the genome newly assembled” section. See also the response to your valuable comment 1.

L22: Genetic-specific signatures is awkwardly phrased.
Response to comment 5
Thanks for this valuable comment. In fact, we were intended to term those genetic changes associated with the development of species-specific traits as “genetic-specific signatures of this species”. We realized that this sentence was confusing and not straightforward. We changed the statement as follows: “genetic changes associated with the development of specific traits in this species”.

L25: Technology, not technique
Response to comment 6
Thank you for your kindly review. We did it.

L57: This sentence is vague, please be specific about what these studies have looked at. The term research-hotspot for this species might be a stretch.
Response to comment 7
Thanks for this comment. Specifically, Recent studies of R. roxellana have focused on behavioral dynamics, population history, and social systems. We removed the term research-hotspot in this sentence.
“Recent studies of R. roxellana have focused on behavioral dynamics, population history, and social systems [5-7],”

L58f: This sentence needs rephrasing. What are the groups?
Response to comment 8
Following this comment, we reworded this sentence and also specify species the groups included.
“Genomic analyses have helped to untangle the molecular evolution of several groups, including maize (Zea mays), bats (Myotis brandtii), and killifish (Nothobranchius furzeri) [8-10]”.

L60: differentiate -> be distinguished
Response to comment 9
Thank you for your kindly review. We did it.

L63: Was there more than one assembly before this study?
Response to comment 10
Thanks for this comment. Actually, there is only one assembly published in 2014 before our study. We reworded this sentence as follows to avoid confusing.
“To date, only a single genome assembly is available for R. roxellana. This assembly, published in 2014, was derived from short sequencing reads generated by the Illumina HiSeq 2000 platform.”

L71f: This sentence needs rephrasing; it is not clear to me what the authors want to say.
Response to comment 11
We followed this comment and reworded this sentence to make it clear enough.
“Indeed, many previously unreported transposable elements and specific genes in maize were identified using an improved reference genome [16].”.

L74,L78: Please be specific with respect to the sequencing technology. "High quality" is subjective and

changes with sequencing technologies, so arguing that no "high quality assembly of R. roxellana has been reported" is debatable.
Response to comment 12
Thanks for this comment. These new sequencing technologies used here referred to PacBio SMRT sequencing, BioNano optical mapping, and Hi-C based chromatin interaction maps. Additionally, we agree that "High-quality" is subjective and changes with sequencing technologies. We reworded this sentence to clarify this point.
"However, the R. roxellana genome has not yet been updated using new sequencing approaches, slowing progress towards a better understanding of this endangered species.".

L75: Ref 15. Also includes an assembly for the Chimpanzee, which is closer to Human than either Gorilla or the Orangutan. 'Widely' should be omitted in this sentence.
Response to comment 13
Following this comment, we added the assembly of the chimpanzee in our manuscript. In addition, we removed 'Widely' in this sentence.
"New sequencing technologies including Pacific Bioscience's single-molecule real-time (SMRT) sequencing, BioNano optical mapping, and Hi-C-based chromatin interaction maps, have been used in several species closely related to humans, including gorillas (Gorilla gorilla gorilla) [17], chimpanzees (Pan troglodytes) [18], and Sumatran orangutans (Pongo abelii) [18], as well as in other species, including the domestic goat (Capra hircus) [19].".

L76: "A lot of new findings" is vague, please specify the specific advantages of the new assemblies.
Response to comment 14
Following this comment, we added several sentences to clarify the specific advantages of the new assemblies.
"Importantly, it was estimated that 87% of the missing reference exons and incomplete gene models were recovered using the new gorilla assembly [17]. In addition, several novel genes expressed in the brain were identified using the new orangutan assembly, and complete immune genes with longer repetitive structures were identified in the updated goat genome [19].".

L81: Through combined -> by combining
Response to comment 15
Thank you for your kindly review. We did it.

L110: Cutadapter -> Cutadapt
Response to comment 16
Thank you for your kindly review. We did it.

L115ff: The value for Kerror was omitted.
Response to comment 17
Thanks for this comment, we added the value for Kerror.
"Finally, a total number of 109,210,004,556 k-mers, 1,159,024,556 k-mers with sequencing errors were generated and the peak k-mer depth was 34.".

L125: Quier -> Quiver
Response to comment 18
Thank you for your kindly review. We did it.

L130: To the best of my knowledge, PBJelly doesn't know how to deal with phased assemblies. All previous assembly steps (Falcon, Quiver, Pilon, sspace) also do not talk about phasing information. Please clarify how phasing was dealt with or maintained at this point.
Response to comment 19
Thanks for your valuable comment. We agree that PBJelly and previous assembly steps (Falcon, Quiver, Pilon) could not deal with phased assemblies. The term "phased genome assembly" here was used to indicate the genome assembly finished at this period, but not the "phased haplotype-resolved genome assembly". This sentence was confusing here, we now say: "Thus, at the end of the first stage, the genome assembly had a contig N50 of 5.72 Mb and a scaffold N50 of 8.20 Mb (Supplementary Table S3).".

L130: The authors only mention the scaffold N50 after gap-filling. I see the contig N50 is mentioned in the supplementary, but I cannot find the contig N50 of the base assembly before gap-filling anywhere. It would be worth to mention it to understand the relative contributions of additional steps.

Response to comment 20
Thanks for your comment. Following gap-filling with PBjelly software, contig N50 increased to 8.2 Mb from N50 of 7.8 Mb at previous step. We added details to clarify this point.
"Using the initial genome assembly, SSPACE-LongRead v1-1 [33] was implemented for getting a longer scaffold by processing PacBio long reads and the initial genome assembly with the command "perl SSPACE-LongRead.pl -c -p ." This procedure generated a genome assembly with scaffold N50 of 7.81 Mb (Supplementary Table S2). The remaining gaps in the assembly were closed using the PBjelly module in the PBSuite (version 15.8.24) [34] with default settings. Thus, at the end of the first stage, the genome assembly had a contig N50 of 5.72 Mb and a scaffold N50 of 8.20 Mb (Supplementary Table S3)."

L136: due -> using
Response to comment 21
Thank you for your kindly review. We did it.

L144: Can the authors comment on the difference between the genome size based on k-mer estimates and the actual assembly size?
Response to comment 22
Thanks for your comment. This difference may be due to the large number of repeat sequences in the genome, which occupied more than 50% of the genome region. Despite the Pacbio reads were used, a lot of repeat sequences were still could not be assembled, for example in the centromeres regions. In addition, we checked the duplicated genes and found only 1.6% duplicated genes compared to 92.4% of complete BUSCO matches. This suggests major duplication did not account for this assembly.

L145: acquired -> assembled
Response to comment 23
Thank you for your kindly review. We did it.

L147ff: It would be great to actually show this, e.g. by checking what the filled gaps contain. What added value does the new assembly have.
Response to comment 24
Thanks for this comment. We made some comparisons between our new assembly and the previous assembly. we aligned our genome against the previous version using MUMMER (v4.0.0beta2) and identified a total of 2,217 insertions in our new assembly. These insertion regions were mainly located in the intergenic and repetitive regions. Further analysis showed that 6,452 gaps in the previous version that were predicted to be filled by >29.7 Mb of sequence in our new assembly. These filled gaps were mainly located in the intergenic and repetitive regions, with a small fraction of the sequence data annotated as gene regions. Also, our new assembly had a higher proportion of repeat sequences (50.82%) as compared to the previous version (46.15%); in particular, the number of LINE (long interspersed elements) transposable elements and tandem repeats was greatly increased (further details are given in the "Identification of repeat elements" section). Thus, the newly assembled genome was substantially more complete and continuous.
We added several sentences to address this comment. See also the response to your valuable comments 1 and 4.

L150: I feel that mapping ratios of Illumina data are not an adequate measure for assembly accuracy, especially given that BWA mem maps all reads very liberaly. I understand the desire to include such a number, a better (albeit not perfect) solution might be to map the Illumina data, perform a standard variant calling and quantify the number of high confidence homozygous alternative variants as a proxy to the assemblies' error rate.
Response to comment 25
Thanks for this comment. We performed a standard variant calling by Samtools, results showed that the number of homozygous SNP was 7690, occupying a proportion of 0.0004% in all SNPs, suggesting a high assembly accuracy rate. We added two sentences and one table (supplementary table S7) to address this comment.
"Genome assembly accuracy was also measured using the standard variant calling method in samtools (http://samtools.sourceforge.net/), with the command "samtools mpileup -q 20 -Q 20 -C 50 -uDEf." We found that the homozygous SNP (single nucleotide polymorphism) s comprised 0.0004% of all SNPs (7,690 of 559,048), suggesting that our genome assembly was highly accurate (Supplementary Table S7). "

L163: identified -> identify
Response to comment 26

Thank you for your kindly review. We did it.

L163: homolog -> homology
Response to comment 27
Thank you for your kindly review. We did it.

L165: I suppose the authors used all of RepBase, not only the TEs within it?
Response to comment 28
Thanks for this comment. Yes, we used all elements in the RepBase database, but not only the TEs within it. We corrected this sentence as follows.
"In the homology approach, we searched the genome for repetitive DNA elements (as listed in the Repbase database v16.02) using RepeatMasker v4.0.6 (http://www.repeatmasker.org/) [29] with the parameters "-a -nolow -no_is -norna -parallel 1" and using RepeatProteinMask (implemented in RepeatMasker).".

L168: The authors ran RepeatModeler in addition to RepeatMasker. It would be interesting to know if they detected repeat elements that are absent from RepBase and might be unknown/lineage specific.
Response to comment 29
We followed this comment and examine the repeat elements detected from RepeatModeler and RepeatMasker respectively. Results showed that several repeat elements including LINE and SINE absent from Repbase database were detected in the de novo approach (Supplementary Table S10). The total length of these repeat elements was 186,195,432bp, accounting for 6.13% of the genome, suggesting that these repeat elements may be specific for R. roxellana.

L178: Specify what database was used.
Response to comment 30
We followed this comment and added two sentences to clarify this point.
"Using BLASTN with an E-value of 1E-10, we identified four rRNAs in the R. roxellana genome homologous to human rRNAs: 28S, 18S, 5.8S, and 5S (GenBank accession numbers NR_003287.2, NR_003286.2, NR_003285.2, and NR_023363.1, respectively)."

L208ff: This sentence is very vague. Please be specific about what this comparison is about, and what "other mammals" were included and why.
Response to comment 31
Thanks for your valuable comment. Here, we want to compare the gene structure information including mRNA length, exon length, intron length and exon number between R. roxellana qinlingensis and other representative mammals. In this sentence, "other mammals" including Homo sapiens, Gorilla gorilla, Macaca mulatta, Rhinopithecus bieti, Rhinopithecus roxellana hubeiensis. We chose these mammals as human and gorilla are the most representative primates with high-quality genome, while Macaca mulatta could represent Cercopithecinae, the sister group of Colobinae consisting the sequencing species Rhinopithecus roxellana qinlingensis. As for R. bieti and R. r. hubeiensis, they were the congeneric species of R. r. qinlingensis, more importantly, the R. r. hubeiensis and R. r. qinlingensis are both the subspecies of Rhinopithecus roxellana.
We added several sentences to clarify this point.
"We also compared the gene structure, including mRNA length, exon length, intron length, and exon number, among R. roxellana qinlingensis and other representative primates (e.g., H. sapiens, G. gorilla, M. mulatta, R. bieti, and R. r, hubeiensis). We found that genome assembly patterns were similar among R. roxellana qinlingensis and the other primates (Supplementary Fig. S2).".

L211: The authors need to specify what they mean by functional annotation, and how this annotation was performed. Assigning a biological function to 22053 seems a bit high.
Response to comment 32
Thanks for this comment. Functional annotation indicated those predicted genes were annotated with the known protein databases to better understand their biological function. We performed the annotation analysis by annotating the predicted genes to the known protein database (NR, SwissProt and KEGG et al.) with the blastp command, and the best match for each gene was identified with the blast E value of 1E-5. Nearly half (10,670 of 22,497) of these genes were annotated to the predicted proteins in NR database derived from the previous genome annotation for the Rhinopithecus roxellana. And it therefore was reasonable for the assignment of 22,053 genes with biological function. We added several sentences to clarify this point.
"To better understand the biological functions of the predicted genes, we used BLASTP (with an E-value of 1E-5) to identify the best match for each predicted gene across several databases, including the NCBI

nonredundant protein database (NR v20180129), SwissProt (v20150821) [54], Kyoto Encyclopedia of Genes and Genomes (KEGG v20160503) [55], InterPro v29.0 [56], Pfam v31.0 [57], and GO (Gene Ontology)[58]. In this way, 22,053 predicted genes (98.42%) were functionally annotated (Supplementary Table S14). Nearly half (10,670 of 22,497) of these genes were annotated to the predicted proteins in NR database derived from the previous genome annotation for Rhinopithecus roxellana."

L235f: The authors present what looks like a GO-term enrichment analysis, but I can't find any mention as to how this analysis was performed.
Response to comment 33
Thanks for this comment. It is true that we performed a GO-term enrichment analysis. This analysis was performed towards the significantly expanded gene families in Rhinopithecus roxellana. We added several sentences to address this comment.
" To explore the significantly expanded gene families, we performed a GO-term enrichment analysis with EnrichPipeline32 [66, 67], using the 1,370 genes belonging to the 314 significantly expanded gene families as input, and using all predicted genes as background. We considered GO term significant if adjusted the P-value was <0.05. We found that the significantly expanded gene families were mainly associated with the hemoglobin complex, energy metabolism, and oxygen transport (Supplementary Table S16)."

L250: I can't find this repository on SRA.
Response to comment 34
Thanks for this comment. The genome assembly and other supporting data have been submitted to GigaDB database and NCBI successfully. However, we did not release them now as interest competition exist and several research groups are also working on this species. We appreciate the editor and reviewers understand the challenges in this situation and we will make related data available soon once this article is published.

Close
