# Supplementary material for: A high-quality genome assembly for the endangered golden snub-nosed monkey (Rhinopithecus roxellana)
Source: Gigascience. 2019 Aug 22;8(8):giz098. doi: 10.1093/gigascience/giz098 (PMC6705546; doi:10.1093/gigascience/giz098)
Supplement: giz098_Response_to_Reviewer_Comments_Revision_1 [file giz098_response_to_reviewer_comments_revision_1.pdf]

Author's Response To Reviewer Comments

Close

Editor Comments to Author:

Based on these reports, and my own assessment as Editor, I am pleased to inform you that it is potentially acceptable for publication in GigaScience, once you have carried out some final essential revisions suggested by our reviewers.

Response to this comment
We are delighted to see these positive comments. Following the instruction from the editor and the two reviewers, we have fully addressed all comments. See our responses below.

Reviewer Comments to Author:

Reviewer #1:
This manuscript reports a new whole genome assembly for an interesting nonhuman primate species, Rhinopithecus roxellana. This is a colobine species that has a number of unusual characteristics, including but not limited to unusual pelage, highly derived facial morphology, and social organization that is not entirely unique but is rare among Old World monkeys or other anthropoids. There are five species in the genus, and all are threatened or endangered, so there is a conservation benefit to this genome sequencing as well as basic comparative primate evolutionary genomics. There is a previously published whole genome assembly for this species, but this new assembly is a significant improvement (see below). Consequently, there are several elements of this work that make it noteworthy.
This is a revised manuscript. This version of the paper is significantly improved from the first version. Most of my comments and concerns have been satisfactorily addressed. But I do have some minor issues with this revision. I believe the authors can easily correct these remaining problems.

1) The sentence in lines 85-87 ("Genomic analyses have helped…") seems unnecessary and out of place. I suggest deleting this sentence.
Response to comment 1
Following this comment, we deleted this sentence. Please see lines 58 - 60 on page 4 for details.

2) Line 141. An N50 value is not the same as an average. The authors should indicate whether this value of 16.69 kb is an average or an N50. The latter is the preferred way to report this statistic.
Response to comment 2
Thanks for this comment. We agree that an N50 value is not the same as an average. we checked this value carefully and found that it indicated an N50 value. We changed the statement as follows (Lines 105, bottom of page 6).

"TheN50 length of the PacBio reads was 16.69 kb.".

3) Line 149: Same comment as #2
Response to comment 3
Thanks for this comment. We changed the statement as follows (Line 110 on top of page 7):
"The N50 length of the molecules used for optical mapping was 338 kb.".

4) Line 301: I would suggest adding the word "non-reference" so that it reads "We found that the homozygous non-reference SNPs comprise 0.0004%...."
Response to comment 4
Following this comment, we added "non-reference" in this sentence. (Line 201 on top of page 12)

"We found that the homozygous non-reference SNPs (single nucleotide polymorphism) comprised 0.0004% of all SNPs (7,690 of 559,048)."

5) Line 309: I think you need "also" inserted - "completeness was also measured…"
Response to comment 5
Thanks for this comment. We followed this comment and inserted "also" in this sentence. (Line 209 on page 12)

"Assembly completeness was also measured using the core eukaryotic gene (CEG)-mapping approach (CEGMA v2.5) [31]".

6) Line 317: There is at least one word missing or out of place here. Please edit.

Response to comment 6
Thanks for your valuable comment. We are sorry we made a mistake here. We revised this sentence (lines 217, bottom of page 12).

"Repeat sequences account for a large proportion of the total genome. It is thus important to identify repeat elements.".

7) Lines 336 - 342. I do not understand how the authors identify copy number variation when they did not study and do not report DNA sequences from multiple individuals. There is only one reference sequence reported in this paper. Did the authors look at copy number differences between haplotypes of that one diploid monkey? This section is very confusing to me. Either the source of the samples used for CNV analysis must be presented, or this could be deleted. Some editing is required.

Response to comment 7
Thanks for your valuable comment. It is true that analysis of one sample does not show copy number variations. We are sorry that this section was not clear enough. The term CNVs analysis, should be better termed duplications in our study. And those duplicate sequences were identified based on read depth. We added several sentences to address this comment (Lines 237-247, top of page 14):

"We also performed duplicate sequences identification analysis, which was fulfilled based on the read depth of Illumina short reads. In brief, we first mapped the Illumina short reads to the assembled genome using BWA with default parameters. Then, the sorted mapping bam file was used as input for CNVnator v0.3.3 [35], a tool targeting alterations in the read depth, with the parameters of "-unique -his 100 -stat 100 -call 100.". The obtained duplicate sequences were filtered, retaining only those where q0 was <0.5 and e-val1 was <0.05. After filtering, 676 duplicate sequences remained, with a total length of 9,198,900 bp (Supplementary Table S12). Further analysis showed that 101 duplications located at the end of scaffolds (5% of the total length in both ends). And there were 136 gene present in the duplicated regions, these genes were mainly involved in basic biological processes such as ribonucleoside binding, phosphatase activity, and protein dephosphorylation et al.".

8) Lines 389 - 391. What animal was used to obtain the heart and skin tissue for RNA sequencing? Were these tissues obtained from the same animal used for DNA sequencing and reference assembly? Please state source of tissue for RNA sequencing.

Thanks for this comment. The animal used for RNA sequencing was the same individual with DNA sequencing and reference assembly. We stated source of tissue for RNA sequencing in Lines 275 -276 (top of page 16):
"High-quality RNAs from the heart and skin tissue of the R. roxellana qinlingensis specimen (the same individual used for DNA sequencing and reference assembly) were sequenced on an Illumina Novaseq 6000 platform.".

9) I think Figure 2 would be better in the Supplement than main text. If the authors think this is important, presenting it in the supplement is fine. But I do not see that this contributes significantly to the major findings of the paper. If the authors feel strongly that it must remain in the main text, that is acceptable and I would not make an issue out of that. But I do not see the major significance beyond providing validation. No biological insight is provided by this figure.

Response to comment 9
Thanks for this valuable comment. The fig. 2 was based on the interaction frequencies between pairs of 100-kb genomic regions, which could be used to indicate the reliability of our assembly. Despite that no great biological insights provided by this figure, this figure was generated with great effort and could convert some information about our data quality, an important topic in this high quality genome work.

Thus, the Figure 2 would be better in the main text.

Reviewer #2: The revision is substantially improved and most of the concerns of the reviewers have been resolved.

CNV analysis: (line 337). I think these are better termed duplications, not CNVs, as analysis of one sample does not show whether they are copy number variable in the population. Are the duplications found at the end of contigs? Were there any gene annotations present in the duplicated sequences.
Response to this comment
Thanks for your valuable comment. It is true that analysis of one sample does not show copy number variations. We are sorry that this section was not clear enough. The term CNVs analysis, should be better termed duplications in our study. And those duplicate sequences were identified based on read depth. Further analysis of the positions of these duplicate sequences showed that 101 duplications located at the end of scaffolds. In addition, there were 136 genes present in the duplicated regions, they were mainly involved in basic biological processes. We added several sentences to address this comment (Lines 237-247, top of page 14):

"We also performed duplicate sequences identification analysis, which was fulfilled based on the read depth of Illumina short reads. In brief, we first mapped the Illumina short reads to the assembled genome using BWA with default parameters. Then, the sorted mapping bam file was used as input for CNVnator v0.3.3 [35], a tool targeting alterations in the read depth, with the parameters of "-unique -his 100 -stat 100 -call 100.". The obtained duplicate sequences were filtered, retaining only those where q0 was <0.5 and e-val1 was <0.05. After filtering, 676 duplicate sequences remained, with a total length of 9,198,900 bp (Supplementary Table S12). Further analysis showed that 101 duplications located at the end of scaffolds (5% of the total length in both ends). And there were 136 gene present in the duplicated regions, these genes were mainly involved in basic biological processes such as ribonucleoside binding, phosphatase activity, and protein dephosphorylation et al.".

Close
