# Supplementary material for: A high-quality genome assembly for the endangered golden snub-nosed monkey (Rhinopithecus roxellana)
Source: Gigascience. 2019 Aug 22;8(8):giz098. doi: 10.1093/gigascience/giz098 (PMC6705546; doi:10.1093/gigascience/giz098)
Supplement: giz098_Reviewer_1_Report_Original_Submission — Jeffrey Rogers -- 4/1/2019 Reviewed [file giz098_reviewer_1_report_original_submission.pdf]

Reviewer Report

Title: A high-quality genome assembly for the endangered golden snub-nosed monkey (Rhinopithecus roxellana)

Version: Original Submission **Date:** 4/1/2019

Reviewer name: Jeffrey Rogers

Reviewer Comments to Author:

This manuscript reports a new whole genome assembly for an interesting nonhuman primate species, Rhinopithecus roxellana. This is a colobine species that has a number of unusual characteristics, including but not limited to unusual pelage, highly derived facial morphology, and social organization that is not entirely unique but is rare among Old World monkeys or other anthropoids. There are five species in the genus, and all are threatened or endangered, so there is a conservation benefit to this genome sequencing as well as basic comparative primate evolutionary genomics. There is a previously published whole genome assembly for this species, but this new assembly is a significant improvement (see below). Consequently, there are several elements of this work that make it noteworthy.

The new assembly is based on an effective and technically advanced combination of approaches. The authors began by sequencing this genome using PacBio Sequel long reads, and assembling them using FALCON and PBjelly. The authors generated Illumina short reads and polished the PacBio/FALCON assembly with those. The authors also make use of BioNano optical mapping and 10X linked-reads to increase completeness and contiguity. Finally, Hi-C mapping is used to produce near full chromosome length scaffolds. The result is a 3.04 gigabase assembly with contig N50 of 5.72 Mb and scaffold N50 of 144.56 Mb. These statistics make this one of the most complete and highly contiguous assemblies available for any nonhuman primate (confirmed using BUSCO and CEGMA analyses). The authors then annotated this genome using a series of annotation software tools, and identified 22,497 genes.

This new genome assembly is a valuable resource for any investigator working on the genetics or genomics of Rhinopithecus. In addition, this is a high quality - high contiguity assembly, so it will be useful for laboratories working on other closely related colobines. Lastly, the authors report some initial analyses of repetitive sequences and gene family expansions and contractions using this new Rhinopithecus assembly.

While this genome sequence seems to be a valuable resource for the primate genomics community, this manuscript has a significant number of serious flaws and problems. One issue is that the quality of the grammar and text is not adequate. I realize that the authors may not be native speakers of English, and that this can be a challenge. But this manuscript needs major assistance in terms of editing before it is ready for serious consideration.

I have other specific concerns as well.

1) This is minor but having two different line numbering systems printed on the same pages causes confusion. I will use the numbers that are actually tied to specific lines in the text, rather than the more densely packed numbers that seem to just run down each page. The authors should delete the dense numbers.

2) Page 4, lines 54-56. While the social organization of Rhinopithecus roxellana is interesting and deserves more study, it seems overly optimistic for the authors to argue that production of this genome assembly will ultimately support genetic studies that make contributions to our "…understanding the behavior patterns of human society in social-anthropology." Studies of comparative social relationships and social organization are important and primates can provide information about human evolution. But this statement seems to me to be overly ambitious in terms of research outcomes.
3) There are mistakes in capitalization and spelling of words. For example, the Shennongjia Mountains are not capitalized in line 63, but "Gorillas" is incorrectly capitalized in line 75 and "Colobine" is regularly capitalized when it need not be. "Quiver" is misspelled in line 125.
4) Line 75 states the gorillas and orangutans "…have the closest genetic relationship with humans" but of course that is chimpanzees and bonobos, not gorillas and/or orangutans.
5) I think the language in line 86 is a bit too optimistic and ambitious. The authors state that this assembly "…may allow us to comprehensively understand R. roxellana…". I do not know what it would mean to "comprehensively understand" a primate species, but I do not think we are yet close to that point.
6) Page 6, line 87: It is not clear to me what the authors mean by "genetic-specific signatures of this species"?
7) Page 6, line 93-94. Was the animal used to produce the DNA for the sequencing wild-caught or captive bred at Louguantai? If captive bred, were the parents wild-caught?
8) Page 7, lines 103-105. BioNano optical mapping is a technique for using restriction enzymes to nick and label DNA at short known target sequences. The map of nicked sites is used to scaffold a genome or confirm the organization of contigs. It is not clear what the authors mean when they state that they "…acquired 463.75 Gb clean reads" from the BioNano Genomics Irys platform. There are no sequence reads generated by the Irys platform. This section does not make sense to me. Instead, the authors should present the actual results of the optical mapping in terms of the number of sites examined and the concordance between the observed BioNano map and the predicted map based on the assembled contigs and scaffolds.
9) I do not think that Figure 2 adds much to this paper. The authors used Hi-C for scaffolding, and that does provide useful data. But simply inserting a figure showing Hi-C interaction frequencies without doing any further analysis of the details of DNA-DNA interaction or characterizing the topologically associating domains provides no significant new information or insight.
10) Page 9, lines 151-152. I do not understand the sentence that begins "With a ratio number…"
11) Page 9-10, lines 152-159. Using BUSCO and CEGMA to assess the completeness of the genome assembly is a very good idea. But the authors should report not just how many BUSCO or CEGMA genes were identified, but how many were complete and unfragmented and how many were complete and fragmented.
12) Page 12, lines 226-227. What fossil calibration times were used?
13) Page 14, lines 232-235. Rhinopithecus gene families were expanded or contracted compared to what taxa? Compared to human? Compared to the ancestral primate genome? Compared to an Old World monkey outgroup?
14) I think two column headings in Table 3 are switched. I doubt that the average intron length for the

Rhinopithecus Augustus gene models is 196bp, while the average exon length for the same gene models is 5,112bp. Seems to me those two labels are probably switched.

Level of Interest

Please indicate how interesting you found the manuscript: Choose an item.

Quality of Written English

Please indicate the quality of language in the manuscript: Choose an item.

Declaration of Competing Interests

Please complete a declaration of competing interests, considering the following questions:

- Have you in the past five years received reimbursements, fees, funding, or salary from an organisation that may in any way gain or lose financially from the publication of this manuscript, either now or in the future?
- Do you hold any stocks or shares in an organisation that may in any way gain or lose financially from the publication of this manuscript, either now or in the future?
- Do you hold or are you currently applying for any patents relating to the content of the manuscript?
- Have you received reimbursements, fees, funding, or salary from an organization that holds or has applied for patents relating to the content of the manuscript?
- Do you have any other financial competing interests?
- Do you have any non-financial competing interests in relation to this paper?

If you can answer no to all of the above, write 'I declare that I have no competing interests' below. If your reply is yes to any, please give details below.

I declare that I have no competing interests

I agree to the open peer review policy of the journal. I understand that my name will be included on my report to the authors and, if the manuscript is accepted for publication, my named report including any attachments I upload will be posted on the website along with the authors' responses. I agree for my report to be made available under an Open Access Creative Commons CC-BY license (http://creativecommons.org/licenses/by/4.0/). I understand that any comments which I do not wish to be included in my named report can be included as confidential comments to the editors, which will not be published.

I agree to the open peer review policy of the journal

To further support our reviewers, we have joined with Publons, where you can gain additional credit to further highlight your hard work (see: https://publons.com/journal/530/gigascience). On publication of this paper, your review will be automatically added to Publons, you can then choose whether or not to claim your Publons credit. I understand this statement.

Yes Choose an item.
