# Supplementary material for: A high-quality genome assembly for the endangered golden snub-nosed monkey (Rhinopithecus roxellana)
Source: Gigascience. 2019 Aug 22;8(8):giz098. doi: 10.1093/gigascience/giz098 (PMC6705546; doi:10.1093/gigascience/giz098)
Supplement: giz098_Reviewer_1_Report_Revision_1 — Jeffrey Rogers -- 5/29/2019 Reviewed [file giz098_reviewer_1_report_revision_1.pdf]

Reviewer Report

Title: A high-quality genome assembly for the endangered golden snub-nosed monkey (Rhinopithecus roxellana)

Version: Revision 1 **Date:** 5/29/2019

Reviewer name: Jeffrey Rogers

Reviewer Comments to Author:

This is a revised manuscript. This version of the paper is significantly improved from the first version. Most of my comments and concerns have been satisfactorily addressed. But I do have some minor issues with this revision. I believe the authors can easily correct these remaining problems.
1) The sentence in lines 85-87 ("Genomic analyses have helped…") seems unnecessary and out of place. I suggest deleting this sentence.
2) Line 141. An N50 value is not the same as an average. The authors should indicate whether this value of 16.69 kb is an average or an N50. The latter is the preferred way to report this statistic.
3) Line 149: Same comment as #2
4) Line 301: I would suggest adding the word "non-reference" so that it reads "We found that the homozygous non-reference SNPs comprise 0.0004%...."
5) Line 309: I think you need "also" inserted - "completeness was also measured…"
6) Line 317: There is at least one word missing or out of place here. Please edit.
7) Lines 336 - 342. I do not understand how the authors identify copy number variation when they did not study and do not report DNA sequences from multiple individuals. There is only one reference sequence reported in this paper. Did the authors look at copy number differences between haplotypes of that one diploid monkey? This section is very confusing to me. Either the source of the samples used for CNV analysis must be presented, or this could be deleted. Some editing is required.
8) Lines 389 - 391. What animal was used to obtain the heart and skin tissue for RNA sequencing? Were these tissues obtained from the same animal used for DNA sequencing and reference assembly? Please state source of tissue for RNA sequencing.
9) I think Figure 2 would be better in the Supplement than main text. If the authors think this is important, presenting it in the supplement is fine. But I do not see that this contributes significantly to the major findings of the paper. If the authors feel strongly that it must remain in the main text, that is acceptable and I would not make an issue out of that. But I do not see the major significance beyond providing validation. No biological insight is provided by this figure.

Level of Interest

Please indicate how interesting you found the manuscript: Choose an item.

Quality of Written English

Please indicate the quality of language in the manuscript: Choose an item.

Declaration of Competing Interests

Please complete a declaration of competing interests, considering the following questions:

- Have you in the past five years received reimbursements, fees, funding, or salary from an organisation that may in any way gain or lose financially from the publication of this manuscript, either now or in the future?
- Do you hold any stocks or shares in an organisation that may in any way gain or lose financially from the publication of this manuscript, either now or in the future?
- Do you hold or are you currently applying for any patents relating to the content of the manuscript?
- Have you received reimbursements, fees, funding, or salary from an organization that holds or has applied for patents relating to the content of the manuscript?
- Do you have any other financial competing interests?
- Do you have any non-financial competing interests in relation to this paper?

If you can answer no to all of the above, write 'I declare that I have no competing interests' below. If your reply is yes to any, please give details below.

I declare that I have no competing interests.

I agree to the open peer review policy of the journal. I understand that my name will be included on my report to the authors and, if the manuscript is accepted for publication, my named report including any attachments I upload will be posted on the website along with the authors' responses. I agree for my report to be made available under an Open Access Creative Commons CC-BY license (http://creativecommons.org/licenses/by/4.0/). I understand that any comments which I do not wish to be included in my named report can be included as confidential comments to the editors, which will not be published.

I agree to the open peer review policy of the journal

To further support our reviewers, we have joined with Publons, where you can gain additional credit to further highlight your hard work (see: https://publons.com/journal/530/gigascience). On publication of this paper, your review will be automatically added to Publons, you can then choose whether or not to claim your Publons credit. I understand this statement.

Yes Choose an item.
