# Supplementary material for: A high-quality genome assembly for the endangered golden snub-nosed monkey (Rhinopithecus roxellana)
Source: Gigascience. 2019 Aug 22;8(8):giz098. doi: 10.1093/gigascience/giz098 (PMC6705546; doi:10.1093/gigascience/giz098)
Supplement: giz098_Reviewer_2_Report_Original_Submission — Jeff Kidd -- 4/2/2019 Reviewed [file giz098_reviewer_2_report_original_submission.pdf]

**Reviewer Report**

**Title: A high-quality genome assembly for the endangered golden snub-nosed monkey (Rhinopithecus roxellana)**

**Version: Original Submission** **Date:** 4/2/2019

**Reviewer name: Jeff Kidd**

**Reviewer Comments to Author:**

The authors present an assembly of golden snub-nosed monkey using a range of sequencing technologies, including long read sequencing. Overall the manuscript is mostly clear to follow and the assembly approaches are standard and appear to be well performed. A very large amount of data was generated, although the methods are very short and some details are lacking, it appears that standard and appropriate assembly approaches were used. Some key details about the generated data are missing, and there are some additional analyses that, if compelted, would greatly improve the manuscript.
I could not find descriptions of the characteristics of the generated data, particularly average/n50 length of Pacbio reads, molecule size of the optical mapping and of 10X data. These are key parameters that should be reported.
Line 104 The description of the Bionano data should be clarified. I am not sure that "reads" is the right term for data from this optical mapping platform. Same for term 'sequence coverage' for optical mapping data in Table 1.
The manuscript would benefit from some comparison of how much better the gene annotation is relative to previous assembly, but this and other biological/comparative analyses may be beyond the scope of this report.
From Supplementary Tables S2-3, it seems that the largest increase in n50 scaffold length came from 10X linked read data, not from the bionano optical map. I do not think this is expected, given that optical map data should provide very long range information. The manuscript would be clearer for the reader if some description for why such a gain was found from 10X data was described, and if such results are typical.
Standard repeat masker, gene prediction, and other analysis is performed. The manuscript would be strengthened by also a consideration of duplicated sequences, which could be identified based on Illumina sequence data read depth. This may be beyond scope of this report, but could be considered.
Has the assembly itself been submitted to proper databases and repositories (such as Genbank)? I could not find this listed, only the raw data.
In table 2 and others, what does the 'number' column mean? For example, are there 151 contigs >= to the N50 length of 5.7mb? The meaning of the columns in the tables should be clearly explained.
The legend for figure 2 is not adequate. What does the color scale signify? What is the reader supposed to conclude from the figure?

**Level of Interest**

Please indicate how interesting you found the manuscript: Choose an item.

**Quality of Written English**

Please indicate the quality of language in the manuscript: Choose an item.

**Declaration of Competing Interests**

Please complete a declaration of competing interests, considering the following questions:

- Have you in the past five years received reimbursements, fees, funding, or salary from an organisation that may in any way gain or lose financially from the publication of this manuscript, either now or in the future?
- Do you hold any stocks or shares in an organisation that may in any way gain or lose financially from the publication of this manuscript, either now or in the future?
- Do you hold or are you currently applying for any patents relating to the content of the manuscript?
- Have you received reimbursements, fees, funding, or salary from an organization that holds or has applied for patents relating to the content of the manuscript?
- Do you have any other financial competing interests?
- Do you have any non-financial competing interests in relation to this paper?

If you can answer no to all of the above, write 'I declare that I have no competing interests' below. If your reply is yes to any, please give details below.

i declare that i have no competing interests

I agree to the open peer review policy of the journal. I understand that my name will be included on my report to the authors and, if the manuscript is accepted for publication, my named report including any attachments I upload will be posted on the website along with the authors' responses. I agree for my report to be made available under an Open Access Creative Commons CC-BY license (http://creativecommons.org/licenses/by/4.0/). I understand that any comments which I do not wish to be included in my named report can be included as confidential comments to the editors, which will not be published.

I agree to the open peer review policy of the journal

To further support our reviewers, we have joined with Publons, where you can gain additional credit to further highlight your hard work (see: https://publons.com/journal/530/gigascience). On publication of this paper, your review will be automatically added to Publons, you can then choose whether or not to claim your Publons credit. I understand this statement.

Yes Choose an item.
