# Supplementary material for: A high-quality genome assembly for the endangered golden snub-nosed monkey (Rhinopithecus roxellana)
Source: Gigascience. 2019 Aug 22;8(8):giz098. doi: 10.1093/gigascience/giz098 (PMC6705546; doi:10.1093/gigascience/giz098)
Supplement: giz098_Reviewer_3_Report_Original_Submission — Tomas Marques-Bonet -- 4/5/2019 Reviewed [file giz098_reviewer_3_report_original_submission.pdf]

Reviewer Report

Title: A high-quality genome assembly for the endangered golden snub-nosed monkey (Rhinopithecus roxellana)

Version: Original Submission **Date:** 4/5/2019

Reviewer name: Tomas Marques-Bonet

Reviewer Comments to Author:

Wang, Wu et al. have produced a high-quality reference genome assembly for the emblematic golden snub-nosed monkey. The authors used a combination of long PacBio reads, 10-X linked reads, Hi-C contact maps, BioNano Optical maps, and Illumina paired end sequences, all of which were sequenced to a very high coverage. The resulting assembly has very high continuity and given the combination of different sequencing strategies essentially gives as good of an assembly as current methods can produce. The authors have used a state-of-the-art approach to produce their assembly, and the applied methodology is appropriate. The authors have also produced a gene annotation based on homology to other species, as well as expression data. The assembly provides a valuable genomic resource to study snub-nosed monkeys specifically, and Asian colobines in general.

General comments:

R. roxellana already has a genome assembly available, as the authors note in the manuscript. However, there is no comparison at all beyond a contig and scaffold N50. It would strengthen the manuscript if the authors could provide some comparisons to the previous assembly, e.g: A comparative repeat analysis, or what specific regions of the assembly were absent in the previous version, what do they contain, how many gaps were filled, how many of the gene family expansions/contractions are only detectable with the high quality assembly etc.

The authors use several different software packages for their analysis. The inclusions of version numbers for the software packages they used seems somewhat arbitrary. Furthermore, no parameter sets apart from "default parameters" are ever presented. Both package versions and parameter settings should absolutely be included, otherwise the methods of the study are not properly understandable. In its current state, I feel the methodological aspects of the manuscript need to be expanded.

The manuscript will benefit from language editing, as at several points the phrasing is somewhat confusing.

Specific comments:

L19, L68, L80: The claim of "incompleteness" or "greatly improved" is not backed by a proper comparison to the previous assembly.

L22: Genetic-specific signatures is awkwardly phrased.

L25: Technology, not technique

L57: This sentence is vague, please be specific about what these studies have looked at. The term research-hotspot for this species might be a stretch.

L58f: This sentence needs rephrasing. What are the groups?

L60: differentiate -> be distinguished

L63: Was there more than one assembly before this study?
L71f: This sentence needs rephrasing; it is not clear to me what the authors want to say.
L74,L78: Please be specific with respect to the sequencing technology. "High quality" is subjective and changes with sequencing technologies, so arguing that no "high quality assembly of R. roxellana has been reported" is debatable.
L75: Ref 15. Also includes an assembly for the Chimpanzee, which is closer to Human than either Gorilla or the Orangutan. 'Widely' should be omitted in this sentence.
L76: "A lot of new findings" is vague, please specify the specific advantages of the new assemblies.
L81: Through combined -> by combining
L110: Cutadapter -> Cutadapt
L115ff: The value for Kerror was omitted.
L125: Quier -> Quiver
L130: To the best of my knowledge, PBJelly doesn't know how to deal with phased assemblies. All previous assembly steps (Falcon, Quiver, Pilon, sspace) also do not talk about phasing information. Please clarify how phasing was dealt with or maintained at this point.
L130: The authors only mention the scaffold N50 after gap-filling. I see the contig N50 is mentioned in the supplementary, but I cannot find the contig N50 of the base assembly before gap-filling anywhere. It would be worth to mention it to understand the relative contributions of additional steps.
L136: due -> using
L144: Can the authors comment on the difference between the genome size based on k-mer estimates and the actual assembly size?
L145: acquired -> assembled
L147ff: It would be great to actually show this, e.g. by checking what the filled gaps contain. What added value does the new assembly have.
L150: I feel that mapping ratios of Illumina data are not an adequate measure for assembly accuracy, especially given that BWA mem maps all reads very liberaly. I understand the desire to include such a number, a better (albeit not perfect) solution might be to map the Illumina data, perform a standard variant calling and quantify the number of high confidence homozygous alternative variants as a proxy to the assemblies' error rate.
L163: identified -> identify
L163: homolog -> homology
L165: I suppose the authors used all of RepBase, not only the TEs within it?
L168: The authors ran RepeatModeler in addition to RepeatMasker. It would be interesting to know if they detected repeat elements that are absent from RepBase and might be unknown/lineage specific.
L178: Specify what database was used.
L208ff: This sentence is very vague. Please be specific about what this comparison is about, and what "other mammals" were included and why.
L211: The authors need to specify what they mean by functional annotation, and how this annotation was performed. Assigning a biological function to 22053 seems a bit high.
L235f: The authors present what looks like a GO-term enrichment analysis, but I can't find any mention as to how this analysis was performed.

L250: I can't find this repository on SRA.
Lukas Kuderna and Tomas Marques-Bonet

Level of Interest

Please indicate how interesting you found the manuscript: Choose an item.

Quality of Written English

Please indicate the quality of language in the manuscript: Choose an item.

Declaration of Competing Interests

Please complete a declaration of competing interests, considering the following questions:

- Have you in the past five years received reimbursements, fees, funding, or salary from an organisation that may in any way gain or lose financially from the publication of this manuscript, either now or in the future?
- Do you hold any stocks or shares in an organisation that may in any way gain or lose financially from the publication of this manuscript, either now or in the future?
- Do you hold or are you currently applying for any patents relating to the content of the manuscript?
- Have you received reimbursements, fees, funding, or salary from an organization that holds or has applied for patents relating to the content of the manuscript?
- Do you have any other financial competing interests?
- Do you have any non-financial competing interests in relation to this paper?

If you can answer no to all of the above, write 'I declare that I have no competing interests' below. If your reply is yes to any, please give details below.

I declare that I have no competing interests

I agree to the open peer review policy of the journal. I understand that my name will be included on my report to the authors and, if the manuscript is accepted for publication, my named report including any attachments I upload will be posted on the website along with the authors' responses. I agree for my report to be made available under an Open Access Creative Commons CC-BY license (http://creativecommons.org/licenses/by/4.0/). I understand that any comments which I do not wish to be included in my named report can be included as confidential comments to the editors, which will not be published.

I agree to the open peer review policy of the journal

To further support our reviewers, we have joined with Publons, where you can gain additional credit to further highlight your hard work (see: https://publons.com/journal/530/gigascience). On publication of this paper, your review will be automatically added to Publons, you can then choose whether or not to claim your Publons credit. I understand this statement.

Yes Choose an item.
